# Contact-Dependent Granzyme B-Mediated Cytotoxicity of Th17-Polarized Cells Toward Human Oligodendrocytes

**DOI:** 10.3389/fimmu.2022.850616

**Published:** 2022-04-11

**Authors:** Hélène Jamann, Qiao-Ling Cui, Haritha L. Desu, Florian Pernin, Olivier Tastet, Alexandre Halaweh, Negar Farzam-kia, Victoria Hannah Mamane, Oumarou Ouédraogo, Aurélie Cleret-Buhot, Audrey Daigneault, Renaud Balthazard, Wendy Klement, Florent Lemaître, Nathalie Arbour, Jack Antel, Jo Anne Stratton, Catherine Larochelle

**Affiliations:** ^1^Centre de Recherche du Centre Hospitalier de l’Université de Montréal (CRCHUM), Université de Montréal, Montreal, QC, Canada; ^2^Department of Neurosciences, Faculty of Medicine, Université de Montréal, Montreal, QC, Canada; ^3^Department of Neurology and Neurosurgery, Montreal Neurological Institute, McGill University, Montreal, QC, Canada; ^4^Department of Microbiology, Immunology and Infectiology, Faculty of Medicine, Université de Montréal, Montreal, QC, Canada

**Keywords:** human oligodendrocytes, neuroinflammation, multiple sclerosis, Th17 cells, granzyme B, CD4 T lymphocytes

## Abstract

Multiple sclerosis (MS) is characterized by the loss of myelin and of myelin-producing oligodendrocytes (OLs) in the central nervous system (CNS). Pro-inflammatory CD4^+^ Th17 cells are considered pathogenic in MS and are harmful to OLs. We investigated the mechanisms driving human CD4^+^ T cell-mediated OL cell death. Using fluorescent and brightfield *in vitro* live imaging, we found that compared to Th2-polarized cells, Th17-polarized cells show greater interactions with primary human OLs and human oligodendrocytic cell line MO3.13, displaying longer duration of contact, lower mean speed, and higher rate of vesicle-like structure formation at the sites of contact. Using single-cell RNA sequencing, we assessed the transcriptomic profile of primary human OLs and Th17-polarized cells in direct contact or separated by an insert. We showed that upon close interaction, OLs upregulate the expression of mRNA coding for chemokines and antioxidant/anti-apoptotic molecules, while Th17-polarized cells upregulate the expression of mRNA coding for chemokines and pro-inflammatory cytokines such as IL-17A, IFN-γ, and granzyme B. We found that secretion of CCL3, CXCL10, IFN-γ, TNFα, and granzyme B is induced upon direct contact in cocultures of human Th17-polarized cells with human OLs. In addition, we validated by flow cytometry and immunofluorescence that granzyme B levels are upregulated in Th17-polarized compared to Th2-polarized cells and are even higher in Th17-polarized cells upon direct contact with OLs or MO3.13 cells compared to Th17-polarized cells separated from OLs by an insert. Moreover, granzyme B is detected in OLs and MO3.13 cells following direct contact with Th17-polarized cells, suggesting the release of granzyme B from Th17-polarized cells into OLs/MO3.13 cells. To confirm granzyme B–mediated cytotoxicity toward OLs, we showed that recombinant human granzyme B can induce OLs and MO3.13 cell death. Furthermore, pretreatment of Th17-polarized cells with a reversible granzyme B blocker (Ac-IEPD-CHO) or a natural granzyme B blocker (serpina3N) improved survival of MO3.13 cells upon coculture with Th17 cells. In conclusion, we showed that human Th17-polarized cells form biologically significant contacts with human OLs and exert direct toxicity by releasing granzyme B.

## 1 Introduction

Multiple sclerosis (MS) is a neuroinflammatory disease characterized by invasion of immune cells into the CNS, leading to demyelination and to oligodendrocytes (OLs) and neuroaxonal damage/death (1). OL alterations in MS have been investigated notably using single-cell RNA sequencing (2); nonetheless, the mechanisms underlying OL death in MS remain poorly understood. As mature OLs express major histocompatibility complex (MHC) I in inflammatory conditions, these cells can be recognized and targeted by CD8 T cells, which are present in MS plaques (3, 4). Indeed, activated CD8 T cells can mediate cytotoxicity toward OLs *in vitro* by different mechanisms including MHC I-restricted cytotoxicity (5–8). CD4^+^ T cells, and more specifically Th17 cells, are key drivers of CNS neuroinflammatory processes in MS (9–12) and its animal model experimental autoimmune encephalitis (EAE) (11, 13). Pro-inflammatory Th17 cells are increased in the peripheral blood and cerebrospinal fluid of active MS patients, notably during relapses (14, 15), and are found in active MS lesions (16). However, as OLs do not express MHC II, direct interactions between Th17 cells and OLs in MS and EAE have remained underexplored.

We have recently reported that Th17 cells can form prolonged contact with OLs *in vivo* in EAE (17). In addition, we established that Th17-polarized cells induce both OL process damage and OL cell death in mouse and human systems. While we identified the contribution of CD29-triggered glutamate secretion by Th17-polarized cells to OL process injury and myelination impairment, glutamate did not induce death of human OLs in primary culture (17). Human OLs are highly resistant to induction of programmed cell death upon exposure to stressors such as high doses of tumor necrosis factor alpha (TNFα) or glutamate, or deprivation of glucose (17–20). Using human OLs in primary culture and human oligodendrocytic cell line MO3.13 (17, 21), we therefore aimed to understand the mechanisms implicated in Th17-mediated cell death of human OLs. In the present work, comparing the real-time interaction of human Th2- and Th17-polarized cells with OLs, we found that Th17-polarized cells form longer contacts with human OLs than their Th2 counterparts. These interactions are associated with the presence of vesicle-like structures at the site of contact between OLs and Th17-polarized cells. Moreover, upon contact with OLs, Th17-polarized cells modified their mRNA expression of genes related to regulation of metabolic processes, to interferon-gamma (IFNγ) signaling, and to expression and secretion of pro-inflammatory cytokines and cytotoxic molecules, in particular granzyme B, a key cytotoxic molecule (22–24). We observed that human Th17-polarized cells expressed up to five times greater levels of granzyme B compared to Th2-polarized cells and that such levels were further upregulated upon direct contact with OLs. In addition, exposure to recombinant human granzyme B decreased the survival of OLs, while blocking Th17-polarized cell-derived granzyme B protected OLs from T cell-mediated cytotoxicity. Finally, CD4^+^ T cells expressing granzyme B were detected in MS lesions. Overall, our data support the notion that human OLs are susceptible to granzyme B secreted by Th17-polarized cells in close contact and that this mechanism could contribute to loss of OLs in MS lesions.

## 2 Material and Methods

### 2.1 OLs and MO3.13 Cell Culture

Human OLs in primary culture were isolated from fresh non-pathological brain tissue from surgical resection for non-tumoral intractable focal epilepsy as previously published (18) and in accordance with the guidelines set by the Biomedical Ethics Unit of McGill University (Protocol ANTJ 1988/3). Briefly, the tissue was cleaned from blood vessels, digested with a DNAse (Sigma, Cat#11284932001, St. Louis, MO, USA)/trypsin (Gibco, Cat# 15090-046, Grand Island, NY, USA) cocktail and smooshed to obtain a cell suspension. Myelin and red blood cells were removed using a 33% Percoll ultracentrifugation. Cells were plated and the day after floating cells (oligodendrocytes) were harvested and plated on poly-L-lysine (2 μg/ml, Sigma, Cat# P1399) and ECM (1:200, Sigma, Cat# E1270) coated wells. During the first 4 days, OLs were allowed to grow in enriched cell medium, composed of DMEM-F12 (Sigma, Cat# D8437), 2 mM glutamine (GlutaMAX, Thermo Fisher, Cat# 35050-061, Waltham, MA, USA), 100 U/ml penicillin/100 µg/ml streptomycin (Invitrogen, Carlsbad, CA, USA, Cat# 15140122), 2 ng/ml T3 (Sigma-Aldrich, Cat# T5516), 1/50 N1 supplement (Sigma, Cat# N6530), 1/50 B27 (Invitrogen, Cat# 12587010), 10 ng/ml bFGF (Sigma-Aldrich, Cat# F0291), and 10 ng/ml PDGF-AA (Sigma-Aldrich, Cat# P3076). After cell process extension, fresh medium without bFGF and PDGF-AA was used. OLs were used for experiments at D8-D10 *in vitro*. Information on tissue is provided in **Table 1**.

**Table 1:** Human primary oligodendrocytes characterization and use.

Tissue information			Experimental use				
Prep	Age	Sex	Live imaging	Coculture FACS^*a*^	Coculture IF^*b*^	scRNA sequencing	Activated granzyme B assay
1	39	M				X	
2	9	M	X		X		
3	4	M	X		X		
4	31	F		X			
5	57	M	X	X	X		
6	20	M		X			
7	35	M	X	X	X		
8	16	M					X
9	16	F					X
10	13	F					X
11	2	M					X
12	68	M		X			
13	10	M		X			

a Flow cytometry.

b Immunofluorescence.

The human oligodendrocytic cell line MO3.13 was cryopreserved in DMEM 40% FBS/20% DMSO. For culture, after gentle thawing at room temperature (RT) in DMEM, MO3.13 cells were plated in DMEM supplemented with 10% FBS, glutamine, and penicillin/streptomycin. When confluent, MO3.13 cells were harvested using warm diluted trypsin 1× (Life Technologies, Cat# 15090-046, Carlsbad, CA, USA) in PBS before plating.

### 2.2 T Cell Polarization

Written informed consent was obtained from every donor prior to sample collection (CRCHUM ethic committee approval numbers SL05.022, SL05.023, and BH07.001). Peripheral venous blood from healthy donors was obtained and used following ethical guidelines from the CRCHUM (Nagano 19.088). Peripheral blood mononuclear cells (PBMCs) were isolated by gradient centrifugation (Ficoll) as previously published (12). Memory CD4^+^ T cells were magnetically sorted (negative selection) according to the manufacturer’s instructions (Miltenyi, Cat# 130-091-893, Bergisch Gladbach, Germany). Memory CD4^+^ T cells were cultured in 6-well plates coated with αCD3 clone OKT3 (2.5 μg/ml, eBioscience, Cat# 16-0037-85, San Diego, CA, USA) in X-VIVO medium (Lonza, Cat# 04-418Q, Walkersville, MD, USA) with αCD28 clone NA/L3 (2 μg/ml, BD Bioscience, Cat# 555725, Franklin Lakes, NJ, USA) and αIFNγ (5 μg/ml, Bio X Cell, Cat# BE0235, Lebanon, NH, USA). For Th2 polarization, human recombinant IL-4 (200 ng/ml, R&D Systems, Cat# 204-IL-020) and αIL-12 (5 μg/ml, Bio X Cell, Cat# BE024) were added, whereas for Th17 polarization cells were cultured with human recombinant IL-23 (25 ng/ml, R&D Systems, Cat# 1290-IL-010, Minneapolis, MN, USA) and with αIL-4 (5 μg/ml, Bio X Cell, Cat# BE0240) as previously published (12). On day 2 of culture, IL-2 (20 U/ml, R&D Systems Cat# 202-IL-050) was added to Th2-polarized cell medium and on days 2 and 3 of culture, IL-23 (25 ng/ml) was added to Th17-polarized cell medium. Polarized T cells were used at after 6 days in culture (D6) for experiments. Representative flow cytometry plots showing the expression of IL-17 and IFNγ are provided in **Supplementary Figure 1**.

### 2.3 Live Imaging

#### 2.3.1 Cell Staining

The oligodendrocytic cells MO3.13 were stained using CellTracker Orange CMRA (Thermo Fisher, Cat# C34551) 4 days prior to coculture. Briefly, cells were harvested and resuspended at 10^6^ cells/ml in DMEM without red phenol (Wisent Bio Products, Cat# 319-050-CL, St-Bruno, Quebec, Canada) and stained with 30 mM CellTracker Orange CMRA for 15 min at 37°C. Staining was quenched by adding FBS for 1 min. After one wash, cells were resuspended in live imaging media (DMEM without red phenol, HEPES 20 mM, 5% FBS, 1% P/S) and plated at 200,000 cells/dish in IbiTreat µ-dishes (35 mm, ibidi, Cat# 81156, Gräfelfing, Germany). Mature primary OLs (D8-10) plated in IbiTreat Quad µ-dishes (35 mm, ibidi, Cat# 80416) were stained on the day of experiment using a live cell-permeable fluorescent probe binding to microtubules (ViaFluor 647 live cell microtubule stain, Biotium, Cat# 70063, Fremont, CA, USA). OLs were incubated with the dye diluted to 2.5× in warm live imaging media for 30 min at 37°C. OLs in primary culture cannot be washed extensively; therefore, the remaining dye was able to stain T cells during the coculture, as T cells also contain polymerized microtubules.

To distinguish polarized T cells from human oligodendrocytic MO3.13 cells and primary OLs, Th17-polarized and Th2-polarized cells (D6) were stained on the day of the acquisition using CellTracker Green CMFDA (Thermo Fisher, Cat# C2925) at 5 mM following the same protocol described above for the CellTracker Orange CMRA. Protocols are summarized in **Supplementary Figure 2**.

#### 2.3.2 Live Imaging Acquisition

Imaging of coculture of T cells (Th2/Th17-polarized cells) with CNS-derived cells (MO3.13 or primary OLs) was performed as previously published by Lemaître et al. (25). Polarized T cells were added at a 1:1 ratio to MO3.13 or primary OLs and immediately subjected to live imaging within the first 10 min of coculture. Cocultures in 35-mm µdishes were placed in a 37°C, 5% CO_2_ top chamber (Tokai Hit, Fujinomiya, Japan). The Fluor 40x/1.3 Oil M27 objective (free WD 0.16 mm; Zeiss, Toronto, Canada) (for fluorescent acquisition) or the alphaPlan Apo 100X/1.46 Oil DICIII objective (free WD 0.11 mm; Zeiss) (for bright field acquisition) was warmed at 37°C using a lens heater before imaging. We used a Zeiss Axio Observer Z1 Yokogawa CSU-X1 spinning disk confocal microscope equipped with a motorized stage, Piezo objectives, and an Evolve EMCDD (512 × 512, 16-bit) monochrome camera (Teledyne Photometrics, Tucson, AZ, USA). During imaging, the Definite Focus module (Zeiss) was used to minimize Z drift. For fluorescent acquisitions, cells were illuminated with 488- and 561-nm (MO3.13—T-cell coculture) or 488- and 635-nm (OLs—T-cell coculture) solid-state lasers. For fluorescent–bright-field acquisition, cells were illuminated with a 488-nm laser. Potential cell phototoxicity was reduced by setting laser power at a maximum of 10% and filtering the total power through a beam-splitter dichroic mirror (20% of the light source emission at the objective). Depending on experiment, acquisitions were programmed on a 10-μm stack as the following: for fluorescent and bright-field acquisitions on OLs, four areas (two on Th2 and two on Th17-polarized coculture wells) were acquired within a 1.5-min interval during 2 (fluorescence) or 1 (bright field) hour. Concerning MO3.13-, Th2-, and Th17-polarized cocultures, 2h-long fluorescence videos were acquired with two different positions for each T-cell subtype acquired and analyzed. For Th17-polarized MO3.13 cells cocultured in bright field, or Th2/Th17-polarized MO3.13 cocultured in fluorescence/bright field acquisitions, four positions were acquired during 15 min with a 0.5-s interval.

#### 2.3.3 Post-Acquisition Processing for Live Imaging With Fluorescence

To improve resolution and contrast, AutoQuant X3 software (Media Cybernetics, Rockville, MD, USA) was used to perform 3D blind deconvolution respecting specificities of dyes, lasers, and physical properties of the microscope. Settings of the deconvolution were 5 iterations and medium noise.

#### 2.3.4 Live Imaging Analysis

Imaris software (v9.6 Bitplane, Oxford Instruments Group, RRID:SCR_007370, http://www.bitplane.com/imaris/imaris, Concord, MA, USA) was used to analyze live-imaging experiments. To track T-cell movements from fluorescent acquisitions, the Imaris spot object algorithm was performed, using the source channel 488 nm to create spots specifically on T cells. All cell tracks were manually checked and corrected if needed. Depending on experiments and the video’s quality, around 10–30 tracks were extracted for each video. For characterization of T-cell behavior, the videos were separated into 30-min periods. Tracks lasting ≥20 min were selected for analysis. Track, shape, and general movement were used to classify the dominant behavior of each T cell for the 30-min period (static, crawling, scanning, no contact). Z-slices and 3D rotations were used to analyze contact duration with OLs or MO3.13 cells (no contact, less than 5 min, between 5 and 10 min, more than 10 min) as previously done (17). During a 30-min period, the behavior or type of contact that lasted for the longest time over the 30-min period was attributed if a given cell displayed more than one type of behavior or contact. Analysis pipeline and examples of cell behaviors are presented in **Supplementary Figure 2**. Static contacts corresponded to cells staying in contact with the same site of the OL, without shape deformation (round shape with flattened membrane at the adhesion surface), evoking the morphology observed during immunological synapse formation (25) and CD4^+^ T-cell calcium influx (26, 27). Crawling cells were defined as T cells moving on OLs (usually in a straight line) with slight shape deformation, whereas scanning cells displayed highly deformed shape (elongated) and were highly mobile on OLs. These last two contact behaviors suggested low calcium influx in CD4^+^ T cells (26, 27) and a low-affinity contact between polarized T cells and OLs and thus could be reminiscent of kinapses (25, 28, 29).

Bright-field acquisitions of T cell-MO3.13/OL cocultures were used to quantify the number of vesicle-like structures in areas of MO3.13 or OLs in direct contact with T cells *versus* areas without direct contact with T cells. MO3.13 or OLs in contact with T cells visualized for at least 50 min were included. Areas of interest were delimited manually using the surface module (Imaris). Areas with or without contact were created on the same OL/MO3.13 cell and next to each other. Vesicle-like structures were defined as bright spots of size 200 to 600 nm within the MO3.13 cell/OL and manually counted in Imaris using the spot module.

### 2.4 Flow Cytometry

At D6, polarized T cells were harvested, washed, and added to OLs (ratio 1:2.5, OL: T cells) or MO3.13 cells (100,000 cells per well, ratio 1:10, MO3.13 cell: T cells) in fresh media in 24-well plates. To prevent direct contact of T cells with OLs or MO3.13 cells, T cells were added to 0.4-μm pore-sized Boyden chambers (“insert” condition). After coculture, cells were detached using PBS EDTA (10 min 37°C) and transferred into 96-well plates for flow cytometry (FACS) staining. To assess the expression of cytokines, cells were stimulated with phorbol 12‐myristate 13‐acetate (PMA) and ionomycin in the presence of brefeldin A for 4 h at 37°C as previously published (12, 30). For FACS staining, cells were incubated with LIVE/DEAD fixable Aqua Dead Cell stain (Invitrogen, Cat# L34957) for 30 min at 4°C. After washing and non-specific site blocking with mouse IgG (60 μg/ml, Thermo Fisher, Cat# 10400C), cells were incubated with O4-APC (Miltenyi, Cat# 130-119-897), CD4-PerCP-Cy5.5 (BD Biosciences, Cat# 560650), and HLA-DR-AlexaFluor700 (BD Biosciences, Cat# 561016) for 20 min at 4°C. For intracellular staining, permeabilization was performed using the Fixation/Permeabilization solution kit (BD Biosciences, Cat# 55714) following the manufacturer’s instruction before incubation with the granzyme B-AlexaFluor700 antibody (BD Biosciences, Cat# 561016). Appropriate isotype controls were used to assess non-specific fluorescence signal. Samples were acquired on an LSR II analysis FACS machine (BS Biosciences) and analyzed using FlowJo Software (FlowJo, RRID:SCR_008520, https://www.flowjo.com/solutions/flowjo).

### 2.5 Immunofluorescence (IF)

#### 2.5.1 Cocultures and Cytospins

After fixation with PFA 4%, permeabilization with Triton 1%, and blocking with donkey serum, cells were incubated overnight at 4°C with the following primary antibodies in 3% donkey serum: rabbit anti-human NogoA (1/100, EMD Millipore, Cat# AB5664P) and/or mouse anti-human granzyme B (1/20, R&D, Cat# MAB2906). After washing, cells were stained with the appropriate secondary antibodies: donkey anti-rabbit Rhodamine Red™-X (RRX, 1/400, Jackson ImmunoResearch, Cat# 711-295-152, West Grove, PA, USA) and donkey anti-mouse AlexaFluor488 (1/400, Invitrogen, Cat# 21202). For cytopsins, a staining step with mouse anti-human CD4-AlexaFluor 647 (Abcam, Cat# ab196147, Cambridge, MA, USA) was added. Finally, nuclei were stained with DAPI (1/1,000, Sigma-Aldrich, Cat# D9542) and cells were mounted on slides with Prolong Gold Antifade (Thermo Fisher, Cat# P36930). As T cells were loaded with CellTracker Green and MO3.13 with CellTracker Orange CMRA, no additional staining was required for visualization. Images were acquired on a Leica SP5 MP confocal machine.

#### 2.5.2 Frozen Sections

Human brain sections from two different MS patients (female RRMS 49 y.o. and female 33 y.o. RRMS) were stained following established protocols (12, 30, 31). After fixation with acetone and ethanol 70% at -20°C and blocking with 10% mixed donkey/goat serum, sections were incubated for 1 h at 20°C with primary antibodies diluted in 3% donkey serum: mouse anti-human granzyme-B (1/20, R&D Systems, Cat#MAB2906) and rabbit anti-human NogoA (1/100, EndMillipore, Cat# AB5664P). Adequate secondary antibodies for these antibodies were used: donkey rabbit RRX (1:500, Jackson ImmunoResearch, Cat#711-295-152) and goat anti-mouse AlexaFluor635 (1:250, Invitrogen, Cat#A31574). Then, sections were blocked again in 10% rabbit serum and stained using mouse anti-human CD4-FITC antibody (1:50, BD Biosciences, Cat#555346), followed by a secondary antibody rabbit anti-FITC (1:50, Invitrogen, Cat#A11090). Finally, nuclei were stained using DAPI. Sections were mounted on slides with Prolong Gold Antifade.

#### 2.5.3 Confocal Microscopy

Confocal images were acquired with a Leica TCS-SP5 inverted microscope using a HCX PL APO CS 63x/1.4 Oil UV objective with the LAS AF software. Excitation was performed using a 405-nm diode laser for DAPI, a 488-nm line of an Argon laser for AF488/CellTracker Green, a 561-nm DPSS laser for CellTracker CMRA Orange, and a 633-nm HeNe laser for AF647. The detection bandwidth was 415–492 nm for DAPI using a PMT, 500–558 nm for CellTracker Green/AF488 using an HyD under the Standard mode, 571–608 nm for CellTracker CMRA/RRX using a HyD under the Standard mode, and 643–713 nm for AF647 using a PMT. A sequential acquisition consisting of CellTracker Green/AF488 and AF647 in sequence 1, CellTracker Orange CMRA/RRX in sequence 2, and DAPI in sequence 3 was performed to avoid cross-excitations and cross-emissions between the different dyes. Images were acquired at a 400-Hz scan speed and final images are 8 bits, 512 × 512 (axial pixel size of 481 nm). Z-stacks were performed with a 1- to 2.3-µm step size for volume rendering. For some images, a zoom 3 to 4 was applied (axial pixel size of 160 to 120 nm).

#### 2.5.4 Image Analysis

For granzyme B fluorescence quantification, a first step for brightness/contrast adjustment and Z-stack projection was performed with a macro on ImageJ (FIJI, RRID: SCR_002285, http://fiji.sc) (32). To quantify CD4^+^granzyme B^+^ cells and median fluorescence intensity in positive cells, the software CellProfiler was used (CellProfiler Image Analysis Software, RRID: SCR_007358, http://cellprofiler.org) (33). To compare the intensity of the granzyme B signal in areas of Th17-polarized cells in direct contact with OLs versus areas without direct contact with OLs, areas of interest were delimited manually using ImageJ. Areas with or without contact were created on the same Th17-polarized cell and next to each other.

### 2.6 Single-Cell RNA-Sequencing

#### 2.6.1 Experiment

One preparation of human OLs in primary culture was plated at 400,000 cells per well in a 24-well plate to obtain sufficient numbers for sequencing and matured for 10 days *in vitro*. Th17-polarized cells were added to OLs (ratio 2:1) directly (contact condition) or separated by a Boyden chamber (insert condition, “no contact”) with a porous membrane of 0.4-μm size (VWR, Cat# 35495). After 12 h of coculture, cells were detached using trypsin, harvested in 0.04% BSA, and passed through a cell strainer (Falcon, Cat# 352340) to obtain single-cell suspension. Finally, single-cell RNA sequencing libraries were created using 10X Chromium v2.0 for single-cell RNA sequencing acquisition with the Illumina NovaSeq600 SP PE150 at the McGill University and Génome Québec, using the droplet sequencing approach (10X Chromium).

#### 2.6.2 Analysis

The cellranger (v3; https://github.com/10XGenomics/cellranger) was used for demultiplexing and gene expression quantification. Reads were aligned to the GRCh38 release of the reference human genome. Downstream processing and analysis were performed in R with the Seurat package (34) (v3; https://satijalab.org/seurat/). Normalization of gene expression was done with the SCTransform workflow. To assign biological relevance to T-cell subpopulations, Gene Set Enrichment Analysis (GSEA) was done using a Fisher test onto the Gene Ontology Biological Processes 2021 annotation. The sets of markers for each population were obtained through the FindAllMarkers function from Seurat, using the MAST method. Differentially expressed genes (DEGs) between the two studied conditions (contact versus insert) were found with the FindMarkers function using the DESeq2 method across each subpopulation of T-cells. For further functional annotation of transcriptional differences, sets of DEGs were formed with the following statistical statements: adjusted p-value < 0.01 and absolute log2Fold change > 0.3. The common core of 71 genes was found by identifying the overlapping DEGs across the relevant T-cell clusters (the cell-cycle-associated population was excluded). The network of significant pathways (p-value <0.01) was obtained with Cytoscape (https://cytoscape.org/) and the ClueGO tool (https://apps.cytoscape.org/apps/cluego).

### 2.7 Granzyme B Functional Assays

#### 2.7.1 Granzyme B Toxicity Assay

Human recombinant granzyme B (R&D Systems Cat# 2906-SE-010) was resuspended in activation buffer (50 mM MES, 50 mM NaCl, pH = 5.5) with 10 μg/ml cathepsin D (R& D Systems, Cat# 2336-CY-010) for 4 h at 37°C. After activation, granzyme B was diluted at indicated concentrations in assay buffer (50 mM Trizma base, pH = 7.5) and added to MO3.13 cells (25,000 cells/well in 48-well plates) or OLs (200,000 cells/wells in 24-well plates) in fresh medium.

#### 2.7.2 Granzyme B Blocker Functional Assay

Th17-polarized cells were added to OLs (ratio 1:2.5, OL: T cells) or MO3.13 cells (100,000 cells per well, ratio 1:10, MO3.13 cell: T cells) in fresh media in 24-well plates. For granzyme B blockade in T cell–MO3.13 cell coculture, Th17-polarized cells were pre-incubated at 37°C for 1 h with a reversible granzyme B blocker Ac-IEPD-CHO (BioVision, Cat# 1119, Milpitas, CA, USA) or the serin protease inhibitor serpin A3N (R&D Systems, Cat# 4709-PI-010) at indicated concentrations in X-VIVO medium. Th17-polarized cells were washed before addition to MO3.13 cells (pretreatment condition). OLs/MO3.13 cells death was assessed by FACS staining after 16 h.

### 2.8 Luminex Assay

Immunoassays on supernatants from OLs and MO3.13 cells in coculture or not with polarized T cells were performed using a Luminex Discovery Assay kit (R&D Systems, Cat# LXSAHM). Briefly, samples were incubated with microparticle cocktail during 2 h at RT. After extensive washing, samples were incubated with biotin-antibody cocktail for 1 h at RT and washed. Biotinylated antibodies were revealed by incubation with streptavidin-PE during 30 min at RT. Results were read on a Luminex 200 (Bio-Rad, Hercules, CA, USA) analyzer.

### 2.9 Statistical Analysis

Graphs and statistics were generated using GraphPad Prism (GraphPad Prism, RRID:SCR_002798, http://www.graphpad.com/) software and R software (35) with ggplot2 (36) and dplyr (37) packages. For the statistical test, all data were tested with the Shapiro test to assess their distribution. Appropriate parametric or non-parametric tests were then applied and are specified in the figure legends. For graphical presentation, data were either presented as bar plot stating the mean +/- SEM, boxplots allowing visualization of data distribution (minimum, first quartile, median, third quartile, and maximum) with mean represented by a diamond, or violin plot representing distribution with an integrated boxplot for data distribution and black dot for mean, as specified in figure legends.

## 3 Results

### 3.1 Contact With Human OLs Modifies Behavior and Adhesion of Th17-Polarized Cells

**Figure 1:**
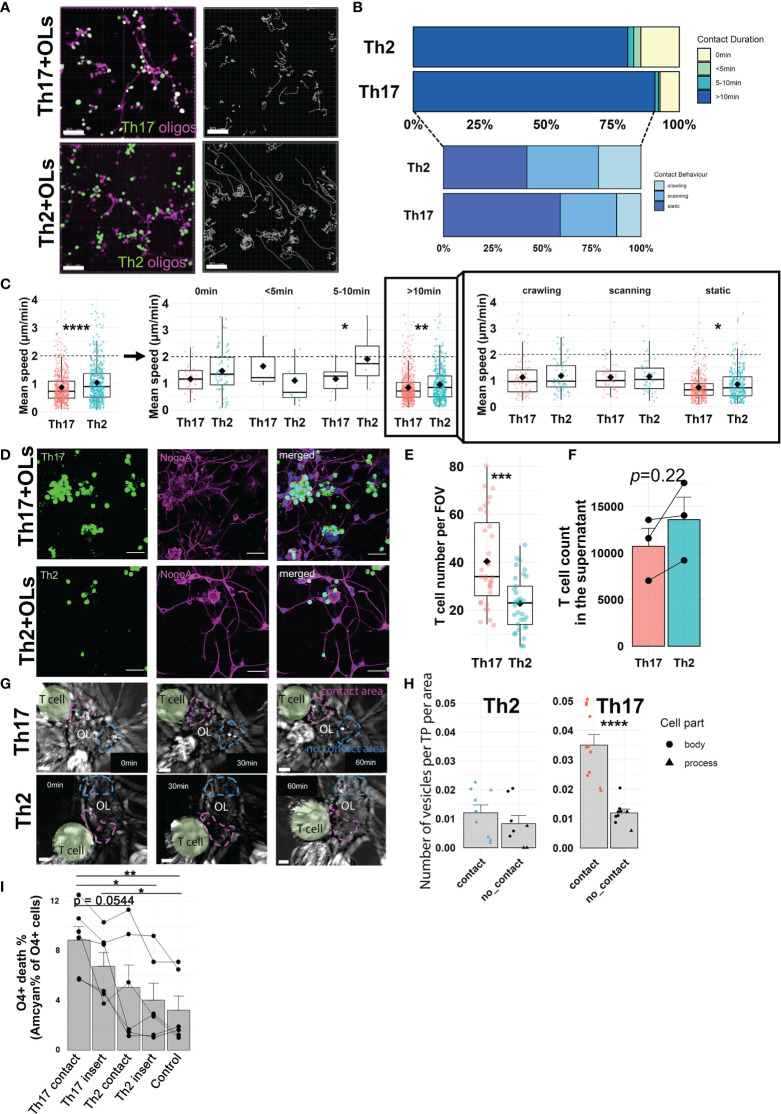
Active interactions of Th17-polarized cells with human primary OLs. **(A)** Representative images (left) and tracks (right, gray lines) of human Th2- or Th17-polarized cells (green) in coculture during 2 h with human OLs in primary culture (magenta), scale bar = 80 μm. **(B)** Proportion of T cells according to duration of contact with OLs (upper panel: no contact, < 5 min, 5–10 min or > 10 min) and contact behavior among T cells displaying a >10-min contact (lower panel: scanning, crawling, static). **(C)** Mean speed of polarized T cells in coculture with primary human OLs according to contact duration (left panel) and contact behavior (right panel) among T cells displaying >10-min contacts. Wilcoxon rank-sum test. **(A–C)** n = 4 different OL preps and 4 T cell donors, n = 526 cells (Th17-polarized) and 485 cells (Th2-polarized), 30-min observation periods. One dot represents one cell, diamond = mean. **(D)** Representative images and **(E)** quantification of adherent human Th2- or Th17-polarized cells (green) after 4 h in coculture with human OLs (magenta) in primary culture (scale bar = 40 μm). Wilcoxon test, n = 4 different OL preps and 4 T cell donors, diamond = mean. **(F)** Number of non-adherent T cells as measured by flow cytometry using AccuCount beads. Paired t-test, p = 0.22. n = 2 OL preps, n = 3 T cell donors. **(G)** Representative images and **(H)** quantification of vesicle-like structures per timepoint (TP) in areas of primary human OLs in contact (magenta) or not (blue) with human Th2- or Th17-polarized cells (vesicle number/TP/µm^2^). T cells are highlighted in green, areas of interest are delimited by dashed line, and vesicle-like structures are pointed by spots (contact in magenta, no contact in blue), n = 3 OL preps and 3 T cell donors, paired t-test. **(I)** Quantification of human primary OL death after 16 h of direct coculture with polarized T cells (contact) or separated by a permeable membrane (insert) at a 1:2.5 ratio. Control condition = no T cells. Each dot represents one donor, n = 6 different OL preps and 6 T cell donors, one way ANOVA with Tukey’s *post hoc* test. *p < 0.05, **p < 0.01, ***p < 0.001, ****p < 0.0001.

To visualize and compare the interactions of human OLs with either pro-inflammatory Th17 or anti-inflammatory Th2 human cell subsets, we developed a live-imaging assay (**Supplementary Figures 2A, B**). Human OLs and CD4^+^ T cells were stained with distinct non-toxic fluorescent dyes and could be easily distinguished during time-lapse acquisition (**Figure 1A**); behavior of individual CD4^+^ T cells was captured during 2 h. We confirmed that plate coating did not affect T-cell behavior and that the contact behavior of human Th17- and Th2-polarized cells was similar in the absence of target cells (**Supplementary Figures 3A, B**). Comparing the behavior of human Th17-polarized vs. Th2-polarized cells in coculture with primary human OLs, we report a higher percentage of Th17-polarized cells displaying a stable (>10 min) interaction with OL cells along with a significantly lower velocity of total Th17-polarized cells and of Th17-polarized cells engaging in interaction of ≥10 min with OLs (**Figures 1A–C**). This suggests a stronger modification of Th17-polarized cell behavior than of their Th2 counterpart upon contact with human OLs. The type of behavior displayed by T cells in stable contact with OLs was further characterized as either static, crawling, or scanning (see *Methods*, **Supplementary Figure 2C**). The proportion of Th17-polarized and Th2-polarized cells exhibiting each type of behavior was similar, but again the velocity of Th17-polarized cells showing prolonged stable contact was lower than that of Th2-polarized cells for the static (most common) behavior on OLs (**Figures 1B, C****)**. To confirm that human Th17-polarized cells show greater biologically relevant interactions with OLs, we assessed the adhesion of polarized T cells following imaging, after 4 h of coculture (**Figure 1D**). In line with our live imaging data, we observed that significantly higher numbers of Th17-polarized cells adhering to OLs remained compared to Th2-polarized cells (**Figure 1E**), in parallel with an increased number of Th2 cells found not interacting with OLs (**Figure 1F**). We obtained similar results using human oligodendrocytic cell line MO3.13 as target cells (**Supplementary Figures 4A–D**), therefore showing that MO3.13 cells are a valid alternative to the limited resource of primary human OLs. These data show that human Th17-polarized cells show a high propensity to interact with and adhere to human OLs.

### 3.2 Contact of Human Th17-Polarized Cells With Human OLs Causes Biological Alterations and OL Death

To further investigate the biological impact of Th17- vs. Th2-polarized cell interaction with OLs in real time, we performed brightfield live imaging on cocultures for 1 h. We analyzed the number of vesicle-like structures [200–600 nm, corresponding to the size of lytic granules (38)] detected in areas of a given OL in stable contact for at least 50 min with T cells (around a third of stable contacts) and compared to areas of the same OL not in contact with T cells (**Figure 1G**). Whereas the number of vesicle-like structures detected did not vary between OL areas in contact or not with Th2-polarized cells, the OL areas in contact with Th17-polarized cells showed a strikingly higher number of vesicle-like structures in OL processes and cell bodies than areas without (**Figure 1H**). In line with this, in MO3.13 cells, we observed transfer of dye from T cells to MO3.13 and similarly found a higher number of vesicle-like structures in areas of contact with Th17-polarized cells (**Supplementary Figures 4E–G**). In addition, to simultaneously compare both T cell subtypes, we added both Th17- and Th2-polarized cells (only one subset was loaded with CellTracker Green) to the same well of MO3.13 cells and performed bright-field/fluorescence live imaging (**Supplementary Figure 4H**). We found that the number of vesicle-like structures was significantly higher in the areas of a given MO3.13 cell in contact with Th17-polarized cells compared to areas in contact with Th2-polarized cells (**Supplementary Figure 4I**). To determine whether a direct prolonged OL-T cell contact was necessary for the observed cytotoxicity, we assessed OL survival after a 16h coculture when a permeable insert separated T cells and OLs. Th2-polarized cells in contact or separated by an insert induced less cell death of human OLs than Th17-polarized cells in contact with OLs (**Figure 1I**). Importantly, after only 16h, Th17-polarized cells when in direct contact compared to when separated by a porous membrane induced a significantly higher death rate of human primary OLs (contact 8.9 ± 2.7% vs. insert 6.8 ± 2.7%, paired t-test p = 0.0431) and of MO3.13 cells (**Supplementary Figure 4J**). Collectively, our results highlight that the interaction of human OLs with Th17-polarized cells results in biological alterations and triggers contact-dependent cell death of OLs.

### 3.3 Contact With Human OLs Leads to Significant Alterations in the Transcriptional Profile of Th17-Polarized Cells

**Figure 2:**
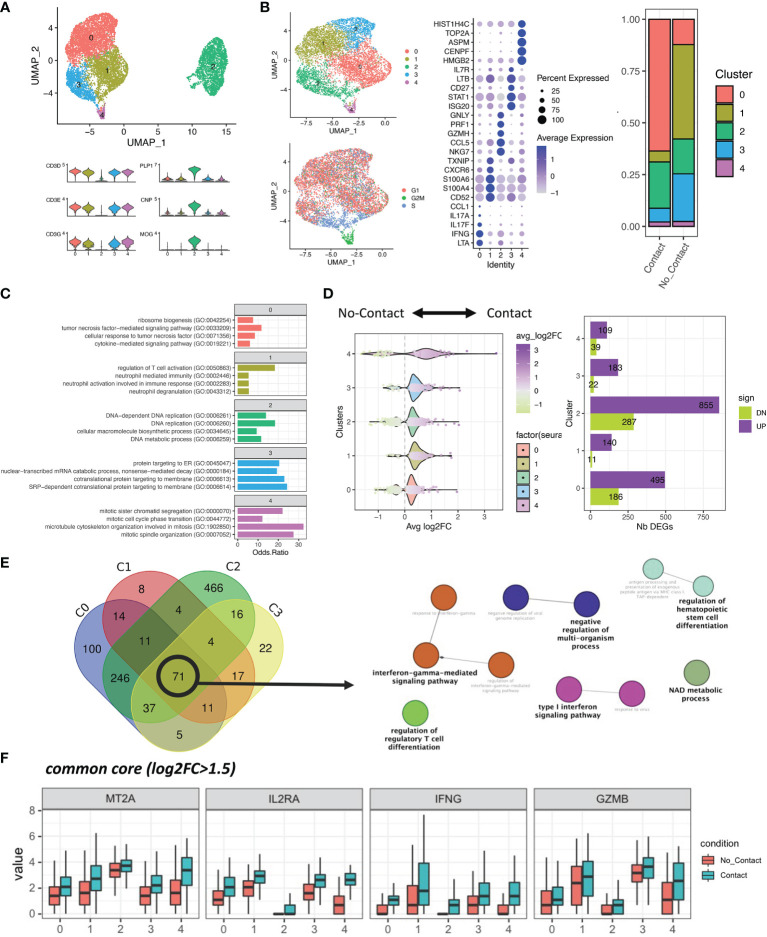
Single-cell RNA sequencing reveals a transcriptional shift of Th17-polarized cells in contact with OLs. **(A)** Upper panel: UMAP of 9,236 cells. Doublets, cells with high mitochondrial content and residual myeloid cells were removed. Lower panel: Expression of T cells and mature oligodendrocyte (OL) canonical markers. **(B)** Left: UMAP of the re-clustered T-cell populations (6,954 cells). Right: Proportions of each unbiased cluster across the two conditions. Middle: Top five markers across the five obtained clusters, as found with MAST. **(C)** Top four most significant biological processes (GO) associated with the significant markers of each cluster. **(D)** Left: Distribution of the log2Fold changes between conditions across the five T-cell clusters. Right: Total number of differentially expressed genes (DEGs) within each cluster, split by upregulated (purple) and downregulated (green) genes. **(E)** Venn diagram showing the overlap of DEGs between biologically relevant clusters (cell cycle-associated cluster removed) and network of biological processes (GO) significantly associated with the common core (71 genes). **(F)** Boxplot representation of the largest (adjusted p value < 0.0001 and log2FC > 1.5 in total Th17-polarized cells contact vs. no contact) upregulated genes upon direct cell contact with OLs across all T-cell clusters (all adjusted p value for contact versus no contact in every cluster < 0.05).

To identify potential contact-dependent death-inducing molecules expressed by Th17 cells, we performed single-cell RNA sequencing of human Th17-polarized cells either in direct contact with primary human OLs or separated by a permeable insert from OLs. After removal of the small number of myeloid (residual microglia) and dying cells, we distinguished OLs with high levels of *CNP*, *PLP*, and *MOG* transcript, and T cells with high mRNA levels of *CD3* (**Figure 2A**). Interestingly, in OLs we found that direct contact with Th17-polarized cells was associated with an upregulation of genes for chemokines CXCL10, CXCL11, and CXCL9 (**Supplementary Figure 5**). In addition, upon direct contact with Th17-polarized cells, OLs upregulated genes for antioxidant/anti-apoptotic molecule superoxide dismutase 2 (39) and anti-inflammatory molecule TNFAIP6 (40), but downregulated Nkx6-2, which is implicated in myelin formation (41). As expected in light of the observed toxicity of Th17-polarized cells in direct contact with OLs (**Figure 1I** and **Supplementary Figure 4J**), the direct contact with Th17-polarized cells was globally associated in OLs with an enrichment of genes associated with pathways linked to inflammation and regulation of cell death/apoptosis but a diminution of genes associated with pathways linked to neuroglial genesis and differentiation, including regulation of lipid metabolic and biosynthetic processes, cell projection, ensheathment of neurons, and oligodendrocyte differentiation (**Supplementary Figure 5** and **Supplementary Table 2**).

Within T cells, we identified 5 subsets for which the top five genes expressed are shown in **Figure 2B**. Cluster 0 is enriched in genes related to pro-inflammatory cytokines (*LTA*, *IFNG*, *IL17A*, and *IL17F*); cluster 1 is associated with cell metabolism (*CD52*, *TXNIP*) and cytoskeleton (*S100A4*, *S100A6*, and *CD52*) genes, suggesting a relative resting state of these cells; cluster 2 was characterized notably by expression of pro-lytic genes (*NKG7*, *GZMH*, *PRF1*, and *GNLY*); cluster 3 was enriched in genes involved in cytokine response signaling (*ISG20*, *STAT1*, *LTB*, *IL7R*) and T-cell survival (*CD27*); and cluster 4 was characterized by genes related to the cell cycle (*HMGB2*, *CENPF*, *ASPM*, *TOP2A*, *HIST1H4*). As cluster 4 represents a minor population mainly defined by cell-cycle genes, and its proportion among “contact” or “no contact” conditions is similar, we focused the subsequent analysis on clusters 0 to 3. We found that clusters of pro-inflammatory and pro-lytic T cells (clusters 0 and 2) represent a higher proportion of Th17-polarized cells after direct contact with OLs, whereas the proportion of resting T cells (cluster 1) and cytokine-responsive T cells (cluster 3) are higher when CD4^+^ T cells are separated by a porous membrane from OLs (**Figure 2B**). GO enrichment analysis of each cluster revealed that pro-inflammatory T cells (cluster 0) are enriched in TNF cytokine signaling among others, and pro-lytic T cells (cluster 2) in pathways implicated in DNA replication and metabolic processes, suggesting effector function and proliferation of these cells. On the other hand, we found that resting T cells (cluster 1) are characterized by pathways related to regulation of T-cell activation and neutrophil mechanisms, suggesting an anti-inflammatory potential. Finally, the pathways identified for cytokine-responsive T cells (cluster 3) involved mainly protein targeting and production (**Figure 2C**). This subanalysis highlighted that direct contact with OLs is associated with enrichment of cell subsets characterized by expression of genes related to inflammation and reduced proportion of subsets characterized by expression of genes associated with regulatory activity.

To find potential candidates responsible for OLs death by direct contact, we compared up- and downregulated genes in each cluster. We found that direct contact with OLs triggers an upregulation of expression of several genes in Th17-polarized cells for every cluster (**Figure 2D**). Moreover, when we looked at the common DEGs between T-cell clusters of interest (0, 1, 2 and 3, “common core”), we found 71 genes that are mostly involved in IFNγ signaling, regulation of cell differentiation, and energetic metabolic processes (**Figure 2E** and **Supplementary Table 1**). These genes coding for IL2 receptor α (*IL2RA*), IFNγ (*IFNG*), granzyme B (*GZMB*), and metallothionein 2A (*MT2A*) showed a more than 1.5 log2 fold change between total Th17-polarized cells in contact versus no contact conditions (**Supplementary Table 3**) and were all significantly elevated in Th17-polarized cells in direct contact with OLs in every cluster (**Figure 2F**). Altogether, these data revealed that RNA expression of pro-inflammatory cytokines and importantly of pro-apoptotic serin protease granzyme B (9) is induced in human Th17-polarized cells upon direct contact with primary human OLs.

### 3.4 Th17-Polarized Cells Express High Levels of Granzyme B After Contact With OLs

**Figure 3:**
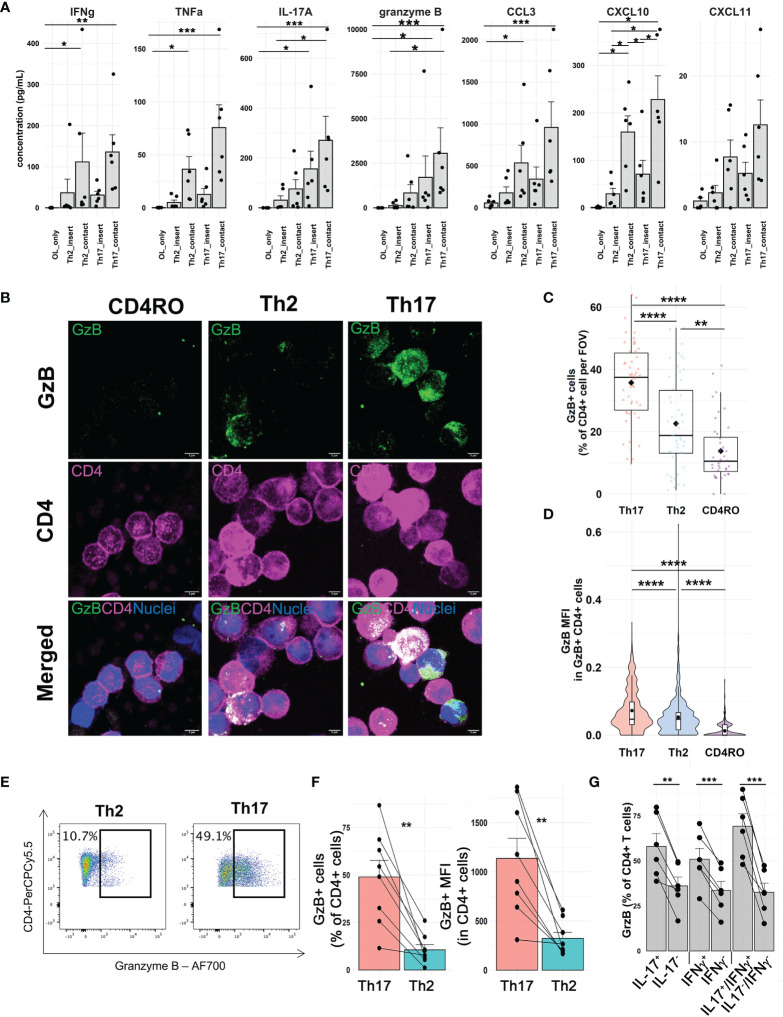
Secretion of pro-inflammatory cytokines, chemokines, and granzyme B upon direct contact between Th17-polarized cells and human OLs. **(A)** Concentration of analytes measured by a Luminex assay in supernatants from human polarized Th cells in direct contact or not with oligodendrocytes (OLs). Each dot represents one donor and OL prep, n = 6 different OL preps, and 6 T cell donors. Friedman test with Dunn’s method or one way ANOVA with Tukey’s *post hoc* test according to distribution for each analyte. **(B)** Representative confocal images (immunofluorescence, scale bar = 5 µm) of granzyme B expression (GzB, green) by CD4^+^ memory T cells *ex vivo* or after Th2 polarization versus Th17 polarization (magenta) and **(C)** quantification of the percentage of CD4^+^GrzB^+^-positive cells per field of view (FOV) and **(D)** the median fluorescence intensity (MFI) per cell in CD4^+^GzB^+^ cells. Wilcoxon rank test, n = 5 donors, diamond = mean. **(E)** Representative flow cytometry pseudocolor dot plots of GzB staining in polarized T cells and **(F)** quantification of percentage of CD4^+^ cells expressing GzB and median fluorescence intensity (MFI) in CD4^+^ T cells after 4 h PMA-ionomycin stimulation, paired t-test, n = 8 donors. **(G)** Percentage of Th17-polarized cells expressing granzyme B gating on IL-17^+^, IFNγ^+^, or double-positive cells compared to their negative counterpart, paired t-test, n = 6 donors. *p < 0.05; **p < 0.01; ***p < 0.001; ****p < 0.0001.

Among genes upregulated by Th17-polarized cells in contact with OLs identified by single-cell RNA sequencing, we validated by immunoassay pro-inflammatory cytokines IL-17A, IFNγ, and TNFα, cytotoxic molecule granzyme B, and chemokines CCL3 and CXCL10, the latter being also upregulated by OLs upon contact with Th17-polarized cells. We moreover measured chemokine CXCL11, whose gene is significantly upregulated only by OLs after contact. In line with our single-cell RNA sequencing data, we found higher levels of pro-inflammatory and cytotoxic molecules in the supernatant after direct contact of Th17-polarized cells with primary human OLs (**Figure 3A**), with a similar trend observed with MO3.13 cells (**Supplementary Figure 6**). Of note, OLs did not secrete a significant amount of any of these molecules in the absence of T cells. Importantly, supernatants from coculture of primary OLs/Th17-polarized cells in direct contact compared to those separated by a porous membrane were associated with significantly higher levels of granzyme B (median value of 1,690.0 pg/ml for contact versus 653.8 pg/ml for insert, Wilcoxon p = 0.0313), IFNγ (mean of 135.8 pg/ml versus 31.2 pg/ml, paired t test p = 0.0334), TNF (mean of 75.8 pg/ml versus 12.7 pg/ml, paired t test p = 0.0232), IL-17A (mean of 270.9 pg/ml versus 156.3 pg/ml, paired t test p = 0.0126), CCL3 (median of 620.2 pg/ml versus 239.6 pg/ml, Wilcoxon p = 0.0313), CXCL10 (mean of 228.1 pg/ml versus 70.9 pg/ml, paired t test p = 0.0044), and CXCL11 (mean of 12.6 pg/ml versus 5.2 pg/ml, paired t test p = 0.0179). We have previously shown that a 48h exposure to TNFα and IFNγ or IL-17 (data not shown) does not induce cell death of mature human OLs in primary culture (17, 18). Granzyme B has however been implicated in Th17-mediated neuronal cell death (9). As Th2-polarized cells did not induce significant OL cell death after 16h of coculture (**Figure 1I**) and secreted significantly lower levels of granzyme B than Th17-polarized cells (**Figures 3B–F**), we first confirmed that Th17-polarized cells are indeed the source of granzyme B in the supernatant of cocultures. Using an automated CellProfiler pipeline to quantify granzyme B-positive cells and its median intensity, we confirmed by immunofluorescence that Th17-polarized cells show the highest proportion of granzyme B positive CD4^+^ cells and that granzyme B^+^ Th17-polarized cells display the highest granzyme B median intensity signal (**Figures 3B–D**). Of note, Th2-polarized cells were enriched in granzyme B compared to non-activated *ex vivo* CD4^+^ memory cells (**Figures 3C, D****)**, but their expression remained much lower than that of Th17-polarized cell levels. These results were moreover confirmed by flow cytometry after PMA-ionomycin stimulation (**Figures 3E, F****)**. Furthermore, among Th17-polarized cells, the proportion of granzyme B-positive cells was higher among IL-17^+^ and IFNγ^+^ cells compared to their negative counterpart (**Figure 3G**).

**Figure 4:**
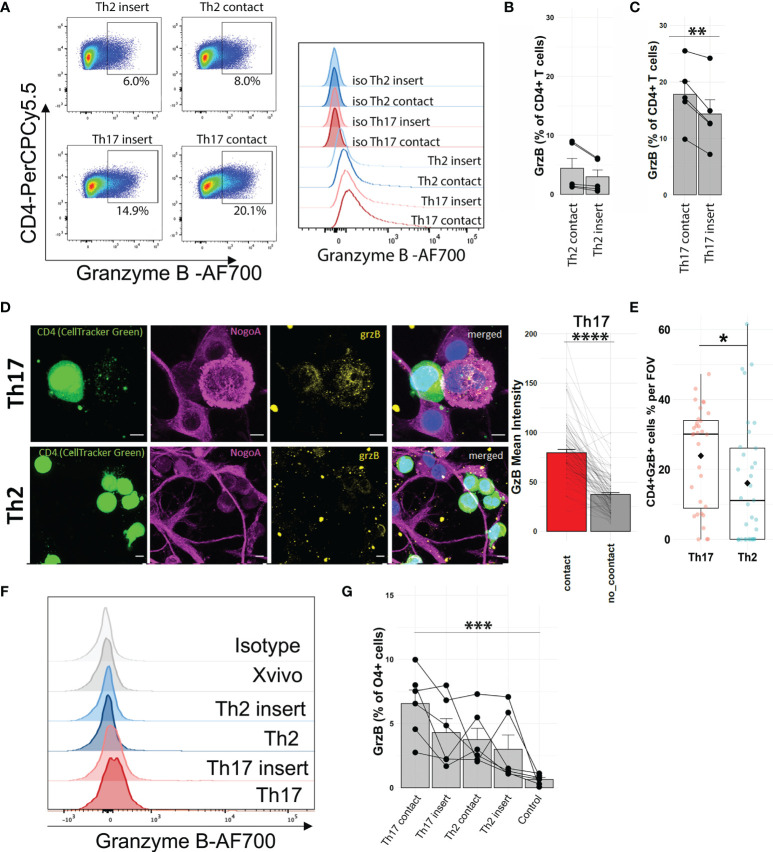
Direct contact with Th17-polarized cells is associated with detection of granzyme B in OLs. **(A)** Representative FACS pseudocolor dot plots and histogram overlay with isotype and **(B, C)** quantification of the proportion of CD4^+^ T cells expressing granzyme B in **(B)** Th2-polarized cells or **(C)** Th17-polarized cells after 16 h of coculture with human primary OL separated by a permeable membrane (insert) or in direct contact, no activation with PMA-ionomycin. Wilcoxon matched-pair test **(B)** or paired t-test **(C)**, n = 5 different OL preps and 5 T cell donors. **(D)** Representative immunofluorescent images of polarized T cell–OL coculture after 4 h and quantification of granzyme B (mean intensity) in areas of Th17-polarized cells in direct contact (red) or not (gray) with OL, n = 3 different OL preps and 4 T cell donors, total of 100 cells (25 cells/donor). **(E)** Quantification of granzyme B^+^ CD4^+^ cells (percentage), scale bar = 5 μm, n = 3 preps, n = 4 donors, Wilcoxon test, diamond = mean. **(F)** Representative FACS histogram and **(G)** quantification of the percentage of O4+ cells expressing granzyme B after 16h coculture with human primary OLs, Friedman test with Dunn’s method, n = 6 OL preps, and 6 T cell donors. **p < 0.01, ***p < 0.001, ****p < 0.0001.

Next, we validated that Th17-polarized cells upregulated their granzyme B levels after direct contact with OLs (no PMA-ionomycin stimulation) (**Figures 4A–C**). To determine if granzyme B can be found in human OLs after contact with Th17-polarized cells, we used immunofluorescence on fixed cocultures of Th17- and Th2-polarized cells and human OLs to localize granzyme B. We observed that granzyme B in Th17-polarized cells is indeed oriented toward areas of contact with OLs (**Figure 4D**). In addition, we found a higher number of granzyme B-positive T cells adhering to OLs following coculture with Th17-polarized compared to Th2-polarized cells (**Figure 4E**). Using flow cytometry, we similarly found that after coculture with T cells we could detect the presence of granzyme B in OLs, revealing that Th17-polarized cells induced higher levels of granzyme B in OLs (**Figures 4F, G****)**, and more so when in direct contact compared to when separated by a porous membrane (contact 6.6 ± 2.6% vs. insert 4.3 ± 2.7%, paired t test p = 0.0535). We further confirmed these results using the MO3.13 human OL cell line (**Supplementary Figure 7**). These data show that Th17-polarized cells in direct contact with OLs show elevated granzyme B expression that is polarized toward the target OLs.

### 3.5 Pharmacological Blockade of Granzyme B Reduces OL Death

**Figure 5:**
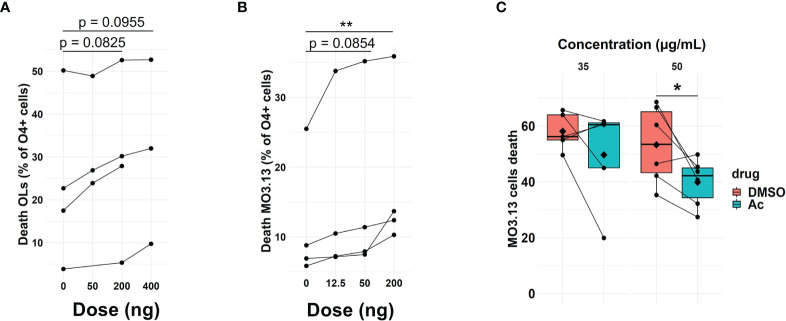
Th17-polarized secretion of granzyme B induces OL death. Percentage of **(A)** OLs (n = 4 different OL preps) and **(B)** MO3.13 cell death as measured by FACS (LIVE/DEAD Amcyan staining) after a 16h incubation with activated granzyme B. Each dot represents one independent experiment, n = 4 independent experiments, Friedmann test with Dunn’s method. **(C)** Percentage of MO3.13 cell death as measured by FACS (LIVE/DEAD Amcyan staining) after a 16h incubation with Th17-polarized cells pretreated with granzyme blocker Ac-IEPD-CHO (Ac) or DMSO control; each dot represents one donor, n = 5–6 T cell donors, diamond = mean, Wilcoxon test (35 μg/ml), and paired t-test (50 μg/ml). *p < 0.05; **p < 0.01.

**Figure 6:**
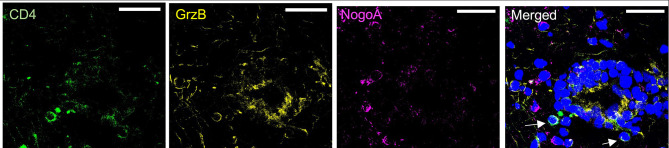
CD4 T cells express granzyme B in proximity to OLs in MS tissue. Representative confocal images of CD4^+^ cells (green) positive for granzyme B (yellow, upper panel) shown by arrows in proximity to mature OLs (NogoA, magenta) in MS tissue. Representative of two donors, scale bar = 30 μm.

Granzyme B can induce toxicity to target cells (42), and we found that Th17-polarized cells can result in the presence of this protease in OLs/MO3.13 cells. To investigate whether granzyme B can induce cell death of human OLs, activated recombinant human granzyme B was added and cell death measured after 16 h. We observed granzyme B toxicity on OLs (**Figure 5A**) and on MO3.13 cells (**Figure 5B**). To prove that granzyme B expressed by Th17-polarized cells contributes to OL death, we pretreated Th17-polarized cells with granzyme B blockers before their addition to MO3.13 cells. MO3.13 cells (without T cells) are resistant to exposure to DMSO (vehicle) or to synthetic granzyme B blocker Ac-IEPD-CHO at doses between 0 and 50 μg/ml (**Supplementary Figure 8A**). When incubating Th17-polarized cells with the granzyme B blocker or vehicle for 1 h before washing and addition to MO3.13 cells, we observed that the blocker does not affect T-cell survival (data not shown) but diminishes MO3.13 cell death rate compared to DMSO control condition (**Figure 5C**). Moreover, similar results were obtained using the natural granzyme B antagonist serpina3N (43, 44) (**Supplementary Figure 8B**). These results show that granzyme B production by Th17-polarized cells plays a role in Th17-mediated OL cell death. Finally, as we were able to demonstrate the presence of granzyme B-expressing CD4^+^ cells in active/chronic active MS brain lesions (**Figure 6**), our data suggest that CNS-infiltrating Th17-polarized cells display a high potential to interact with OLs and mediate OL cell death through secretion of granzyme B in MS.

## 4 Discussion

Mechanisms leading to OL damage in MS, the prototypical inflammatory demyelinating disease characterized by immune-mediated neuroglial injury, remain unclear. Previous studies have suggested OL death mechanisms that may contribute to MS pathology, such as ferroptosis (45), pyroptosis (46), or prolonged glucose deprivation/inflammation in the microenvironment of OLs in the CNS (18, 19). However, these mechanisms were not directly linked to interactions with infiltrating immune cells but rather to indirect consequences of cell invasion. Moreover, compared to rodent OLs and human OL precursor cells, mature human OLs are highly resistant to stressors such as pro-inflammatory cytokines (TNFα and IFNγ), excitotoxicity, and glucose deprivation (17–20). The presence of CD4^+^ T cells in NAWM and lesions in the CNS of MS patients has been extensively described (3, 47–50). In parallel, it has been shown that CD4^+^ T cells can induce OL death *in vitro* (17, 51), suggesting that direct CD4^+^ T cell-mediated OL death could participate to neuroglial injury in MS. We recently reported that one pathogenic subset of CD4^+^ T cells, Th17 cells, can form prolonged contact with OLs in EAE and secrete glutamate which causes rapid damage to OLs processes. However, we showed that Th17-polarized cells but not glutamate can subsequently induce OL death, suggesting that the first consequence of Th17-polarized cells on OLs would be process injury and myelination impairment through CD29-triggered release of glutamate, followed by OL death through yet unidentified mechanisms (17). Herein we showed that human Th17-polarized cells show a great capacity to target and adhere to human OLs, leading to important biological changes in Th17-polarized cells that will result in higher cytotoxicity. Furthermore, we found that granzyme B plays a role in OL cell death induced by Th17-polarized cells, which could be relevant to MS in light of the presence of CD4^+^ T cells expressing granzyme B that we observed MS CNS tissue.

Few to no human mature OLs express the protein MHC II at a detectable level (17), ruling out mismatch of MHC II between OLs and T cell donors as a mechanism underlying T cell-mediated OL injury in our experimental setup. Although the proportion of cells in contact and the velocity of Th17- and Th2-polarized cells is relatively similar in the absence of target cells, Th2-polarized cells showed significantly less interactions with oligodendroglial cells and conserved a higher motility on OLs and MO3.13 cells than Th17-polarized cells, even among cells able to form prolonged contact (**Figure 1** and **Supplementary Figure 4**). Longer contact and lower speed are usually associated with significant biological alterations in T cells, for example calcium influx during activation of Th17 cells by APCs (28, 29, 52, 53). Cytotoxic CD4^+^ T-cell morphological characteristics upon release of lytic granules into target cells involve either a round shape with adhesion to the surface of the target cell or the formation of lamellipodia with an elongated shape (54). These are respectively reminiscent of our static behavior in CD4^+^ T cells engaged in stable contacts with human OLs (adhering round shape) and of our scanning behavior (elongated adhering shape). The higher proportion of Th17-polarized cells forming stable contacts with OLs and therefore displaying static or scanning behavior compared to Th2 cells could suggest a higher cytotoxic potential through release of lytic granules from their part. This is supported by our bright-field live imaging showing the presence of vesicle-like structures in the size of lytic granules in OLs and MO3.13 cells in areas in contact with Th17- but not Th2-polarized cells. Interestingly, in our setup T cells appeared to interact more with cell bodies than processes of OLs, which might be due to technical factors (size/volume of surface, imaging technique) but could also reflect the rapid loss of processes with preservation of the cell body by OLs under stress conditions (17, 18) and/or differences in the level of expression of OL–T cell binding partners on the OL cell body versus processes.

We have recently reported that up to 30% of CD4^+^ T cells found in the CNS tissue of MS subjects, in either normal-appearing white matter or white matter lesion, are in contact with OLs (17). As CD4^+^ T cells can exert cytotoxicity toward OLs (51) but mature human OLs are rather resistant to soluble stressors (17–20), we used single-cell RNA sequencing to understand how contact with human OL shapes T-cell response and identify the mechanisms underlying OL death mediated by Th17 cell-polarized cells. Interestingly, we found that upon direct contact with Th17-polarized cells, OLs significantly upregulate their expression of chemokines CXCL10 and CXCL11, whose levels in the CSF are associated with clinical evolution in multiple sclerosis (55, 56). These chemokines are implicated in the recruitment of T cells to sites of inflammation (57–59) and could therefore promote interactions between Th17-polarized cells and OLs. In particular, CXCL10 directly promotes expression of IFNγ by activated CD4 T cells (60). In parallel, our data demonstrate that human Th17-polarized cells are strongly affected by their direct contact with human OLs, showing an important shift toward a pro-inflammatory phenotype. We observed the upregulation of genes linked to metabolism, such as glycolysis-related nicotinamide adenine dinucleotide (NAD) (61–65) and of TNFα and IFNγ signaling pathways, which we were able to confirm at the protein level by immunoassays. Of note, contact with OLs is associated with an upregulation of IL-2RA mRNA in Th17-polarized cells, which is a gene associated with MS susceptibility (66) and with pathogenic Th17 cells in EAE (67). IFNγ and IL-2 signaling is associated with expression of *TBX21*, a transcription factor that was upregulated in Th17-polarized cells upon contact with OLs (**Supplementary Table 2**) and induces expression of granzyme B (68). Importantly, we report that direct contact of human Th17-polarized cells with human OLs leads to an upregulation of secretion of granzyme B in the supernatant and of levels of granzyme B mRNA and protein in Th17-polarized cells. While expression of *GZMB* remains low in OLs even after direct contact with Th17-polarized cells (**Supplementary Figure 9A**), we cannot exclude that OLs exposed to activated T cells contribute to the increased levels of granzyme B in the supernatant and within OLs. Nevertheless, as we showed that granzyme B can induce OL cell death and that interfering with granzyme B activity of Th17-polarized cells protect human oligodendrocytic MO3.13 cells, we have identified granzyme B-mediated cytotoxicity as a mechanism implicated in immune-mediated human OL cell death. Our data therefore show that direct contact of CD4^+^ T cells with OLs promotes a Th17/Th1 pro-inflammatory phenotype with secretion of cytokines and chemokines that could play a role in sustaining CNS-targeted autoimmune processes by destabilizing and activating the BBB, promoting the recruitment and activation of immune cells, and exerting direct cytotoxicity toward OLs (69–76).

Granzyme B is a serin protease expressed by many immune cell types. Described initially in CD8 T cells as an essential mediator for killing target cells (77), granzyme B is also highly expressed in NK cells and other T cells, notably activated CD4^+^ T cells (9, 78, 79). Granzyme B is well known to induce cell apoptosis, chromatin condensation, DNA disintegration (80), and pro-apoptotic caspase-7 launch (24) or cell metabolism/function alterations (23). In line with previous publications, we report that among Th17-polarized cells, IL-17^+^ T cells preferentially express granzyme B (9) but that a high proportion of IFNγ^+^ CD4 T cells also express granzyme B (81). Cytotoxic IFNγ^+^CD28^negative^ CD4 T cells are enriched in the peripheral blood of MS patients; these cells can contribute to immune-mediated OL injury (82–84). Interestingly, we observed that the expression (raw counts) of *CD28* mRNA was generally low, especially in cells with high expression of *GZMB* in Th17-polarized cells in coculture with OLs by single-cell RNA sequencing (**Supplementary Figure 9B**). Granzyme B levels are elevated in the CSF of MS patients (42) and CD4^+^ T cells from MS patients secrete more granzyme B than healthy controls in the relapsing–remitting (85) and progressive forms (86). In addition, pro-inflammatory CD4^+^ T cells secreting granzyme B can disrupt the blood–brain barrier (87) and cause glial fibrillary acid protein fragmentation in human primary astrocytes *in vitro* (22). Higher levels of natural granzyme B antagonist serpina3N were found in the CSF of MS patients, particularly in the progressive forms, and were correlated with neurofilament levels, suggesting that CNS cells attempt but do not succeed to prevent granzyme B toxicity in MS (88). In EAE, serpina3N reduced disease severity and delayed onset of symptoms (89). As serpina3N injections in EAE did not stop CD4^+^ and CD8^+^ T-cell infiltration, granzyme B inhibition is considered to play a role directly in mediating injury to neuroglial cells in the CNS (89), in line with a protective effect of the granzyme B inhibitor on human fetal neurons cocultured with activated T cells (89, 90). Although subject to the limitations inherent to *in vitro* studies, as well-described discrepancies between the vulnerability of rodent OLs compared to human OLs exist (20, 91), our human study is relevant for MS. Importantly, we found CD4^+^ T cells expressing granzyme B in *postmortem* CNS tissue of MS patients (**Figure 6**), in line with the previously described presence of CNS-infiltrating CD4^+^ T cells expressing granzyme B in the CNS tissue from one RRMS (92) and one SPMS subject (86). Taken together, our data support the hypothesis that granzyme B is a mechanism implicated in the complex deleterious cross talk between OLs and Th17-polarized cells in MS. As neuroprotective therapies are still lacking, our work provides new insights into potential therapeutic targets to limit neuroglial injury initiated by Th17-polarized cells.

## Data Availability Statement

The RNAseq datasets generated for this study were deposited in the Gene Expression Omnibus, accession GSE196953. The raw data supporting the conclusions of this article will be made available by the authors upon request.

## Ethics Statement

The studies involving human participants were reviewed and approved by the Centre Hospitalier de l’Université de Montréal Institutional Research Ethic Board. Written informed consent was obtained from every donor prior to sample collection (CRCHUM ethic committee approval numbers SL05.022, SL05.023 and BH07.001). Peripheral venous blood from healthy donors was obtained and used following ethical guidelines from the CRCHUM (Nagano 19.088).

## Author Contributions

CL and HJ conceived and designed the study. HJ performed the main experimental work with help from VM, OO, AD, FL, and ACB. HJ, CL, HD, OT, and AH analyzed the data. CL and HJ performed the immunofluorescence experiments. HJ performed the live-imaging experiments. HJ, HD, RB, and AD performed human T cell and OLs/MO3.13 cell coculture experiments. AD and WK performed Luminex assays. QC, FP, NF, NA, and JA provided oligodendrocyte cultures. JS and Q-LC prepared samples for single-cell RNA sequencing data. OT performed analysis of single-cell RNA sequencing experiments. CL and HJ evaluated all data and wrote and edited the manuscript. HD, JA, NA, and JS provided critical scientific input. All authors contributed to the article and approved the submitted version.

## Funding

This work was financially supported by the Canadian Institutes of Health Research (CIHR 162430 to CL) and the Multiple Sclerosis Society of Canada (Grant EGID 3322 to CL and studentship to HJ, RB and VHM). CL holds a Junior Scholar Award from the Fonds de Recherche du Québec-Santé (FRQ-S). OO received a Canadian Francophonie scholarship.

## Conflict of Interest

The authors declare that the research was conducted in the absence of any commercial or financial relationships that could be construed as a potential conflict of interest.

## Publisher’s Note

All claims expressed in this article are solely those of the authors and do not necessarily represent those of their affiliated organizations, or those of the publisher, the editors and the reviewers. Any product that may be evaluated in this article, or claim that may be made by its manufacturer, is not guaranteed or endorsed by the publisher.

## References

[1] Dendrou CA Fugger L Friese MA. Immunopathology of Multiple Sclerosis. Nat Rev Immunol (2015) 15(9):545–58. doi: 10.1038/nri3871 26250739

[2] Jakel S Agirre E Falcao AM van Bruggen D Lee KW Knuesel I. Altered Human Oligodendrocyte Heterogeneity in Multiple Sclerosis. Nature (2019) 566(7745):543–7. doi: 10.1038/s41586-019-0903-2 PMC6544546 30747918

[3] van Nierop GP van Luijn MM Michels SS Melief MJ Janssen M Langerak AW. Phenotypic and Functional Characterization of T Cells in White Matter Lesions of Multiple Sclerosis Patients. Acta Neuropathol (2017) 134(3):383–401. doi: 10.1007/s00401-017-1744-4 28624961 PMC5563341

[4] Fransen NL Hsiao CC van der Poel M Engelenburg HJ Verdaasdonk K Vincenten MCJ. Tissue-Resident Memory T Cells Invade the Brain Parenchyma in Multiple Sclerosis White Matter Lesions. Brain (2020) 143(6):1714–30. doi: 10.1093/brain/awaa117 32400866

[5] Saikali P Antel JP Newcombe J Chen Z Freedman M Blain M. NKG2D-Mediated Cytotoxicity Toward Oligodendrocytes Suggests a Mechanism for Tissue Injury in Multiple Sclerosis. J Neurosci (2007) 27(5):1220–8. doi: 10.1523/JNEUROSCI.4402-06.2007 PMC6673175 17267578

[6] Göbel K Melzer N Herrmann AM Schuhmann MK Bittner S Ip CW. Collateral Neuronal Apoptosis in CNS Gray Matter During an Oligodendrocyte-Directed CD8(+) T Cell Attack. Glia (2010) 58(4):469–80. doi: 10.1002/glia.20938 19780193

[7] Ruijs TC Freedman MS Grenier YG Olivier A Antel JP. Human Oligodendrocytes Are Susceptible to Cytolysis by Major Histocompatibility Complex Class I-Restricted Lymphocytes. J Neuroimmunol (1990) 27(2-3):89–97. doi: 10.1016/0165-5728(90)90058-U 1970580 PMC7119690

[8] Denic A Wootla B Rodriguez M. CD8(+) T Cells in Multiple Sclerosis. Expert Opin Ther Targets (2013) 17(9):1053–66. doi: 10.1517/14728222.2013.815726 PMC3928018 23829711

[9] Kebir H Kreymborg K Ifergan I Dodelet-Devillers A Cayrol R Bernard M. Human TH17 Lymphocytes Promote Blood-Brain Barrier Disruption and Central Nervous System Inflammation. Nat Med (2007) 13(10):1173–5. doi: 10.1038/nm1651 PMC5114125 17828272

[10] Kebir H Ifergan I Alvarez JI Bernard M Poirier J Arbour N. Preferential Recruitment of Interferon-Gamma-Expressing TH17 Cells in Multiple Sclerosis. Ann Neurol (2009) 66(3):390–402. doi: 10.1002/ana.21748 19810097

[11] Luchtman DW Ellwardt E Larochelle C Zipp F. IL-17 and Related Cytokines Involved in the Pathology and Immunotherapy of Multiple Sclerosis: Current and Future Developments. Cytokine Growth Factor Rev (2014) 25(4):403–13. doi: 10.1016/j.cytogfr.2014.07.013 25153998

[12] Larochelle C Cayrol R Kebir H Alvarez JI Lecuyer MA Ifergan I. Melanoma Cell Adhesion Molecule Identifies Encephalitogenic T Lymphocytes and Promotes Their Recruitment to the Central Nervous System. Brain (2012) 135(Pt 10):2906–24. doi: 10.1093/brain/aws212 22975388

[13] Codarri L Gyulveszi G Tosevski V Hesske L Fontana A Magnenat L. RORgammat Drives Production of the Cytokine GM-CSF in Helper T Cells, Which Is Essential for the Effector Phase of Autoimmune Neuroinflammation. Nat Immunol (2011) 12(6):560–7. doi: 10.1038/ni.2027 21516112

[14] Fujii C Kondo T Ochi H Okada Y Hashi Y Adachi T. Altered T Cell Phenotypes Associated With Clinical Relapse of Multiple Sclerosis Patients Receiving Fingolimod Therapy. Sci Rep (2016) 6:35314. doi: 10.1038/srep35314 27752051 PMC5082790

[15] Matusevicius D Kivisäkk P He B Kostulas N Ozenci V Fredrikson S. Interleukin-17 mRNA Expression in Blood and CSF Mononuclear Cells Is Augmented in Multiple Sclerosis. Mult Scler (1999) 5(2):101–4. doi: 10.1177/135245859900500206 10335518

[16] Tzartos JS Friese MA Craner MJ Palace J Newcombe J Esiri MM. Interleukin-17 Production in Central Nervous System-Infiltrating T Cells and Glial Cells Is Associated With Active Disease in Multiple Sclerosis. Am J Pathol (2008) 172(1):146–55. doi: 10.2353/ajpath.2008.070690 PMC2189615 18156204

[17] Larochelle C Wasser B Jamann H Loffel JT Cui QL Tastet O. Pro-Inflammatory T Helper 17 Directly Harms Oligodendrocytes in Neuroinflammation. Proc Natl Acad Sci USA (2021) 118(34):e2025813118. doi: 10.1073/pnas.2025813118 PMC8403833 34417310

[18] Cui QL Khan D Rone M TSR V Johnson RM Lin YH. Sublethal Oligodendrocyte Injury: A Reversible Condition in Multiple Sclerosis? Ann Neurol (2017) 81(6):811–24. doi: 10.1002/ana.24944 28470695

[19] Rao VTS Khan D Cui QL Fuh SC Hossain S Almazan G. Distinct Age and Differentiation-State Dependent Metabolic Profiles of Oligodendrocytes Under Optimal and Stress Conditions. PloS One (2017) 12(8):e0182372. doi: 10.1371/journal.pone.0182372 28792512 PMC5549710

[20] Wosik K Ruffini F Almazan G Olivier A Nalbantoglu J Antel JP. Resistance of Human Adult Oligodendrocytes to AMPA/kainate Receptor-Mediated Glutamate Injury. Brain (2004) 127(Pt 12):2636–48. doi: 10.1093/brain/awh302 15509624

[21] Buntinx M Vanderlocht J Hellings N Vandenabeele F Lambrichts I Raus J. Characterization of Three Human Oligodendroglial Cell Lines as a Model to Study Oligodendrocyte Injury: Morphology and Oligodendrocyte-Specific Gene Expression. J Neurocytol (2003) 32(1):25–38. doi: 10.1023/A:1027324230923 14618099

[22] Stopnicki B Blain M Cui QL Kennedy TE Antel JP Healy LM. Helper CD4 T Cells Expressing Granzyme B Cause Glial Fibrillary Acidic Protein Fragmentation in Astrocytes in an MHCII-Independent Manner. Glia (2019) 67(4):582–93. doi: 10.1002/glia.23503 30444064

[23] Heibein JA Barry M Motyka B Bleackley RC. Granzyme B-Induced Loss of Mitochondrial Inner Membrane Potential (Delta Psi M) and Cytochrome C Release Are Caspase Independent. J Immunol (1999) 163(9):4683–93. 10528165

[24] Yang X Stennicke HR Wang B Green DR Jänicke RU Srinivasan A. Granzyme B Mimics Apical Caspases. Description of a Unified Pathway for Trans-Activation of Executioner Caspase-3 and -7. J Biol Chem (1998) 273(51):34278–83. doi: 10.1074/jbc.273.51.34278 9852092

[25] Lemaitre F Carmena Moratalla A Farzam-Kia N Carpentier Solorio Y Tastet O Cleret-Buhot A. Capturing T Lymphocytes' Dynamic Interactions With Human Neural Cells Using Time-Lapse Microscopy. Front Immunol (2021) 12:668483. doi: 10.3389/fimmu.2021.668483 33968073 PMC8100528

[26] Negulescu PA Krasieva TB Khan A Kerschbaum HH Cahalan MD. Polarity of T Cell Shape, Motility, and Sensitivity to Antigen. Immunity (1996) 4(5):421–30. doi: 10.1016/S1074-7613(00)80409-4 8630728

[27] Lin W Suo Y Deng Y Fan Z Zheng Y Wei X. Morphological Change of CD4(+) T Cell During Contact With DC Modulates T-Cell Activation by Accumulation of F-Actin in the Immunology Synapse. BMC Immunol (2015) 16:49. doi: 10.1186/s12865-015-0108-x 26306899 PMC4549951

[28] Moreau HD Lemaitre F Garrod KR Garcia Z Lennon-Dumenil AM Bousso P. Signal Strength Regulates Antigen-Mediated T-Cell Deceleration by Distinct Mechanisms to Promote Local Exploration or Arrest. Proc Natl Acad Sci USA (2015) 112(39):12151–6. doi: 10.1073/pnas.1506654112 PMC4593123 26371316

[29] Friedman RS Beemiller P Sorensen CM Jacobelli J Krummel MF. Real-Time Analysis of T Cell Receptors in Naive Cells *In Vitro* and *In Vivo* Reveals Flexibility in Synapse and Signaling Dynamics. J Exp Med (2010) 207(12):2733–49. doi: 10.1084/jem.20091201 PMC2989766 21041455

[30] Larochelle C Uphaus T Broux B Gowing E Paterka M Michel L. EGFL7 Reduces CNS Inflammation in Mouse. Nat Commun (2018) 9(1):819. doi: 10.1038/s41467-018-03186-z 29483510 PMC5827652

[31] Larochelle C Lecuyer MA Alvarez JI Charabati M Saint-Laurent O Ghannam S. Melanoma Cell Adhesion Molecule-Positive CD8 T Lymphocytes Mediate Central Nervous System Inflammation. Ann Neurol (2015) 78(1):39–53. doi: 10.1002/ana.24415 25869475

[32] Schindelin J Arganda-Carreras I Frise E Kaynig V Longair M Pietzsch T. Fiji: An Open-Source Platform for Biological-Image Analysis. Nat Methods (2012) 9(7):676–82. doi: 10.1038/nmeth.2019 PMC3855844 22743772

[33] McQuin C Goodman A Chernyshev V Kamentsky L Cimini BA Karhohs KW. CellProfiler 3.0: Next-Generation Image Processing for Biology. PloS Biol (2018) 16(7):e2005970. doi: 10.1371/journal.pbio.2005970 29969450 PMC6029841

[34] Hao Y Hao S Andersen-Nissen E Mauck WM Zheng S Butler A. Integrated Analysis of Multimodal Single-Cell Data. Cell (2021) 184(13):3573–87.e29. doi: 10.1016/j.cell.2021.04.048 34062119 PMC8238499

[35] Team RC. R: A Language and Environment for Statistical Computing. Vienna: Foundation for Statistical Computing (2013).

[36] Wickham H. Ggplot2: Elegant Graphics for Data Analysis. Springer (2015).

[37] Wickham H Francois R Henry L Müller K. Dplyr: A Grammar of Data Manipulation. R Package Version 04, vol. 3. (2015). p156. Available at: http://CRAN.R-project.org/package=dplyr.

[38] Martina JA Wu XS Catalfamo M Sakamoto T Yi C Hammer JA 3rd. Imaging of Lytic Granule Exocytosis in CD8+ Cytotoxic T Lymphocytes Reveals a Modified Form of Full Fusion. Cell Immunol (2011) 271(2):267–79. doi: 10.1016/j.cellimm.2011.07.004 PMC3407469 21843881

[39] Butts BD Houde C Mehmet H. Maturation-Dependent Sensitivity of Oligodendrocyte Lineage Cells to Apoptosis: Implications for Normal Development and Disease. Cell Death Differ (2008) 15(7):1178–86. doi: 10.1038/cdd.2008.70 18483490

[40] Watanabe R Sato Y Ozawa N Takahashi Y Koba S Watanabe T. Emerging Roles of Tumor Necrosis Factor-Stimulated Gene-6 in the Pathophysiology and Treatment of Atherosclerosis. Int J Mol Sci (2018) 19(2):465. doi: 10.3390/ijms19020465 PMC5855687 29401724

[41] Dorboz I Aiello C Simons C Stone RT Niceta M Elmaleh M. Biallelic Mutations in the Homeodomain of NKX6-2 Underlie a Severe Hypomyelinating Leukodystrophy. Brain (2017) 140(10):2550–6. doi: 10.1093/brain/awx207 28969374

[42] Wang T Lee MH Johnson T Allie R Hu L Calabresi PA. Activated T-Cells Inhibit Neurogenesis by Releasing Granzyme B: Rescue by Kv1.3 Blockers. J Neurosci (2010) 30(14):5020–7. doi: 10.1523/JNEUROSCI.0311-10.2010 PMC2878660 20371822

[43] Sun J Bird CH Sutton V McDonald L Coughlin PB De Jong TA. A Cytosolic Granzyme B Inhibitor Related to the Viral Apoptotic Regulator Cytokine Response Modifier A Is Present in Cytotoxic Lymphocytes. J Biol Chem (1996) 271(44):27802–9. doi: 10.1074/jbc.271.44.27802 8910377

[44] Sipione S Simmen KC Lord SJ Motyka B Ewen C Shostak I. Identification of a Novel Human Granzyme B Inhibitor Secreted by Cultured Sertoli Cells. J Immunol (2006) 177(8):5051–8. doi: 10.4049/jimmunol.177.8.5051 17015688

[45] Jhelum P Santos-Nogueira E Teo W Haumont A Lenoël I Stys PK. Ferroptosis Mediates Cuprizone-Induced Loss of Oligodendrocytes and Demyelination. J Neurosci (2020) 40(48):9327–41. doi: 10.1523/JNEUROSCI.1749-20.2020 PMC7687057 33106352

[46] McKenzie BA Mamik MK Saito LB Boghozian R Monaco MC Major EO. Caspase-1 Inhibition Prevents Glial Inflammasome Activation and Pyroptosis in Models of Multiple Sclerosis. Proc Natl Acad Sci USA (2018) 115(26):E6065–e74. doi: 10.1073/pnas.1722041115 PMC6042136 29895691

[47] Fransen NL Crusius JBA Smolders J Mizee MR van Eden CG Luchetti S. Post-Mortem Multiple Sclerosis Lesion Pathology Is Influenced by Single Nucleotide Polymorphisms. Brain Pathol (2020) 30(1):106–19. doi: 10.1111/bpa.12760 PMC6916567 31228212

[48] Larochelle C Uphaus T Prat A Zipp F. Secondary Progression in Multiple Sclerosis: Neuronal Exhaustion or Distinct Pathology? Trends Neurosci (2016) 39(5):325–39. doi: 10.1016/j.tins.2016.02.001 26987259

[49] Lassmann H. Mechanisms of White Matter Damage in Multiple Sclerosis. Glia (2014) 62(11):1816–30. doi: 10.1002/glia.22597 24470325

[50] Reich DS Lucchinetti CF Calabresi PA. Multiple Sclerosis. N Engl J Med (2018) 378(2):169–80. doi: 10.1056/NEJMra1401483 PMC6942519 29320652

[51] Zaguia F Saikali P Ludwin S Newcombe J Beauseigle D McCrea E. Cytotoxic NKG2C+ CD4 T Cells Target Oligodendrocytes in Multiple Sclerosis. J Immunol (2013) 190(6):2510–8. doi: 10.4049/jimmunol.1202725 23396942

[52] Weber KS Miller MJ Allen PM. Th17 Cells Exhibit a Distinct Calcium Profile From Th1 and Th2 Cells and Have Th1-Like Motility and NF-AT Nuclear Localization. J Immunol (2008) 180(3):1442–50. doi: 10.4049/jimmunol.180.3.1442 18209039

[53] Moseman EA Blanchard AC Nayak D McGavern DB. T Cell Engagement of Cross-Presenting Microglia Protects the Brain From a Nasal Virus Infection. Sci Immunol (2020) 5(48):eabb1817. doi: 10.1126/sciimmunol.abb1817 PMC7416530 32503876

[54] Frevert U Moreno A Calvo-Calle JM Klotz C Nardin E. Imaging Effector Functions of Human Cytotoxic CD4+ T Cells Specific for Plasmodium Falciparum Circumsporozoite Protein. Int J Parasitol (2009) 39(1):119–32. doi: 10.1016/j.ijpara.2008.06.014 PMC3021960 18723023

[55] Nohejlova H Kayserova J Capek V Toman T Krsek P Liba Z. Paediatric Onset of Multiple Sclerosis: Analysis of Chemokine and Cytokine Levels in the Context of the Early Clinical Course. Mult Scler Relat Disord (2020) 46:102467. doi: 10.1016/j.msard.2020.102467 32889374

[56] Puthenparampil M Stropparo E Zywicki S Bovis F Cazzola C Federle L. Wide Cytokine Analysis in Cerebrospinal Fluid at Diagnosis Identified CCL-3 as a Possible Prognostic Factor for Multiple Sclerosis. Front Immunol (2020) 11:174. doi: 10.3389/fimmu.2020.00174 32194540 PMC7066207

[57] Lee JH Kim B Jin WJ Kim HH Ha H Lee ZH. Pathogenic Roles of CXCL10 Signaling Through CXCR3 and TLR4 in Macrophages and T Cells: Relevance for Arthritis. Arthritis Res Ther (2017) 19(1):163. doi: 10.1186/s13075-017-1353-6 28724396 PMC5518115

[58] Tokunaga R Zhang W Naseem M Puccini A Berger MD Soni S. CXCL9, CXCL10, CXCL11/CXCR3 Axis for Immune Activation - A Target for Novel Cancer Therapy. Cancer Treat Rev (2018) 63:40–7. doi: 10.1016/j.ctrv.2017.11.007 PMC5801162 29207310

[59] Karin N. CXCR3 Ligands in Cancer and Autoimmunity, Chemoattraction of Effector T Cells, and Beyond. Front Immunol (2020) 11:976. doi: 10.3389/fimmu.2020.00976 32547545 PMC7274023

[60] Vasquez RE Xin L Soong L. Effects of CXCL10 on Dendritic Cell and CD4+ T-Cell Functions During Leishmania Amazonensis Infection. Infect Immun (2008) 76(1):161–9. doi: 10.1128/IAI.00825-07 PMC2223631 17998308

[61] Cluxton D Petrasca A Moran B Fletcher JM. Differential Regulation of Human Treg and Th17 Cells by Fatty Acid Synthesis and Glycolysis. Front Immunol (2019) 10:115. doi: 10.3389/fimmu.2019.00115 30778354 PMC6369198

[62] Berod L Friedrich C Nandan A Freitag J Hagemann S Harmrolfs K. *De Novo* Fatty Acid Synthesis Controls the Fate Between Regulatory T and T Helper 17 Cells. Nat Med (2014) 20(11):1327–33. doi: 10.1038/nm.3704 25282359

[63] Wagner A Wang C Fessler J DeTomaso D Avila-Pacheco J Kaminski J. Metabolic Modeling of Single Th17 Cells Reveals Regulators of Autoimmunity. Cell (2021) 184(16):4168–85.e21. doi: 10.1016/j.cell.2021.05.045 34216539 PMC8621950

[64] Chatterjee S Daenthanasanmak A Chakraborty P Wyatt MW Dhar P Selvam SP. CD38-NAD(+)Axis Regulates Immunotherapeutic Anti-Tumor T Cell Response. Cell Metab (2018) 27(1):85–100.e8. doi: 10.1016/j.cmet.2017.10.006 29129787 PMC5837048

[65] Elkhal A Rodriguez Cetina Biefer H Heinbokel T Uehara H Quante M Seyda M. NAD(+) Regulates Treg Cell Fate and Promotes Allograft Survival *via* a Systemic IL-10 Production That Is CD4(+) CD25(+) Foxp3(+) T Cells Independent. Sci Rep (2016) 6:22325. doi: 10.1038/srep22325 26928119 PMC4772111

[66] Wang XX Chen T. Meta-Analysis of the Association of IL2RA Polymorphisms Rs2104286 and Rs12722489 With Multiple Sclerosis Risk. Immunol Invest (2018) 47(5):431–42. doi: 10.1080/08820139.2018.1425699 29648897

[67] Hoppmann N Graetz C Paterka M Poisa-Beiro L Larochelle C Hasan M. New Candidates for CD4 T Cell Pathogenicity in Experimental Neuroinflammation and Multiple Sclerosis. Brain J Neurol (2015) 138(Pt 4):902–17. doi: 10.1093/brain/awu408 25665584

[68] Hua L Yao S Pham D Jiang L Wright J Sawant D. Cytokine-Dependent Induction of CD4+ T Cells With Cytotoxic Potential During Influenza Virus Infection. J Virol (2013) 87(21):11884–93. doi: 10.1128/JVI.01461-13 PMC3807312 23986597

[69] Larochelle C Alvarez JI Prat A. How do Immune Cells Overcome the Blood-Brain Barrier in Multiple Sclerosis? FEBS Lett (2011) 585(23):3770–80. doi: 10.1016/j.febslet.2011.04.066 21550344

[70] Setiadi AF Abbas AR Jeet S Wong K Bischof A Peng I. IL-17A Is Associated With the Breakdown of the Blood-Brain Barrier in Relapsing-Remitting Multiple Sclerosis. J Neuroimmunol (2019) 332:147–54. doi: 10.1016/j.jneuroim.2019.04.011 31034962

[71] Christy AL Walker ME Hessner MJ Brown MA. Mast Cell Activation and Neutrophil Recruitment Promotes Early and Robust Inflammation in the Meninges in EAE. J Autoimmun (2013) 42:50–61. doi: 10.1016/j.jaut.2012.11.003 23267561

[72] Valentin-Torres A Savarin C Barnett J Bergmann CC. Blockade of Sustained Tumor Necrosis Factor in a Transgenic Model of Progressive Autoimmune Encephalomyelitis Limits Oligodendrocyte Apoptosis and Promotes Oligodendrocyte Maturation. J Neuroinflamm (2018) 15(1):121. doi: 10.1186/s12974-018-1164-y PMC5916830 29690885

[73] Valentin-Torres A Savarin C Hinton DR Phares TW Bergmann CC Stohlman SA. Sustained TNF Production by Central Nervous System Infiltrating Macrophages Promotes Progressive Autoimmune Encephalomyelitis. J Neuroinflamm (2016) 13:46. doi: 10.1186/s12974-016-0513-y PMC4763407 26906225

[74] Rahman MT Ghosh C Hossain M Linfield D Rezaee F Janigro D. IFN-γ, IL-17A, or Zonulin Rapidly Increase the Permeability of the Blood-Brain and Small Intestinal Epithelial Barriers: Relevance for Neuro-Inflammatory Diseases. Biochem Biophys Res Commun (2018) 507(1–4):274–9. doi: 10.1016/j.bbrc.2018.11.021 30449598

[75] Liu KK Dorovini-Zis K. Differential Regulation of CD4+ T Cell Adhesion to Cerebral Microvascular Endothelium by the β-Chemokines CCL2 and CCL3. Int J Mol Sci (2012) 13(12):16119–40. doi: 10.3390/ijms131216119 PMC3546682 23203188

[76] Cui LY Chu SF Chen NH. The Role of Chemokines and Chemokine Receptors in Multiple Sclerosis. Int Immunopharmacol (2020) 83:106314. doi: 10.1016/j.intimp.2020.106314 32197226 PMC7156228

[77] Masson D Tschopp J. A Family of Serine Esterases in Lytic Granules of Cytolytic T Lymphocytes. Cell (1987) 49(5):679–85. doi: 10.1016/0092-8674(87)90544-7 3555842

[78] Grossman WJ Verbsky JW Tollefsen BL Kemper C Atkinson JP Ley TJ. Differential Expression of Granzymes A and B in Human Cytotoxic Lymphocyte Subsets and T Regulatory Cells. Blood (2004) 104(9):2840–8. doi: 10.1182/blood-2004-03-0859 15238416

[79] Hahn S Gehri R Erb P. Mechanism and Biological Significance of CD4-Mediated Cytotoxicity. Immunol Rev (1995) 146:57–79. doi: 10.1111/j.1600-065X.1995.tb00684.x 7493761

[80] Shi L Kraut RP Aebersold R Greenberg AH. A Natural Killer Cell Granule Protein That Induces DNA Fragmentation and Apoptosis. J Exp Med (1992) 175(2):553–66. doi: 10.1084/jem.175.2.553 PMC2119135 1732416

[81] Lin L Couturier J Yu X Medina MA Kozinetz CA Lewis DE. Granzyme B Secretion by Human Memory CD4 T Cells Is Less Strictly Regulated Compared to Memory CD8 T Cells. BMC Immunol (2014) 15:36. doi: 10.1186/s12865-014-0036-1 25245659 PMC4195902

[82] Broux B Markovic-Plese S Stinissen P Hellings N. Pathogenic Features of CD4+CD28- T Cells in Immune Disorders. Trends Mol Med (2012) 18(8):446–53. doi: 10.1016/j.molmed.2012.06.003 22784556

[83] Broux B Pannemans K Zhang X Markovic-Plese S Broekmans T Eijnde BO. CX(3)CR1 Drives Cytotoxic CD4(+)CD28(-) T Cells Into the Brain of Multiple Sclerosis Patients. J Autoimmun (2012) 38(1):10–9. doi: 10.1016/j.jaut.2011.11.006 22123179

[84] Peeters LM Vanheusden M Somers V Van Wijmeersch B Stinissen P Broux B. Cytotoxic CD4+ T Cells Drive Multiple Sclerosis Progression. Front Immunol (2017) 8:1160. doi: 10.3389/fimmu.2017.01160 28979263 PMC5611397

[85] Pradella F Boldrini VO Marques AM Morais GAD Francelin C Cocenza RS. Cytotoxic Activity of CD4 T Cells During the Early Stage of Autoimmune Neuroinflammation. bioRxiv (2020) 2020.03.10.985614. doi: 10.1101/2020.03.10.985614

[86] Raveney BJE Sato W Takewaki D Zhang C Kanazawa T Lin Y. Involvement of Cytotoxic Eomes-Expressing CD4(+) T Cells in Secondary Progressive Multiple Sclerosis. Proc Natl Acad Sci USA (2021) 118(11):e2021818118. doi: 10.1073/pnas.2021818118 33836594 PMC7980371

[87] Klotz L Kuzmanov I Hucke S Gross CC Posevitz V Dreykluft A. B7-H1 Shapes T-Cell-Mediated Brain Endothelial Cell Dysfunction and Regional Encephalitogenicity in Spontaneous CNS Autoimmunity. Proc Natl Acad Sci USA (2016) 113(41):E6182–91. doi: 10.1073/pnas.1601350113 PMC5068266 27671636

[88] Fissolo N Matute-Blanch C Osman M Costa C Pinteac R Miró B. CSF SERPINA3 Levels Are Elevated in Patients With Progressive MS. Neurol Neuroimmunol Neuroinflamm (2021) 8(2):e941. doi: 10.1212/NXI.0000000000000941 33436375 PMC8105904

[89] Haile Y Carmine-Simmen K Olechowski C Kerr B Bleackley RC Giuliani F. Granzyme B-Inhibitor Serpina3n Induces Neuroprotection *In Vitro* and *In Vivo* . J Neuroinflamm (2015) 12:157. doi: 10.1186/s12974-015-0376-7 PMC4558826 26337722

[90] Haile Y Simmen KC Pasichnyk D Touret N Simmen T Lu JQ. Granule-Derived Granzyme B Mediates the Vulnerability of Human Neurons to T Cell-Induced Neurotoxicity. J Immunol (2011) 187(9):4861–72. doi: 10.4049/jimmunol.1100943 21964027

[91] Rone MB Cui QL Fang J Wang LC Zhang J Khan D. Oligodendrogliopathy in Multiple Sclerosis: Low Glycolytic Metabolic Rate Promotes Oligodendrocyte Survival. J Neurosci (2016) 36(17):4698–707. doi: 10.1523/JNEUROSCI.4077-15.2016 PMC6601725 27122029

[92] Larochelle C Metz I Lecuyer MA Terouz S Roger M Arbour N. Immunological and Pathological Characterization of Fatal Rebound MS Activity Following Natalizumab Withdrawal. Mult Scler (2017) 23(1):72–81. doi: 10.1177/1352458516641775 27037182

