# Supplementary material for: Contact-Dependent Granzyme B-Mediated Cytotoxicity of Th17-Polarized Cells Toward Human Oligodendrocytes
Source: Front Immunol. 2022 Apr 11;13:850616. doi: 10.3389/fimmu.2022.850616 (PMC9035748; doi:10.3389/fimmu.2022.850616)
Supplement: Supplementary file 1 [file DataSheet_1.docx]

Supplementary Material

# Supplementary Figures and Tables

## Supplementary Figures


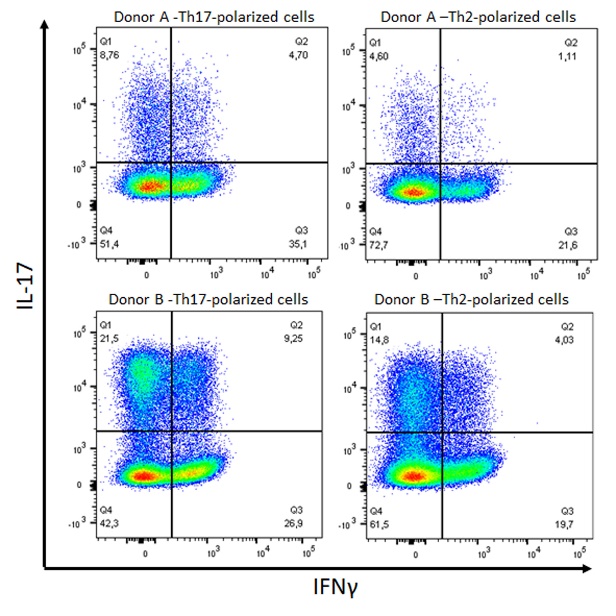


**Supplementary Figure 1: Expression of cytokines by human Th2-polarized and Th17-polarized cells after activation for 6 days in vitro**

Representative flow cytometry plots (n = 2 donors) showing expression of IL-17 and IFNγ by human memory CD4 T cells activated with αCD3/αCD28 for 6 days in the presence of IL-23 (Th17 polarization) or IL-4 and IL-2 (Th2 polarization).

##
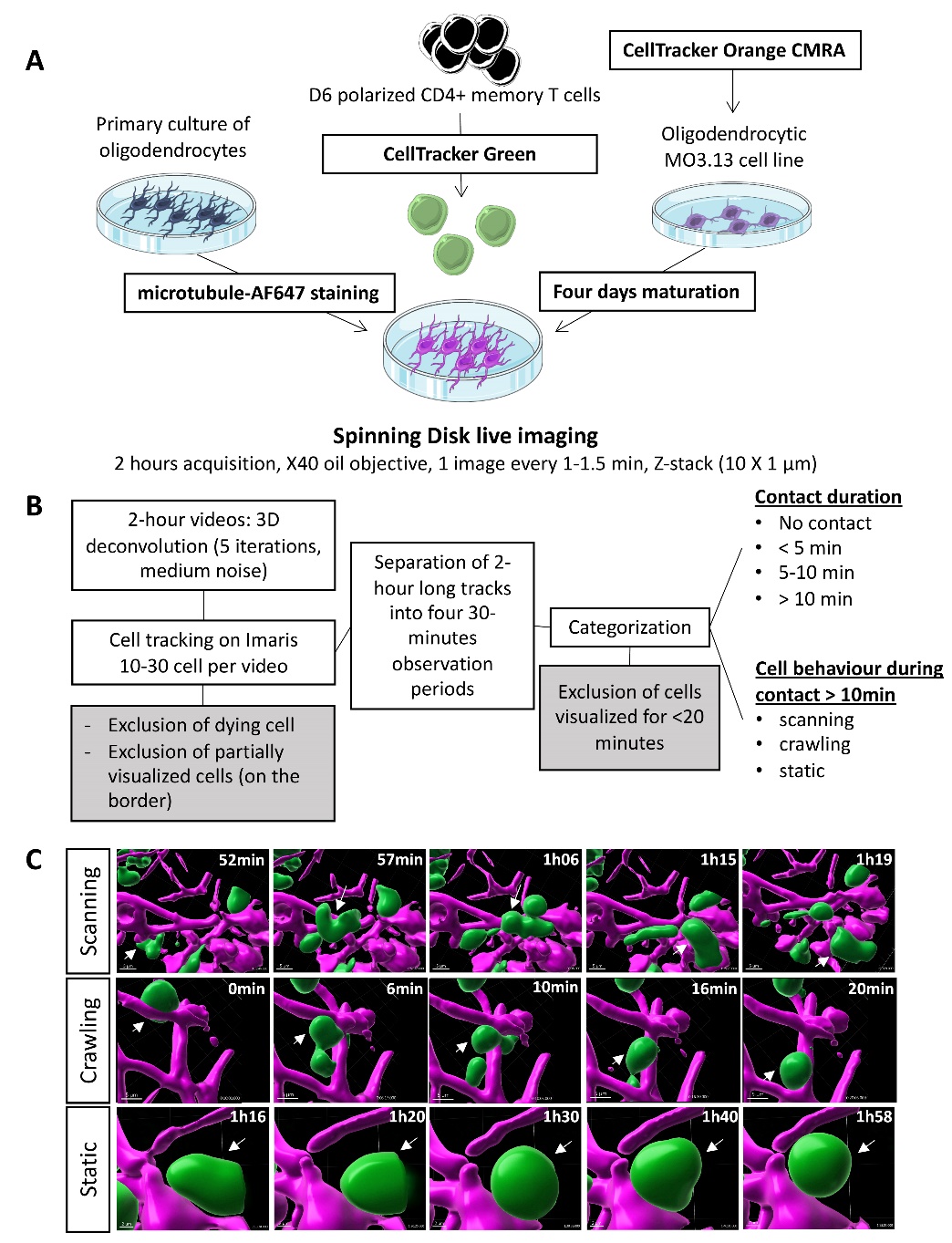


**Supplementary Figure 2: Experimental design and analysis of live imaging of OLs/T cells interactions**

(A) Schematic of OLs and CD4^+^ T cells preparation for fluorescent live imaging and (B) analysis pipeline of the obtained movies. In addition to categorization based on duration of contact with primary human OLs or human MO3.13 oligodendrocytic cell line, the predominant behavior of T cells displaying contact > 10 min was characterized as scanning, crawling or static. (C) Representative examples of scanning (upper panels), crawling (middle panels) and static (lower panels) cell behavior during contact. All images were generated from live imaging videos of Th17-polarized cells (green) and primary OLs (magenta) coculture with Imaris surface module. Scale bars = 5μm (scanning and crawling) or 2μm (static).

**Supplementary Figure 3: Coating condition for OLs does not influence mobility of polarized T cells**

(A) Representative tracks from Th2 and Th17-polarized cells with or without poly-L-lysine/ECM plating, scale bar = 20 μm. (B) Mean speed of polarized T cells in these conditions. A dashed line delimits mean speed of 2 μm/min and mean is represented by a diamond; n = 3 donors, n = 85 Th17-polarized cells no plating, n = 66 Th17-polarized cells plating, n = 105 Th2-polarized cells no plating, n = 119 Th2-polarized cells plating. Wilcoxon rank test, **p<0.01.


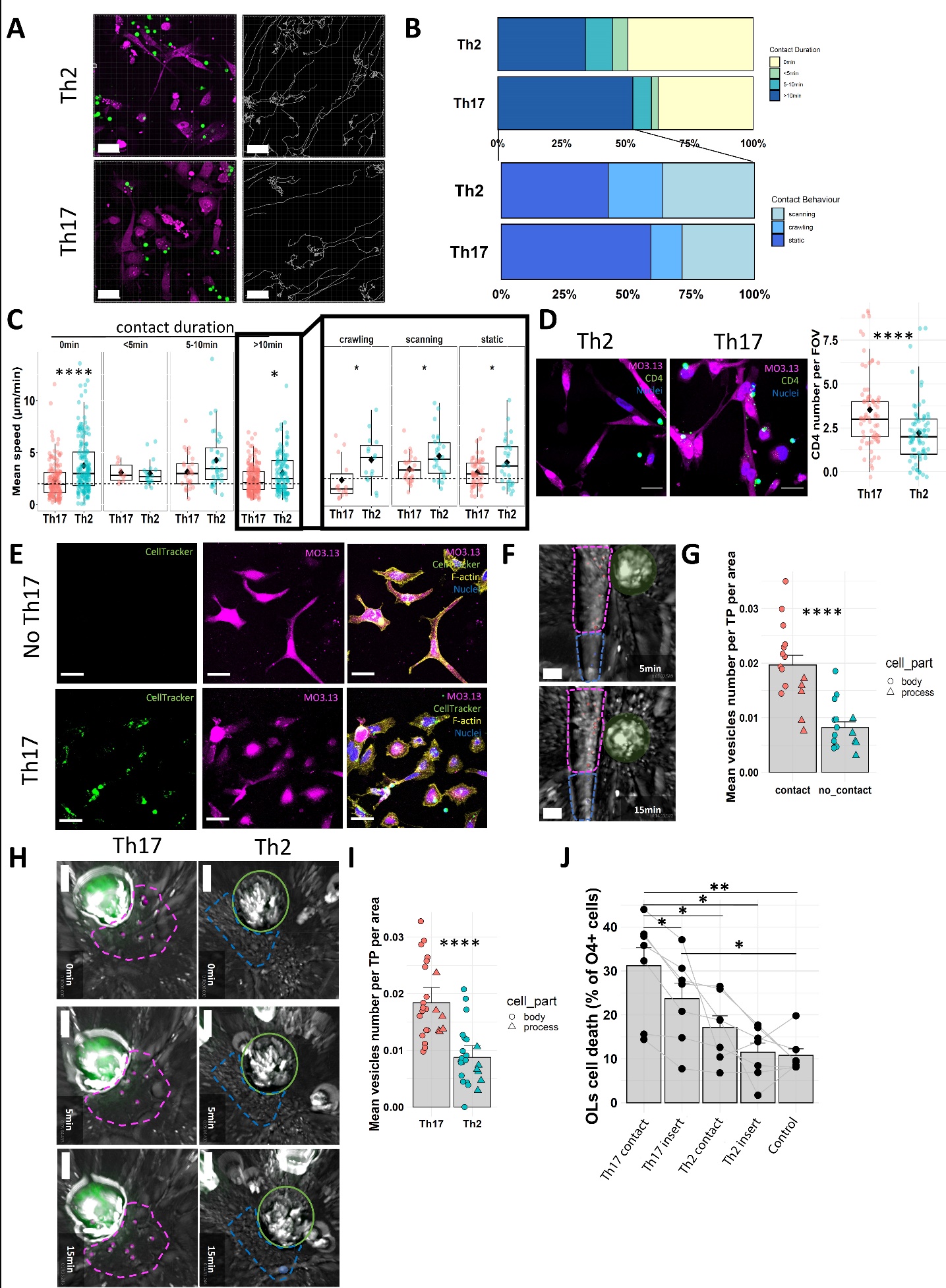


**Supplementary Figure 4: Active interactions of Th17 cells with MO3.13 cells leads to cytotoxicity toward MO3.13 cells**

(A) Representative images and tracks of Th2- or Th17-polarized cells in culture with human oligodendrocytic cell line MO3.13. Scale bar = 80μm. (B) Proportion of T cells according to duration of contact with MO3.13 (no contact, <5 min, 5-10 min or >10 min) and contact behavior (scanning, crawling, static), n = 5 donors. (C) Mean speed of T cells in coculture with MO3.13 according to contact duration (left panel) and contact behavior (right panel) among T cells displaying longer contacts (>10 min). Wilcoxon test, diamond = mean, n = 5 donors. (D) Representative images of polarized CD4^+^ T cells–MO3.13 cells coculture after 4h and quantification of CD4^+^ cell number per field of view (FOV), n = 6 donors, diamond = mean. Scale bar = 40μm. (E) Representative confocal images of live imaging MO3.13 cells in coculture with polarized Th17 cells or not. Polarized Th17 cells were loaded with CellTracker green before coculture, MO3.13 cells with Orange CMRA. Scale bar = 40 μm. (F) Representative image of live imaging from Th17 polarized cells-MO3.13 cells coculture (brightfield) and (G) quantification of number of vesicles per time point (TP) per area of MO3.13 in contact with T cells vs areas without contact with T cells (vesicle number/TP/µm^2^). Scale bar = 5 μm, T cells are highlighted in green, vesicles are designed by spots, areas of interest are delimited by a dashed line (magenta = contact, blue = no contact). Unpaired t-test, n = 4 donors, each dot represents one area of interest. (H) Representative image of live imaging from Th17- and Th2-polarized cells-MO3.13 cells coculture (brightfield with fluorescence) and (I) quantification of vesicles number per TP per area of interest in MO3.13 in contact with Th2 or with Th17 (magenta = Th17, blue = Th2). Wilcoxon test, n = 6 donors, each dot represents one area of interest. (J) Quantification of MO3.13 cell death after 16h of direct coculture with polarized T cells (contact) or separated by a permeable membrane (insert) at a 1:10 ratio. Control condition = no T cells. Each dot represents one donor, n = 7 donors, one way ANOVA followed by Tukey’s multiple comparisons. * p<0.05; ** p<0.01; **** p<0.0001.


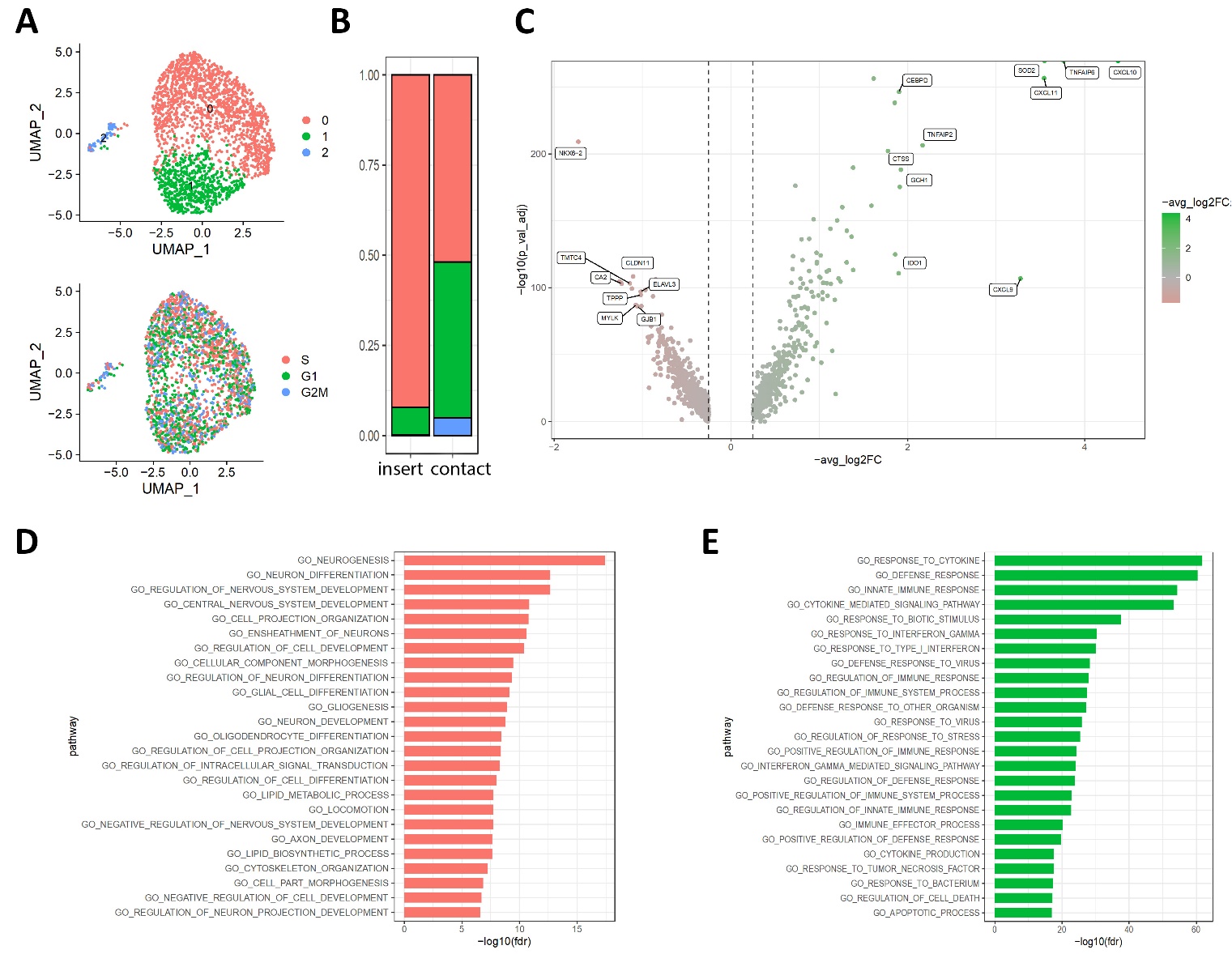


**Supplementary Figure 5: single cell RNA sequencing shows a transcriptional shift in primary human OLs in direct contact with Th17-polarized cells**

(A) UMAP of the re-clustered oligodendrocyte (OLs) populations. (B) Proportion of each unbiased clusters across the two conditions (OLs separated from Th17-polarized cells by an insert versus in direct contact). C) Volcano plot (x = average log2 fold-change, y = -log10(adjusted P-value)) showing differentially expressed genes (DEGs) between OL subpopulations 0 and 1, (cluster 1, enriched in direct contact condition, and cluster 0, enriched in insert condition). Colors are associated with the cluster color in which a gene is upregulated. (D, E) Top 25 most significant pathways from a gene set enrichment analysis on Gene Ontology: Biological Processes using a fisher-test, (D) for genes associated with cluster 0 (insert) and (E) for genes associated with cluster 1 (contact).

**Supplementary Figure 6: Luminex assay on supernatant of cocultures of polarized human Th2- or Th17-polarized cells with MO3.13 cells**

A) Concentration of analytes measured by a Luminex assay in supernatants from human polarized-T cells in direct contact or not with MO3.13 cells. Each dot represents one donor. n = 4 donors. Friedman test with Dunn’s method. *p<0.05; **p<0.01.


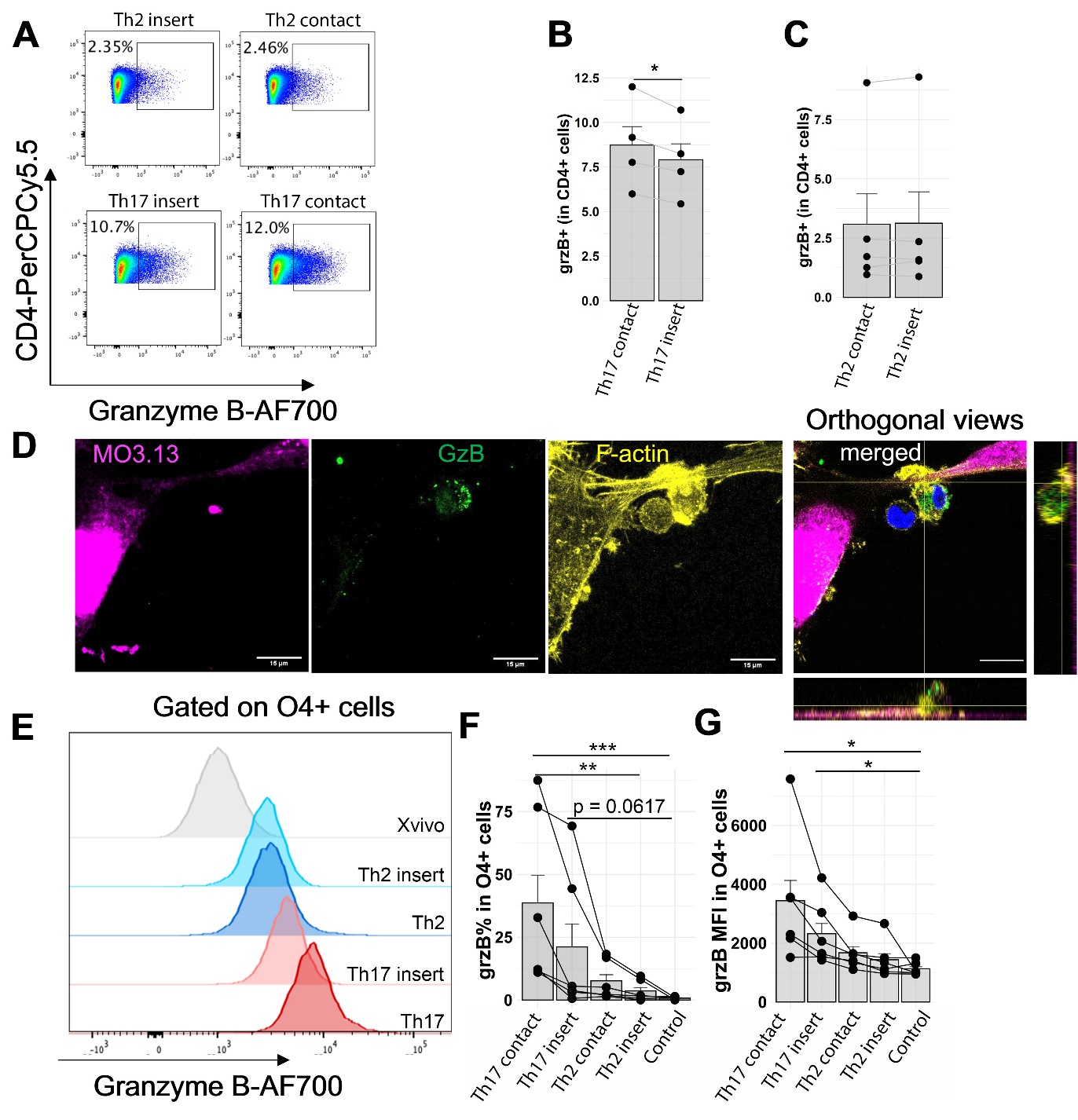


**Supplementary Figure 7: Direct contact with Th17-polarized cells results in granzyme B-positive MO3.13 cells**

(A) Representative FACS pseudocolor dot plots and (B-C) quantification of granzyme B^+^ CD4^+^  cells percentage in polarized CD4^+^ T cells after 16h coculture with MO3.13 cells (no activation with PMA-ionomycin), Paired t-test, n = 4 donors. (D) Representative immunofluorescence images of Th17-polarized cells–MO3.13 coculture after 4 hours of coculture. Green = granzyme B, yellow = F-actin, magenta = Orange CMRA (MO3.13), blue = nuclei. Scale bar = 15μm. Representative of n = 4 donors. (E) Representative FACS histogram and quantification of (F) granzyme B^+^ O4^+^ cells percentage and (G) granzyme B MFI in O4^+^ cells after 16 hours coculture with MO3.13 cells, Friedman test followed by Dunn’s, n = 6 donors. *p<0.05; ** p<0.01; *** p<0.001.

**Supplementary Figure 8: Granzyme B blocker Ac-IEPD-CHO is not toxic to MO3.13 cells at ≤50 µg/ml and serpina3N natural granzyme blocker protects MO3.13 cells from cytotoxicity mediated by Th17-polarized cells**

(A) Percentage of MO3.13 cell death as measured by FACS (LIVE/DEAD Amcyan staining) after 16h incubation with granzyme blocker Ac-IEPD-CHO (Ac) or DMSO control, n = 2-5 per condition, dot = mean. (B) MO3.13 cell death as measured by FACS (LIVE/DEAD Amcyan staining) after 16h incubation with Th17 cells pre-treated with granzyme blocker serpina3N or TrisNaCl (vehicle) control, each dot represents one donor, n = 3-6 donors, diamond = mean, paired t-test, p = 0.19 for 1μg/mL concentration and p = 0.065 for 2μg/mL concentration.

**
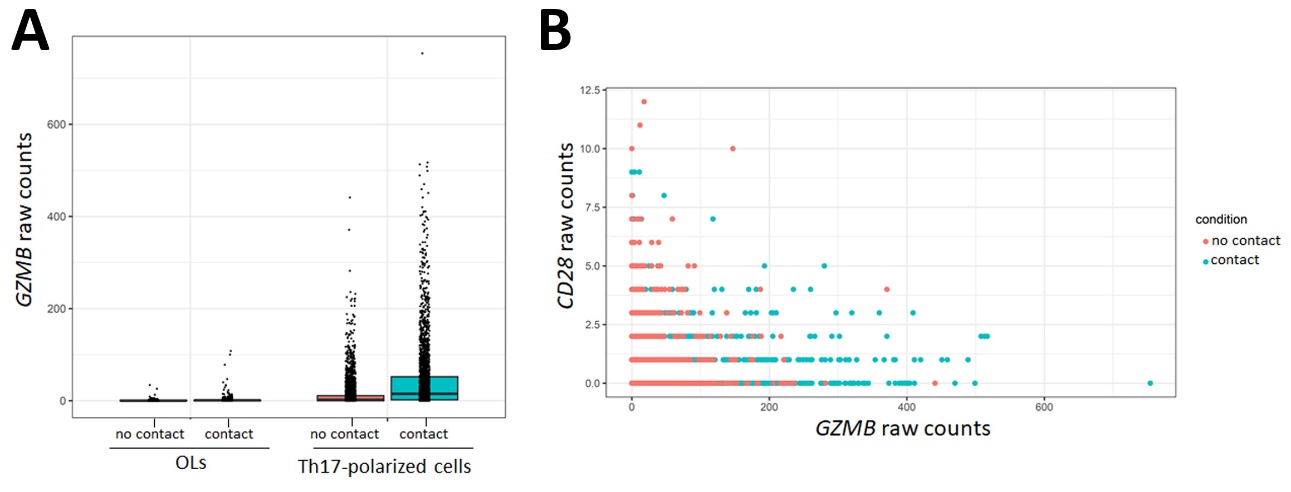
Supplementary Figure 9: Low expression of *GZMB* in primary OLs after contact with Th17-polarized cells and in Th17-polarized cells expressing *CD28* (single cell RNA sequencing)**

(A) Raw counts for *GZMB* in human primary OLs and Th17-polarized cells across the two conditions (no contact: OLs separated from Th17-polarized cells by an insert; contact: OLs in direct contact with Th17-polarized cells), scRNAseq. (B) Correlation between *CD28* raw counts and GZMB raw counts in human Th17-polarized cells in direct contact with OLs or separated by an insert, scRNAseq.

## Supplementary Tables

| **Common core top DEGs in Th17 cells** | **Name** | **Function** | **References** |
| --- | --- | --- | --- |
| SOD2 | super oxide dismutase 2 | Anti-oxidative and anti-apoptotic mitochondrial protein, target of master redox regulator transcription factor *Nrf2*, converts superoxide to H2O2 which acts on NF-κB and simulates IL-2 production, expressed by activated human memory CD4 T cells and murine Th17 cells | (1-3) |
| IRF1 | interferon regulatory factor 1 | Transcriptional regulator implicated in innate and adaptive immune response to viruses and bacteria, apoptosis, tumor suppression, regulation of transcription of IFN and IFN-inducible genes, Th1 development and induction of IL-10-producing Tr1. Knock-out of IRF1 is associated with EAE worsening. | (4, 5) |
| RUNX3 | RUNX family transcription factor 3 | Transcription factor implicated in regulation of genes associated with lymphocyte expansion, activation, upregulation of cytotoxic molecules, migration. Knockdown decrease expression of perforin and granzyme B by activated CD4 T cells. | (6, 7) |
| CHMP5 | Charged multivesicular body protein 5 | Involved in receptor degradation, formation of endocytic multivesicular bodies, TCR-mediated signaling and T cell development through stabilization of pro-survival Bcl-2 | (8, 9) |
| TPI1 | Triosephosphate isomerase 1 | Enzyme implicated in glucose metabolism, participate to metabolic reprogramming during T cell activation | (10, 11) |
| HSPD1 | Heat shock protein family D (Hsp60) Member 1 | Mitochondrial protein, up-regulated in activated T cells, epitopes of Hsp60 can be presented by T cells on MHC molecule, implicated in downregulation of inflammation | (12) |
| DYNLT1 | Dynein light chain Tctex-Type 1 | Accessory component to the cytoplasmic dynein 1 complex implicated in retrograde transport of vesicles along microtubules, could be implicated in T cell cytokinesis | (13) |
| ADAM19 | ADAM metallopeptidase domain 19 | Membrane-anchored protein implicated in cell migration, adhesion, interactions, neurogenesis, overexpressed in effector vs naive T cells, upregulated in CNS-infiltrating T cells in EAE | (14, 15) |
| TAP1 | Transporter 1, ATP binding cassette subfamily B member | Membrane-associated member of the ABC transporters, associated with antigen processing to load on MHC I, expressed by T cells | (16) |
| HSPA8 | Heat shock protein family D (Hsp70) Member 8 | Involved in antigenic peptide presentation by MHC II, in disassembly of clathrin-coated vesicles, regulation of lysosome activity and autophagy. Increased expression at the surface of T cells in lupus mice | (17) |
| UBE2L6 | Ubiquitin/ISG15 conjugating enzyme E2 L6 | Catalyzes attachment of ubiquitin-like modifier ISG15 to target protein, implicated in neutrophil differentiation | (18) |
| PARP14 | Poly(ADP-ribose) polymerase family member 14 | Anti-apoptotic, promotes survival of cancer cells, regulate aerobic glycolysis, could decrease STAT1 phosphorylation and enhance STAT6, promotes Th17 differentiation and T follicular helper development | (19-22) |
| MTHFD2 | Methylenetetrahydrofolate dehydrogenase (NADP+ dependent) 2, methenyltetrahydrofolate | Mitochondrial bifunctional enzyme, inhibition in CD4 T cells associated with lower EAE scores, increased T reg activity and promotes a shift towards anti-inflammatory phenotype | (23) |
| OAS1 | 2'-5'-oligoadenylate synthetase 1 | Induced by interferons, inhibits viral replication, could play a role in apoptosis, differentiation, reported as a susceptibility gene for multiple sclerosis associated with disease severity | (24) |
| SUB1 | SUB1 regulator of transcription | Induced by oxidative stress, would protect DNA from oxidative damage | (25) |
| FURIN | Furin, paired basic amino acid cleaving enzyme | Membrane bound protease, induced in activated T cells, can activate MMPs, deletion impairs T reg function, overexpression increases IL-2 levels, regulates NFAT and NF-κB transcription as well as transcription triggered by cytokine signaling in CD4 T cells, could contribute to NMDAR-associated pathology in neurons | (26-29) |
| CALR | Calreticulin | Calcium-buffering protein in the endoplasmic reticulum that can be expressed at the surface or secreted in stress conditions, implicated in cell adhesion and regulation of T cell activation, activates dendritic cells and macrophages, modulated by TNF, expressed in MS lesions and increased levels in the serum of MS patients | (30-32) |
| WARS | Tryptophanyl-TRNA synthetase 1 | Catalyzes aminoacylation of tRNA(trp) with tryptophan, induced by interferon, can be secreted and act as ligand for TLRs inducing pro-inflammatory cytokine and chemokines, decreases expression of PD-1 on T cells | (33-35) |
| IRF7 | Interferon regulatory factor 7 | Transcriptional regulator binding to interferon-stimulated response element, could play a role in EBV latency, increased expression and SNP polymorphism associated with systemic lupus erythematosus but deletion increases EAE severity and IL-1β, IL-17, CCL2 and CXCL10, drives IFNα/β expression in response to viruses | (36, 37) |
| LDHA | Lactate dehydrogenase A | Induced in activated T cells, mediates aerobic glygoclysis, promotes IFN-γ expression and Th17 pathologic responses, deletion protects mice against autoimmunity in Foxp3 mutated Scurfy mice, deletion in CD4 T cells confers EAE resistance | (38, 39) |
| XAF1 | X-linked inhibitor of apoptosis associated factor 1 | Regulator of IAP proteins (inhibitor of apoptosis protein), triggered by IFNs, counteracts anti-caspase activity of BIRC4, mutual antagonism with MT2A, upregulated in activated T cells, upregulated in EAE and reducing levels of XIAP is protective in EAE through increased apoptosis of activated T cells infiltrating the CNS, overexpression worsens EAE and is associated with resistance of T cells to apoptosis | (40-43) |
| DNAJA1 | DnaJ heat shock protein family (Hsp40) member A1 | Co-chaperone for Hsp70 (which is upregulated in MS lesions), inhibits translocation of BAX to mitochondria in the context of cellular stress (anti-apoptotic), Hsp40 antigens decreases proliferation of T cells from rheumatoid arthritis patients | (44-46) |
| SURF4 | Surfeit 4 | Cargo receptor of endoplasmic reticulum implicated in secretion, selectively transports lipoproteines from the endoplasmic reticulum, facilitates STING retrieval at the endoplasmic reticulum-Golgi complex, modulates calcium entry in lymphocytes | (47-50) |
| SPATS2L | Spermatogenesis associated serine rich 2 like | Poly(A) RNA binding, associated with gene expression and ribosomal biogenesis, upregulated in treated MS, could be more abundant in the cerebrospinal fluid of MS patients showing disability progression, overexpressed in T cells from patients with psoriasis, increased expression following treatment with IFNβ in MS | (51-54) |
| EPSTI1 | Epithelial stromal interaction 1 | Interferon-response gene, enriched in pathogenic Th17 cells (human and mice), reduced by methotrexate treatment in rheumatoid arthritis patients, failitates activation of NF-κB signalling | (55-57) |
| ALDOA | Aldolase, fructose-biphosphonate A | Glycolytic enzyme, inhibition of ALDOA suppresses classical activation of NLRP3 inflammasome and triggers mitophagy, induced in T cells in hypoxic conditions | (58, 59) |
| CREM | CAMP responsive element modulator | Transcriptional regulator binding to the cAMP response element (CRE), in T lymphocytes upregulates IL-17A gene, increased in T cells from patients with systemic lupus erythematosus, linked to low expression of PD-1 on CD4 T cells | (60, 61) |
| ISG15 | ISG15 ubiquitin like modifier | Ubiquitin-like protein induced by interferon and other pathogenic stimuli, implicated in immune response to viral infection, can be secreted and induce proliferation of NK and chemotaxis for neutrophils, secreted form interacts with ITGAL leading to secretion of IFN-γ and IL-10, upregulated at specific timepoints in EAE and cuprizone models | (62, 63) |
| HSP90AA1 | Heat shock protein 90 alpha family class A member 1 | Stress inducible isoform of Hsp90, chaperone protein, in human T cells is increased by IL-2, IL-4 and IL-13, differentially expressed in human MS cortical lesions | (64-66) |
| NAPA | NSF attachment protein alpha | Member of the SNAP family with an essential role in vesicular transport, docking and fusion of vesicles to target membranes (SNAP-SNARE complex), member of the CRAC channel complex, reduced expression leads to altered CD4 T cell signaling and gene expression, decreased cytokine production and lower proliferation | (67) |
| EIF2AK2 | Eukaryotic translation initiation factor 2 alpha kinase 2 | Serine/threonine kinase induced by IFN, inhibits viral synthesis, implicated in cell proliferation and promotes apoptosis, pro-inflammatory, activates NK-κB pathway, implicated in inflammasome priming, rare genetic variants associated with multiple sclerosis, protein expression in CD4+ T cells from MS patients is different compared to controls | (68-70) |
| UBE2S | Ubiquitin conjugating enzyme E2 S | Implicated in the formation of polyubiquitin chains that play a role in cell cycle and TLR signaling, is inhibited by dimethyl fumarate, accelerates cell cycle, promotes proliferation and survival (anti-apoptotic) of cancer cells | (71-73) |
| IL2RA | Interleukin 2 receptor subunit alpha (CD25) | Specifically binds IL-2, induced by T cell activation and provides positive feedback loop, induces IFN-γ and also GM-CSF in human cells, implicated in Treg survival and activity, SNPs associated with multiple sclerosis influence expression of IL2RA (CD25) on CD4+ T cells, target of daclizumab (former MS disease-modifying therapy) | (74-78) |
| SRSF7 | Serine and arginine rich splicing factor 7 | Implicated in mRNA splicing and mRNA export and translation. Target of miR-181a-5p, which is up-regulated in CSF cells of RRMS patients. Increased upon KO of AIM2 in Tregs which restrains autoimmunity and enhances stability of Tregs. Implicated in splicing Fas and inhibiting apoptosis in human cell lines. | (79-81) |
| PTGDS | Prostaglandin D2 Synthase | Catalyzes conversion of PGH2 to PGD2, which functions as a neuromodulator and trophic factor in the CNS. PTGDS is mostly expressed in the brain and is involved in multiple CNS functions, such as sleep, allodynia and protection against plaques. It is known to be expressed in OPCs | (82-84) |
| BST2 | Bone marrow stromal cell antigen 2 | Induced by interferon signaling and may play a role in pre-B cell growth and HIV-1 pathogenesis. The protein was originally cloned from a RA derived cell line and plays a largely antiviral role in cells. It is expressed in B cells, T cells, monocytes, macrophages and plasmacytoid dendritic cells | (85-87) |
| TPM4 | Tropomyosin alpha4 chain | Member of the tropomyosin family, binds to actin filaments in cells and plays a role in calcium dependent regulation for striated muscle contraction. In non muscle cells, this protein stabilizes actin filaments and is known to localize to the post synaptic region of the CNS in mouse hippocampi | (88, 89) |
| IFI6 | Interferon alpha inducible protein 6 | Induced by interferon and is a prosurvival protein that plays a role in the regulation of apoptosis | (90, 91) |
| NARF | Nuclear Prelamin A recognition factor | The protein encoded by this gene and binds to the prenylated prelamin A carboxyl-terminal tail domain and removes the cysteine residue so that prelamin A is processed into mature lamin A. NARF has been linked to mitochondrial defects, iron dysregulation and MS pathology | (92, 93) |
| IFNG | Interferon gamma | A type II interferon that is primarily secreted by T cells and NK cells; can regulate Th1/Th2 balance, promote activation of macrophages, enhance antigen presentation and a lot of other factors. Highly expressed in MS lesions | (94, 95) |
| HSPA5 | Binding immunoglobulin protein | This protein is a ER chaperone that plays a role in protein folding and quality control. In response to stress, this protein moves to the cell surface and serves as a receptor and associates with MHC I | (96, 97) |
| GBP1 | Guanylate Binding protein 1 | Hydrolyzes GTP to GMP and its expression is increased in response to IFNγ. GBP1 protects against apoptosis and inhibits cell proliferation during inflammation and infection. During homeostasis, it plays a role in cytoskeleton regulation and autophagy | (98-100) |
| OAS2 | 2'-5' Oligadenylate Synthetase 2 | This protein is induced by interferons and is involved in the innate immune response to viral infection. Mediates antiviral effect via both the RNASEL-dependent and independent pathways and may also play a role in apoptosis, and differentiation | (101) |
| GLUL | Glutamate Ammonia Ligase | Catalyzes the synthesis of glutamine; in the brain it regulates the levels of toxic ammonia and converst neurotoxic glutamate to harmless glutamine. It is also required for endothelial cell migration during development | (102-104) |
| GZMB | Granzyme B | A serine protease that is important for the induction of target cell apoptosis when secreted by NK cells and cytotoxic T cells. Granzyme B is also neurotoxic and target cell death occurs by triggering pyroptosis via Gasdermin E cleavage after granzyme B is delivered to the cell. | (105-108) |
| CYTIP | Cytohesin 1 Interacting Protein | The encoded protein modulates the activation of Auxin Response Factors (ARF) genes; it is weakly expressed in resting NK cells and T cells and induced during dendritic cell maturation/T cell activation. Also been implicated in modulation of LFA-1/ICAM1 interaction | (109-111) |
| HSP90AB1 | Heat Shock protein 90 alpha family class B member 1 | Part of the heat shock protein 90 family and is a molecular chaperone that regulates proteins involved in cell cycle control/signal transduction. It has been shown to play a role in inflammation, modulate transcription and have ATPase activity | (112-114) |
| SOCS1 | Suppressor of cytokine signaling 1 | Negative regulator of the JAK/STAT pathway by inhibiting JAK kinase activity. Expression of SOCS1 is increased in response to cytokine exposure. SOCS1 is expressed by immune cells and cells in the CNS and plays a role in cytokine/chemokine production, activation of microglia/astrocytes and other inflammatory processes | (115, 116) |
| ACTB | Beta actin | Encodes for one of the six actin proteins. Actin polymerization produces filaments that help maintain the cytoskeletal structure of a cell, therefore playing a role in cell motility and contraction. Beta-actin also plays a role in axon guidance, synaptogenesis and CNS disease. Loss of ACTB can result in developmental issues | (117, 118) |
| IFI35 | Interferon induced protein 35 | Is a regulator of innate immune response signaling pathways. Expression of IFI35 is induced by interferon exposure and IFI35 can be both an intracellular regulator by inhibiting NFkB (resulting in inhibition of endothelial cell proliferation) and can also function as a DAMPs to promote inflammation | (119, 120) |
| ENO1 | Enolase 1 | Enzyme that catalyzes conversion of 2-phosphoglyderate to phosphoenolpyruvate. Also plays a role in hypoxia tolerance, growth control, stimulates immunoglobulin production and can act as a receptor on leukocytes and neurons | (121, 122) |
| PRELID1 | PRELI domain containing 1 | This protein regulates the mitochondrial apoptotic pathway by promoting accumulation of cardiolipin in the mitochondria. It also is expressed in Th cells and regulates their differentiation by inhibiting STAT6 | (123, 124) |
| MX1 | MX Dynamin Like GTPase 1 | Antiviral interferon-inducible GTPase; Is known to inhibit replication of multiple viruses and enhance ER stress mediated cell death after influenza virus infection | (125-127) |
| LY6E | Lymphocyte Antigen 8 family member E | Cell surface protein that regulates T cell physiology (proliferation, differentiation and activation). Interferes with spike protein mediated membrane fusion and thereby restricts entry of human coronaviruses. | (128-130) |
| RBM8A | RNA Binding Motif Protein 8A | Core component of the spliceosome/exon junction complex (EJC) and is required for pre-mRNA splicing, therefore playing a role in nuclear RNA export, RNA localization within the cell and translation efficiency. Also part of the MAGOH-RBM8A complex, which is involved in the nonsense-mediated decay pathway and inhibits formation of proapoptotic isoforms of BclX. Involved in proliferation/differentiation of neural progenitors | (131-133) |
| NDUFS5 | NADH dehydrogenase [ubiquinone] iron-sulfer proetin 5 | Subunit of mitochondrial membrane respiratory chain complex I. Complex I function is to transfer electrons from NADH to the respiratory chain. Genetic variants in complex 1 have been associated with MS | (134, 135) |
| LAP3 | Leucine Aminopeptidase 3 | This protein catalyzes the removal of N-terminal hydrophobic amino acids from various peptides and is involved in the metabolism of glutathione, suggesting it may help control cell redox. It has also been shown to promote glioma progression | (136-138) |
| PSMB3 | Proteasome 20S subunit beta 3 | This protein is a non-catalytic component of the 20S proteasome complex involved in the degradation of most intracellular proteins. This proteasome interacts with one or two proteasome activators and regulates ubiquitin-independent and dependent protein degradation. | (139-141) |
| TXN | Thioredoxin | This protein plays a role in various redox reactions and catalyzes dithiol-disulfide exchange reactions and also contributes to cellular response to Nitric Oxide. It also promotes AP-1 transcriptional activity | (142-144) |
| PSME2 | Proteasome activator subunit 2 | Part of the immunoproteasome, which forms in cells during inflammation. This protein/proteasome is required for antigen processing | (145) |
| PPA1 | inorganic pyrophosphatase 1 | Catalyzes hydrolysis of pyrophosphate to inorganic phosphate (important for phsophate metabolism in cells). Has been found to promote tumor progression in various tumor types and play a role in neurite outgrowth in cells. | (146-149) |
| CEBPB | CCAAT enhancer binding protein beta | Transcription factor that regulates expression of immune/inflammatory response genes. Inhibits proliferation in T cells and promotes differentiation into Th2 cells by repressing myc expression. CEBPB also plays a role in neurons after injury and is upregulated in MS tissue samples. | (150-153) |
| PARP9 | Poly(ADP-Ribose) polymerase family member 9 | This proteins plays a role in immune responses (like interferon mediated antiviral defense) and DNA damage repair. Promotes proinflammatory cytokine production in macrophages. | (154-157) |
| MT2A | Metallothionein 2A | Part of the metallothionein family and plays a role in antioxidant response and is important for homeostatic control of metal in cells. MT2 was also found to be present at the BBB during EAE in rats and has been shown to be neuroprotective in various diseases | (158-160) |
| ACTG1 | Actin gamma 1 | Cytoplasmic actin found in all cell types and is involved in cell motility. Mutations in ACTG1 are linked to Baraitser Winter syndrome and nonsyndromic hearing loss | (161, 162) |
| CHCHD2 | Coiled-coil-helix-coiled-coil-helix domain containing 2 | Mitochondrial protein that regulates oxidative phosphorylation and acts as a transcription factor for cytochrome c oxidase subunit. CHCHD2 also regulates cell migration and apoptosis. Loss of CHCHD2 results in an age-dependent loss of dopamine neurons and mutations in this gene are associated with ALS/FTD | (163-166) |
| IL2RB | interleukin 2 receptor subunit beta | Subunit of the IL2 receptor that is involved in T cell mediated immune responses. Also involved in stimulation of neutrophil phagocytosis via IL15. Polymorphisms in IL2RB have been identified in MS patients | (167-169) |
| TYMP | thymidine phosphorylase | Angiogenic protein that promotes growth of endothelial cells and is highly specific to act only on endothelial cells. It has also been implicated in cancer and BBB integrity | (170-173) |
| SATB1 | Special AT-rich sequence binding protein 1 | Matrix protein that binds to DNA and modulates genes essential for maturation of CD8SP T cells. Also found to promote differentiation of Neural progenitor cells in the SVZ | (174, 175) |
| PSMB9 | proteasome 20S subunit beta 9 | Subunit of the core proteosome 20S and replaces PSMB6 in the immunoproteasome, which plays a role in antigen presentation and MHC expression. | (176, 177) |
| PGK1 | phosphoglycerase kinase 1 | Glycolytic enzyme that converts 1,3-diphosphoglycerate to 3-phosphoglycerate and regulates Keap1-NRF2 signaling. Mutation in this gene has been associated with neurological dysfunctions | (178, 179) |

**Supplementary Table 1**: Common core genes (71 genes) upregulated in Th17 cell clusters C1-C3 following coculture in condition of direct contact with mature oligodendrocytes (OLs) compared to in condition of separation by a porous membrane (insert), single cell RNA sequencing, 1 OL donor and 1 T cell donor.

| DEGs OLs (cluster 0 vs. cluster 1) | p_val | avg_log2FC | pct.1 | pct.2 | p_val_adj |
| --- | --- | --- | --- | --- | --- |
| CXCL10 | 0 | -4,37582 | 0,635 | 0,977 | 0 |
| SOD2 | 0 | -3,54612 | 0,768 | 0,995 | 0 |
| TNFAIP6 | 0 | -3,76238 | 0,298 | 0,972 | 0 |
| CXCL11 | 1,5E-261 | -3,54155 | 0,197 | 0,834 | 2,4E-257 |
| GBP1 | 2,4E-261 | -1,61425 | 0,992 | 1 | 4E-257 |
| CEBPD | 1,9E-251 | -1,90189 | 0,698 | 0,992 | 3,1E-247 |
| NFKBIA | 3,8E-243 | -1,85272 | 0,618 | 0,98 | 6,2E-239 |
| NKX6-2 | 5E-214 | 1,724283 | 0,99 | 0,886 | 8,2E-210 |
| TNFAIP2 | 2,3E-211 | -2,16637 | 0,42 | 0,925 | 3,8E-207 |
| ICAM1 | 3,6E-207 | -1,77707 | 0,498 | 0,945 | 6E-203 |
| IFIH1 | 1,2E-194 | -1,38066 | 0,908 | 0,998 | 2E-190 |
| CTSS | 3,9E-193 | -1,92333 | 0,198 | 0,77 | 6,5E-189 |
| B2M | 3,9E-181 | -0,72922 | 1 | 1 | 6,5E-177 |
| GCH1 | 3,1E-180 | -1,91009 | 0,114 | 0,657 | 5,1E-176 |
| GBP2 | 2,7E-166 | -1,58997 | 0,753 | 0,98 | 4,5E-162 |
| NAMPT | 4,5E-165 | -1,25989 | 0,912 | 0,994 | 7,4E-161 |
| PPA1 | 4,1E-156 | -0,93517 | 0,976 | 1 | 6,8E-152 |
| WTAP | 4,1E-155 | -1,20083 | 0,7 | 0,937 | 6,8E-151 |
| PLSCR1 | 5,5E-149 | -1,12483 | 0,647 | 0,966 | 9,2E-145 |
| CD38 | 1,7E-147 | -1,31015 | 0,129 | 0,644 | 2,8E-143 |
| TNFAIP3 | 4,3E-143 | -1,36514 | 0,227 | 0,745 | 7,2E-139 |
| PSME2 | 1,7E-141 | -0,88487 | 0,988 | 1 | 2,8E-137 |
| HLA-B | 4,6E-141 | -0,79752 | 0,999 | 1 | 7,5E-137 |
| CD274 | 4E-131 | -0,96784 | 0,802 | 0,982 | 6,6E-127 |
| GBP4 | 8,4E-130 | -1,85588 | 0,582 | 0,879 | 1,4E-125 |
| NCOA7 | 4,1E-129 | -1,01592 | 0,799 | 0,954 | 6,8E-125 |
| HLA-A | 1,9E-128 | -0,70296 | 1 | 1 | 3,1E-124 |
| OPTN | 1,4E-125 | -0,8097 | 0,964 | 0,997 | 2,3E-121 |
| OAS3 | 3,6E-124 | -0,9732 | 0,682 | 0,954 | 5,9E-120 |
| BIRC3 | 7,2E-124 | -1,30871 | 0,747 | 0,986 | 1,2E-119 |
| IRF1 | 3,1E-123 | -0,97059 | 0,979 | 1 | 5,1E-119 |
| HLA-C | 8,9E-119 | -0,69825 | 1 | 1 | 1,5E-114 |
| TYMP | 3,3E-118 | -1,38253 | 0,289 | 0,654 | 5,5E-114 |
| MX1 | 5E-118 | -1,10954 | 0,841 | 0,986 | 8,3E-114 |
| FAM177A1 | 2E-117 | -0,88439 | 0,965 | 0,997 | 3,3E-113 |
| ISG15 | 3,6E-117 | -1,08544 | 0,99 | 1 | 5,9E-113 |
| IDO1 | 1,1E-115 | -1,89766 | 0,29 | 0,743 | 1,8E-111 |
| CLDN11 | 2,3E-113 | 1,104477 | 0,993 | 0,962 | 3,8E-109 |
| GLUL | 4,6E-113 | -1,00003 | 0,998 | 1 | 7,6E-109 |
| IFITM3 | 1,6E-112 | -1,01114 | 0,921 | 0,997 | 2,7E-108 |
| DAAM2 | 7,4E-112 | -1,21758 | 0,678 | 0,894 | 1,2E-107 |
| CXCL9 | 9E-112 | -3,27533 | 0,201 | 0,573 | 1,5E-107 |
| LITAF | 9,4E-112 | -0,78818 | 0,955 | 0,992 | 1,6E-107 |
| CAMK2N1 | 5,1E-111 | 0,850642 | 0,996 | 0,971 | 8,5E-107 |
| HLA-F | 9,2E-110 | -0,90364 | 0,629 | 0,928 | 1,5E-105 |
| ERMN | 1,2E-109 | 1,258928 | 0,895 | 0,639 | 2E-105 |
| IFIT2 | 1,4E-109 | -1,24 | 0,975 | 1 | 2,3E-105 |
| JUNB | 1,4E-108 | -1,04319 | 0,677 | 0,951 | 2,2E-104 |
| LAP3 | 1,4E-108 | -0,68387 | 0,986 | 1 | 2,3E-104 |
| RELA | 2,3E-108 | -1,0825 | 0,456 | 0,797 | 3,8E-104 |
| TMTC4 | 4,2E-108 | 1,141268 | 0,93 | 0,739 | 6,9E-104 |
| CA2 | 4,9E-108 | 1,234892 | 0,948 | 0,697 | 8,2E-104 |
| PARP14 | 5E-108 | -0,81529 | 0,982 | 1 | 8,3E-104 |
| PNRC1 | 8,4E-108 | -1,12926 | 0,811 | 0,962 | 1,4E-103 |
| SUB1 | 8,1E-105 | -0,7859 | 0,953 | 0,995 | 1,3E-100 |
| CALM2 | 2E-104 | 0,675193 | 1 | 0,994 | 3,4E-100 |
| TRIM2 | 2,8E-104 | 1,117924 | 0,92 | 0,639 | 4,7E-100 |
| RELB | 3,4E-104 | -0,85499 | 0,376 | 0,825 | 5,6E-100 |
| MAP6D1 | 2,4E-103 | 0,965294 | 0,549 | 0,095 | 3,9E-99 |
| ELAVL3 | 4,5E-102 | 1,019025 | 0,912 | 0,644 | 7,42E-98 |
| EPSTI1 | 7,7E-102 | -0,91325 | 0,71 | 0,943 | 1,27E-97 |
| GBP3 | 1,7E-101 | -1,03718 | 0,697 | 0,903 | 2,79E-97 |
| TPPP | 1,6E-99 | 1,017901 | 0,939 | 0,791 | 2,65E-95 |
| RNF19A | 3,8E-99 | -0,98945 | 0,563 | 0,856 | 6,36E-95 |
| OASL | 5,6E-99 | -1,09236 | 0,53 | 0,891 | 9,24E-95 |
| TIFA | 9,3E-99 | -0,76006 | 0,097 | 0,516 | 1,54E-94 |
| DLG1 | 1,67E-98 | 0,880932 | 0,986 | 0,922 | 2,76E-94 |
| ISG20 | 2,84E-98 | -0,931 | 0,932 | 0,991 | 4,69E-94 |
| DYNLT1 | 3,36E-98 | -0,70804 | 0,972 | 1 | 5,56E-94 |
| VAMP5 | 6,59E-98 | -0,73103 | 0,986 | 1 | 1,09E-93 |
| IFI44L | 2,27E-96 | -1,03642 | 0,657 | 0,932 | 3,76E-92 |
| IER3 | 8,33E-96 | -1,17474 | 0,178 | 0,584 | 1,38E-91 |
| CD47 | 1,4E-94 | -0,6615 | 0,996 | 1 | 2,31E-90 |
| MYLK | 5,97E-92 | 1,073422 | 0,95 | 0,78 | 9,88E-88 |
| RNF114 | 8,79E-92 | -0,67247 | 0,881 | 0,991 | 1,45E-87 |
| MARCKSL1 | 3,52E-91 | 1,009893 | 0,999 | 0,992 | 5,83E-87 |
| GJB1 | 5,12E-91 | 1,055915 | 0,784 | 0,404 | 8,47E-87 |
| HMGA1 | 1,7E-90 | -0,83494 | 0,916 | 0,995 | 2,81E-86 |
| UBE2Z | 7,98E-90 | -0,87104 | 0,878 | 0,966 | 1,32E-85 |
| NFKB2 | 1,28E-89 | -0,79656 | 0,282 | 0,71 | 2,12E-85 |
| VCAM1 | 7,44E-89 | -0,86973 | 0,015 | 0,313 | 1,23E-84 |
| WARS | 2,56E-88 | -0,72654 | 0,988 | 1 | 4,24E-84 |
| OAS1 | 4,75E-86 | -0,94635 | 0,586 | 0,88 | 7,86E-82 |
| MAP7 | 7,35E-85 | 0,780919 | 0,931 | 0,72 | 1,22E-80 |
| TMX1 | 2,58E-84 | -0,74337 | 0,751 | 0,934 | 4,28E-80 |
| HAPLN3 | 9,55E-83 | -0,86075 | 0,154 | 0,551 | 1,58E-78 |
| MYRF | 1,9E-80 | 0,82827 | 0,917 | 0,717 | 3,14E-76 |
| POMP | 6,17E-80 | -0,55494 | 0,989 | 1 | 1,02E-75 |
| PHLDA3 | 2,13E-79 | 0,875414 | 0,754 | 0,379 | 3,52E-75 |
| MB21D1 | 4,26E-79 | -0,76491 | 0,17 | 0,553 | 7,06E-75 |
| CD74 | 1,29E-78 | -1,08214 | 0,509 | 0,783 | 2,14E-74 |
| RNF213 | 3,95E-78 | -0,7722 | 0,963 | 0,998 | 6,54E-74 |
| LRRC4C | 6,69E-78 | -0,80676 | 0,327 | 0,694 | 1,11E-73 |
| IFIT3 | 6,93E-77 | -0,77603 | 0,988 | 1 | 1,15E-72 |
| RAPGEF5 | 4,4E-76 | 0,901938 | 0,813 | 0,467 | 7,28E-72 |
| CEBPB | 8,54E-76 | -0,84181 | 0,744 | 0,945 | 1,41E-71 |
| DHX58 | 1,67E-75 | -0,73446 | 0,433 | 0,777 | 2,76E-71 |
| TUBB4A | 3,13E-75 | 0,783109 | 0,994 | 0,98 | 5,18E-71 |
| ZNFX1 | 6,23E-75 | -0,73542 | 0,688 | 0,917 | 1,03E-70 |
| ANKS1B | 6,54E-75 | 0,67404 | 0,988 | 0,923 | 1,08E-70 |
| NFKB1 | 7,61E-75 | -0,81585 | 0,447 | 0,774 | 1,26E-70 |
| HLA-E | 1,52E-74 | -0,55504 | 0,993 | 1 | 2,51E-70 |
| IFI35 | 2,23E-74 | -0,6219 | 0,948 | 0,997 | 3,7E-70 |
| NRCAM | 3,78E-74 | -0,80935 | 0,635 | 0,888 | 6,25E-70 |
| APOL2 | 6,21E-74 | -0,72235 | 0,966 | 1 | 1,03E-69 |
| FAM222A | 2,08E-73 | 0,821902 | 0,653 | 0,269 | 3,44E-69 |
| SLC25A28 | 2,6E-73 | -0,69386 | 0,59 | 0,866 | 4,31E-69 |
| CAPN3 | 5,13E-73 | 0,811107 | 0,975 | 0,848 | 8,49E-69 |
| MIR219A2 | 1,66E-72 | 0,809903 | 0,441 | 0,081 | 2,74E-68 |
| LIFR | 4,28E-72 | -0,86882 | 0,431 | 0,728 | 7,08E-68 |
| FAM102A | 8,46E-72 | 0,8099 | 0,945 | 0,808 | 1,4E-67 |
| C1R | 1,83E-69 | -0,90346 | 0,258 | 0,564 | 3,03E-65 |
| RASSF2 | 5,52E-69 | 0,89356 | 0,875 | 0,664 | 9,14E-65 |
| CORO2B | 7,29E-69 | 0,768365 | 0,845 | 0,593 | 1,21E-64 |
| TPRN | 1,22E-68 | 0,798156 | 0,709 | 0,352 | 2,02E-64 |
| ZCCHC24 | 1,56E-68 | 0,911809 | 0,691 | 0,381 | 2,58E-64 |
| LDLRAD4 | 4,03E-68 | 0,937635 | 0,724 | 0,382 | 6,67E-64 |
| PLEKHA4 | 1,08E-67 | -0,67567 | 0,246 | 0,608 | 1,78E-63 |
| FSCN1 | 5,32E-67 | 0,924211 | 0,824 | 0,573 | 8,8E-63 |
| GLTP | 6,2E-67 | 0,77071 | 0,934 | 0,811 | 1,03E-62 |
| PIP4K2A | 6,63E-67 | 0,678717 | 0,955 | 0,891 | 1,1E-62 |
| STMN1 | 2,33E-66 | 0,712488 | 0,988 | 0,949 | 3,85E-62 |
| SYNM | 1,18E-65 | 0,807677 | 0,734 | 0,392 | 1,95E-61 |
| RSAD2 | 1,76E-65 | -0,76094 | 0,919 | 0,998 | 2,92E-61 |
| RTN4 | 1,85E-65 | 0,458678 | 1 | 1 | 3,06E-61 |
| ABHD17A | 5,24E-65 | 0,653796 | 0,972 | 0,889 | 8,68E-61 |
| DPYSL2 | 1,77E-64 | 0,658172 | 0,99 | 0,955 | 2,93E-60 |
| SLC48A1 | 1,93E-64 | 0,784703 | 0,883 | 0,653 | 3,2E-60 |
| RAB40B | 4,33E-64 | 0,743311 | 0,742 | 0,406 | 7,17E-60 |
| UGT8 | 4,94E-64 | 0,933209 | 0,975 | 0,922 | 8,18E-60 |
| BST2 | 6,94E-64 | -0,97339 | 0,322 | 0,639 | 1,15E-59 |
| BATF3 | 9,23E-64 | -0,71489 | 0,247 | 0,588 | 1,53E-59 |
| GNG7 | 1,33E-63 | 0,819154 | 0,974 | 0,896 | 2,2E-59 |
| SEPW1 | 1,69E-63 | 0,592682 | 1 | 0,992 | 2,8E-59 |
| ZFP36 | 3,76E-63 | -0,8432 | 0,243 | 0,57 | 6,23E-59 |
| ANLN | 1,42E-62 | 0,754045 | 0,826 | 0,533 | 2,36E-58 |
| OAS2 | 3,37E-62 | -0,77612 | 0,222 | 0,564 | 5,57E-58 |
| SCD5 | 3,44E-62 | 0,727023 | 0,939 | 0,803 | 5,7E-58 |
| IFI6 | 6,64E-62 | -0,65135 | 0,928 | 0,988 | 1,1E-57 |
| PML | 1,45E-61 | -0,60641 | 0,749 | 0,939 | 2,4E-57 |
| FA2H | 2,54E-61 | 0,812622 | 0,633 | 0,33 | 4,21E-57 |
| SAT1 | 3,43E-61 | -0,95334 | 0,972 | 0,991 | 5,68E-57 |
| LACTB | 5,9E-61 | -0,70116 | 0,305 | 0,633 | 9,78E-57 |
| TRIM22 | 1,02E-60 | -0,72894 | 0,64 | 0,869 | 1,69E-56 |
| ZNF267 | 1,83E-60 | -0,71147 | 0,453 | 0,768 | 3,02E-56 |
| BAZ1A | 2,51E-60 | -0,7285 | 0,611 | 0,842 | 4,16E-56 |
| TAP1 | 3,47E-60 | -0,48359 | 0,982 | 1 | 5,75E-56 |
| DDX58 | 4,72E-60 | -0,80268 | 0,62 | 0,84 | 7,82E-56 |
| CKAP4 | 7,78E-60 | -0,70628 | 0,496 | 0,762 | 1,29E-55 |
| SNTB2 | 1,32E-59 | -0,73641 | 0,62 | 0,823 | 2,18E-55 |
| WSCD1 | 1,74E-59 | 0,652663 | 0,48 | 0,144 | 2,88E-55 |
| CXXC5 | 3,23E-59 | 0,694304 | 0,813 | 0,492 | 5,35E-55 |
| CNTN2 | 3,58E-59 | 0,730486 | 0,939 | 0,868 | 5,93E-55 |
| MVB12B | 8,34E-59 | 0,706209 | 0,77 | 0,528 | 1,38E-54 |
| ARHGAP21 | 1,01E-58 | 0,553694 | 0,997 | 0,983 | 1,67E-54 |
| SAMD9L | 2,25E-58 | -0,82125 | 0,464 | 0,743 | 3,72E-54 |
| DNAJB2 | 2,86E-58 | 0,69295 | 0,704 | 0,381 | 4,73E-54 |
| AC058791.1 | 2,98E-58 | -0,84436 | 0,367 | 0,717 | 4,93E-54 |
| IL33 | 8,19E-58 | -1,14656 | 0,016 | 0,227 | 1,36E-53 |
| PXK | 2,06E-57 | 0,719382 | 0,81 | 0,531 | 3,41E-53 |
| CFLAR | 3,11E-57 | -0,59296 | 0,842 | 0,951 | 5,15E-53 |
| TNFSF10 | 4,33E-57 | -0,72293 | 0,127 | 0,41 | 7,16E-53 |
| SORT1 | 5,66E-57 | 0,635938 | 0,955 | 0,842 | 9,37E-53 |
| ELOVL1 | 5,81E-57 | 0,827173 | 0,928 | 0,793 | 9,61E-53 |
| RAB33A | 6,01E-57 | 0,703717 | 0,655 | 0,313 | 9,95E-53 |
| UBL3 | 1,24E-56 | 0,730162 | 0,938 | 0,81 | 2,05E-52 |
| CR1L | 1,45E-56 | -0,54145 | 0,13 | 0,452 | 2,41E-52 |
| RIPK2 | 1,55E-56 | -0,66052 | 0,336 | 0,644 | 2,56E-52 |
| APC2 | 4,31E-56 | 0,717495 | 0,506 | 0,187 | 7,14E-52 |
| SLAIN1 | 4,53E-56 | 0,697746 | 0,968 | 0,888 | 7,5E-52 |
| CDK18 | 5,36E-56 | 0,721103 | 0,97 | 0,943 | 8,87E-52 |
| USP14 | 1,06E-55 | -0,63244 | 0,734 | 0,891 | 1,75E-51 |
| KCNJ10 | 1,39E-55 | 0,627371 | 0,402 | 0,095 | 2,3E-51 |
| ARL9 | 3,26E-55 | -0,50638 | 0,111 | 0,406 | 5,4E-51 |
| RAB8B | 3,37E-55 | -0,63276 | 0,727 | 0,88 | 5,57E-51 |
| PVRL2 | 6,09E-55 | -0,61968 | 0,589 | 0,843 | 1,01E-50 |
| MAPK8IP1 | 6,86E-55 | 0,708946 | 0,813 | 0,561 | 1,14E-50 |
| CCNL1 | 1,58E-54 | -0,63641 | 0,649 | 0,833 | 2,61E-50 |
| BTG3 | 3,27E-54 | -0,68505 | 0,555 | 0,779 | 5,42E-50 |
| NMI | 3,93E-54 | -0,59994 | 0,823 | 0,935 | 6,51E-50 |
| LDB3 | 5,05E-54 | 0,687984 | 0,48 | 0,171 | 8,37E-50 |
| TAPBP | 7,07E-54 | -0,53888 | 0,912 | 0,977 | 1,17E-49 |
| GADD45B | 7,58E-54 | -0,7765 | 0,448 | 0,757 | 1,25E-49 |
| TUBA1A | 8,54E-54 | 0,631536 | 0,999 | 0,998 | 1,41E-49 |
| TOP1 | 9,25E-54 | -0,5462 | 0,943 | 1 | 1,53E-49 |
| SOX10 | 1,51E-53 | 0,662935 | 0,811 | 0,518 | 2,5E-49 |
| PMP2 | 2,29E-53 | 0,827748 | 0,963 | 0,868 | 3,79E-49 |
| FDFT1 | 2,41E-53 | 0,739421 | 0,873 | 0,682 | 4E-49 |
| ATF3 | 3,24E-53 | -0,70795 | 0,881 | 0,946 | 5,36E-49 |
| CDC42EP1 | 6,79E-53 | 0,613143 | 0,48 | 0,161 | 1,12E-48 |
| MAL | 2,16E-52 | 0,596508 | 0,988 | 0,948 | 3,57E-48 |
| AIF1L | 2,49E-52 | 0,606739 | 0,963 | 0,876 | 4,12E-48 |
| MAG | 4,8E-52 | 0,48704 | 0,999 | 0,998 | 7,95E-48 |
| SYNJ2 | 7,56E-52 | 0,714964 | 0,8 | 0,548 | 1,25E-47 |
| IFIT1 | 1,02E-51 | -0,92828 | 0,922 | 0,985 | 1,69E-47 |
| PSMA2 | 1,13E-51 | -0,55229 | 0,773 | 0,925 | 1,87E-47 |
| IL18BP | 1,55E-51 | -0,58992 | 0,796 | 0,951 | 2,56E-47 |
| GPRC5B | 1,88E-51 | 0,586273 | 0,958 | 0,86 | 3,12E-47 |
| PPP1R14A | 2,63E-51 | 0,871285 | 0,968 | 0,922 | 4,35E-47 |
| TNFRSF21 | 6,58E-51 | 0,749189 | 0,774 | 0,516 | 1,09E-46 |
| SSNA1 | 7,62E-51 | 0,605233 | 0,885 | 0,673 | 1,26E-46 |
| FBN1 | 9,02E-51 | -0,58423 | 0,156 | 0,438 | 1,49E-46 |
| MCF2L | 1,32E-50 | 0,633173 | 0,498 | 0,19 | 2,19E-46 |
| RGL1 | 2,78E-50 | -0,63933 | 0,613 | 0,823 | 4,6E-46 |
| AC011997.1 | 4E-50 | -0,63202 | 0,431 | 0,707 | 6,63E-46 |
| MT2A | 4,76E-50 | -0,56753 | 1 | 1 | 7,88E-46 |
| GAL3ST1 | 5,11E-50 | 0,526558 | 0,373 | 0,086 | 8,46E-46 |
| RARRES1 | 1,17E-49 | -0,56399 | 0,068 | 0,315 | 1,94E-45 |
| AATK | 1,36E-49 | 0,650852 | 0,726 | 0,429 | 2,25E-45 |
| TKT | 2,62E-49 | 0,605464 | 0,936 | 0,782 | 4,34E-45 |
| GPX1 | 3,83E-49 | -0,6608 | 0,726 | 0,871 | 6,34E-45 |
| TSC22D1 | 4,18E-49 | 0,799288 | 0,696 | 0,401 | 6,92E-45 |
| TNFRSF1A | 5,4E-49 | -0,51906 | 0,206 | 0,528 | 8,95E-45 |
| VIM | 6,44E-49 | -1,02281 | 0,829 | 0,949 | 1,07E-44 |
| HSPA2 | 7,12E-49 | 0,690979 | 0,748 | 0,482 | 1,18E-44 |
| PREX1 | 7,19E-49 | 0,662094 | 0,875 | 0,719 | 1,19E-44 |
| PDGFA | 9,17E-49 | 0,574483 | 0,458 | 0,151 | 1,52E-44 |
| C8orf4 | 1,53E-48 | -0,65656 | 0,063 | 0,303 | 2,53E-44 |
| PRIMA1 | 1,62E-48 | 0,461716 | 0,319 | 0,052 | 2,68E-44 |
| PHF11 | 1,68E-48 | -0,51831 | 0,827 | 0,948 | 2,78E-44 |
| MOBP | 2,49E-48 | 0,825027 | 0,899 | 0,737 | 4,13E-44 |
| HAS2 | 2,83E-48 | -0,60983 | 0,048 | 0,276 | 4,68E-44 |
| C4orf3 | 3,38E-48 | -0,50248 | 0,879 | 0,949 | 5,6E-44 |
| IDI1 | 4,73E-48 | 0,676741 | 0,901 | 0,768 | 7,83E-44 |
| SAP18 | 5,07E-48 | -0,49749 | 0,936 | 0,975 | 8,39E-44 |
| ANP32A | 5,67E-48 | 0,608977 | 0,715 | 0,436 | 9,4E-44 |
| CD68 | 7,08E-48 | -0,62504 | 0,192 | 0,499 | 1,17E-43 |
| DNAJA1 | 9,12E-48 | -0,51676 | 0,968 | 0,994 | 1,51E-43 |
| ARNTL2 | 1,12E-47 | -0,44005 | 0,062 | 0,31 | 1,86E-43 |
| FAM69C | 1,19E-47 | 0,635609 | 0,557 | 0,252 | 1,97E-43 |
| STXBP6 | 1,51E-47 | 0,639862 | 0,773 | 0,508 | 2,5E-43 |
| RAB1A | 1,82E-47 | -0,54734 | 0,759 | 0,885 | 3,01E-43 |
| FAM181B | 1,94E-47 | 0,652691 | 0,629 | 0,304 | 3,21E-43 |
| PSME1 | 1,95E-47 | -0,42326 | 0,992 | 1 | 3,24E-43 |
| NFKBIZ | 1,96E-47 | -0,63055 | 0,139 | 0,41 | 3,24E-43 |
| EEPD1 | 3,83E-47 | 0,547095 | 0,471 | 0,163 | 6,34E-43 |
| ODF2L | 4,57E-47 | -0,60687 | 0,617 | 0,802 | 7,57E-43 |
| ARHGEF37 | 6,77E-47 | 0,581152 | 0,558 | 0,257 | 1,12E-42 |
| CXADR | 7,23E-47 | 0,652193 | 0,555 | 0,241 | 1,2E-42 |
| APOL1 | 7,58E-47 | -0,74113 | 0,502 | 0,76 | 1,26E-42 |
| PLPP2 | 2,99E-46 | 0,600357 | 0,828 | 0,587 | 4,95E-42 |
| STX11 | 3,94E-46 | -0,40642 | 0,052 | 0,278 | 6,52E-42 |
| TNIP1 | 4,24E-46 | -0,54738 | 0,382 | 0,636 | 7,02E-42 |
| JAM2 | 6,14E-46 | -0,3634 | 0,043 | 0,26 | 1,02E-41 |
| LAPTM4A | 6,84E-46 | -0,49102 | 0,784 | 0,896 | 1,13E-41 |
| SYNE3 | 7,51E-46 | 0,461859 | 0,299 | 0,048 | 1,24E-41 |
| DYNLL1 | 1,09E-45 | 0,470857 | 1 | 0,991 | 1,8E-41 |
| TMEM151A | 8,64E-45 | 0,701978 | 0,799 | 0,594 | 1,43E-40 |
| AMER2 | 9,59E-45 | 0,805966 | 0,782 | 0,553 | 1,59E-40 |
| NTRK2 | 1,15E-44 | -0,61588 | 0,565 | 0,797 | 1,9E-40 |
| RHOB | 1,97E-44 | 0,794328 | 0,636 | 0,349 | 3,25E-40 |
| AGAP1 | 2,45E-44 | 0,609557 | 0,772 | 0,547 | 4,06E-40 |
| RYBP | 2,64E-44 | 0,654435 | 0,822 | 0,63 | 4,37E-40 |
| DGKH | 3,15E-44 | -0,63545 | 0,25 | 0,536 | 5,21E-40 |
| DRAM1 | 3,72E-44 | -0,48479 | 0,194 | 0,481 | 6,15E-40 |
| KLK10 | 5,49E-44 | -0,57172 | 0,269 | 0,567 | 9,09E-40 |
| CALM1 | 7,13E-44 | 0,375271 | 1 | 1 | 1,18E-39 |
| RHBDF2 | 8,79E-44 | -0,46895 | 0,149 | 0,436 | 1,46E-39 |
| DIXDC1 | 9,23E-44 | 0,557111 | 0,538 | 0,233 | 1,53E-39 |
| NUDT3 | 1,26E-43 | -0,48861 | 0,796 | 0,929 | 2,08E-39 |
| TMEM60 | 1,7E-43 | -0,59612 | 0,513 | 0,751 | 2,82E-39 |
| BHLHE41 | 4,18E-43 | 0,640073 | 0,534 | 0,246 | 6,93E-39 |
| C3orf38 | 7,2E-43 | -0,53528 | 0,485 | 0,707 | 1,19E-38 |
| TMCC3 | 7,27E-43 | 0,5277 | 0,474 | 0,181 | 1,2E-38 |
| PAQR8 | 7,79E-43 | 0,577591 | 0,943 | 0,88 | 1,29E-38 |
| MBP | 8,38E-43 | 0,704348 | 0,999 | 1 | 1,39E-38 |
| PLEKHH1 | 9,07E-43 | 0,639022 | 0,671 | 0,43 | 1,5E-38 |
| FAM46A | 1,52E-42 | -0,54109 | 0,336 | 0,633 | 2,51E-38 |
| SCN1B | 1,58E-42 | 0,590157 | 0,869 | 0,699 | 2,61E-38 |
| ANKH | 2,41E-42 | 0,610949 | 0,722 | 0,478 | 3,99E-38 |
| APOBEC3G | 2,95E-42 | -0,60419 | 0,442 | 0,694 | 4,89E-38 |
| SEPP1 | 3,62E-42 | 0,661559 | 0,983 | 0,962 | 5,99E-38 |
| SLC45A3 | 4,73E-42 | 0,512311 | 0,443 | 0,161 | 7,84E-38 |
| NFIA | 5,33E-42 | 0,607003 | 0,651 | 0,37 | 8,82E-38 |
| CHD1 | 6,44E-42 | -0,54353 | 0,393 | 0,651 | 1,07E-37 |
| HTRA1 | 7,9E-42 | 0,524715 | 0,973 | 0,906 | 1,31E-37 |
| RP11-79H23.3 | 9,24E-42 | -0,30089 | 0,018 | 0,19 | 1,53E-37 |
| FGFR1 | 9,87E-42 | -0,55925 | 0,262 | 0,513 | 1,63E-37 |
| MID1IP1 | 1,08E-41 | 0,580123 | 0,807 | 0,599 | 1,79E-37 |
| ADGRG1 | 1,18E-41 | 0,631067 | 0,759 | 0,502 | 1,95E-37 |
| RRAS | 1,22E-41 | -0,4978 | 0,218 | 0,499 | 2,02E-37 |
| SECTM1 | 1,23E-41 | -0,53709 | 0,082 | 0,316 | 2,04E-37 |
| NFIX | 1,48E-41 | 0,570286 | 0,874 | 0,722 | 2,45E-37 |
| PDE1A | 1,67E-41 | 0,514603 | 0,48 | 0,189 | 2,76E-37 |
| ARID5A | 1,84E-41 | -0,53796 | 0,214 | 0,492 | 3,04E-37 |
| TMCC2 | 1,88E-41 | 0,425181 | 0,309 | 0,065 | 3,12E-37 |
| SLC6A8 | 2,87E-41 | 0,554429 | 0,723 | 0,469 | 4,75E-37 |
| DACT3 | 2,98E-41 | 0,514295 | 0,486 | 0,195 | 4,93E-37 |
| YWHAQ | 3,92E-41 | 0,44752 | 1 | 1 | 6,48E-37 |
| MAP3K8 | 4,54E-41 | -0,34777 | 0,04 | 0,238 | 7,51E-37 |
| FBXL7 | 7,92E-41 | -0,51812 | 0,478 | 0,713 | 1,31E-36 |
| TMC6 | 9,53E-41 | 0,582295 | 0,669 | 0,418 | 1,58E-36 |
| S1PR5 | 1,48E-40 | 0,470811 | 0,406 | 0,132 | 2,45E-36 |
| TTC3 | 1,64E-40 | 0,563096 | 0,855 | 0,664 | 2,72E-36 |
| LRRC8D | 2,01E-40 | 0,542429 | 0,525 | 0,249 | 3,33E-36 |
| CPM | 2,07E-40 | 0,542585 | 0,451 | 0,184 | 3,43E-36 |
| ATP5I | 2,36E-40 | 0,459957 | 0,997 | 0,991 | 3,91E-36 |
| CFL2 | 2,62E-40 | 0,494479 | 0,941 | 0,834 | 4,34E-36 |
| KCNQ1OT1 | 3,5E-40 | 0,852334 | 0,68 | 0,407 | 5,79E-36 |
| PADI2 | 3,87E-40 | 0,602177 | 0,837 | 0,68 | 6,41E-36 |
| F3 | 9,03E-40 | -0,42971 | 0,099 | 0,335 | 1,5E-35 |
| ARL5B | 9,15E-40 | -0,56899 | 0,429 | 0,68 | 1,52E-35 |
| GNG11 | 9,35E-40 | -0,47024 | 0,141 | 0,39 | 1,55E-35 |
| HCN2 | 1,1E-39 | 0,794457 | 0,806 | 0,664 | 1,82E-35 |
| PVRL1 | 1,19E-39 | 0,544954 | 0,601 | 0,338 | 1,97E-35 |
| SFRP1 | 1,44E-39 | -0,72977 | 0,565 | 0,791 | 2,38E-35 |
| ARL6IP5 | 2,03E-39 | 0,44778 | 0,978 | 0,916 | 3,36E-35 |
| BIN1 | 2,7E-39 | 0,54298 | 0,838 | 0,668 | 4,46E-35 |
| C1S | 3,08E-39 | -0,38849 | 0,032 | 0,217 | 5,11E-35 |
| S100B | 3,48E-39 | 0,48787 | 0,999 | 1 | 5,76E-35 |
| ACTR6 | 6,25E-39 | -0,47779 | 0,647 | 0,833 | 1,03E-34 |
| TAP2 | 7,74E-39 | -0,46882 | 0,84 | 0,954 | 1,28E-34 |
| SHTN1 | 8,25E-39 | 0,509955 | 0,906 | 0,799 | 1,37E-34 |
| HSP90AA1 | 8,81E-39 | -0,30181 | 1 | 1 | 1,46E-34 |
| FNTA | 1,13E-38 | -0,43641 | 0,955 | 0,989 | 1,87E-34 |
| SH3BP5 | 1,42E-38 | 0,710186 | 0,773 | 0,545 | 2,35E-34 |
| NINJ1 | 1,83E-38 | -0,54379 | 0,232 | 0,464 | 3,03E-34 |
| CLDND1 | 1,95E-38 | 0,579667 | 0,983 | 0,957 | 3,22E-34 |
| CUEDC1 | 2,1E-38 | 0,505468 | 0,572 | 0,307 | 3,48E-34 |
| EPB41L1 | 2,52E-38 | 0,535014 | 0,478 | 0,214 | 4,16E-34 |
| TRAFD1 | 3,76E-38 | -0,4872 | 0,666 | 0,86 | 6,22E-34 |
| PRKCSH | 4,02E-38 | 0,509038 | 0,911 | 0,788 | 6,65E-34 |
| PPP2R2B | 4,53E-38 | 0,539402 | 0,864 | 0,674 | 7,5E-34 |
| SH3D19 | 7,08E-38 | 0,52135 | 0,553 | 0,273 | 1,17E-33 |
| PACS2 | 1,02E-37 | 0,503217 | 0,986 | 0,96 | 1,68E-33 |
| AMD1 | 1,21E-37 | 0,653117 | 0,824 | 0,627 | 2E-33 |
| SMIM5 | 1,29E-37 | 0,330198 | 0,2 | 0,015 | 2,13E-33 |
| UBB | 1,91E-37 | -0,3668 | 0,999 | 1 | 3,16E-33 |
| DPYSL5 | 1,93E-37 | 0,57631 | 0,507 | 0,253 | 3,19E-33 |
| PCBP4 | 2,32E-37 | 0,527374 | 0,725 | 0,453 | 3,84E-33 |
| GPR37 | 2,46E-37 | 0,586426 | 0,826 | 0,654 | 4,08E-33 |
| ATP1B1 | 3,19E-37 | 0,421641 | 0,999 | 0,997 | 5,28E-33 |
| HERC5 | 3,23E-37 | -0,66659 | 0,68 | 0,853 | 5,35E-33 |
| SLC44A1 | 3,28E-37 | 0,406205 | 1 | 1 | 5,42E-33 |
| DPY19L1 | 4,81E-37 | 0,634077 | 0,611 | 0,39 | 7,96E-33 |
| NFE2L2 | 5,51E-37 | -0,39257 | 0,985 | 0,994 | 9,12E-33 |
| CLIC4 | 6,53E-37 | -0,4601 | 0,928 | 0,98 | 1,08E-32 |
| IPO13 | 9,84E-37 | 0,544851 | 0,664 | 0,432 | 1,63E-32 |
| GMPR | 1,04E-36 | -0,53611 | 0,231 | 0,479 | 1,72E-32 |
| VAMP2 | 1,76E-36 | 0,436209 | 0,983 | 0,966 | 2,91E-32 |
| SSTR2 | 1,94E-36 | -0,42318 | 0,03 | 0,2 | 3,2E-32 |
| ACTR3 | 2,14E-36 | -0,47098 | 0,905 | 0,943 | 3,55E-32 |
| MEGF9 | 3,85E-36 | 0,530829 | 0,667 | 0,418 | 6,37E-32 |
| TAX1BP1 | 4,86E-36 | -0,4421 | 0,953 | 0,983 | 8,05E-32 |
| GADD45G | 5,25E-36 | -0,86788 | 0,365 | 0,555 | 8,7E-32 |
| SLC12A2 | 6,06E-36 | 0,608961 | 0,926 | 0,846 | 1E-31 |
| TMEM50A | 1,24E-35 | -0,41547 | 0,91 | 0,957 | 2,05E-31 |
| EPB41L3 | 1,3E-35 | 0,523519 | 0,505 | 0,269 | 2,16E-31 |
| TYRO3 | 1,96E-35 | 0,546783 | 0,615 | 0,392 | 3,24E-31 |
| MCL1 | 2,21E-35 | -0,5035 | 0,735 | 0,896 | 3,66E-31 |
| PIK3C2B | 4,36E-35 | 0,460272 | 0,406 | 0,16 | 7,22E-31 |
| XAF1 | 4,55E-35 | -0,59058 | 0,498 | 0,757 | 7,53E-31 |
| GNAQ | 5,08E-35 | 0,533997 | 0,816 | 0,622 | 8,41E-31 |
| FBXO32 | 6,57E-35 | 0,621693 | 0,621 | 0,375 | 1,09E-30 |
| TMEM125 | 6,67E-35 | 0,511211 | 0,377 | 0,129 | 1,1E-30 |
| C6orf1 | 8,23E-35 | -0,38293 | 0,989 | 1 | 1,36E-30 |
| CD46 | 8,57E-35 | -0,49219 | 0,788 | 0,914 | 1,42E-30 |
| CPEB4 | 1,05E-34 | 0,556194 | 0,731 | 0,505 | 1,73E-30 |
| KIAA0232 | 1,23E-34 | 0,552839 | 0,605 | 0,364 | 2,04E-30 |
| ANTXR2 | 1,51E-34 | -0,5139 | 0,281 | 0,531 | 2,5E-30 |
| CA14 | 1,55E-34 | 0,420618 | 0,325 | 0,103 | 2,57E-30 |
| CCDC109B | 1,58E-34 | -0,44212 | 0,813 | 0,932 | 2,62E-30 |
| GPR37L1 | 2,17E-34 | 0,576795 | 0,516 | 0,253 | 3,59E-30 |
| NUAK1 | 2,42E-34 | 0,365314 | 0,251 | 0,049 | 4E-30 |
| TMC7 | 2,85E-34 | 0,432441 | 0,389 | 0,149 | 4,72E-30 |
| HHIP | 2,95E-34 | 0,557053 | 0,571 | 0,303 | 4,88E-30 |
| PSMB8 | 4,01E-34 | -0,35724 | 0,987 | 1 | 6,64E-30 |
| B3GAT1 | 4,51E-34 | 0,533462 | 0,813 | 0,653 | 7,46E-30 |
| ECHDC1 | 4,9E-34 | -0,47723 | 0,779 | 0,917 | 8,11E-30 |
| AK5 | 7,92E-34 | 0,489414 | 0,595 | 0,329 | 1,31E-29 |
| DDX60L | 1,33E-33 | -0,55496 | 0,65 | 0,826 | 2,2E-29 |
| GNAZ | 1,86E-33 | 0,44691 | 0,453 | 0,204 | 3,09E-29 |
| SALL1 | 2,08E-33 | 0,491635 | 0,48 | 0,235 | 3,45E-29 |
| OSBPL1A | 2,26E-33 | 0,452732 | 0,957 | 0,899 | 3,75E-29 |
| TRIM25 | 2,59E-33 | -0,51484 | 0,598 | 0,791 | 4,29E-29 |
| DHCR24 | 3,87E-33 | 0,62324 | 0,955 | 0,912 | 6,4E-29 |
| C5orf56 | 4E-33 | -0,41115 | 0,93 | 0,982 | 6,62E-29 |
| PAQR4 | 5,45E-33 | 0,508644 | 0,737 | 0,522 | 9,03E-29 |
| RALA | 5,98E-33 | -0,52247 | 0,8 | 0,883 | 9,91E-29 |
| PTMA | 7,67E-33 | 0,403718 | 1 | 1 | 1,27E-28 |
| ARF3 | 8,83E-33 | -0,43862 | 0,671 | 0,831 | 1,46E-28 |
| HNRNPF | 1,21E-32 | -0,41575 | 0,892 | 0,94 | 2E-28 |
| COBL | 1,53E-32 | 0,457444 | 0,859 | 0,664 | 2,53E-28 |
| SYNDIG1 | 2,27E-32 | 0,501401 | 0,682 | 0,447 | 3,76E-28 |
| SAMD9 | 2,76E-32 | -0,54779 | 0,654 | 0,828 | 4,58E-28 |
| TXN | 2,92E-32 | -0,45129 | 0,996 | 0,997 | 4,84E-28 |
| CREM | 2,93E-32 | -0,49658 | 0,466 | 0,694 | 4,85E-28 |
| CNP | 2,94E-32 | 0,640259 | 0,998 | 1 | 4,86E-28 |
| ST18 | 3,34E-32 | 0,527179 | 0,542 | 0,306 | 5,53E-28 |
| C7orf50 | 3,45E-32 | 0,49328 | 0,797 | 0,596 | 5,72E-28 |
| PLA2G16 | 3,52E-32 | 0,380519 | 0,999 | 0,997 | 5,83E-28 |
| DUSP7 | 3,65E-32 | 0,420793 | 0,337 | 0,108 | 6,05E-28 |
| SYNE2 | 4,9E-32 | -0,5826 | 0,152 | 0,349 | 8,12E-28 |
| HIVEP2 | 5,42E-32 | -0,40321 | 0,208 | 0,45 | 8,98E-28 |
| PTP4A2 | 6,38E-32 | 0,412886 | 0,979 | 0,945 | 1,06E-27 |
| CDC42SE1 | 6,44E-32 | -0,45837 | 0,827 | 0,922 | 1,07E-27 |
| RBP7 | 6,65E-32 | 0,541769 | 0,773 | 0,556 | 1,1E-27 |
| XRN1 | 6,87E-32 | -0,50467 | 0,653 | 0,796 | 1,14E-27 |
| ARF6 | 7,88E-32 | -0,41283 | 0,767 | 0,903 | 1,3E-27 |
| TMOD2 | 9,09E-32 | 0,495992 | 0,684 | 0,45 | 1,5E-27 |
| VRK2 | 9,88E-32 | -0,47815 | 0,623 | 0,765 | 1,64E-27 |
| CARNS1 | 1,15E-31 | 0,584564 | 0,819 | 0,687 | 1,9E-27 |
| IFNG | 1,15E-31 | -0,3335 | 0,402 | 0,679 | 1,9E-27 |
| UBE2D3 | 1,85E-31 | -0,38119 | 0,939 | 0,975 | 3,06E-27 |
| GLS | 1,86E-31 | -0,4754 | 0,481 | 0,674 | 3,07E-27 |
| ZFHX4 | 2,14E-31 | -0,49416 | 0,684 | 0,829 | 3,55E-27 |
| SOX8 | 2,43E-31 | 0,4871 | 0,404 | 0,171 | 4,03E-27 |
| HELZ2 | 3,28E-31 | -0,44559 | 0,642 | 0,822 | 5,44E-27 |
| MGLL | 4,27E-31 | -0,61404 | 0,509 | 0,69 | 7,07E-27 |
| CLASP2 | 4,59E-31 | 0,478874 | 0,681 | 0,499 | 7,61E-27 |
| BRF1 | 4,75E-31 | 0,504547 | 0,409 | 0,189 | 7,87E-27 |
| CLN8 | 4,86E-31 | 0,433422 | 0,431 | 0,198 | 8,05E-27 |
| YBX3 | 5,15E-31 | -0,59497 | 0,102 | 0,298 | 8,52E-27 |
| SQLE | 6,4E-31 | 0,651474 | 0,825 | 0,624 | 1,06E-26 |
| ERBB3 | 9,24E-31 | 0,398314 | 0,277 | 0,074 | 1,53E-26 |
| CTNNBL1 | 9,39E-31 | -0,4303 | 0,407 | 0,633 | 1,56E-26 |
| NFKBIE | 9,79E-31 | -0,33888 | 0,103 | 0,303 | 1,62E-26 |
| DUSP5 | 1,13E-30 | -0,37119 | 0,126 | 0,346 | 1,87E-26 |
| IL32 | 1,42E-30 | -0,69052 | 0,787 | 0,811 | 2,34E-26 |
| PCSK6 | 1,44E-30 | 0,602786 | 0,766 | 0,608 | 2,38E-26 |
| CHMP5 | 1,69E-30 | -0,37412 | 0,944 | 0,983 | 2,8E-26 |
| PSMB10 | 1,99E-30 | -0,39329 | 0,792 | 0,934 | 3,29E-26 |
| PLCH2 | 2,05E-30 | 0,480792 | 0,416 | 0,2 | 3,4E-26 |
| RSU1 | 2,07E-30 | -0,43923 | 0,689 | 0,808 | 3,43E-26 |
| PTPN2 | 2,23E-30 | -0,42747 | 0,345 | 0,567 | 3,69E-26 |
| HARS | 2,29E-30 | -0,43989 | 0,416 | 0,614 | 3,79E-26 |
| PSMA4 | 2,4E-30 | -0,38898 | 0,99 | 0,997 | 3,97E-26 |
| GBP7 | 2,79E-30 | -0,29657 | 0,048 | 0,215 | 4,62E-26 |
| LMO2 | 3,16E-30 | -0,37563 | 0,095 | 0,28 | 5,24E-26 |
| TUBB2B | 3,72E-30 | 0,925459 | 0,646 | 0,447 | 6,16E-26 |
| SCML1 | 3,88E-30 | -0,44951 | 0,325 | 0,555 | 6,42E-26 |
| SPECC1 | 4,16E-30 | 0,466196 | 0,704 | 0,484 | 6,89E-26 |
| SPPL2A | 4,3E-30 | -0,43665 | 0,615 | 0,776 | 7,12E-26 |
| LHFP | 4,49E-30 | -0,46619 | 0,576 | 0,762 | 7,43E-26 |
| INPP5F | 4,8E-30 | 0,47735 | 0,484 | 0,243 | 7,95E-26 |
| ARL8A | 5,02E-30 | 0,432195 | 0,922 | 0,846 | 8,3E-26 |
| ATOX1 | 7,74E-30 | -0,33833 | 0,996 | 1 | 1,28E-25 |
| PSAT1 | 8,48E-30 | 0,497755 | 0,95 | 0,891 | 1,4E-25 |
| NACC2 | 1,12E-29 | 0,448724 | 0,509 | 0,283 | 1,85E-25 |
| PRR18 | 1,17E-29 | 0,376964 | 0,265 | 0,068 | 1,94E-25 |
| PFN2 | 1,52E-29 | 0,47761 | 0,809 | 0,608 | 2,51E-25 |
| SOCS1 | 1,69E-29 | -0,51003 | 0,618 | 0,799 | 2,8E-25 |
| MBNL2 | 1,73E-29 | 0,449008 | 0,924 | 0,811 | 2,86E-25 |
| SOCS3 | 1,85E-29 | -0,50486 | 0,458 | 0,679 | 3,07E-25 |
| SLC24A2 | 2,06E-29 | 0,524357 | 0,92 | 0,799 | 3,41E-25 |
| CALCOCO2 | 2,29E-29 | -0,40754 | 0,908 | 0,954 | 3,79E-25 |
| RALGDS | 2,37E-29 | 0,49709 | 0,771 | 0,602 | 3,93E-25 |
| SDCBP | 2,57E-29 | -0,45098 | 0,604 | 0,751 | 4,26E-25 |
| FLOT1 | 2,89E-29 | -0,39738 | 0,833 | 0,911 | 4,78E-25 |
| FOXO1 | 3,19E-29 | 0,444318 | 0,565 | 0,323 | 5,27E-25 |
| DMTN | 5,2E-29 | 0,348474 | 0,269 | 0,072 | 8,61E-25 |
| MET | 5,36E-29 | -0,4287 | 0,186 | 0,404 | 8,87E-25 |
| EIF5A | 6,02E-29 | -0,36042 | 0,846 | 0,946 | 9,97E-25 |
| ADAP1 | 7,76E-29 | 0,47448 | 0,549 | 0,318 | 1,28E-24 |
| P2RX7 | 7,95E-29 | -0,50507 | 0,851 | 0,925 | 1,32E-24 |
| ALPK1 | 9,37E-29 | -0,34244 | 0,109 | 0,318 | 1,55E-24 |
| CD9 | 1,01E-28 | 0,333842 | 1 | 1 | 1,68E-24 |
| GJC2 | 1,16E-28 | 0,359813 | 0,239 | 0,054 | 1,92E-24 |
| SVIP | 1,52E-28 | 0,496742 | 0,849 | 0,697 | 2,52E-24 |
| RPL6 | 1,56E-28 | 0,404954 | 0,998 | 0,995 | 2,58E-24 |
| PLEKHA1 | 1,6E-28 | 0,525456 | 0,647 | 0,445 | 2,65E-24 |
| TPD52 | 1,86E-28 | 0,40574 | 0,661 | 0,427 | 3,08E-24 |
| ANXA5 | 2,07E-28 | -0,33067 | 0,974 | 0,997 | 3,43E-24 |
| CCNE2 | 2,1E-28 | 0,330934 | 0,203 | 0,035 | 3,48E-24 |
| GAMT | 4,09E-28 | 0,481001 | 0,887 | 0,77 | 6,78E-24 |
| INF2 | 4,14E-28 | 0,454331 | 0,754 | 0,625 | 6,85E-24 |
| STOM | 5,03E-28 | -0,44394 | 0,378 | 0,567 | 8,33E-24 |
| VLDLR | 5,23E-28 | 0,466038 | 0,522 | 0,304 | 8,67E-24 |
| LRRFIP2 | 5,47E-28 | 0,486928 | 0,735 | 0,524 | 9,05E-24 |
| PELI1 | 5,67E-28 | -0,39382 | 0,241 | 0,47 | 9,38E-24 |
| GIPC1 | 6,99E-28 | 0,455117 | 0,759 | 0,568 | 1,16E-23 |
| PHLDB1 | 7,5E-28 | 0,494473 | 0,604 | 0,398 | 1,24E-23 |
| EIF1 | 8,29E-28 | 0,298423 | 1 | 1 | 1,37E-23 |
| LPGAT1 | 9,67E-28 | -0,4517 | 0,796 | 0,877 | 1,6E-23 |
| SECISBP2L | 9,77E-28 | 0,52412 | 0,903 | 0,802 | 1,62E-23 |
| CMPK2 | 1,01E-27 | -0,36831 | 0,834 | 0,959 | 1,68E-23 |
| FAS | 1,34E-27 | -0,43643 | 0,358 | 0,575 | 2,22E-23 |
| CXCL2 | 1,45E-27 | -0,53123 | 0,019 | 0,147 | 2,4E-23 |
| CDV3 | 1,48E-27 | -0,39102 | 0,907 | 0,94 | 2,45E-23 |
| MRPL32 | 1,6E-27 | -0,41368 | 0,564 | 0,742 | 2,65E-23 |
| IRF2BP2 | 1,72E-27 | 0,445414 | 0,771 | 0,587 | 2,85E-23 |
| DBI | 1,92E-27 | -0,36461 | 0,992 | 0,998 | 3,18E-23 |
| PHACTR3 | 2,08E-27 | 0,488596 | 0,553 | 0,353 | 3,44E-23 |
| FN3K | 2,1E-27 | 0,35835 | 0,374 | 0,151 | 3,48E-23 |
| KCNK10 | 2,16E-27 | 0,508559 | 0,487 | 0,255 | 3,58E-23 |
| RAB31 | 2,27E-27 | -0,4502 | 0,664 | 0,833 | 3,76E-23 |
| STOX2 | 2,37E-27 | 0,360693 | 0,323 | 0,111 | 3,92E-23 |
| A1BG | 2,72E-27 | 0,440481 | 0,634 | 0,412 | 4,51E-23 |
| GALNT15 | 3E-27 | 0,457992 | 0,406 | 0,195 | 4,98E-23 |
| LGI3 | 3,02E-27 | 0,472452 | 0,84 | 0,72 | 5E-23 |
| PTRH1 | 3,2E-27 | -0,38145 | 0,352 | 0,585 | 5,3E-23 |
| SP100 | 3,43E-27 | -0,40238 | 0,833 | 0,939 | 5,68E-23 |
| NGFR | 3,54E-27 | 0,582425 | 0,689 | 0,507 | 5,86E-23 |
| IFITM2 | 3,69E-27 | -0,53699 | 0,156 | 0,312 | 6,11E-23 |
| MTDH | 3,95E-27 | 0,35105 | 0,992 | 0,978 | 6,53E-23 |
| GTF2I | 4,06E-27 | 0,444703 | 0,81 | 0,69 | 6,72E-23 |
| NFE2L3 | 4,22E-27 | -0,37911 | 0,256 | 0,504 | 6,99E-23 |
| VMP1 | 5,68E-27 | -0,42573 | 0,892 | 0,966 | 9,41E-23 |
| THBS2 | 5,8E-27 | 0,473582 | 0,481 | 0,272 | 9,61E-23 |
| LARP6 | 6,75E-27 | 0,451579 | 0,879 | 0,727 | 1,12E-22 |
| CARD19 | 6,82E-27 | 0,423225 | 0,51 | 0,304 | 1,13E-22 |
| GCSH | 8,54E-27 | 0,406095 | 0,877 | 0,725 | 1,41E-22 |
| RBM8A | 9,06E-27 | -0,38735 | 0,828 | 0,899 | 1,5E-22 |
| C4orf32 | 9,16E-27 | -0,41982 | 0,31 | 0,524 | 1,52E-22 |
| RGS16 | 9,18E-27 | -0,80412 | 0,549 | 0,702 | 1,52E-22 |
| ARHGEF7 | 9,82E-27 | 0,410559 | 0,496 | 0,289 | 1,63E-22 |
| PLEKHH2 | 1,01E-26 | -0,43917 | 0,29 | 0,519 | 1,67E-22 |
| BAMBI | 1,13E-26 | 0,421458 | 0,377 | 0,151 | 1,87E-22 |
| TTYH2 | 1,44E-26 | 0,464806 | 0,736 | 0,558 | 2,38E-22 |
| MORC3 | 1,52E-26 | -0,43957 | 0,593 | 0,745 | 2,52E-22 |
| DSCAML1 | 2,09E-26 | 0,352107 | 0,303 | 0,103 | 3,47E-22 |
| GSN | 2,31E-26 | 0,430541 | 1 | 1 | 3,83E-22 |
| GALNT7 | 2,34E-26 | 0,426398 | 0,651 | 0,424 | 3,87E-22 |
| KLHL32 | 2,71E-26 | 0,462706 | 0,473 | 0,269 | 4,49E-22 |
| TMEM63B | 2,88E-26 | -0,40602 | 0,526 | 0,725 | 4,76E-22 |
| BCL3 | 3,2E-26 | -0,33207 | 0,126 | 0,326 | 5,3E-22 |
| N4BP1 | 4,35E-26 | -0,42724 | 0,574 | 0,727 | 7,2E-22 |
| CYP27A1 | 4,64E-26 | 0,429079 | 0,659 | 0,488 | 7,69E-22 |
| TFEB | 4,67E-26 | 0,393879 | 0,405 | 0,201 | 7,73E-22 |
| CLCN4 | 5,13E-26 | 0,413915 | 0,499 | 0,283 | 8,49E-22 |
| ABCA1 | 5,31E-26 | -0,43653 | 0,086 | 0,269 | 8,79E-22 |
| AFMID | 5,76E-26 | 0,30832 | 0,258 | 0,072 | 9,53E-22 |
| SEPT7 | 8,26E-26 | 0,348152 | 0,999 | 1 | 1,37E-21 |
| UNC93B1 | 9,06E-26 | -0,40868 | 0,279 | 0,504 | 1,5E-21 |
| MX2 | 1,22E-25 | -0,45014 | 0,116 | 0,306 | 2,02E-21 |
| TIMP2 | 1,27E-25 | 0,401986 | 0,994 | 0,994 | 2,1E-21 |
| NOC3L | 1,34E-25 | -0,46787 | 0,454 | 0,637 | 2,22E-21 |
| USP18 | 1,73E-25 | -0,45523 | 0,418 | 0,636 | 2,86E-21 |
| TPM3 | 1,83E-25 | -0,37421 | 0,894 | 0,934 | 3,02E-21 |
| NUPR1 | 1,86E-25 | -1,18403 | 0,43 | 0,584 | 3,07E-21 |
| LDLRAP1 | 1,92E-25 | 0,364428 | 0,347 | 0,152 | 3,18E-21 |
| KLF13 | 1,94E-25 | 0,401318 | 0,807 | 0,602 | 3,2E-21 |
| SAMHD1 | 1,99E-25 | -0,42671 | 0,456 | 0,67 | 3,29E-21 |
| CCSER2 | 2,06E-25 | -0,3866 | 0,708 | 0,816 | 3,41E-21 |
| MSMO1 | 2,18E-25 | 0,569555 | 0,941 | 0,911 | 3,61E-21 |
| FOSL2 | 2,19E-25 | -0,39305 | 0,207 | 0,412 | 3,63E-21 |
| RTP4 | 2,36E-25 | -0,40888 | 0,726 | 0,86 | 3,91E-21 |
| OLIG1 | 2,97E-25 | 0,281855 | 0,16 | 0,02 | 4,92E-21 |
| FAM111A | 3,29E-25 | -0,40878 | 0,56 | 0,768 | 5,46E-21 |
| RAB11FIP4 | 3,31E-25 | 0,344835 | 0,343 | 0,134 | 5,49E-21 |
| LRRN3 | 3,39E-25 | -0,27305 | 0,031 | 0,166 | 5,61E-21 |
| CD44 | 3,45E-25 | -0,55516 | 0,841 | 0,929 | 5,7E-21 |
| DICER1 | 3,66E-25 | -0,50594 | 0,932 | 0,975 | 6,05E-21 |
| LHPP | 4,83E-25 | 0,462123 | 0,877 | 0,779 | 8E-21 |
| SLC23A2 | 5,26E-25 | 0,454763 | 0,69 | 0,49 | 8,72E-21 |
| BRSK1 | 7,02E-25 | 0,364569 | 0,403 | 0,192 | 1,16E-20 |
| CHN2 | 8,36E-25 | 0,443043 | 0,577 | 0,372 | 1,38E-20 |
| NRIP1 | 8,43E-25 | -0,41854 | 0,429 | 0,618 | 1,4E-20 |
| SERINC5 | 9,66E-25 | 0,480365 | 0,874 | 0,731 | 1,6E-20 |
| NOL4L | 1,16E-24 | 0,266929 | 0,197 | 0,04 | 1,92E-20 |
| TMEM63A | 1,39E-24 | 0,447183 | 0,762 | 0,61 | 2,31E-20 |
| EPN2 | 1,53E-24 | 0,435308 | 0,844 | 0,725 | 2,54E-20 |
| OMG | 1,55E-24 | 0,429772 | 0,434 | 0,246 | 2,57E-20 |
| ELMO2 | 1,72E-24 | -0,3675 | 0,3 | 0,521 | 2,86E-20 |
| SNCA | 1,76E-24 | 0,421538 | 0,757 | 0,571 | 2,92E-20 |
| HERC6 | 2E-24 | -0,44022 | 0,535 | 0,733 | 3,31E-20 |
| ELOVL7 | 2,07E-24 | -0,35465 | 0,25 | 0,452 | 3,43E-20 |
| MACF1 | 2,23E-24 | -0,44612 | 0,9 | 0,949 | 3,69E-20 |
| SCO2 | 2,53E-24 | -0,36906 | 0,256 | 0,425 | 4,19E-20 |
| C4orf48 | 3E-24 | 0,370908 | 0,997 | 0,998 | 4,97E-20 |
| ARHGAP22 | 3,97E-24 | 0,282396 | 0,227 | 0,057 | 6,57E-20 |
| CDK19 | 3,98E-24 | 0,396606 | 0,549 | 0,329 | 6,59E-20 |
| DOCK5 | 3,99E-24 | 0,40648 | 0,561 | 0,372 | 6,6E-20 |
| AGPAT4 | 4,05E-24 | 0,382396 | 0,395 | 0,201 | 6,7E-20 |
| ASPHD2 | 4,13E-24 | -0,37145 | 0,226 | 0,427 | 6,84E-20 |
| UBE2L6 | 4,18E-24 | -0,30759 | 0,959 | 0,992 | 6,92E-20 |
| NCOR2 | 4,54E-24 | 0,383488 | 0,497 | 0,28 | 7,52E-20 |
| MT1M | 4,7E-24 | -0,51356 | 0,155 | 0,344 | 7,79E-20 |
| SCPEP1 | 5,42E-24 | -0,38807 | 0,731 | 0,849 | 8,98E-20 |
| NUB1 | 5,5E-24 | -0,35382 | 0,904 | 0,962 | 9,11E-20 |
| HES6 | 5,59E-24 | 0,509637 | 0,606 | 0,416 | 9,25E-20 |
| RRAGC | 5,89E-24 | -0,37339 | 0,685 | 0,843 | 9,75E-20 |
| SAR1A | 5,95E-24 | -0,37319 | 0,709 | 0,828 | 9,85E-20 |
| MMP25-AS1 | 5,98E-24 | -0,3382 | 0,192 | 0,407 | 9,9E-20 |
| FIS1 | 5,99E-24 | 0,326194 | 0,994 | 0,985 | 9,92E-20 |
| ROGDI | 5,99E-24 | 0,433577 | 0,678 | 0,501 | 9,92E-20 |
| MOG | 6,15E-24 | 0,45926 | 0,961 | 0,955 | 1,02E-19 |
| XYLT1 | 6,29E-24 | 0,370612 | 0,308 | 0,117 | 1,04E-19 |
| USP30-AS1 | 8,36E-24 | -0,40935 | 0,303 | 0,527 | 1,38E-19 |
| SLCO4A1-AS1 | 8,55E-24 | 0,358856 | 0,27 | 0,095 | 1,41E-19 |
| FAM13C | 9,72E-24 | 0,435415 | 0,555 | 0,338 | 1,61E-19 |
| DECR1 | 1,02E-23 | -0,36973 | 0,671 | 0,819 | 1,69E-19 |
| TMIGD3 | 1,11E-23 | 0,294134 | 0,173 | 0,029 | 1,84E-19 |
| SELK | 1,13E-23 | -0,3593 | 0,815 | 0,92 | 1,88E-19 |
| SLC7A14 | 1,29E-23 | 0,446573 | 0,425 | 0,217 | 2,14E-19 |
| IFIT5 | 1,55E-23 | -0,36159 | 0,793 | 0,922 | 2,57E-19 |
| MAP7D1 | 1,63E-23 | 0,415776 | 0,802 | 0,67 | 2,7E-19 |
| RP11-138A9.2 | 1,73E-23 | -0,36571 | 0,222 | 0,424 | 2,86E-19 |
| FGFR1OP2 | 1,89E-23 | 0,400075 | 0,711 | 0,558 | 3,13E-19 |
| TRIM26 | 2,71E-23 | -0,36949 | 0,48 | 0,674 | 4,48E-19 |
| PLPP3 | 2,84E-23 | 0,468448 | 0,736 | 0,553 | 4,7E-19 |
| MAPT | 2,95E-23 | 0,393649 | 0,72 | 0,527 | 4,88E-19 |
| MFAP3L | 3,13E-23 | 0,327662 | 0,368 | 0,158 | 5,18E-19 |
| APBB1 | 3,24E-23 | 0,415746 | 0,66 | 0,53 | 5,36E-19 |
| TEX2 | 3,44E-23 | 0,358255 | 0,432 | 0,229 | 5,7E-19 |
| IFI16 | 3,58E-23 | -0,32235 | 0,978 | 0,998 | 5,93E-19 |
| PIM3 | 3,68E-23 | -0,41954 | 0,847 | 0,911 | 6,09E-19 |
| UBTD1 | 3,73E-23 | 0,300568 | 0,284 | 0,103 | 6,18E-19 |
| RPL23 | 3,82E-23 | 0,401291 | 0,997 | 0,985 | 6,32E-19 |
| ANGPTL2 | 5,78E-23 | 0,443266 | 0,526 | 0,335 | 9,57E-19 |
| CNDP1 | 6,04E-23 | 0,595861 | 0,993 | 0,995 | 1E-18 |
| NHLRC3 | 6,2E-23 | -0,40998 | 0,558 | 0,731 | 1,03E-18 |
| PPM1K | 6,25E-23 | -0,41123 | 0,804 | 0,909 | 1,04E-18 |
| RUNX2 | 6,58E-23 | 0,356792 | 0,376 | 0,177 | 1,09E-18 |
| B4GALT5 | 6,99E-23 | -0,40527 | 0,642 | 0,75 | 1,16E-18 |
| METRN | 7E-23 | 0,463597 | 0,868 | 0,727 | 1,16E-18 |
| FJX1 | 7,9E-23 | -0,60795 | 0,198 | 0,39 | 1,31E-18 |
| MYO10 | 8,46E-23 | 0,381694 | 0,399 | 0,209 | 1,4E-18 |
| SEMA4C | 9,68E-23 | 0,332295 | 0,307 | 0,12 | 1,6E-18 |
| ARHGEF10 | 1,03E-22 | 0,376708 | 0,431 | 0,243 | 1,71E-18 |
| SORL1 | 1,06E-22 | 0,393504 | 0,416 | 0,206 | 1,76E-18 |
| STK39 | 1,16E-22 | 0,381909 | 0,573 | 0,375 | 1,92E-18 |
| KPNA2 | 1,18E-22 | -0,38241 | 0,399 | 0,575 | 1,96E-18 |
| RTN3 | 1,26E-22 | 0,321348 | 0,986 | 0,966 | 2,08E-18 |
| SCD | 1,32E-22 | 0,501996 | 0,994 | 0,991 | 2,19E-18 |
| PEBP1 | 1,78E-22 | 0,258677 | 1 | 1 | 2,95E-18 |
| CTD-2336O2.1 | 2,26E-22 | 0,279169 | 0,274 | 0,095 | 3,74E-18 |
| HABP4 | 2,29E-22 | 0,400348 | 0,745 | 0,571 | 3,79E-18 |
| GATM | 2,52E-22 | 0,382947 | 0,946 | 0,849 | 4,17E-18 |
| MAML2 | 2,54E-22 | -0,36239 | 0,292 | 0,492 | 4,2E-18 |
| ADA | 2,63E-22 | 0,379414 | 0,347 | 0,167 | 4,35E-18 |
| STXBP1 | 2,72E-22 | -0,39121 | 0,439 | 0,61 | 4,51E-18 |
| HIVEP1 | 2,74E-22 | -0,33967 | 0,2 | 0,404 | 4,54E-18 |
| GLDC | 2,97E-22 | -0,34522 | 0,054 | 0,183 | 4,92E-18 |
| NCAM1 | 3,54E-22 | 0,325689 | 0,983 | 0,94 | 5,85E-18 |
| ADPGK | 3,8E-22 | -0,34676 | 0,283 | 0,479 | 6,29E-18 |
| FAM171A1 | 4,5E-22 | 0,436264 | 0,696 | 0,55 | 7,45E-18 |
| HIP1 | 4,65E-22 | 0,40994 | 0,615 | 0,429 | 7,69E-18 |
| PRKCZ | 4,7E-22 | 0,39295 | 0,602 | 0,409 | 7,78E-18 |
| FREM2 | 4,74E-22 | 0,466738 | 0,644 | 0,455 | 7,84E-18 |
| DOPEY1 | 5,98E-22 | -0,37308 | 0,245 | 0,445 | 9,9E-18 |
| RGS10 | 6,33E-22 | -0,32832 | 0,088 | 0,249 | 1,05E-17 |
| ZFHX4-AS1 | 6,41E-22 | -0,30001 | 0,128 | 0,303 | 1,06E-17 |
| KDELC2 | 6,77E-22 | -0,32528 | 0,199 | 0,402 | 1,12E-17 |
| CDK6 | 7,05E-22 | 0,382698 | 0,46 | 0,253 | 1,17E-17 |
| PLP1 | 7,17E-22 | 0,703655 | 0,998 | 1 | 1,19E-17 |
| GNAO1 | 7,36E-22 | 0,419882 | 0,633 | 0,438 | 1,22E-17 |
| POLR2F | 9,06E-22 | 0,375585 | 0,904 | 0,811 | 1,5E-17 |
| TIMP1 | 9,11E-22 | -0,51454 | 0,862 | 0,922 | 1,51E-17 |
| ICK | 9,52E-22 | 0,472186 | 0,598 | 0,427 | 1,58E-17 |
| CCDC88A | 1,01E-21 | 0,423594 | 0,869 | 0,802 | 1,67E-17 |
| SYPL1 | 1,27E-21 | 0,369004 | 0,885 | 0,78 | 2,11E-17 |
| EEA1 | 1,42E-21 | -0,38986 | 0,762 | 0,869 | 2,35E-17 |
| LPIN2 | 1,47E-21 | -0,38129 | 0,453 | 0,642 | 2,44E-17 |
| MGAT5 | 1,74E-21 | 0,448404 | 0,542 | 0,375 | 2,87E-17 |
| BCAR1 | 1,78E-21 | 0,363827 | 0,419 | 0,221 | 2,95E-17 |
| IRF7 | 1,93E-21 | -0,36767 | 0,662 | 0,817 | 3,2E-17 |
| CD40 | 2,23E-21 | -0,29552 | 0,056 | 0,189 | 3,7E-17 |
| MRPL14 | 2,38E-21 | -0,31751 | 0,791 | 0,888 | 3,94E-17 |
| AKR1B1 | 2,38E-21 | -0,36706 | 0,647 | 0,78 | 3,94E-17 |
| PSMA3 | 2,43E-21 | -0,30525 | 0,945 | 0,975 | 4,03E-17 |
| RNF144A | 2,66E-21 | 0,324308 | 0,351 | 0,16 | 4,41E-17 |
| HAPLN2 | 2,72E-21 | 0,522509 | 0,764 | 0,613 | 4,5E-17 |
| HMGCR | 2,76E-21 | 0,554698 | 0,578 | 0,425 | 4,57E-17 |
| FAM63B | 3,02E-21 | 0,392995 | 0,533 | 0,335 | 5E-17 |
| IFT57 | 3,09E-21 | -0,36394 | 0,287 | 0,498 | 5,12E-17 |
| EIF2AK2 | 3,15E-21 | -0,32418 | 0,941 | 0,977 | 5,21E-17 |
| MMS19 | 3,25E-21 | 0,325979 | 0,378 | 0,192 | 5,38E-17 |
| DNAJC1 | 3,26E-21 | -0,38134 | 0,582 | 0,743 | 5,4E-17 |
| WWTR1 | 3,31E-21 | -0,41499 | 0,474 | 0,65 | 5,48E-17 |
| SERPINB8 | 3,5E-21 | -0,34163 | 0,3 | 0,499 | 5,79E-17 |
| DBP | 3,78E-21 | 0,257939 | 0,213 | 0,058 | 6,26E-17 |
| C19orf12 | 3,9E-21 | -0,35716 | 0,727 | 0,862 | 6,46E-17 |
| ZEB2 | 4,09E-21 | 0,356363 | 0,928 | 0,886 | 6,77E-17 |
| ALKBH7 | 4,09E-21 | 0,371444 | 0,779 | 0,624 | 6,78E-17 |
| CITED1 | 4,11E-21 | -0,72412 | 0,207 | 0,413 | 6,8E-17 |
| PSMA7 | 4,21E-21 | -0,27544 | 0,997 | 0,998 | 6,97E-17 |
| EML2 | 4,33E-21 | 0,333894 | 0,458 | 0,249 | 7,17E-17 |
| RP11-172H24.4 | 5,22E-21 | 0,311967 | 0,343 | 0,151 | 8,65E-17 |
| FAR1 | 5,74E-21 | 0,388608 | 0,639 | 0,535 | 9,51E-17 |
| LINC00657 | 5,83E-21 | 0,388518 | 0,819 | 0,679 | 9,65E-17 |
| SEMA7A | 5,87E-21 | 0,348124 | 0,358 | 0,167 | 9,72E-17 |
| SLC39A14 | 6,09E-21 | -0,32843 | 0,135 | 0,3 | 1,01E-16 |
| PTPRA | 6,39E-21 | -0,35395 | 0,891 | 0,945 | 1,06E-16 |
| BTG1 | 6,61E-21 | -0,44926 | 0,826 | 0,914 | 1,09E-16 |
| HEG1 | 6,72E-21 | -0,40383 | 0,526 | 0,687 | 1,11E-16 |
| ELOVL6 | 6,91E-21 | 0,429588 | 0,551 | 0,369 | 1,14E-16 |
| C8orf46 | 7,99E-21 | -0,36161 | 0,884 | 0,948 | 1,32E-16 |
| ABCA2 | 9,31E-21 | 0,339211 | 0,978 | 0,971 | 1,54E-16 |
| YWHAZ | 1,01E-20 | 0,300126 | 0,998 | 0,988 | 1,68E-16 |
| NOVA1 | 1,08E-20 | 0,386556 | 0,601 | 0,436 | 1,79E-16 |
| TXNL4A | 1,15E-20 | 0,330355 | 0,925 | 0,865 | 1,91E-16 |
| MIER1 | 1,29E-20 | -0,32652 | 0,71 | 0,822 | 2,13E-16 |
| ITPK1 | 1,47E-20 | 0,40779 | 0,704 | 0,548 | 2,43E-16 |
| SUN2 | 1,51E-20 | 0,37226 | 0,627 | 0,439 | 2,51E-16 |
| ZKSCAN1 | 1,68E-20 | 0,41182 | 0,651 | 0,501 | 2,78E-16 |
| ILVBL | 1,72E-20 | 0,396432 | 0,708 | 0,565 | 2,85E-16 |
| APPL2 | 1,74E-20 | 0,262395 | 0,195 | 0,052 | 2,89E-16 |
| PGAM1 | 1,86E-20 | -0,36113 | 0,817 | 0,906 | 3,07E-16 |
| IQSEC1 | 2,14E-20 | 0,289155 | 0,274 | 0,108 | 3,54E-16 |
| C1orf198 | 2,48E-20 | 0,381187 | 0,766 | 0,624 | 4,1E-16 |
| EFHD1 | 2,49E-20 | 0,31867 | 0,99 | 0,949 | 4,13E-16 |
| RELL1 | 2,96E-20 | -0,27506 | 0,086 | 0,238 | 4,9E-16 |
| AC010226.4 | 2,98E-20 | -0,32524 | 0,251 | 0,447 | 4,94E-16 |
| POLR2I | 3,07E-20 | 0,386262 | 0,853 | 0,748 | 5,08E-16 |
| OTUD4 | 3,24E-20 | -0,35614 | 0,456 | 0,648 | 5,37E-16 |
| SLC27A4 | 3,48E-20 | 0,314502 | 0,35 | 0,171 | 5,77E-16 |
| ZNF536 | 3,75E-20 | 0,390688 | 0,628 | 0,439 | 6,21E-16 |
| BLVRA | 3,85E-20 | -0,33529 | 0,349 | 0,544 | 6,38E-16 |
| ARHGEF2 | 4,59E-20 | 0,383223 | 0,603 | 0,418 | 7,6E-16 |
| RBCK1 | 5,35E-20 | -0,33659 | 0,812 | 0,911 | 8,86E-16 |
| PTX3 | 5,55E-20 | -0,61876 | 0,018 | 0,109 | 9,2E-16 |
| EXT1 | 5,76E-20 | -0,29345 | 0,181 | 0,37 | 9,53E-16 |
| FAM84B | 6,03E-20 | 0,355384 | 0,443 | 0,252 | 9,99E-16 |
| CYLD | 6,18E-20 | -0,37746 | 0,914 | 0,955 | 1,02E-15 |
| NDUFB2 | 6,27E-20 | 0,325396 | 0,988 | 0,98 | 1,04E-15 |
| NOD2 | 6,31E-20 | -0,31463 | 0,158 | 0,327 | 1,04E-15 |
| COPA | 6,32E-20 | 0,383537 | 0,886 | 0,808 | 1,05E-15 |
| NDRG1 | 6,53E-20 | 0,363074 | 0,898 | 0,788 | 1,08E-15 |
| BMP2 | 7,26E-20 | -0,34309 | 0,072 | 0,209 | 1,2E-15 |
| RAB30 | 7,59E-20 | -0,37625 | 0,432 | 0,588 | 1,26E-15 |
| RDX | 7,86E-20 | -0,41894 | 0,993 | 0,998 | 1,3E-15 |
| TAGLN2 | 9,18E-20 | -0,36401 | 0,839 | 0,914 | 1,52E-15 |
| PRUNE2 | 1,04E-19 | 0,381349 | 0,823 | 0,699 | 1,72E-15 |
| GADD45A | 1,08E-19 | 0,423446 | 0,361 | 0,174 | 1,79E-15 |
| SFXN3 | 1,39E-19 | 0,349322 | 0,476 | 0,295 | 2,3E-15 |
| KLK6 | 1,47E-19 | 0,373113 | 0,994 | 0,991 | 2,44E-15 |
| JHDM1D-AS1 | 1,53E-19 | -0,28486 | 0,132 | 0,296 | 2,53E-15 |
| SFPQ | 1,56E-19 | -0,38668 | 0,854 | 0,92 | 2,59E-15 |
| MAT2A | 1,62E-19 | 0,399655 | 0,753 | 0,582 | 2,68E-15 |
| FKBP4 | 1,69E-19 | 0,370268 | 0,46 | 0,278 | 2,79E-15 |
| BACE1 | 1,69E-19 | 0,414059 | 0,864 | 0,811 | 2,79E-15 |
| ARID5B | 1,7E-19 | -0,45016 | 0,611 | 0,677 | 2,81E-15 |
| KIAA0930 | 1,82E-19 | 0,415738 | 0,722 | 0,575 | 3,02E-15 |
| VSTM2B | 2,11E-19 | 0,268017 | 0,207 | 0,061 | 3,5E-15 |
| KANK1 | 2,17E-19 | 0,284866 | 0,283 | 0,109 | 3,59E-15 |
| PARP8 | 2,22E-19 | -0,30462 | 0,222 | 0,415 | 3,68E-15 |
| SLC37A1 | 2,52E-19 | -0,27833 | 0,147 | 0,324 | 4,18E-15 |
| GOLGA4 | 2,61E-19 | 0,4396 | 0,761 | 0,611 | 4,33E-15 |
| GNG5 | 2,64E-19 | -0,29995 | 0,923 | 0,959 | 4,38E-15 |
| OLIG2 | 2,73E-19 | 0,282954 | 0,212 | 0,066 | 4,52E-15 |
| UBE2R2 | 2,81E-19 | 0,35273 | 0,663 | 0,487 | 4,65E-15 |
| BBC3 | 3,27E-19 | -0,35294 | 0,294 | 0,495 | 5,41E-15 |
| LMNA | 3,53E-19 | 0,283353 | 0,972 | 0,937 | 5,85E-15 |
| GK | 3,62E-19 | -0,30186 | 0,196 | 0,367 | 6E-15 |
| RPS6KA2 | 3,65E-19 | 0,383434 | 0,781 | 0,648 | 6,04E-15 |
| PTPN13 | 3,79E-19 | 0,359311 | 0,68 | 0,484 | 6,27E-15 |
| ELK1 | 3,9E-19 | -0,28144 | 0,173 | 0,346 | 6,45E-15 |
| JAK2 | 4,08E-19 | -0,36121 | 0,649 | 0,8 | 6,76E-15 |
| SEMA4D | 4,27E-19 | 0,453291 | 0,757 | 0,671 | 7,06E-15 |
| CASP1 | 4,27E-19 | -0,27717 | 0,114 | 0,272 | 7,07E-15 |
| CD81 | 4,85E-19 | -0,32179 | 0,998 | 1 | 8,04E-15 |
| PSMB9 | 5,03E-19 | -0,26691 | 0,992 | 1 | 8,32E-15 |
| TCEAL7 | 5,5E-19 | 0,375359 | 0,538 | 0,333 | 9,1E-15 |
| IFI27 | 6,16E-19 | -0,44188 | 0,154 | 0,326 | 1,02E-14 |
| SGCB | 6,61E-19 | -0,2968 | 0,89 | 0,922 | 1,09E-14 |
| PIK3R1 | 6,84E-19 | 0,407583 | 0,625 | 0,444 | 1,13E-14 |
| CLCN3 | 7,49E-19 | 0,360032 | 0,906 | 0,797 | 1,24E-14 |
| FGL2 | 7,53E-19 | -0,33117 | 0,065 | 0,198 | 1,25E-14 |
| TRAPPC2L | 8,12E-19 | 0,325867 | 0,909 | 0,82 | 1,34E-14 |
| MRPL17 | 8,14E-19 | -0,32624 | 0,473 | 0,665 | 1,35E-14 |
| SERPING1 | 8,65E-19 | -0,29863 | 0,043 | 0,151 | 1,43E-14 |
| HMGCS1 | 9,03E-19 | 0,638405 | 0,841 | 0,82 | 1,49E-14 |
| PNPT1 | 9,48E-19 | -0,36898 | 0,538 | 0,679 | 1,57E-14 |
| MEGF10 | 9,56E-19 | 0,363091 | 0,517 | 0,332 | 1,58E-14 |
| LARP1 | 9,62E-19 | 0,342449 | 0,866 | 0,745 | 1,59E-14 |
| NAV2 | 1,02E-18 | 0,387003 | 0,561 | 0,369 | 1,69E-14 |
| ZNF365 | 1,02E-18 | 0,275989 | 0,283 | 0,121 | 1,7E-14 |
| CREB3 | 1,07E-18 | -0,30035 | 0,407 | 0,613 | 1,78E-14 |
| IL15RA | 1,09E-18 | -0,31644 | 0,292 | 0,485 | 1,81E-14 |
| IGFBP4 | 1,17E-18 | -0,30869 | 0,048 | 0,164 | 1,93E-14 |
| MPST | 1,23E-18 | 0,352619 | 0,578 | 0,398 | 2,04E-14 |
| RAP2A | 1,45E-18 | 0,361671 | 0,657 | 0,484 | 2,4E-14 |
| PPFIBP2 | 1,54E-18 | 0,295378 | 0,35 | 0,18 | 2,56E-14 |
| TF | 1,73E-18 | 0,526942 | 0,993 | 0,995 | 2,86E-14 |
| ARMCX1 | 1,87E-18 | -0,31304 | 0,245 | 0,41 | 3,1E-14 |
| KIAA1033 | 2,04E-18 | -0,33626 | 0,834 | 0,919 | 3,37E-14 |
| MOB3B | 2,07E-18 | 0,422798 | 0,79 | 0,671 | 3,42E-14 |
| PMAIP1 | 2,28E-18 | -0,39485 | 0,102 | 0,249 | 3,77E-14 |
| FUT8 | 2,35E-18 | 0,346754 | 0,591 | 0,425 | 3,89E-14 |
| PCYOX1 | 2,39E-18 | 0,336659 | 0,487 | 0,301 | 3,96E-14 |
| TMEM208 | 2,87E-18 | -0,31051 | 0,844 | 0,928 | 4,76E-14 |
| SSBP3 | 3,05E-18 | 0,275659 | 0,318 | 0,141 | 5,05E-14 |
| PBX3 | 3,13E-18 | 0,311338 | 0,381 | 0,201 | 5,18E-14 |
| FGFR2 | 3,53E-18 | 0,498152 | 0,729 | 0,631 | 5,84E-14 |
| SGCD | 3,64E-18 | 0,334107 | 0,272 | 0,117 | 6,03E-14 |
| SLC44A2 | 3,83E-18 | 0,36938 | 0,746 | 0,593 | 6,34E-14 |
| KAZN | 3,9E-18 | 0,354049 | 0,461 | 0,278 | 6,46E-14 |
| MTURN | 4,46E-18 | 0,379957 | 0,748 | 0,575 | 7,38E-14 |
| PPP1R15A | 4,64E-18 | -0,36933 | 0,685 | 0,811 | 7,69E-14 |
| GIT1 | 5,19E-18 | 0,372447 | 0,769 | 0,628 | 8,6E-14 |
| ABHD6 | 5,23E-18 | 0,331783 | 0,494 | 0,332 | 8,66E-14 |
| SLC25A36 | 5,33E-18 | 0,325472 | 0,487 | 0,293 | 8,82E-14 |
| AGO2 | 6,04E-18 | 0,326872 | 0,456 | 0,273 | 1E-13 |
| CASP3 | 6,32E-18 | -0,33647 | 0,285 | 0,465 | 1,05E-13 |
| ANKRD39 | 6,68E-18 | 0,31567 | 0,403 | 0,243 | 1,11E-13 |
| WDR45 | 6,98E-18 | -0,32146 | 0,476 | 0,657 | 1,16E-13 |
| CNTLN | 7,11E-18 | -0,3319 | 0,268 | 0,441 | 1,18E-13 |
| RNF130 | 7,22E-18 | 0,28296 | 0,963 | 0,9 | 1,19E-13 |
| DHCR7 | 7,38E-18 | 0,366523 | 0,496 | 0,32 | 1,22E-13 |
| PALM2 | 7,67E-18 | -0,31472 | 0,185 | 0,356 | 1,27E-13 |
| LACC1 | 7,74E-18 | -0,3248 | 0,284 | 0,465 | 1,28E-13 |
| GSTK1 | 9,01E-18 | -0,30016 | 0,833 | 0,914 | 1,49E-13 |
| HLA-DRA | 9,36E-18 | -0,55868 | 0,235 | 0,326 | 1,55E-13 |
| CCDC71L | 9,49E-18 | -0,3697 | 0,556 | 0,713 | 1,57E-13 |
| SYT11 | 1,04E-17 | 0,37364 | 0,979 | 0,963 | 1,72E-13 |
| KCND3 | 1,05E-17 | 0,396542 | 0,748 | 0,621 | 1,74E-13 |
| BOK | 1,06E-17 | 0,339726 | 0,481 | 0,303 | 1,76E-13 |
| TMEM184B | 1,08E-17 | 0,297898 | 0,336 | 0,178 | 1,78E-13 |
| COLGALT2 | 1,08E-17 | 0,369484 | 0,673 | 0,515 | 1,79E-13 |
| NACAD | 1,21E-17 | 0,359013 | 0,892 | 0,839 | 2E-13 |
| SLC25A3 | 1,24E-17 | 0,269473 | 0,985 | 0,949 | 2,06E-13 |
| TSPAN15 | 1,45E-17 | 0,367917 | 0,847 | 0,714 | 2,4E-13 |
| MICALL2 | 1,47E-17 | -0,29022 | 0,411 | 0,616 | 2,43E-13 |
| SERINC3 | 1,54E-17 | 0,341776 | 0,873 | 0,797 | 2,56E-13 |
| RAB29 | 1,56E-17 | -0,33677 | 0,343 | 0,521 | 2,58E-13 |
| TNFSF13B | 1,6E-17 | -0,31186 | 0,05 | 0,157 | 2,65E-13 |
| TFPI2 | 1,66E-17 | -0,44181 | 0,075 | 0,204 | 2,74E-13 |
| TBL1XR1 | 1,7E-17 | 0,343804 | 0,465 | 0,284 | 2,81E-13 |
| BUB3 | 1,75E-17 | 0,335245 | 0,64 | 0,479 | 2,89E-13 |
| HNRNPA2B1 | 2,09E-17 | -0,2655 | 0,984 | 0,998 | 3,47E-13 |
| SQRDL | 2,33E-17 | -0,25362 | 0,105 | 0,252 | 3,86E-13 |
| EFR3B | 2,43E-17 | 0,278593 | 0,32 | 0,155 | 4,03E-13 |
| JAK1 | 2,45E-17 | -0,32915 | 0,564 | 0,713 | 4,06E-13 |
| SH3GL3 | 2,48E-17 | 0,332411 | 0,467 | 0,306 | 4,1E-13 |
| ETFB | 2,62E-17 | 0,313817 | 0,951 | 0,891 | 4,33E-13 |
| PTPRF | 2,66E-17 | 0,331666 | 0,619 | 0,469 | 4,4E-13 |
| ATP1A1 | 2,66E-17 | 0,353583 | 0,924 | 0,88 | 4,4E-13 |
| C1orf122 | 2,67E-17 | 0,294958 | 0,99 | 0,975 | 4,43E-13 |
| GBP5 | 2,72E-17 | -0,31876 | 0,108 | 0,253 | 4,5E-13 |
| PDE8A | 2,77E-17 | 0,328031 | 0,433 | 0,253 | 4,58E-13 |
| NMT1 | 3,04E-17 | -0,32287 | 0,567 | 0,725 | 5,04E-13 |
| MIR155HG | 3,08E-17 | -0,27525 | 0,079 | 0,178 | 5,1E-13 |
| PFKFB3 | 3,4E-17 | -0,37342 | 0,212 | 0,367 | 5,63E-13 |
| ACP2 | 3,54E-17 | -0,35157 | 0,454 | 0,608 | 5,86E-13 |
| DLG2 | 3,65E-17 | 0,331705 | 0,587 | 0,419 | 6,05E-13 |
| NES | 3,72E-17 | -0,40096 | 0,224 | 0,372 | 6,17E-13 |
| QKI | 3,91E-17 | 0,365561 | 0,999 | 0,995 | 6,47E-13 |
| CBR1 | 3,91E-17 | 0,357127 | 0,986 | 0,972 | 6,47E-13 |
| ZBED3 | 4,14E-17 | 0,307222 | 0,347 | 0,174 | 6,85E-13 |
| RNF138 | 4,27E-17 | -0,29302 | 0,274 | 0,461 | 7,07E-13 |
| SESN3 | 4,33E-17 | 0,332253 | 0,278 | 0,127 | 7,16E-13 |
| HIPK2 | 4,87E-17 | 0,378143 | 0,957 | 0,92 | 8,07E-13 |
| JAM3 | 4,99E-17 | 0,340084 | 0,82 | 0,688 | 8,26E-13 |
| CNOT4 | 5,05E-17 | -0,30416 | 0,436 | 0,614 | 8,36E-13 |
| MYO9B | 5,07E-17 | 0,353406 | 0,743 | 0,604 | 8,39E-13 |
| MTMR10 | 5,35E-17 | 0,35199 | 0,569 | 0,425 | 8,85E-13 |
| ATF5 | 5,4E-17 | -0,383 | 0,198 | 0,366 | 8,94E-13 |
| STAT2 | 5,53E-17 | -0,3078 | 0,913 | 0,966 | 9,16E-13 |
| LAMTOR4 | 5,56E-17 | 0,261995 | 0,99 | 0,977 | 9,21E-13 |
| RNASE1 | 6,1E-17 | 0,450319 | 0,957 | 0,942 | 1,01E-12 |
| BCAT1 | 6,32E-17 | 0,366092 | 0,347 | 0,18 | 1,05E-12 |
| DGKZ | 7,14E-17 | 0,331093 | 0,66 | 0,516 | 1,18E-12 |
| NR1D2 | 7,14E-17 | 0,302617 | 0,345 | 0,18 | 1,18E-12 |
| SHISA5 | 7,64E-17 | 0,332494 | 0,739 | 0,601 | 1,26E-12 |
| CRYL1 | 7,87E-17 | 0,3342 | 0,717 | 0,578 | 1,3E-12 |
| CSNK1E | 7,98E-17 | 0,327857 | 0,695 | 0,576 | 1,32E-12 |
| CCDC112 | 8,9E-17 | 0,311571 | 0,352 | 0,189 | 1,47E-12 |
| CDKN1A | 9E-17 | -0,30867 | 0,667 | 0,826 | 1,49E-12 |
| HOMER3 | 9,13E-17 | 0,386624 | 0,917 | 0,842 | 1,51E-12 |
| EVI2A | 9,36E-17 | 0,254751 | 0,254 | 0,101 | 1,55E-12 |
| PRDX1 | 9,62E-17 | 0,261607 | 1 | 1 | 1,59E-12 |
| PAPSS1 | 9,74E-17 | 0,347546 | 0,684 | 0,565 | 1,61E-12 |
| PLEKHM2 | 1,01E-16 | -0,30832 | 0,447 | 0,627 | 1,67E-12 |
| ARFGEF3 | 1,04E-16 | 0,36029 | 0,709 | 0,581 | 1,72E-12 |
| LBR | 1,05E-16 | 0,322919 | 0,472 | 0,306 | 1,73E-12 |
| FAM134B | 1,2E-16 | 0,281087 | 0,975 | 0,923 | 1,99E-12 |
| NLGN3 | 1,21E-16 | 0,312404 | 0,334 | 0,174 | 2,01E-12 |
| TMED5 | 1,3E-16 | -0,31018 | 0,657 | 0,756 | 2,15E-12 |
| SFT2D2 | 1,39E-16 | -0,33567 | 0,731 | 0,845 | 2,31E-12 |
| ATP11C | 1,41E-16 | -0,30584 | 0,324 | 0,51 | 2,34E-12 |
| FAM53B | 1,42E-16 | 0,336121 | 0,836 | 0,75 | 2,36E-12 |
| RND3 | 1,45E-16 | -0,30143 | 0,031 | 0,127 | 2,4E-12 |
| CPEB2 | 1,56E-16 | 0,370164 | 0,606 | 0,478 | 2,58E-12 |
| LANCL1 | 1,69E-16 | 0,328073 | 0,537 | 0,401 | 2,8E-12 |
| TRIB3 | 1,83E-16 | 0,473892 | 0,42 | 0,269 | 3,03E-12 |
| ETV7 | 1,92E-16 | -0,293 | 0,873 | 0,94 | 3,18E-12 |
| EID2 | 1,96E-16 | -0,27968 | 0,245 | 0,407 | 3,25E-12 |
| LRRC4B | 2,07E-16 | 0,259707 | 0,254 | 0,104 | 3,42E-12 |
| UROS | 2,1E-16 | 0,320942 | 0,758 | 0,604 | 3,48E-12 |
| FNTB | 2,1E-16 | 0,360896 | 0,682 | 0,551 | 3,48E-12 |
| BICD2 | 2,44E-16 | 0,284543 | 0,338 | 0,186 | 4,04E-12 |
| SQSTM1 | 2,58E-16 | -0,28515 | 0,997 | 0,998 | 4,28E-12 |
| DESI1 | 2,71E-16 | -0,32279 | 0,646 | 0,768 | 4,48E-12 |
| SOX2 | 2,75E-16 | -0,48951 | 0,841 | 0,862 | 4,55E-12 |
| IL15 | 2,89E-16 | -0,34104 | 0,391 | 0,553 | 4,79E-12 |
| SEPT8 | 2,9E-16 | 0,331486 | 0,927 | 0,876 | 4,8E-12 |
| BMI1 | 3,03E-16 | 0,298343 | 0,573 | 0,441 | 5,02E-12 |
| CBX4 | 3,06E-16 | 0,317549 | 0,454 | 0,276 | 5,07E-12 |
| TARSL2 | 3,26E-16 | 0,306369 | 0,398 | 0,241 | 5,39E-12 |
| ILK | 3,35E-16 | -0,29013 | 0,761 | 0,851 | 5,55E-12 |
| PDPN | 3,45E-16 | -0,39829 | 0,021 | 0,103 | 5,71E-12 |
| ITPKB | 3,45E-16 | 0,405844 | 0,602 | 0,435 | 5,71E-12 |
| TNKS2 | 3,53E-16 | -0,33065 | 0,766 | 0,845 | 5,84E-12 |
| IGF2BP2 | 3,58E-16 | -0,26179 | 0,14 | 0,296 | 5,92E-12 |
| ADAR | 3,92E-16 | -0,25916 | 0,953 | 0,974 | 6,5E-12 |
| CAV1 | 3,95E-16 | 0,437247 | 0,435 | 0,247 | 6,54E-12 |
| CDC42SE2 | 4,02E-16 | -0,31456 | 0,697 | 0,826 | 6,66E-12 |
| DIP2C | 4,16E-16 | 0,317911 | 0,762 | 0,618 | 6,9E-12 |
| GPSM2 | 4,36E-16 | 0,29332 | 0,431 | 0,258 | 7,22E-12 |
| WNK1 | 4,51E-16 | 0,357656 | 0,902 | 0,866 | 7,47E-12 |
| GREM1 | 4,66E-16 | -0,48238 | 0,429 | 0,581 | 7,71E-12 |
| SPP1 | 4,86E-16 | 0,385902 | 0,977 | 0,965 | 8,04E-12 |
| IFI27L1 | 4,88E-16 | -0,29127 | 0,292 | 0,475 | 8,08E-12 |
| EIF1AY | 5,09E-16 | -0,27723 | 0,799 | 0,828 | 8,43E-12 |
| DYNLL2 | 5,27E-16 | 0,334008 | 0,643 | 0,501 | 8,73E-12 |
| XBP1 | 5,36E-16 | -0,36165 | 0,697 | 0,8 | 8,88E-12 |
| APBB2 | 5,76E-16 | 0,330884 | 0,782 | 0,639 | 9,54E-12 |
| BNIP3L | 6,2E-16 | 0,337823 | 0,736 | 0,599 | 1,03E-11 |
| DNM3 | 6,41E-16 | 0,337751 | 0,81 | 0,694 | 1,06E-11 |
| NDUFA9 | 6,42E-16 | -0,28099 | 0,806 | 0,859 | 1,06E-11 |
| DPYSL3 | 6,49E-16 | -0,29745 | 0,201 | 0,361 | 1,07E-11 |
| GABPB1-AS1 | 6,81E-16 | 0,35517 | 0,45 | 0,278 | 1,13E-11 |
| DYNLT3 | 7,44E-16 | -0,32321 | 0,428 | 0,605 | 1,23E-11 |
| UBA6 | 7,81E-16 | -0,31803 | 0,693 | 0,797 | 1,29E-11 |
| PDE1C | 8,19E-16 | 0,327328 | 0,576 | 0,418 | 1,36E-11 |
| SLC25A13 | 8,23E-16 | 0,327027 | 0,493 | 0,343 | 1,36E-11 |
| PSD3 | 8,64E-16 | 0,320327 | 0,421 | 0,264 | 1,43E-11 |
| ACAP3 | 8,65E-16 | 0,291987 | 0,418 | 0,25 | 1,43E-11 |
| ZNF652 | 8,91E-16 | 0,298714 | 0,393 | 0,226 | 1,47E-11 |
| NDUFS7 | 9,43E-16 | 0,274964 | 0,985 | 0,968 | 1,56E-11 |
| MLLT6 | 1,01E-15 | -0,30743 | 0,399 | 0,571 | 1,67E-11 |
| PCYT2 | 1,1E-15 | 0,308459 | 0,654 | 0,518 | 1,82E-11 |
| MYEOV2 | 1,12E-15 | 0,28285 | 0,922 | 0,859 | 1,86E-11 |
| PER3 | 1,15E-15 | 0,293175 | 0,423 | 0,241 | 1,91E-11 |
| EGR2 | 1,19E-15 | -0,27933 | 0,042 | 0,135 | 1,97E-11 |
| TECPR2 | 1,26E-15 | 0,299255 | 0,407 | 0,252 | 2,09E-11 |
| GRIK4 | 1,35E-15 | 0,281656 | 0,376 | 0,226 | 2,23E-11 |
| KIAA0355 | 1,35E-15 | 0,301067 | 0,465 | 0,296 | 2,24E-11 |
| ERMP1 | 1,36E-15 | 0,314485 | 0,498 | 0,341 | 2,25E-11 |
| ANKRD28 | 1,38E-15 | 0,344706 | 0,627 | 0,464 | 2,29E-11 |
| ATP6V0B | 1,38E-15 | 0,27364 | 0,994 | 0,983 | 2,29E-11 |
| GNAI1 | 1,43E-15 | 0,351003 | 0,753 | 0,61 | 2,37E-11 |
| SRPK2 | 1,45E-15 | 0,277447 | 0,98 | 0,974 | 2,4E-11 |
| UBXN4 | 1,56E-15 | -0,27841 | 0,966 | 0,975 | 2,58E-11 |
| CERS1 | 1,62E-15 | 0,300143 | 0,458 | 0,286 | 2,68E-11 |
| PHACTR4 | 1,69E-15 | -0,31001 | 0,751 | 0,854 | 2,8E-11 |
| DHX36 | 1,72E-15 | -0,28844 | 0,806 | 0,888 | 2,84E-11 |
| SNX6 | 1,95E-15 | -0,25313 | 0,967 | 0,982 | 3,22E-11 |
| GCLM | 2E-15 | -0,36577 | 0,682 | 0,783 | 3,31E-11 |
| SOGA1 | 2,04E-15 | 0,271198 | 0,325 | 0,177 | 3,38E-11 |
| SLF2 | 2,48E-15 | 0,311686 | 0,601 | 0,459 | 4,11E-11 |
| NUDCD1 | 2,54E-15 | -0,27484 | 0,21 | 0,366 | 4,2E-11 |
| ADSS | 3E-15 | 0,320224 | 0,61 | 0,458 | 4,97E-11 |
| LZTS2 | 3,02E-15 | 0,328746 | 0,624 | 0,505 | 4,99E-11 |
| NNT-AS1 | 3,02E-15 | 0,278996 | 0,395 | 0,221 | 5E-11 |
| RASA3 | 3,07E-15 | 0,258001 | 0,287 | 0,141 | 5,08E-11 |
| CYTH1 | 3,16E-15 | 0,298625 | 0,436 | 0,278 | 5,23E-11 |
| PCDH17 | 3,21E-15 | -0,31427 | 0,939 | 0,977 | 5,31E-11 |
| KATNAL1 | 3,25E-15 | 0,293318 | 0,491 | 0,31 | 5,38E-11 |
| CBLB | 3,45E-15 | -0,28513 | 0,247 | 0,386 | 5,71E-11 |
| PEX5L | 3,55E-15 | 0,348898 | 0,806 | 0,696 | 5,87E-11 |
| RMND5A | 3,58E-15 | 0,267281 | 0,374 | 0,204 | 5,93E-11 |
| ATP6V0E2 | 3,7E-15 | 0,310618 | 0,643 | 0,515 | 6,12E-11 |
| ENO2 | 4,04E-15 | 0,302077 | 0,661 | 0,495 | 6,69E-11 |
| RCN1 | 4,06E-15 | -0,31898 | 0,351 | 0,527 | 6,71E-11 |
| PDCD5 | 4,08E-15 | -0,29885 | 0,754 | 0,845 | 6,76E-11 |
| CREB3L2 | 4,22E-15 | 0,27108 | 0,351 | 0,19 | 6,99E-11 |
| LLGL1 | 4,26E-15 | 0,311303 | 0,588 | 0,429 | 7,05E-11 |
| GPR62 | 4,35E-15 | 0,282781 | 0,376 | 0,207 | 7,21E-11 |
| EIF5 | 4,82E-15 | -0,29209 | 0,878 | 0,932 | 7,98E-11 |
| HEPACAM | 4,85E-15 | 0,363009 | 0,759 | 0,642 | 8,03E-11 |
| ENPP4 | 5,12E-15 | 0,305461 | 0,5 | 0,355 | 8,48E-11 |
| MICAL3 | 5,3E-15 | 0,321388 | 0,595 | 0,441 | 8,77E-11 |
| H2AFX | 5,43E-15 | 0,302962 | 0,413 | 0,272 | 8,99E-11 |
| RP11-161M6.2 | 5,52E-15 | 0,313772 | 0,547 | 0,382 | 9,13E-11 |
| CERCAM | 5,57E-15 | 0,350085 | 0,613 | 0,458 | 9,22E-11 |
| MUM1 | 5,72E-15 | 0,291521 | 0,445 | 0,273 | 9,47E-11 |
| CITED4 | 6,08E-15 | -0,50798 | 0,283 | 0,427 | 1,01E-10 |
| FNBP1 | 6,88E-15 | 0,319623 | 0,757 | 0,619 | 1,14E-10 |
| CMC2 | 6,94E-15 | 0,301558 | 0,723 | 0,582 | 1,15E-10 |
| RILPL1 | 7,13E-15 | 0,310702 | 0,657 | 0,516 | 1,18E-10 |
| ARHGAP23 | 7,26E-15 | 0,322767 | 0,616 | 0,498 | 1,2E-10 |
| EIF4G3 | 7,55E-15 | 0,330096 | 0,697 | 0,568 | 1,25E-10 |
| BBX | 7,6E-15 | -0,27901 | 0,953 | 0,986 | 1,26E-10 |
| DYNC1I2 | 7,62E-15 | 0,271134 | 0,972 | 0,96 | 1,26E-10 |
| CD22 | 7,64E-15 | 0,250575 | 0,28 | 0,147 | 1,26E-10 |
| RB1 | 8,42E-15 | -0,29876 | 0,565 | 0,674 | 1,39E-10 |
| HMSD | 8,72E-15 | -0,27953 | 0,301 | 0,481 | 1,44E-10 |
| SIRPA | 8,86E-15 | 0,265915 | 0,316 | 0,166 | 1,47E-10 |
| FAM200B | 9,67E-15 | 0,31333 | 0,54 | 0,379 | 1,6E-10 |
| WWP1 | 1,01E-14 | 0,280755 | 0,425 | 0,258 | 1,68E-10 |
| ZBTB4 | 1,02E-14 | 0,281214 | 0,433 | 0,273 | 1,68E-10 |
| LIMCH1 | 1,03E-14 | 0,328318 | 0,91 | 0,86 | 1,71E-10 |
| SASH1 | 1,04E-14 | 0,288366 | 0,748 | 0,579 | 1,73E-10 |
| INSR | 1,18E-14 | 0,293541 | 0,473 | 0,301 | 1,95E-10 |
| HP1BP3 | 1,43E-14 | 0,309123 | 0,824 | 0,713 | 2,36E-10 |
| GNB4 | 1,53E-14 | -0,29942 | 0,657 | 0,799 | 2,53E-10 |
| CAT | 1,53E-14 | 0,312857 | 0,562 | 0,41 | 2,54E-10 |
| HSD17B12 | 1,54E-14 | 0,33643 | 0,864 | 0,836 | 2,55E-10 |
| CNKSR3 | 1,56E-14 | -0,33902 | 0,269 | 0,439 | 2,59E-10 |
| FAM89A | 1,65E-14 | 0,372011 | 0,722 | 0,587 | 2,73E-10 |
| CSRP1 | 1,66E-14 | 0,329003 | 0,887 | 0,799 | 2,74E-10 |
| FUS | 1,73E-14 | -0,26417 | 0,845 | 0,916 | 2,86E-10 |
| CTSO | 1,74E-14 | -0,31773 | 0,677 | 0,805 | 2,88E-10 |
| SLC25A4 | 1,87E-14 | 0,333082 | 0,725 | 0,601 | 3,1E-10 |
| REL | 1,93E-14 | -0,26615 | 0,201 | 0,361 | 3,19E-10 |
| HIP1R | 2,17E-14 | 0,255508 | 0,328 | 0,169 | 3,59E-10 |
| KDM7A | 2,17E-14 | -0,31271 | 0,331 | 0,496 | 3,6E-10 |
| PRRC2C | 2,18E-14 | -0,27033 | 0,906 | 0,955 | 3,61E-10 |
| THRA | 2,26E-14 | 0,304179 | 0,531 | 0,364 | 3,74E-10 |
| TRAF4 | 2,38E-14 | 0,263975 | 0,372 | 0,209 | 3,93E-10 |
| UHRF1BP1 | 2,45E-14 | -0,28421 | 0,255 | 0,406 | 4,06E-10 |
| TNFAIP8 | 2,6E-14 | -0,27594 | 0,148 | 0,272 | 4,31E-10 |
| KLF9 | 2,71E-14 | -0,31617 | 0,598 | 0,743 | 4,48E-10 |
| IL17F | 2,73E-14 | -0,26535 | 0,249 | 0,422 | 4,53E-10 |
| MYD88 | 2,79E-14 | -0,2745 | 0,356 | 0,518 | 4,62E-10 |
| RNF220 | 2,92E-14 | 0,31492 | 0,573 | 0,421 | 4,83E-10 |
| CHCHD10 | 2,93E-14 | 0,322887 | 0,677 | 0,555 | 4,84E-10 |
| UBE2D1 | 3,66E-14 | -0,28481 | 0,535 | 0,677 | 6,05E-10 |
| DSTYK | 3,77E-14 | 0,28523 | 0,402 | 0,241 | 6,24E-10 |
| CRYAB | 3,82E-14 | 0,388594 | 1 | 0,998 | 6,33E-10 |
| NAV1 | 3,91E-14 | 0,29237 | 0,36 | 0,206 | 6,47E-10 |
| SLC35D2 | 4,59E-14 | 0,297134 | 0,637 | 0,482 | 7,59E-10 |
| KLF7 | 4,82E-14 | 0,305638 | 0,629 | 0,464 | 7,98E-10 |
| PYGB | 5,01E-14 | 0,279919 | 0,472 | 0,303 | 8,3E-10 |
| MED17 | 5,11E-14 | -0,26532 | 0,34 | 0,484 | 8,45E-10 |
| HDAC2 | 5,19E-14 | 0,286972 | 0,553 | 0,407 | 8,59E-10 |
| CRB2 | 5,25E-14 | -0,31048 | 0,308 | 0,473 | 8,7E-10 |
| DDHD1 | 5,31E-14 | 0,26297 | 0,349 | 0,192 | 8,79E-10 |
| TMEM256 | 5,39E-14 | 0,288375 | 0,724 | 0,593 | 8,93E-10 |
| PTPDC1 | 5,44E-14 | 0,300503 | 0,388 | 0,238 | 9,01E-10 |
| MTMR2 | 5,91E-14 | 0,289462 | 0,469 | 0,316 | 9,79E-10 |
| AKAP6 | 5,92E-14 | 0,302362 | 0,786 | 0,645 | 9,81E-10 |
| NRBP2 | 6,44E-14 | 0,314095 | 0,646 | 0,512 | 1,07E-09 |
| CIRBP | 6,54E-14 | 0,289806 | 0,963 | 0,935 | 1,08E-09 |
| REEP3 | 6,95E-14 | 0,272864 | 0,902 | 0,849 | 1,15E-09 |
| UBTF | 7,51E-14 | 0,27802 | 0,444 | 0,276 | 1,24E-09 |
| USF2 | 7,55E-14 | 0,290594 | 0,702 | 0,553 | 1,25E-09 |
| RCAN2 | 7,68E-14 | 0,307363 | 0,541 | 0,392 | 1,27E-09 |
| GPAT3 | 8,03E-14 | 0,28078 | 0,21 | 0,088 | 1,33E-09 |
| RASGRF2 | 8,79E-14 | 0,25051 | 0,265 | 0,129 | 1,45E-09 |
| CTTNBP2 | 8,92E-14 | 0,274017 | 0,402 | 0,238 | 1,48E-09 |
| CHADL | 9,44E-14 | 0,367288 | 0,856 | 0,806 | 1,56E-09 |
| FAIM2 | 9,62E-14 | 0,289775 | 0,906 | 0,848 | 1,59E-09 |
| LCP2 | 9,8E-14 | -0,26335 | 0,189 | 0,343 | 1,62E-09 |
| DOCK7 | 1E-13 | -0,27658 | 0,297 | 0,438 | 1,66E-09 |
| VAPA | 1,06E-13 | 0,257948 | 0,95 | 0,909 | 1,75E-09 |
| AIFM2 | 1,06E-13 | -0,28505 | 0,221 | 0,358 | 1,75E-09 |
| FAM107B | 1,13E-13 | 0,289113 | 0,96 | 0,928 | 1,87E-09 |
| ATP6V1A | 1,17E-13 | -0,31512 | 0,59 | 0,702 | 1,93E-09 |
| DOHH | 1,38E-13 | 0,280642 | 0,496 | 0,359 | 2,29E-09 |
| KRT10 | 1,41E-13 | 0,294684 | 0,848 | 0,753 | 2,34E-09 |
| HNRNPDL | 1,6E-13 | -0,26021 | 0,946 | 0,969 | 2,65E-09 |
| TDRD7 | 1,68E-13 | -0,26015 | 0,328 | 0,485 | 2,79E-09 |
| CMTM5 | 1,75E-13 | 0,318289 | 0,962 | 0,949 | 2,9E-09 |
| HES4 | 1,76E-13 | -0,30238 | 0,076 | 0,175 | 2,91E-09 |
| GRHPR | 1,82E-13 | 0,265752 | 0,873 | 0,777 | 3,02E-09 |
| C11orf24 | 2,01E-13 | 0,302717 | 0,718 | 0,593 | 3,34E-09 |
| SMARCA5 | 2,02E-13 | -0,30047 | 0,887 | 0,931 | 3,34E-09 |
| CCNY | 2,2E-13 | 0,290969 | 0,631 | 0,481 | 3,65E-09 |
| C9orf91 | 2,22E-13 | -0,28684 | 0,322 | 0,465 | 3,67E-09 |
| JUN | 2,4E-13 | -0,45106 | 0,364 | 0,522 | 3,97E-09 |
| RECQL | 2,41E-13 | -0,27949 | 0,516 | 0,654 | 4E-09 |
| RNF141 | 2,43E-13 | 0,293267 | 0,676 | 0,579 | 4,02E-09 |
| MRPS34 | 2,47E-13 | 0,272781 | 0,742 | 0,618 | 4,1E-09 |
| ZMYND8 | 2,57E-13 | 0,288673 | 0,385 | 0,224 | 4,26E-09 |
| FOXN2 | 2,59E-13 | 0,310837 | 0,569 | 0,418 | 4,29E-09 |
| KLF3 | 2,59E-13 | 0,288008 | 0,611 | 0,45 | 4,29E-09 |
| FAM120A | 2,71E-13 | 0,308172 | 0,799 | 0,714 | 4,48E-09 |
| SHISA4 | 2,73E-13 | 0,287705 | 0,5 | 0,372 | 4,53E-09 |
| C17orf89 | 2,91E-13 | 0,301128 | 0,663 | 0,538 | 4,82E-09 |
| MAPK8IP3 | 3,03E-13 | 0,268393 | 0,48 | 0,323 | 5,02E-09 |
| C1orf56 | 3,03E-13 | -0,33586 | 0,456 | 0,55 | 5,02E-09 |
| FAM73A | 3,07E-13 | 0,293606 | 0,73 | 0,636 | 5,08E-09 |
| CCL3 | 3,1E-13 | -0,25433 | 0,351 | 0,527 | 5,13E-09 |
| SEMA6A | 3,16E-13 | 0,337731 | 0,768 | 0,651 | 5,23E-09 |
| TMEM14A | 3,18E-13 | 0,258927 | 0,431 | 0,272 | 5,26E-09 |
| MED10 | 3,33E-13 | -0,27501 | 0,719 | 0,81 | 5,52E-09 |
| CYB5A | 3,82E-13 | -0,25963 | 0,786 | 0,846 | 6,32E-09 |
| PLEKHG3 | 4,11E-13 | -0,27582 | 0,272 | 0,392 | 6,8E-09 |
| DLL1 | 4,19E-13 | 0,256554 | 0,254 | 0,118 | 6,93E-09 |
| MPDZ | 4,32E-13 | 0,281727 | 0,449 | 0,307 | 7,15E-09 |
| ABCD3 | 4,52E-13 | 0,253055 | 0,477 | 0,373 | 7,49E-09 |
| TMEM206 | 5,24E-13 | 0,303397 | 0,726 | 0,608 | 8,68E-09 |
| SLC27A1 | 5,48E-13 | 0,294833 | 0,742 | 0,622 | 9,07E-09 |
| USP15 | 6,01E-13 | -0,29147 | 0,529 | 0,665 | 9,96E-09 |
| SPRY2 | 6,29E-13 | 0,265783 | 0,346 | 0,209 | 1,04E-08 |
| MVD | 6,33E-13 | 0,357375 | 0,563 | 0,444 | 1,05E-08 |
| GAB2 | 6,34E-13 | 0,262343 | 0,39 | 0,244 | 1,05E-08 |
| GPC1 | 6,38E-13 | 0,294343 | 0,688 | 0,564 | 1,06E-08 |
| SRCIN1 | 7,15E-13 | 0,30409 | 0,618 | 0,524 | 1,18E-08 |
| TMEM97 | 7,23E-13 | 0,38184 | 0,451 | 0,316 | 1,2E-08 |
| RPL13 | 7,61E-13 | 0,26727 | 1 | 1 | 1,26E-08 |
| PREPL | 7,82E-13 | 0,290893 | 0,735 | 0,61 | 1,29E-08 |
| RBM22 | 8,57E-13 | -0,28092 | 0,518 | 0,644 | 1,42E-08 |
| DHTKD1 | 8,72E-13 | -0,2725 | 0,519 | 0,674 | 1,44E-08 |
| C12orf76 | 8,83E-13 | 0,290198 | 0,753 | 0,618 | 1,46E-08 |
| ENY2 | 8,91E-13 | -0,27002 | 0,728 | 0,813 | 1,47E-08 |
| SCAMP1-AS1 | 9,36E-13 | -0,2581 | 0,314 | 0,473 | 1,55E-08 |
| RASAL2 | 9,46E-13 | -0,3045 | 0,393 | 0,556 | 1,57E-08 |
| SPSB1 | 9,57E-13 | -0,28861 | 0,422 | 0,548 | 1,59E-08 |
| YWHAH | 9,96E-13 | 0,371682 | 0,742 | 0,625 | 1,65E-08 |
| NIPA1 | 1,02E-12 | 0,279406 | 0,542 | 0,398 | 1,69E-08 |
| DUSP15 | 1,09E-12 | 0,303729 | 0,374 | 0,232 | 1,81E-08 |
| CALU | 1,1E-12 | 0,297102 | 0,855 | 0,78 | 1,83E-08 |
| TECR | 1,1E-12 | 0,270285 | 0,959 | 0,942 | 1,83E-08 |
| RBM7 | 1,11E-12 | -0,25253 | 0,359 | 0,518 | 1,83E-08 |
| ATMIN | 1,18E-12 | 0,26325 | 0,465 | 0,321 | 1,96E-08 |
| PRRG1 | 1,4E-12 | 0,293758 | 0,565 | 0,438 | 2,32E-08 |
| CTSD | 1,47E-12 | 0,26209 | 0,964 | 0,948 | 2,44E-08 |
| TMEM55A | 1,54E-12 | 0,253658 | 0,479 | 0,313 | 2,54E-08 |
| PARP4 | 1,56E-12 | -0,27371 | 0,435 | 0,581 | 2,59E-08 |
| PEX11B | 1,73E-12 | -0,25296 | 0,221 | 0,336 | 2,87E-08 |
| SLC25A11 | 1,84E-12 | 0,272205 | 0,654 | 0,505 | 3,04E-08 |
| CGGBP1 | 1,91E-12 | -0,27182 | 0,766 | 0,843 | 3,16E-08 |
| FNDC3A | 1,92E-12 | -0,28745 | 0,67 | 0,782 | 3,17E-08 |
| ADNP | 1,99E-12 | 0,255009 | 0,421 | 0,27 | 3,3E-08 |
| ADO | 1,99E-12 | 0,278832 | 0,46 | 0,309 | 3,3E-08 |
| ABHD12 | 2E-12 | 0,275627 | 0,84 | 0,754 | 3,31E-08 |
| PARP12 | 2,04E-12 | -0,28041 | 0,508 | 0,657 | 3,37E-08 |
| HNRNPM | 2,5E-12 | -0,27032 | 0,724 | 0,81 | 4,14E-08 |
| RBMXL1 | 2,57E-12 | -0,27951 | 0,522 | 0,631 | 4,26E-08 |
| RABL6 | 2,68E-12 | 0,254978 | 0,472 | 0,329 | 4,43E-08 |
| TANK | 2,72E-12 | -0,26517 | 0,678 | 0,759 | 4,5E-08 |
| ETNK1 | 2,8E-12 | -0,27589 | 0,555 | 0,677 | 4,64E-08 |
| SH3PXD2B | 2,86E-12 | 0,254314 | 0,351 | 0,209 | 4,73E-08 |
| LRP2 | 2,88E-12 | 0,292329 | 0,746 | 0,611 | 4,77E-08 |
| CAMK2D | 2,98E-12 | -0,25086 | 0,872 | 0,929 | 4,94E-08 |
| SLC30A7 | 3,24E-12 | -0,25555 | 0,389 | 0,545 | 5,36E-08 |
| SEC11C | 3,28E-12 | 0,293582 | 0,957 | 0,919 | 5,43E-08 |
| C5orf24 | 3,6E-12 | 0,264388 | 0,704 | 0,585 | 5,97E-08 |
| PRRX1 | 3,69E-12 | -0,31329 | 0,151 | 0,226 | 6,11E-08 |
| APOD | 3,95E-12 | 0,304376 | 0,972 | 0,96 | 6,55E-08 |
| ENPP6 | 4,6E-12 | 0,325723 | 0,519 | 0,37 | 7,61E-08 |
| SPHK1 | 4,62E-12 | -0,26025 | 0,132 | 0,26 | 7,65E-08 |
| CCL5 | 4,83E-12 | -0,50401 | 0,187 | 0,275 | 8E-08 |
| PGP | 4,97E-12 | 0,25079 | 0,567 | 0,461 | 8,23E-08 |
| MIDN | 5,28E-12 | 0,306134 | 0,704 | 0,555 | 8,74E-08 |
| CPD | 5,29E-12 | 0,276719 | 0,716 | 0,642 | 8,75E-08 |
| CELSR2 | 5,31E-12 | 0,274055 | 0,408 | 0,292 | 8,8E-08 |
| H1F0 | 5,8E-12 | 0,353427 | 0,356 | 0,229 | 9,6E-08 |
| COL9A2 | 5,84E-12 | 0,283904 | 0,387 | 0,247 | 9,68E-08 |
| GTF3A | 5,87E-12 | 0,25244 | 0,833 | 0,734 | 9,72E-08 |
| CLIC1 | 6,36E-12 | -0,32324 | 0,424 | 0,556 | 1,05E-07 |
| AHCYL1 | 6,5E-12 | -0,29678 | 0,786 | 0,848 | 1,08E-07 |
| ATG101 | 6,78E-12 | -0,25373 | 0,4 | 0,548 | 1,12E-07 |
| RAB13 | 7,48E-12 | -0,30054 | 0,288 | 0,436 | 1,24E-07 |
| ZMYM2 | 7,77E-12 | 0,281087 | 0,578 | 0,436 | 1,29E-07 |
| STX12 | 7,95E-12 | -0,25136 | 0,868 | 0,926 | 1,32E-07 |
| ATP2A2 | 8,02E-12 | 0,302335 | 0,706 | 0,611 | 1,33E-07 |
| ITGB8 | 8,47E-12 | 0,32805 | 0,724 | 0,607 | 1,4E-07 |
| PSMG2 | 8,72E-12 | -0,25036 | 0,651 | 0,765 | 1,44E-07 |
| VWA1 | 8,75E-12 | 0,299791 | 0,921 | 0,897 | 1,45E-07 |
| BCAS1 | 8,9E-12 | 0,311105 | 0,926 | 0,869 | 1,47E-07 |
| TRUB2 | 9,11E-12 | 0,250382 | 0,377 | 0,252 | 1,51E-07 |
| PICALM | 9,81E-12 | 0,259099 | 0,857 | 0,805 | 1,62E-07 |
| PLEKHA2 | 9,96E-12 | -0,27174 | 0,263 | 0,402 | 1,65E-07 |
| CBX6 | 1,08E-11 | 0,259604 | 0,543 | 0,387 | 1,79E-07 |
| C10orf90 | 1,15E-11 | 0,282489 | 0,579 | 0,427 | 1,9E-07 |
| HSPA5 | 1,24E-11 | 0,373554 | 0,786 | 0,676 | 2,05E-07 |
| SYT14 | 1,24E-11 | 0,294998 | 0,501 | 0,378 | 2,06E-07 |
| SWI5 | 1,34E-11 | 0,254497 | 0,493 | 0,352 | 2,21E-07 |
| TAOK1 | 1,36E-11 | 0,292067 | 0,782 | 0,68 | 2,25E-07 |
| CXorf38 | 1,38E-11 | -0,25508 | 0,512 | 0,647 | 2,29E-07 |
| ZBTB37 | 1,42E-11 | 0,254684 | 0,426 | 0,273 | 2,36E-07 |
| NLGN4Y | 1,5E-11 | -0,28014 | 0,436 | 0,575 | 2,48E-07 |
| TOMM70A | 1,52E-11 | -0,27426 | 0,463 | 0,598 | 2,51E-07 |
| PHACTR1 | 1,54E-11 | -0,27589 | 0,449 | 0,59 | 2,54E-07 |
| IDH1 | 1,54E-11 | 0,259311 | 0,447 | 0,296 | 2,55E-07 |
| ANAPC5 | 1,61E-11 | 0,263689 | 0,695 | 0,591 | 2,66E-07 |
| FGD5-AS1 | 1,68E-11 | 0,261321 | 0,611 | 0,492 | 2,78E-07 |
| KLHL24 | 2,13E-11 | 0,251079 | 0,378 | 0,233 | 3,52E-07 |
| NPAS3 | 2,22E-11 | 0,286353 | 0,62 | 0,482 | 3,68E-07 |
| LIPA | 2,23E-11 | 0,26846 | 0,795 | 0,699 | 3,69E-07 |
| SEMA6D | 2,33E-11 | -0,28861 | 0,211 | 0,347 | 3,85E-07 |
| AES | 2,5E-11 | 0,262758 | 0,775 | 0,662 | 4,15E-07 |
| SRFBP1 | 2,57E-11 | -0,26031 | 0,339 | 0,484 | 4,26E-07 |
| PYCR2 | 2,68E-11 | 0,254765 | 0,682 | 0,544 | 4,43E-07 |
| CBR3 | 2,77E-11 | -0,29541 | 0,301 | 0,424 | 4,58E-07 |
| USP54 | 2,77E-11 | 0,272353 | 0,651 | 0,53 | 4,59E-07 |
| SGK1 | 2,87E-11 | 0,335797 | 0,837 | 0,763 | 4,75E-07 |
| APLP1 | 2,99E-11 | 0,351107 | 0,986 | 0,98 | 4,95E-07 |
| NRXN2 | 3,03E-11 | 0,256375 | 0,506 | 0,396 | 5,01E-07 |
| PHYHIPL | 3,08E-11 | 0,356478 | 0,702 | 0,608 | 5,09E-07 |
| MAFB | 3,11E-11 | -0,28038 | 0,091 | 0,195 | 5,15E-07 |
| ZNF462 | 3,36E-11 | -0,28477 | 0,495 | 0,614 | 5,56E-07 |
| DDIT3 | 3,44E-11 | 0,436988 | 0,873 | 0,814 | 5,7E-07 |
| ATP6V1G1 | 3,46E-11 | -0,25539 | 0,966 | 0,983 | 5,73E-07 |
| LGMN | 3,98E-11 | -0,26798 | 0,294 | 0,415 | 6,58E-07 |
| MAPKAPK2 | 4,09E-11 | -0,25553 | 0,447 | 0,578 | 6,77E-07 |
| RNF187 | 4,43E-11 | 0,310284 | 0,784 | 0,667 | 7,33E-07 |
| ZNF532 | 4,72E-11 | 0,26936 | 0,538 | 0,413 | 7,82E-07 |
| GLRX2 | 4,97E-11 | 0,326062 | 0,617 | 0,492 | 8,23E-07 |
| PLIN3 | 5,06E-11 | 0,268612 | 0,504 | 0,367 | 8,38E-07 |
| ABHD2 | 5,41E-11 | 0,328131 | 0,697 | 0,593 | 8,96E-07 |
| ZNF618 | 6,46E-11 | -0,26002 | 0,283 | 0,422 | 1,07E-06 |
| ENPP2 | 6,7E-11 | 0,299549 | 0,992 | 0,991 | 1,11E-06 |
| TTL | 6,7E-11 | 0,276285 | 0,558 | 0,435 | 1,11E-06 |
| ZMYND11 | 6,8E-11 | 0,265814 | 0,575 | 0,449 | 1,13E-06 |
| YPEL2 | 7,64E-11 | 0,262183 | 0,405 | 0,275 | 1,27E-06 |
| ELL2 | 8,08E-11 | -0,31643 | 0,473 | 0,616 | 1,34E-06 |
| TNRC6C | 8,97E-11 | 0,274142 | 0,553 | 0,429 | 1,48E-06 |
| FEM1B | 1,2E-10 | 0,259591 | 0,548 | 0,422 | 1,98E-06 |
| ASPA | 1,2E-10 | 0,268604 | 0,631 | 0,525 | 2E-06 |
| RCAN1 | 1,31E-10 | 0,328066 | 0,8 | 0,685 | 2,18E-06 |
| IGSF8 | 1,34E-10 | 0,272925 | 0,857 | 0,814 | 2,23E-06 |
| FADS1 | 1,35E-10 | 0,321923 | 0,667 | 0,538 | 2,24E-06 |
| STRN | 1,41E-10 | 0,251593 | 0,481 | 0,335 | 2,34E-06 |
| MFSD12 | 1,42E-10 | 0,265078 | 0,754 | 0,664 | 2,35E-06 |
| AGPS | 1,51E-10 | 0,252264 | 0,52 | 0,387 | 2,51E-06 |
| RTFDC1 | 1,52E-10 | 0,279157 | 0,936 | 0,923 | 2,51E-06 |
| TMEM165 | 1,52E-10 | -0,25302 | 0,817 | 0,856 | 2,52E-06 |
| HERPUD1 | 1,58E-10 | 0,272215 | 0,649 | 0,504 | 2,62E-06 |
| NCAM2 | 1,59E-10 | -0,26738 | 0,638 | 0,728 | 2,64E-06 |
| EEF1D | 1,98E-10 | 0,27856 | 0,977 | 0,96 | 3,29E-06 |
| TULP4 | 2,69E-10 | 0,262877 | 0,662 | 0,542 | 4,46E-06 |
| NENF | 2,99E-10 | 0,256099 | 0,99 | 0,98 | 4,94E-06 |
| PAK2 | 3,28E-10 | 0,251269 | 0,842 | 0,767 | 5,43E-06 |
| FN1 | 3,98E-10 | -0,30939 | 0,088 | 0,183 | 6,59E-06 |
| NPC1 | 3,99E-10 | 0,252974 | 0,775 | 0,704 | 6,6E-06 |
| SCRN1 | 4,02E-10 | 0,263642 | 0,671 | 0,555 | 6,65E-06 |
| TJP2 | 4,34E-10 | -0,26303 | 0,598 | 0,71 | 7,18E-06 |
| PLLP | 4,92E-10 | 0,268296 | 0,958 | 0,942 | 8,15E-06 |
| FASN | 5,19E-10 | 0,299938 | 0,793 | 0,702 | 8,59E-06 |
| SLC25A1 | 5,79E-10 | 0,250461 | 0,571 | 0,445 | 9,59E-06 |
| RAP1A | 6,01E-10 | 0,255219 | 0,847 | 0,779 | 9,95E-06 |
| TMEM41B | 7,41E-10 | 0,257011 | 0,616 | 0,51 | 1,23E-05 |
| UNC5B | 7,51E-10 | 0,257338 | 0,843 | 0,747 | 1,24E-05 |
| ANKRD11 | 8,26E-10 | 0,268223 | 0,859 | 0,785 | 1,37E-05 |
| EGR1 | 9,13E-10 | -0,42834 | 0,139 | 0,258 | 1,51E-05 |
| MAPRE2 | 9,21E-10 | 0,251975 | 0,799 | 0,722 | 1,52E-05 |
| CACYBP | 1,05E-09 | 0,286754 | 0,764 | 0,68 | 1,74E-05 |
| PCYT1A | 1,15E-09 | -0,25903 | 0,489 | 0,607 | 1,9E-05 |
| CCDC50 | 1,21E-09 | -0,26879 | 0,62 | 0,737 | 2E-05 |
| MTSS1 | 1,25E-09 | 0,252885 | 0,559 | 0,439 | 2,06E-05 |
| C19orf60 | 1,56E-09 | 0,251828 | 0,731 | 0,624 | 2,59E-05 |
| INSIG1 | 1,87E-09 | -0,3571 | 0,82 | 0,892 | 3,09E-05 |
| CTNNB1 | 2,57E-09 | -0,26435 | 0,658 | 0,737 | 4,25E-05 |
| LSAMP | 2,61E-09 | 0,25619 | 0,626 | 0,542 | 4,32E-05 |
| SPOCD1 | 2,67E-09 | -0,4153 | 0,241 | 0,347 | 4,41E-05 |
| CALR | 2,69E-09 | 0,261699 | 0,938 | 0,908 | 4,46E-05 |
| VEGFB | 2,98E-09 | 0,266356 | 0,68 | 0,593 | 4,94E-05 |
| AMOTL2 | 3,94E-09 | 0,303501 | 0,708 | 0,676 | 6,52E-05 |
| LNPEP | 4,94E-09 | 0,259727 | 0,539 | 0,435 | 8,18E-05 |
| SLC38A2 | 5,07E-09 | 0,25778 | 0,78 | 0,665 | 8,39E-05 |
| DCXR | 5,15E-09 | 0,256698 | 0,852 | 0,783 | 8,53E-05 |
| XPOT | 5,27E-09 | 0,256924 | 0,631 | 0,518 | 8,73E-05 |
| CST3 | 6,42E-09 | -0,37115 | 0,328 | 0,436 | 0,000106 |
| SCRG1 | 7,2E-09 | 0,328303 | 0,736 | 0,628 | 0,000119 |
| FNIP2 | 8,14E-09 | -0,27853 | 0,825 | 0,889 | 0,000135 |
| PROX1 | 8,25E-09 | 0,282956 | 0,764 | 0,668 | 0,000137 |
| POU3F3 | 1,89E-08 | 0,25566 | 0,516 | 0,407 | 0,000312 |
| SHC2 | 1,9E-08 | 0,264195 | 0,514 | 0,416 | 0,000315 |
| NECAB1 | 2,2E-08 | 0,290556 | 0,692 | 0,59 | 0,000365 |
| TUBB2A | 2,54E-08 | 0,270403 | 0,772 | 0,694 | 0,00042 |
| NEAT1 | 2,87E-08 | 0,27522 | 1 | 0,998 | 0,000476 |
| IER2 | 4,26E-08 | -0,30789 | 0,256 | 0,364 | 0,000705 |
| SOX4 | 6,24E-08 | -0,28149 | 0,339 | 0,472 | 0,001033 |
| C1orf61 | 1,39E-07 | 0,287125 | 0,603 | 0,513 | 0,002308 |
| ZMAT3 | 2,88E-07 | 0,272619 | 0,762 | 0,693 | 0,004771 |
| SEMA3B | 5,19E-07 | 0,259976 | 0,79 | 0,728 | 0,008585 |
| ID4 | 6,31E-07 | -0,33748 | 0,081 | 0,161 | 0,010443 |
| UCHL1 | 2,33E-06 | 0,28854 | 0,85 | 0,802 | 0,03864 |
| HIST1H1C | 2,36E-06 | 0,553881 | 0,236 | 0,169 | 0,039021 |
| PEG10 | 3,7E-06 | 0,254184 | 0,48 | 0,376 | 0,061194 |
| MEST | 1,29E-05 | 0,279851 | 0,258 | 0,18 | 0,213802 |
| LTB | 2,76E-05 | 0,273366 | 0,306 | 0,215 | 0,457009 |
| CDKN2A | 8,04E-05 | -0,38209 | 0,134 | 0,197 | 1 |
| NPTX1 | 0,000121 | -0,33372 | 0,11 | 0,166 | 1 |
| GDF15 | 0,000289 | 0,260924 | 0,184 | 0,117 | 1 |
| SERPINE2 | 0,01142 | -0,25398 | 0,264 | 0,296 | 1 |

**Supplementary Table 2**: Differentially expressed genes (DEGs) in primary human oligodendrocytes (OLs) following 12h coculture with Th17 cells. Cluster 0 (associated with insert condition, OLs and Th17 cells separation by a porous membrane) is compared to cluster 1 (associated with contact condition, OLs and Th17 cells are in direct contact), single cell RNA sequencing. n = 1 OL prep and 1 T cell donor.

| DEGs total Th17 cells (direct contact vs. insert) | p_val | avg_log2FC | pct.1 | pct.2 | p_val_adj |
| --- | --- | --- | --- | --- | --- |
| IFNG | 0 | 3.56999937658258 | 0.851 | 0.471 | 0 |
| IL17F | 1.95716577562316e-139 | 2.9630802453529 | 0.482 | 0.072 | 3.62604103249703e-135 |
| CCL3 | 1.4668022734853e-155 | 2.32328874891478 | 0.61 | 0.211 | 2.71754457208622e-151 |
| LTA | 0 | 2.24128999559539 | 0.69 | 0.32 | 0 |
| IL17A | 1.89297012265906e-72 | 1.86299441096388 | 0.199 | 0.043 | 3.50710574625045e-68 |
| MT2A | 0 | 1.73438554840646 | 0.977 | 0.87 | 0 |
| GZMB | 4.87579024437015e-241 | 1.72897541887298 | 0.889 | 0.743 | 9.03337658574457e-237 |
| IL2RA | 0 | 1.69339489007243 | 0.98 | 0.834 | 0 |
| CCL1 | NA | 1.58388619070885 | 0.156 | 0.03 | NA |
| CCL4 | NA | 1.53302498218871 | 0.429 | 0.191 | NA |
| IL9 | NA | 1.48135686606709 | 0.12 | 0.004 | NA |
| CD52 | 0 | -1.3331504398659 | 0.924 | 0.989 | 0 |
| ZBED2 | 1.03249801776557e-229 | 1.32712763934021 | 0.654 | 0.255 | 1.91290907751427e-225 |
| FABP5 | 0 | 1.30497859742487 | 0.941 | 0.805 | 0 |
| LTB | 3.27916694511331e-132 | -1.280343351007 | 0.764 | 0.927 | 6.07531259921144e-128 |
| CSF2 | NA | 1.25198336374071 | 0.243 | 0.106 | NA |
| RARRES3 | 0 | -1.21385188658655 | 0.725 | 0.969 | 0 |
| MIR155HG | 3.7468239700175e-162 | 1.2134267002177 | 0.644 | 0.288 | 6.94174076925143e-158 |
| RGS16 | 3.81131733683461e-127 | 1.19926315627673 | 0.386 | 0.095 | 7.06122762995349e-123 |
| LMNA | 6.98679094138781e-180 | 1.18563447311366 | 0.719 | 0.454 | 1.29444275771092e-175 |
| NME1 | 0 | 1.14498717206277 | 0.828 | 0.497 | 0 |
| MX1 | 0 | 1.14092744644037 | 0.61 | 0.29 | 0 |
| IQCG | 2.97636521695693e-158 | 1.13829142561135 | 0.594 | 0.416 | 5.5143118374561e-154 |
| HSP90AB1 | 0 | 1.09221581808797 | 1 | 0.997 | 0 |
| DUSP4 | 6.44494435635561e-220 | 1.07684353971974 | 0.786 | 0.463 | 1.194054840902e-215 |
| BATF3 | 1.45749291129717e-215 | 1.07594749128037 | 0.778 | 0.533 | 2.70029711676026e-211 |
| TNFRSF4 | 3.28847223171266e-264 | 1.06761699541087 | 0.942 | 0.841 | 6.09255250369405e-260 |
| CSF1 | 2.91198666979984e-190 | 1.04268704725556 | 0.422 | 0.19 | 5.39503770313817e-186 |
| FYB | 6.08494010939573e-254 | -1.03227562651735 | 0.765 | 0.939 | 1.12735685406775e-249 |
| ARL4C | 3.89288122741053e-215 | -0.96980781845305 | 0.587 | 0.883 | 7.21234105002348e-211 |
| YPEL3 | 4.77450558076756e-268 | -0.91583816531981 | 0.438 | 0.819 | 8.84572648948806e-264 |
| TNFRSF18 | 8.29486446212382e-221 | 0.906780563527208 | 0.959 | 0.849 | 1.53678953889768e-216 |
| METRNL | 1.03648232950764e-299 | 0.903108027281385 | 0.647 | 0.284 | 1.9202908118788e-295 |
| CDK6 | 4.55776097942566e-276 | 0.899422779011057 | 0.892 | 0.68 | 8.44416376658193e-272 |
| HSPD1 | 0 | 0.896283914583282 | 0.972 | 0.911 | 0 |
| SOCS1 | 1.65101421883612e-276 | 0.893776208778583 | 0.713 | 0.366 | 3.05883404323769e-272 |
| RANBP1 | 0 | 0.875998691697416 | 0.953 | 0.837 | 0 |
| PDCD4 | 3.7294285534573e-229 | -0.864683980609287 | 0.425 | 0.751 | 6.90951228099034e-225 |
| WARS | 0 | 0.864156230812391 | 0.879 | 0.593 | 0 |
| CYCS | 0 | 0.852655229797532 | 0.955 | 0.816 | 0 |
| SRM | 1.2096151189616e-274 | 0.8493908416813 | 0.855 | 0.673 | 2.24105393090016e-270 |
| IL7R | 1.5883490824378e-34 | -0.841455240977501 | 0.229 | 0.386 | 2.94273434503251e-30 |
| HSPA5 | 7.34200318523634e-230 | 0.833618520766672 | 0.92 | 0.797 | 1.36025293012874e-225 |
| CXCL10 | 4.70051295633912e-119 | 0.819688126327499 | 0.689 | 0.27 | 8.70864035420949e-115 |
| HSP90AA1 | 0 | 0.818855421925555 | 0.999 | 1 | 0 |
| ANXA1 | 1.81174841134032e-90 | -0.817929577268454 | 0.449 | 0.675 | 3.35662628169021e-86 |
| HSPE1 | 0 | 0.808081080040775 | 0.986 | 0.961 | 0 |
| CTLA4 | 3.57924370505463e-139 | 0.80340672894791 | 0.673 | 0.487 | 6.63126481235471e-135 |
| MRTO4 | 5.98710371587024e-265 | 0.795725398195926 | 0.668 | 0.36 | 1.10923070543928e-260 |
| 06-sept | 8.87665438802037e-242 | -0.787910402965905 | 0.816 | 0.96 | 1.64457775846853e-237 |
| TUBA1B | 4.02102908136785e-154 | 0.786449573480289 | 0.926 | 0.809 | 7.44976057905021e-150 |
| KIAA1551 | 5.45804328199108e-155 | -0.783841144815139 | 0.48 | 0.743 | 1.01121167885449e-150 |
| TXNIP | 2.99270941836428e-124 | -0.777679208380193 | 0.404 | 0.661 | 5.54459273940349e-120 |
| EIF5A | 0 | 0.768621430161856 | 0.988 | 0.945 | 0 |
| LDHA | 0 | 0.76187690300684 | 1 | 0.994 | 0 |
| NEAT1 | 1.34057216984839e-69 | -0.759688044001351 | 0.961 | 0.996 | 2.48367805907812e-65 |
| HNRNPAB | 3.98715145739506e-241 | 0.756969656488596 | 0.861 | 0.687 | 7.38699550511583e-237 |
| RAN | 0 | 0.750812889857058 | 0.996 | 0.972 | 0 |
| NCL | 3.64794677387521e-221 | 0.745682002036521 | 0.982 | 0.961 | 6.75855098795861e-217 |
| CCL5 | 8.7873457334527e-31 | -0.742860076997766 | 0.629 | 0.733 | 1.62803154403678e-26 |
| CCND2 | 4.05226209347036e-137 | 0.74073484320644 | 0.66 | 0.427 | 7.50762598057254e-133 |
| ITM2B | 5.08526267111704e-179 | -0.737344242019755 | 0.771 | 0.937 | 9.42146615077853e-175 |
| SATB1 | 1.36426240507455e-183 | 0.731432047379693 | 0.912 | 0.831 | 2.52756895788162e-179 |
| CREM | 1.74301464862967e-198 | 0.731179103849117 | 0.731 | 0.474 | 3.22928323951619e-194 |
| DDX21 | 1.49507341804055e-203 | 0.724995810465766 | 0.855 | 0.688 | 2.76992252160373e-199 |
| MTHFD2 | 2.03847524178925e-255 | 0.723565594824355 | 0.811 | 0.547 | 3.77668308046295e-251 |
| AHNAK | 1.21310250891473e-129 | -0.721909972399012 | 0.758 | 0.928 | 2.24751501826632e-125 |
| NHP2 | 0 | 0.712075297521148 | 0.922 | 0.783 | 0 |
| MRPL12 | 6.75803812376104e-259 | 0.70886243955736 | 0.705 | 0.438 | 1.25206172318921e-254 |
| PPP2R5C | 2.48948026369563e-166 | -0.707686654259305 | 0.648 | 0.867 | 4.6122600845489e-162 |
| TOMM40 | 1.446163408875e-250 | 0.704321258886856 | 0.678 | 0.375 | 2.67930694762271e-246 |
| C1QBP | 1.02198872588449e-274 | 0.703860054349126 | 0.922 | 0.813 | 1.8934385124462e-270 |
| GMFG | 2.16428186340165e-216 | -0.702880019382335 | 0.962 | 0.997 | 4.00976500832423e-212 |
| PRMT1 | 1.4664371684235e-261 | 0.6994577793623 | 0.834 | 0.644 | 2.71686814193823e-257 |
| DCTPP1 | 6.85486527560193e-240 | 0.698898793783368 | 0.734 | 0.467 | 1.27000088961077e-235 |
| IL2RB | 5.12924386971082e-214 | 0.697588465545036 | 0.884 | 0.737 | 9.50295011741324e-210 |
| NOP56 | 1.47335321463699e-193 | 0.694915455096215 | 0.818 | 0.657 | 2.72968150075795e-189 |
| C12orf75 | 1.33704372400352e-122 | -0.694064757903243 | 0.83 | 0.926 | 2.47714090746132e-118 |
| EVL | 9.85438841447475e-125 | -0.69042323402254 | 0.797 | 0.954 | 1.82572254154974e-120 |
| TPI1 | 8.20136106916527e-274 | 0.690264162644902 | 0.999 | 0.992 | 1.51946616528425e-269 |
| SRSF7 | 3.69353634980682e-289 | 0.68855522853159 | 0.948 | 0.83 | 6.84301479528709e-285 |
| ZBTB32 | 3.53863641455029e-175 | 0.686603331598399 | 0.469 | 0.142 | 6.55603168523732e-171 |
| TRAF3IP3 | 6.80927608492075e-172 | -0.686437141654769 | 0.706 | 0.885 | 1.26155458025327e-167 |
| PGAM1 | 1.13985736627687e-295 | 0.682676757127379 | 0.988 | 0.924 | 2.11181374250115e-291 |
| LIMD2 | 5.55811188598139e-166 | -0.682575507345331 | 0.834 | 0.957 | 1.02975138911577e-161 |
| PPA1 | 0 | 0.677337678541763 | 0.972 | 0.895 | 0 |
| ITGA4 | 2.57343599934272e-117 | -0.67567998728769 | 0.473 | 0.727 | 4.76780487598226e-113 |
| SNRPD1 | 0 | 0.67434417544428 | 0.964 | 0.89 | 0 |
| PHB | 0 | 0.671641535637272 | 0.905 | 0.716 | 0 |
| KLF6 | 1.34383702355331e-108 | -0.671474343266582 | 0.605 | 0.833 | 2.48972685353721e-104 |
| GBP1 | 9.16086314073359e-175 | 0.664618105063221 | 0.911 | 0.745 | 1.69723311408371e-170 |
| PPP1R14B | 4.55454476225049e-206 | 0.662894215998938 | 0.895 | 0.77 | 8.43820508102148e-202 |
| PTPRC | 2.59247290061446e-136 | -0.66158858218555 | 0.955 | 0.998 | 4.80307454296841e-132 |
| SERBP1 | 3.4509002400873e-302 | 0.661567855549088 | 0.994 | 0.969 | 6.39348287480975e-298 |
| EBNA1BP2 | 1.54977796380741e-223 | 0.660575119912421 | 0.676 | 0.415 | 2.87127363354598e-219 |
| HNRNPDL | 4.62758340307679e-253 | 0.657060645859205 | 0.915 | 0.776 | 8.57352377088037e-249 |
| TUBB | 1.13144149462521e-144 | 0.654566145132182 | 0.884 | 0.754 | 2.09622165709213e-140 |
| SH2D2A | 1.40920190788504e-186 | 0.651500451274265 | 0.897 | 0.7 | 2.61082837473861e-182 |
| TBX21 | 8.07614629617395e-171 | 0.650237390779267 | 0.48 | 0.22 | 1.49626762429215e-166 |
| RPS17 | 0 | 0.648057185014669 | 0.999 | 0.997 | 0 |
| GPATCH4 | 2.20740207351328e-187 | 0.647602814773499 | 0.681 | 0.419 | 4.08965382159805e-183 |
| TNF | 1.63134987133232e-53 | 0.647288528874008 | 0.452 | 0.351 | 3.0224019066174e-49 |
| SNHG15 | 8.16296128042675e-165 | 0.639931591994103 | 0.688 | 0.445 | 1.51235183642466e-160 |
| PNRC1 | 1.47255015597463e-134 | -0.638580365529795 | 0.645 | 0.875 | 2.72819367397421e-130 |
| MT-ND5 | 8.90701603899672e-165 | 0.636941205871023 | 0.997 | 0.998 | 1.65020286154492e-160 |
| P2RX5 | 6.5140545595472e-134 | 0.636494967305118 | 0.698 | 0.541 | 1.20685888824731e-129 |
| ISG15 | 1.27466159472638e-160 | 0.636222237021166 | 0.941 | 0.924 | 2.36156553654957e-156 |
| EED | 8.67182137068837e-162 | 0.633685671545336 | 0.646 | 0.45 | 1.60662834534743e-157 |
| RUNX3 | 5.57707451840249e-153 | 0.629352349138209 | 0.873 | 0.744 | 1.03326459602443e-148 |
| ADAM19 | 5.43646997003249e-155 | 0.628621715315151 | 0.666 | 0.421 | 1.00721479134792e-150 |
| TC2N | 8.26871202021612e-127 | -0.627412961097237 | 0.118 | 0.433 | 1.53194427598544e-122 |
| IFITM2 | 4.45441965672384e-101 | -0.627153952184017 | 0.769 | 0.901 | 8.25270329801225e-97 |
| CCT2 | 7.91894493248531e-236 | 0.626519746673927 | 0.916 | 0.802 | 1.46714292764155e-231 |
| ICOS | 1.24909981567266e-134 | 0.622784721533587 | 0.603 | 0.417 | 2.31420722849674e-130 |
| NPM1 | 0 | 0.619523157338548 | 1 | 0.999 | 0 |
| PAICS | 1.05183316034582e-179 | 0.619398029660002 | 0.594 | 0.309 | 1.9487312961727e-175 |
| EVI2B | 1.01605541103621e-149 | -0.614298127659576 | 0.252 | 0.582 | 1.88244586002678e-145 |
| MT1X | 4.24539421102646e-143 | 0.611067024763185 | 0.659 | 0.452 | 7.86544185476873e-139 |
| RPL22L1 | 1.40358966413182e-164 | 0.609285084175653 | 0.908 | 0.808 | 2.60043057073702e-160 |
| CMSS1 | 2.55386123009743e-186 | 0.607985756192595 | 0.542 | 0.248 | 4.73153870100151e-182 |
| PTMA | 1.00377318948943e-280 | 0.600421739032755 | 1 | 1 | 1.85969058816707e-276 |
| CCT3 | 8.56684668095897e-259 | 0.598438261205299 | 0.979 | 0.932 | 1.58717968458127e-254 |
| CCT5 | 8.71429930317948e-234 | 0.59747546790015 | 0.933 | 0.823 | 1.61449823190006e-229 |
| CD99 | 1.94436881936898e-125 | -0.597184412686291 | 0.853 | 0.959 | 3.60233211164491e-121 |
| EPAS1 | 7.51288910739866e-102 | 0.597080402453355 | 0.383 | 0.194 | 1.39191296492775e-97 |
| HSPH1 | 2.03379323227562e-140 | 0.596272985087497 | 0.805 | 0.598 | 3.76800872143704e-136 |
| FAM65B | 9.17165123916755e-134 | -0.595193317347646 | 0.273 | 0.569 | 1.69923182508057e-129 |
| CUTA | 1.25310640034378e-132 | -0.594667503844373 | 0.788 | 0.924 | 2.32163022791692e-128 |
| NOP14 | 8.98344012897946e-144 | 0.59447869749558 | 0.691 | 0.483 | 1.66436195269603e-139 |
| ZFP36L2 | 2.21097318173103e-77 | -0.594003566316221 | 0.484 | 0.696 | 4.09627001379308e-73 |
| TUBA1C | 1.02538686588276e-150 | 0.592100362710223 | 0.679 | 0.431 | 1.89973424642099e-146 |
| ALOX5AP | 6.67279162105516e-61 | -0.591209024324032 | 0.757 | 0.88 | 1.23626810363289e-56 |
| TIMM13 | 1.28604350929025e-213 | 0.590222274622825 | 0.829 | 0.642 | 2.38265280966205e-209 |
| SELL | 5.4138089829518e-47 | -0.590121479331745 | 0.469 | 0.576 | 1.00301639027148e-42 |
| MT1E | 1.06349506335189e-62 | 0.589467308521328 | 0.398 | 0.289 | 1.97033730387205e-58 |
| CD320 | 4.9809708629938e-168 | 0.588530933275159 | 0.666 | 0.416 | 9.22824471786861e-164 |
| CD3G | 1.2143787341206e-120 | -0.586052718819724 | 0.893 | 0.96 | 2.24987948070523e-116 |
| TRBC2 | 9.70441857731325e-72 | -0.585807346504011 | 0.96 | 0.988 | 1.79793762981883e-67 |
| RBM8A | 1.1411587322299e-268 | 0.585476787734405 | 0.98 | 0.919 | 2.11422478320233e-264 |
| CCT6A | 2.74785450967831e-213 | 0.584654330258171 | 0.936 | 0.828 | 5.090950050081e-209 |
| PSME2 | 0 | 0.580462378824191 | 0.998 | 0.989 | 0 |
| PYCARD | 2.97807408938805e-97 | -0.579998021061793 | 0.604 | 0.826 | 5.51747786540924e-93 |
| SOX4 | 1.1632551652879e-26 | 0.579909897751849 | 0.3 | 0.198 | 2.15516284472889e-22 |
| DKC1 | 1.24732774625224e-160 | 0.579897011880211 | 0.684 | 0.482 | 2.31092411548152e-156 |
| TNFRSF25 | 1.12058751320203e-123 | 0.578990656021567 | 0.717 | 0.52 | 2.07611248570941e-119 |
| SLIRP | 1.22558184484739e-253 | 0.576803806067258 | 0.951 | 0.871 | 2.27063548394876e-249 |
| ODC1 | 4.75122247705005e-139 | 0.575298704789917 | 0.488 | 0.239 | 8.80258988323063e-135 |
| BTG1 | 9.5105309861776e-74 | -0.571385948853455 | 0.862 | 0.969 | 1.76201607580912e-69 |
| PA2G4 | 2.34466198065277e-231 | 0.57041494215967 | 0.978 | 0.951 | 4.34395525155539e-227 |
| ARID5A | 7.92223029446966e-91 | 0.569258828434451 | 0.634 | 0.431 | 1.46775160665639e-86 |
| SLC27A2 | 1.45463632545577e-147 | 0.568983593585855 | 0.7 | 0.46 | 2.6950047201719e-143 |
| BZW1 | 7.15670125929484e-185 | 0.567394726880481 | 0.962 | 0.867 | 1.32592204230956e-180 |
| PRDX1 | 3.38472288764567e-255 | 0.566031885726241 | 0.998 | 0.994 | 6.27087609394113e-251 |
| SMS | 3.70317951608062e-142 | 0.564951181462497 | 0.71 | 0.53 | 6.86088068944257e-138 |
| IL32 | 1.48821227356158e-93 | -0.562230339366841 | 0.998 | 1 | 2.75721087922753e-89 |
| LCK | 1.07232350523638e-123 | -0.559103422193157 | 0.836 | 0.942 | 1.98669375815144e-119 |
| S100A4 | 6.91346845202397e-102 | -0.558658966167912 | 0.998 | 1 | 1.28085830010648e-97 |
| TNFAIP8 | 7.05174454259379e-111 | 0.558305069330061 | 0.949 | 0.891 | 1.30647671140635e-106 |
| ATP1B3 | 4.4601595338358e-184 | 0.554243962394985 | 0.896 | 0.774 | 8.26333756833759e-180 |
| SOD2 | 4.10737790922309e-189 | 0.551723370041476 | 0.68 | 0.389 | 7.60973905241762e-185 |
| FURIN | 8.12615789767908e-134 | 0.551350735862339 | 0.882 | 0.78 | 1.505533273703e-129 |
| ARHGDIB | 3.22922689790929e-143 | -0.551076342683797 | 0.985 | 0.998 | 5.98278867375655e-139 |
| PMAIP1 | 2.68887557887321e-99 | 0.548936498717967 | 0.475 | 0.252 | 4.98167978497839e-95 |
| TXNDC17 | 1.02173809092522e-198 | 0.547946294608023 | 0.958 | 0.883 | 1.89297416105716e-194 |
| NPM3 | 6.10838336702779e-145 | 0.547477159602918 | 0.461 | 0.168 | 1.13170018640924e-140 |
| SLC3A2 | 5.23374709539895e-116 | 0.546614478829346 | 0.784 | 0.628 | 9.69656324364564e-112 |
| RPP30 | 6.97363459351633e-159 | 0.545492804099553 | 0.667 | 0.448 | 1.29200528114077e-154 |
| SNRPE | 2.66392277145073e-235 | 0.543074128050014 | 0.961 | 0.906 | 4.93544971866677e-231 |
| IFI44L | 1.7521239849893e-26 | 0.541845822648425 | 0.225 | 0.031 | 3.24616010698967e-22 |
| CD27 | 7.9449038911197e-47 | -0.540849746900385 | 0.229 | 0.407 | 1.47195234390775e-42 |
| LAP3 | 1.16034466323707e-180 | 0.540617634197879 | 0.814 | 0.612 | 2.14977055757931e-176 |
| ATM | 2.70792078260894e-112 | -0.537938574569568 | 0.324 | 0.597 | 5.01696483393958e-108 |
| POLR2H | 1.11907630149343e-198 | 0.537730673993992 | 0.839 | 0.662 | 2.07331266377688e-194 |
| RCSD1 | 1.91836238994726e-96 | -0.536301736921599 | 0.604 | 0.797 | 3.55414999985529e-92 |
| ASB2 | 1.57515786896996e-76 | -0.53550563296021 | 0.287 | 0.539 | 2.91829498384064e-72 |
| HSPA8 | 1.8679593042728e-206 | 0.535213603876707 | 0.999 | 0.999 | 3.46076820302621e-202 |
| CYTIP | 6.80647878356144e-123 | 0.535077623438627 | 0.943 | 0.932 | 1.26103632423043e-118 |
| CRIP1 | 1.65530534422118e-77 | -0.533698060303615 | 0.896 | 0.961 | 3.06678421123859e-73 |
| PFDN2 | 1.30136908596851e-201 | 0.533559799033797 | 0.949 | 0.857 | 2.41104650557385e-197 |
| SDF2L1 | 1.30690274118235e-134 | 0.533143130751282 | 0.796 | 0.629 | 2.42129870858854e-130 |
| SURF4 | 2.53885271626868e-174 | 0.532020011089233 | 0.855 | 0.659 | 4.70373242743099e-170 |
| TUBB4B | 1.96093959117541e-110 | 0.530420628914425 | 0.773 | 0.585 | 3.63303278057068e-106 |
| PDCD5 | 1.77288326095971e-192 | 0.526012758721372 | 0.898 | 0.806 | 3.28462081758005e-188 |
| RPS15A | 0 | -0.525777235268986 | 1 | 1 | 0 |
| EIF4G1 | 6.87015388120274e-155 | 0.525222042425769 | 0.728 | 0.509 | 1.27283340957043e-150 |
| NOP16 | 9.55026392903026e-148 | 0.52382992357531 | 0.497 | 0.241 | 1.76937739813144e-143 |
| FKBP4 | 2.30428183612754e-153 | 0.523732102781239 | 0.557 | 0.308 | 4.2691429577935e-149 |
| TSC22D3 | 6.47011049700258e-88 | -0.521065686450241 | 0.198 | 0.44 | 1.19871737177967e-83 |
| H1FX | 4.76548233873601e-72 | -0.520941456347554 | 0.556 | 0.727 | 8.8290091289762e-68 |
| SRSF3 | 1.60354059191887e-228 | 0.520827291857267 | 0.988 | 0.956 | 2.97087965464809e-224 |
| UBE2S | 1.00334457369786e-121 | 0.520291317490603 | 0.808 | 0.602 | 1.85889649169002e-117 |
| ANP32E | 6.15754015809098e-130 | 0.51740927485049 | 0.887 | 0.741 | 1.14080746508952e-125 |
| CXCL8 | NA | 0.517054880382093 | 0.187 | 0.044 | NA |
| DNAJA1 | 2.54669060402922e-174 | 0.516432387648203 | 0.989 | 0.948 | 4.71825368208494e-170 |
| TNFRSF9 | 4.27749665733452e-54 | 0.513890122103551 | 0.339 | 0.168 | 7.92491805704367e-50 |
| TRBC1 | 9.66490342772159e-24 | -0.513280215773833 | 0.736 | 0.782 | 1.79061665805398e-19 |
| PCNA | 7.0063031732784e-76 | 0.512476327205859 | 0.596 | 0.45 | 1.29805778891329e-71 |
| EIF2AK2 | 1.13568334655127e-134 | 0.511404604171748 | 0.65 | 0.478 | 2.10408053615553e-130 |
| APRT | 4.00540592039857e-263 | 0.511403832048836 | 0.977 | 0.948 | 7.42081554872244e-259 |
| SLC7A5 | 8.48589828820106e-128 | 0.511311879174347 | 0.476 | 0.218 | 1.57218237585501e-123 |
| PGK1 | 4.40821289168814e-190 | 0.511095733370951 | 0.983 | 0.961 | 8.16709602443062e-186 |
| CDC42SE2 | 2.21584097844828e-96 | -0.510789885730171 | 0.843 | 0.937 | 4.10528858077113e-92 |
| CCL20 | 1.47312045291963e-15 | 0.510326696609248 | 0.196 | 0.105 | 2.72925026312421e-11 |
| UQCC2 | 5.52784992525663e-178 | 0.510315352492875 | 0.83 | 0.65 | 1.0241447556523e-173 |
| MYDGF | 5.64230461603824e-169 | 0.508154089885725 | 0.855 | 0.674 | 1.04534977621341e-164 |
| GLA | 2.71993457261283e-85 | 0.506172502792495 | 0.464 | 0.31 | 5.03922278267979e-81 |
| S1PR4 | 5.25425756310846e-74 | -0.505996806873198 | 0.663 | 0.813 | 9.73456298717103e-70 |
| NAP1L4 | 2.08602335822021e-71 | -0.505409405698078 | 0.833 | 0.899 | 3.86477547577459e-67 |
| VDAC1 | 4.70658817934322e-205 | 0.504294040791613 | 0.943 | 0.865 | 8.71989591986918e-201 |
| TXN | 8.95359511229598e-162 | 0.50402736328476 | 0.999 | 0.996 | 1.65883256645508e-157 |
| SLFN5 | 3.21522800137943e-56 | -0.503736734566807 | 0.649 | 0.818 | 5.95685291815567e-52 |
| NOLC1 | 9.0592725009403e-132 | 0.50323550365498 | 0.535 | 0.287 | 1.67841141624921e-127 |
| SQSTM1 | 1.09477597598243e-67 | -0.502954327032199 | 0.837 | 0.915 | 2.02829145070265e-63 |
| GNL3 | 9.86547397544389e-135 | 0.502909861729858 | 0.633 | 0.434 | 1.82777636343049e-130 |
| IFI35 | 1.27106478028557e-149 | 0.502092796524854 | 0.742 | 0.558 | 2.35490171843508e-145 |
| MT-ND4L | 1.77316128328315e-98 | 0.500628897419952 | 0.889 | 0.851 | 3.2851359095387e-94 |
| WDR43 | 2.60716108419501e-110 | 0.499904207881588 | 0.603 | 0.413 | 4.83028734068809e-106 |
| MYO1F | 5.14761233094829e-87 | -0.499403217972267 | 0.359 | 0.608 | 9.5369813655479e-83 |
| ENO1 | 1.22860893385006e-191 | 0.497949776979359 | 1 | 1 | 2.27624377174401e-187 |
| BATF | 8.53400735903691e-104 | 0.495425279768595 | 0.957 | 0.926 | 1.58109554340877e-99 |
| EIF1AX | 9.50018236054456e-156 | 0.495220916860023 | 0.846 | 0.689 | 1.76009878593809e-151 |
| PKM | 1.88559187525208e-169 | 0.494747061118013 | 1 | 0.998 | 3.49343606727953e-165 |
| PRDX4 | 3.83723437478525e-151 | 0.493639225265597 | 0.777 | 0.579 | 7.10924412616463e-147 |
| RP11-138A9.2 | 1.00408539119082e-82 | 0.493042517858835 | 0.617 | 0.492 | 1.86026900425923e-78 |
| GPSM3 | 9.45409431194879e-97 | -0.492879338147126 | 0.771 | 0.919 | 1.75156005317475e-92 |
| SARAF | 1.06509233282284e-90 | -0.492819978418674 | 0.782 | 0.903 | 1.97329656502087e-86 |
| APBB1IP | 1.65106751591933e-90 | -0.491370643046396 | 0.668 | 0.849 | 3.05893278674374e-86 |
| IFI6 | 2.27338585259157e-106 | 0.491011609941753 | 0.611 | 0.478 | 4.2119019690964e-102 |
| IRF7 | 8.55905867071322e-135 | 0.490962935882821 | 0.46 | 0.232 | 1.58573679992304e-130 |
| PBXIP1 | 1.87815044899779e-83 | -0.490816095298108 | 0.305 | 0.566 | 3.47964933685821e-79 |
| PLIN2 | 1.92966654107343e-62 | -0.485675489439366 | 0.689 | 0.772 | 3.57509320064674e-58 |
| CALR | 1.38483959177509e-128 | 0.485109581823739 | 0.982 | 0.971 | 2.56569231168172e-124 |
| TNFRSF8 | 1.66781877233332e-60 | 0.484901146269484 | 0.334 | 0.089 | 3.08996783950194e-56 |
| POLD2 | 1.02631512671715e-148 | 0.484538968699608 | 0.58 | 0.341 | 1.90145403526887e-144 |
| GPR171 | 1.98326018971097e-57 | -0.483698858400786 | 0.811 | 0.906 | 3.67438615347751e-53 |
| STIP1 | 3.44699080400575e-140 | 0.483103830603712 | 0.845 | 0.681 | 6.38623986258146e-136 |
| MIF | 7.28788934394996e-228 | 0.480923305572888 | 0.999 | 0.995 | 1.35022725875361e-223 |
| ZNF593 | 1.46475485008315e-134 | 0.480112710884856 | 0.647 | 0.459 | 2.71375131074905e-130 |
| NIFK | 3.49965103876955e-123 | 0.478718629416627 | 0.815 | 0.691 | 6.48380347952835e-119 |
| H3F3B | 5.29538406035325e-213 | 0.47853789375475 | 1 | 1 | 9.81075804861647e-209 |
| CCT7 | 1.98469127944362e-174 | 0.47789925798894 | 0.894 | 0.765 | 3.67703753342519e-170 |
| SNRPB | 2.78943921324692e-224 | 0.47740370566763 | 0.988 | 0.964 | 5.16799403038258e-220 |
| LYRM4 | 2.94252545638232e-139 | 0.47597841614505 | 0.612 | 0.39 | 5.45161691303952e-135 |
| CTSL | 3.59295366838591e-64 | 0.475640440334996 | 0.311 | 0.162 | 6.65666526141857e-60 |
| YBX1 | 5.19926477549006e-230 | 0.474703014062318 | 1 | 0.998 | 9.63267784955044e-226 |
| KCNN4 | 3.49639422843911e-140 | 0.473973353669228 | 0.616 | 0.362 | 6.47776958702913e-136 |
| CLU | 1.11681889790154e-44 | -0.47373356831517 | 0.457 | 0.607 | 2.06913037214218e-40 |
| MGST3 | 5.38416859560918e-70 | -0.473333261404187 | 0.616 | 0.794 | 9.97524915708513e-66 |
| IMPDH2 | 6.88694486657084e-133 | 0.473247128576494 | 0.641 | 0.435 | 1.27594427542958e-128 |
| MANF | 5.18138062010147e-113 | 0.472504652247858 | 0.854 | 0.713 | 9.59954387486199e-109 |
| CTSH | 9.78035179891869e-49 | 0.471916103961788 | 0.677 | 0.597 | 1.81200577778567e-44 |
| MCM7 | 1.67563626811179e-63 | 0.470571221835093 | 0.347 | 0.171 | 3.10445131393071e-59 |
| LIMS1 | 1.22209312830697e-58 | -0.469621471043788 | 0.686 | 0.805 | 2.26417193881432e-54 |
| CCT4 | 1.26306041858686e-155 | 0.469469353417976 | 0.893 | 0.764 | 2.34007203751588e-151 |
| IFI27L2 | 6.18127150434559e-87 | -0.467876934322748 | 0.809 | 0.925 | 1.14520417161011e-82 |
| CYLD | 6.42812740460621e-62 | -0.465990684413888 | 0.651 | 0.808 | 1.19093916425139e-57 |
| MRPS26 | 8.19235607386829e-132 | 0.465392567885563 | 0.674 | 0.496 | 1.51779780980558e-127 |
| TYMS | 2.36169324090077e-35 | 0.465368597408837 | 0.3 | 0.122 | 4.37550906741686e-31 |
| GBP5 | 3.48154740599104e-27 | -0.464855486802531 | 0.759 | 0.891 | 6.4502628790796e-23 |
| IER3 | 9.44906786355884e-30 | 0.464699305251919 | 0.476 | 0.327 | 1.75062880308155e-25 |
| ANKRD28 | 5.17115777736534e-44 | -0.463551734709088 | 0.479 | 0.659 | 9.58060401412476e-40 |
| SYNE2 | 1.31377255449041e-46 | -0.462855460678232 | 0.596 | 0.757 | 2.43402641170439e-42 |
| RPL23 | 5.06929727891998e-256 | 0.462512524049477 | 1 | 0.999 | 9.39188706865504e-252 |
| HSPA9 | 4.87612947506609e-134 | 0.46160119619706 | 0.723 | 0.535 | 9.03400507845494e-130 |
| TMSB4X | 1.15989812110386e-98 | -0.461584993859748 | 1 | 1 | 2.14894324896912e-94 |
| ELF1 | 9.10819572245638e-77 | -0.460627031593008 | 0.717 | 0.849 | 1.68747542149949e-72 |
| TFRC | 8.43824786602196e-74 | 0.459355558854813 | 0.622 | 0.463 | 1.56335418213789e-69 |
| IFIT3 | 1.82556603778081e-81 | 0.457927545171049 | 0.4 | 0.143 | 3.3822261981965e-77 |
| ACAP1 | 4.5006208787606e-66 | -0.457450549051194 | 0.822 | 0.927 | 8.33830030207976e-62 |
| TCP1 | 3.91717929499066e-134 | 0.456638640578091 | 0.818 | 0.665 | 7.2573580798292e-130 |
| SRSF5 | 1.50841895038636e-81 | -0.456495397445333 | 0.884 | 0.959 | 2.79464778938082e-77 |
| GINS2 | 1.05103464475386e-31 | 0.455631608836622 | 0.251 | 0.085 | 1.94725188633548e-27 |
| GZMK | 6.08982749960985e-13 | -0.454870156590876 | 0.072 | 0.124 | 1.12826234085272e-08 |
| S100A6 | 1.5452580260677e-54 | -0.454554183131286 | 0.999 | 1 | 2.86289954489562e-50 |
| TOMM7 | 1.7860135545173e-130 | -0.453600151022619 | 0.981 | 0.995 | 3.3089473124542e-126 |
| JAK1 | 2.31308293266539e-72 | -0.452307587508904 | 0.835 | 0.924 | 4.28544874934917e-68 |
| GIMAP7 | 8.72975059481754e-40 | -0.450522648809846 | 0.778 | 0.871 | 1.61736089270184e-35 |
| RNF213 | 8.93549389422616e-97 | 0.449525011509557 | 0.993 | 0.994 | 1.65547895378328e-92 |
| CCT8 | 9.66895973693357e-161 | 0.449100143535255 | 0.925 | 0.84 | 1.79136817046168e-156 |
| COMMD6 | 5.49607645665727e-117 | -0.449090977498126 | 0.964 | 0.992 | 1.01825808512489e-112 |
| RBM3 | 7.93439232715442e-182 | 0.447984765207198 | 0.973 | 0.925 | 1.4700048664519e-177 |
| HAPLN3 | 2.42678020562716e-105 | 0.447977029763598 | 0.439 | 0.18 | 4.49609568696544e-101 |
| ANK3 | 2.1519327234885e-67 | 0.447693647798782 | 0.472 | 0.331 | 3.98688575680715e-63 |
| RSL1D1 | 3.93520437147768e-139 | 0.447249708593901 | 0.872 | 0.765 | 7.29075313903669e-135 |
| PIM3 | 9.50964861743055e-59 | 0.446017388095196 | 0.53 | 0.394 | 1.76185259935136e-54 |
| KLRB1 | 1.14711138012846e-21 | -0.445650721716104 | 0.11 | 0.212 | 2.12525325396399e-17 |
| SYTL3 | 7.56727662143849e-94 | 0.44483534553391 | 0.614 | 0.444 | 1.40198933965391e-89 |
| BOP1 | 1.39798575957876e-118 | 0.443674497598174 | 0.464 | 0.217 | 2.59004821677156e-114 |
| BOLA3 | 5.51950126931497e-138 | 0.442720156304468 | 0.739 | 0.531 | 1.02259800016598e-133 |
| NUCB2 | 1.0244847709027e-29 | -0.442177912922634 | 0.426 | 0.578 | 1.89806293505144e-25 |
| GLUL | 5.74565336325973e-102 | 0.441886282927792 | 0.604 | 0.4 | 1.06449719861113e-97 |
| IRF4 | 1.04344034403145e-55 | 0.439980617252486 | 0.54 | 0.371 | 1.93318192538706e-51 |
| GADD45GIP1 | 3.23041678470832e-166 | 0.439048433419815 | 0.953 | 0.886 | 5.9849931770291e-162 |
| SELPLG | 6.20504354717308e-59 | -0.437862720419506 | 0.423 | 0.644 | 1.14960841798476e-54 |
| SH3BGRL | 3.8492115080527e-77 | -0.437679580396527 | 0.601 | 0.783 | 7.13143416096923e-73 |
| IFRD2 | 5.49517451844436e-112 | 0.437336727852219 | 0.451 | 0.215 | 1.01809098303219e-107 |
| SKAP1 | 8.60642871424774e-73 | -0.437006426618487 | 0.535 | 0.711 | 1.59451304788868e-68 |
| CYBA | 2.24139410810512e-65 | -0.435368525536405 | 0.979 | 0.997 | 4.15263086408636e-61 |
| RABAC1 | 4.96610652146526e-66 | -0.435318834851145 | 0.756 | 0.888 | 9.20070555231869e-62 |
| CDKN1A | 2.45054430955096e-76 | 0.432570428393529 | 0.414 | 0.208 | 4.54012344230507e-72 |
| RP11-138A9.1 | 4.51438853967067e-80 | 0.431244652389269 | 0.489 | 0.349 | 8.36380764744784e-76 |
| RBL2 | 1.32401500409901e-83 | -0.431180210423601 | 0.281 | 0.502 | 2.45300259809423e-79 |
| SSBP1 | 9.16440718408083e-188 | 0.431148429225732 | 0.983 | 0.961 | 1.69788971899466e-183 |
| TRAC | 2.54341019524006e-43 | -0.430815456859432 | 0.948 | 0.977 | 4.71217606872125e-39 |
| TPT1 | 1.72727842776695e-177 | -0.430798264404908 | 1 | 1 | 3.20012874312383e-173 |
| RAB37 | 2.43660463932886e-43 | -0.430625814714924 | 0.096 | 0.324 | 4.51429741528458e-39 |
| ARPC5L | 2.28467201506841e-125 | 0.429031541294362 | 0.897 | 0.79 | 4.23281184231725e-121 |
| IMP4 | 3.60064129608197e-122 | 0.428992029893622 | 0.542 | 0.319 | 6.67090812925106e-118 |
| EIF3B | 3.08637775810651e-114 | 0.428899765875235 | 0.668 | 0.471 | 5.71813207244393e-110 |
| GTPBP4 | 1.43108942132044e-99 | 0.428719553833519 | 0.734 | 0.564 | 2.65137937088038e-95 |
| CAST | 7.21094085229788e-59 | -0.428714154138456 | 0.864 | 0.949 | 1.33597101170523e-54 |
| MALAT1 | 1.24252940120403e-22 | -0.428350032494069 | 1 | 1 | 2.30203422161071e-18 |
| PUM3 | 1.26591265881775e-113 | 0.427910092715371 | 0.523 | 0.305 | 2.34535638299165e-109 |
| NDUFAF2 | 8.74118659654474e-101 | 0.427865270791434 | 0.613 | 0.407 | 1.61947964074184e-96 |
| MRPL52 | 7.10390290978021e-153 | 0.427302897647103 | 0.945 | 0.871 | 1.31614009209498e-148 |
| MRPL3 | 1.32574712733353e-120 | 0.427186291421111 | 0.645 | 0.458 | 2.45621170281083e-116 |
| RSRP1 | 8.13909095680348e-62 | -0.426850913180891 | 0.514 | 0.687 | 1.50792938156698e-57 |
| MRPL4 | 1.5240479139576e-126 | 0.426657173715674 | 0.769 | 0.604 | 2.82360357018924e-122 |
| HMGB1 | 6.02012716591507e-88 | -0.426093626703854 | 1 | 1 | 1.11534896002909e-83 |
| SYNCRIP | 2.03313215034609e-115 | 0.425942267925822 | 0.848 | 0.704 | 3.76678393494621e-111 |
| PDIA6 | 8.56435957908974e-126 | 0.425923389912736 | 0.941 | 0.886 | 1.58671889921796e-121 |
| SLC9A3R1 | 8.90935832025888e-64 | -0.425740347792293 | 0.632 | 0.751 | 1.65063681599436e-59 |
| KIF2A | 2.6133032768538e-98 | 0.42390401970569 | 0.835 | 0.733 | 4.84166698102704e-94 |
| SLC1A5 | 3.12741936072951e-90 | 0.422652654624429 | 0.477 | 0.282 | 5.79416984962357e-86 |
| DCUN1D5 | 2.33861593691327e-107 | 0.421786242087738 | 0.608 | 0.436 | 4.33275374631922e-103 |
| PNISR | 3.54308688921052e-62 | -0.420690392924304 | 0.882 | 0.939 | 6.56427707964032e-58 |
| JUNB | 8.07039061615037e-79 | 0.420356984279531 | 0.939 | 0.881 | 1.49520126945418e-74 |
| IL4I1 | 1.8808002050124e-44 | 0.420242611374994 | 0.296 | 0.115 | 3.48455853982647e-40 |
| CD37 | 6.21129651515562e-70 | -0.420219922069567 | 0.708 | 0.855 | 1.15076690536288e-65 |
| CHCHD2 | 3.50676293395518e-280 | 0.420147668540869 | 1 | 0.999 | 6.49697968773877e-276 |
| PTPN22 | 3.52549589080105e-60 | -0.420047194054583 | 0.384 | 0.593 | 6.53168623688711e-56 |
| TYMP | 5.25845512423213e-103 | 0.419916118571774 | 0.899 | 0.848 | 9.74233980866487e-99 |
| IL12RB2 | 8.63883681905653e-94 | 0.419345680526591 | 0.411 | 0.202 | 1.6005172974666e-89 |
| ITGAL | 1.52232200790269e-44 | -0.419129108406022 | 0.75 | 0.869 | 2.82040598404132e-40 |
| AHR | 1.08380667632047e-70 | 0.41891633040826 | 0.529 | 0.372 | 2.00796862921893e-66 |
| VDR | 6.65539348773047e-100 | 0.418078586590517 | 0.486 | 0.273 | 1.23304475147182e-95 |
| BRIX1 | 6.51616969013296e-108 | 0.417825128988041 | 0.598 | 0.425 | 1.20725075849093e-103 |
| C19orf24 | 1.84152548847449e-117 | 0.417270830873391 | 0.751 | 0.576 | 3.41179427249669e-113 |
| PSMD14 | 6.03839807498386e-124 | 0.415813707209 | 0.8 | 0.628 | 1.11873401135226e-119 |
| UQCRB | 7.11125703364584e-99 | -0.414628205736833 | 0.987 | 0.998 | 1.31750259062357e-94 |
| ZNF282 | 1.44392781835911e-73 | 0.414548227429294 | 0.371 | 0.153 | 2.67516506907393e-69 |
| NAMPT | 2.77331621695638e-60 | 0.413779550038462 | 0.656 | 0.5 | 5.13812295515509e-56 |
| MTRNR2L8 | 3.1662665807831e-66 | 0.413580041023881 | 0.432 | 0.328 | 5.86614209421685e-62 |
| EIF4A1 | 1.39892993541073e-105 | 0.413573688018675 | 0.639 | 0.458 | 2.59179749133546e-101 |
| HMGA1 | 6.01366253216778e-85 | 0.413309169251549 | 0.527 | 0.369 | 1.11415125733473e-80 |
| ERP29 | 5.46746841090008e-63 | -0.413039689327161 | 0.659 | 0.792 | 1.01295787248746e-58 |
| TSTD1 | 1.53501978370966e-67 | -0.412671044107436 | 0.502 | 0.689 | 2.84393115327889e-63 |
| H2AFZ | 3.62594246398467e-84 | 0.41223036837859 | 0.983 | 0.971 | 6.71778360302439e-80 |
| GIMAP1 | 8.33150344316985e-61 | -0.411577984371151 | 0.359 | 0.548 | 1.54357764291608e-56 |
| SET | 4.67315944133529e-146 | 0.411528653334138 | 0.992 | 0.984 | 8.65796249696189e-142 |
| RPL7L1 | 2.3608177244575e-112 | 0.41119072926841 | 0.76 | 0.6 | 4.37388699810242e-108 |
| C15orf48 | 1.1012201695192e-21 | 0.410861087060126 | 0.209 | 0.075 | 2.04023060806823e-17 |
| PRF1 | 3.06368935674509e-19 | 0.410720844682396 | 0.35 | 0.292 | 5.67609727124164e-15 |
| SYNE1 | 6.62356418190773e-71 | -0.410697187275369 | 0.222 | 0.443 | 1.22714773598205e-66 |
| ILF2 | 4.87136384404535e-117 | 0.410367088621934 | 0.795 | 0.64 | 9.02517579386283e-113 |
| CCDC6 | 1.18747845666611e-92 | 0.409873213925281 | 0.593 | 0.399 | 2.20004133666531e-88 |
| TNRC6B | 3.03460902781055e-55 | -0.409605447332936 | 0.514 | 0.695 | 5.62222014582461e-51 |
| PSMA3 | 1.67010413553265e-141 | 0.409120126838225 | 0.923 | 0.826 | 3.09420193190134e-137 |
| KLF2 | 6.57846276402103e-17 | -0.408109822123905 | 0.08 | 0.17 | 1.21879179629018e-12 |
| SLC38A5 | 1.32361220096342e-82 | 0.406220165410516 | 0.401 | 0.168 | 2.45225632472492e-78 |
| SLC1A4 | 6.06378259386978e-58 | -0.405946997009729 | 0.283 | 0.477 | 1.12343700116625e-53 |
| DDX17 | 1.10623924905846e-50 | -0.405852184798527 | 0.88 | 0.946 | 2.04952945673062e-46 |
| PRKACB | 1.16982990156994e-74 | -0.405662591590598 | 0.261 | 0.485 | 2.16734385863863e-70 |
| NUDC | 2.95752483650782e-125 | 0.405102239013173 | 0.919 | 0.825 | 5.47940626459805e-121 |
| CCM2 | 4.08189227771686e-62 | -0.405085367580464 | 0.509 | 0.672 | 7.56252182292603e-58 |
| NASP | 4.56876086903338e-75 | 0.403700146988051 | 0.753 | 0.641 | 8.46454326205815e-71 |
| SRSF2 | 1.22504863161966e-131 | 0.403480546440994 | 0.931 | 0.859 | 2.26964759980174e-127 |
| TP53INP1 | 3.39849790533144e-49 | -0.403245656385181 | 0.144 | 0.357 | 6.29639706920757e-45 |
| GYPC | 1.55510533310148e-51 | -0.401600737052275 | 0.685 | 0.794 | 2.88114365063711e-47 |
| 09-sept | 1.48759417336641e-58 | -0.401165262303221 | 0.52 | 0.686 | 2.75606572499595e-54 |
| CEBPB | 1.59593273349129e-85 | 0.400722320113139 | 0.513 | 0.375 | 2.95678457533931e-81 |
| ISOC2 | 1.3652170616171e-109 | 0.399452032454306 | 0.511 | 0.303 | 2.529337650058e-105 |
| KMT2A | 3.66334406024211e-46 | -0.39938427750869 | 0.756 | 0.852 | 6.78707754041056e-42 |
| MCM4 | 4.52664008056059e-69 | 0.399079180612043 | 0.389 | 0.219 | 8.38650607725461e-65 |
| PIK3IP1 | 2.30478506356961e-54 | -0.399006308271139 | 0.153 | 0.38 | 4.27007528727542e-50 |
| PHB2 | 2.05913074785819e-122 | 0.398870128914992 | 0.805 | 0.667 | 3.81495153655686e-118 |
| SSRP1 | 1.36090668422925e-106 | 0.398627996802899 | 0.627 | 0.44 | 2.52135181387153e-102 |
| RPS27 | 1.47391528319772e-250 | -0.397531218500265 | 1 | 1 | 2.73072284518041e-246 |
| COPRS | 3.63690014229021e-108 | 0.396918189745812 | 0.491 | 0.273 | 6.73808489362107e-104 |
| MBP | 5.34054461577474e-49 | -0.396844246391584 | 0.878 | 0.945 | 9.89442700964585e-45 |
| FBL | 5.30665727537564e-108 | 0.396620090012926 | 0.743 | 0.575 | 9.83164393408845e-104 |
| HCST | 1.18982563129085e-36 | -0.396300777552484 | 0.719 | 0.817 | 2.20438994709255e-32 |
| TAP1 | 4.07805739192595e-105 | 0.395616448967628 | 0.926 | 0.848 | 7.5554169300212e-101 |
| LPIN2 | 1.52100390903279e-51 | -0.394812494444603 | 0.511 | 0.684 | 2.81796394226505e-47 |
| BCL11B | 1.12370546404063e-47 | -0.394577844846173 | 0.491 | 0.666 | 2.08188911322808e-43 |
| ATIC | 4.54605848722168e-104 | 0.39370623941508 | 0.584 | 0.386 | 8.42248255927561e-100 |
| CCR7 | 1.27094770608263e-34 | 0.393517765101523 | 0.589 | 0.406 | 2.35468481505929e-30 |
| GNA15 | 1.56892598543865e-72 | 0.393269747352957 | 0.513 | 0.364 | 2.90674917322218e-68 |
| GRPEL1 | 3.92089804528467e-107 | 0.392860601746837 | 0.58 | 0.38 | 7.2642478084989e-103 |
| MYC | 1.42567917375198e-46 | 0.392472872764808 | 0.432 | 0.29 | 2.6413558052103e-42 |
| TNIP2 | 7.78439645308597e-84 | 0.392193720235315 | 0.711 | 0.545 | 1.44221513086324e-79 |
| MRPS12 | 3.71306767655901e-126 | 0.392126155552446 | 0.832 | 0.728 | 6.87920048436087e-122 |
| DDIT4 | 6.07478394703113e-56 | 0.390962247912401 | 0.865 | 0.794 | 1.12547522186646e-51 |
| EEF1E1 | 1.37162219790844e-101 | 0.390723669006578 | 0.631 | 0.463 | 2.54120444606496e-97 |
| LYAR | 2.08241786504805e-68 | 0.390597063181709 | 0.637 | 0.505 | 3.85809557857453e-64 |
| OAS1 | 1.36776591021251e-64 | 0.390284017729106 | 0.354 | 0.181 | 2.53405990185072e-60 |
| STK10 | 5.72841467013984e-61 | -0.389443165348171 | 0.398 | 0.593 | 1.06130338593681e-56 |
| EPB41 | 6.88525961417241e-64 | -0.388775150485027 | 0.248 | 0.439 | 1.27563204871772e-59 |
| PSMC3 | 4.37852237171427e-109 | 0.388745695196516 | 0.784 | 0.627 | 8.11208839807503e-105 |
| C8orf33 | 1.36356915275444e-103 | 0.387888454592153 | 0.537 | 0.338 | 2.52628456930815e-99 |
| CCDC53 | 1.46182742585179e-62 | -0.387738559438682 | 0.44 | 0.625 | 2.70832767187562e-58 |
| RP5-1171I10.5 | 4.77539365484354e-35 | -0.38727706138339 | 0.886 | 0.949 | 8.84737182432863e-31 |
| HINT1 | 2.3229551877894e-99 | -0.386807544546409 | 1 | 1 | 4.30373907641742e-95 |
| BST2 | 6.79052637630003e-102 | 0.386710763291589 | 0.954 | 0.941 | 1.25808082173711e-97 |
| TOP1 | 7.71732913743157e-104 | 0.386323129781691 | 0.928 | 0.854 | 1.42978956929195e-99 |
| SNRPF | 1.31045403161378e-142 | 0.385732334207394 | 0.945 | 0.898 | 2.42787818437086e-138 |
| SMAD3 | 3.58077602730062e-62 | -0.384588287588307 | 0.224 | 0.444 | 6.63410374577986e-58 |
| YRDC | 3.97895881898432e-90 | 0.383904673287844 | 0.422 | 0.207 | 7.37181700393226e-86 |
| IL16 | 5.2436375448276e-36 | -0.383739667272252 | 0.105 | 0.328 | 9.7148872793021e-32 |
| ELL2 | 7.45195246903883e-85 | 0.383231643413749 | 0.471 | 0.286 | 1.38062323393882e-80 |
| CD3D | 1.24594690432049e-46 | -0.383156158235491 | 0.993 | 0.997 | 2.30836582963457e-42 |
| CD151 | 1.36425280929855e-95 | 0.382816979862395 | 0.558 | 0.359 | 2.52755117978742e-91 |
| SNU13 | 1.7082115407175e-142 | 0.382617341484015 | 0.969 | 0.929 | 3.16480352148731e-138 |
| HELLS | 1.56635179738707e-53 | 0.382374782564699 | 0.421 | 0.296 | 2.90197997501902e-49 |
| NDUFB1 | 1.07960827599467e-64 | -0.382070782600122 | 0.916 | 0.969 | 2.00019025293533e-60 |
| CHEK1 | 7.5387551484577e-31 | 0.381620225189375 | 0.264 | 0.107 | 1.39670516635476e-26 |
| NDUFAB1 | 1.71942199255763e-145 | 0.381491368641869 | 0.94 | 0.876 | 3.18557312561152e-141 |
| SUN2 | 1.37137765083234e-62 | -0.381259132938439 | 0.356 | 0.568 | 2.54075137369707e-58 |
| N4BP2L2 | 6.9049569525083e-42 | -0.381144088466564 | 0.858 | 0.94 | 1.27928137459121e-37 |
| SLC43A3 | 1.33010447881638e-34 | 0.379992559130192 | 0.295 | 0.092 | 2.46428456790311e-30 |
| H2AFJ | 8.28033212670901e-60 | -0.379597670867036 | 0.349 | 0.553 | 1.53409713311538e-55 |
| TUFM | 4.17402697550936e-117 | 0.378055783304653 | 0.867 | 0.752 | 7.73321977752619e-113 |
| EIF3J | 5.29722955064571e-95 | 0.377398549673928 | 0.889 | 0.792 | 9.8141771884813e-91 |
| VAMP8 | 8.52206788586292e-59 | -0.377345494569058 | 0.875 | 0.941 | 1.57888351721382e-54 |
| RRP1 | 8.46997913832059e-96 | 0.37733718560832 | 0.46 | 0.249 | 1.56923303495666e-91 |
| RPL34 | 6.80459191893639e-220 | -0.377017403116377 | 1 | 1 | 1.26068674482135e-215 |
| HDGF | 2.05814099275308e-88 | 0.376749414465744 | 0.771 | 0.635 | 3.81311781727364e-84 |
| AKAP13 | 5.17216333131841e-43 | -0.376624513241738 | 0.648 | 0.805 | 9.58246700393362e-39 |
| CLEC2B | 5.48231463318813e-32 | -0.376562252311011 | 0.61 | 0.776 | 1.01570843209076e-27 |
| VMP1 | 2.49177600125321e-32 | -0.376164309630232 | 0.916 | 0.971 | 4.61651339752182e-28 |
| CAPG | 1.32579971583697e-30 | -0.375489414780361 | 0.298 | 0.414 | 2.45630913353116e-26 |
| EIF6 | 1.53840016520271e-116 | 0.375149560574022 | 0.892 | 0.785 | 2.85019398607106e-112 |
| PSMB3 | 2.20058985273914e-154 | 0.374703844101244 | 0.992 | 0.974 | 4.0770328201698e-150 |
| PNO1 | 2.58081293075008e-89 | 0.373970259935078 | 0.466 | 0.267 | 4.78147211680067e-85 |
| TOMM22 | 1.2960234932327e-109 | 0.372927142261173 | 0.762 | 0.626 | 2.40114272591223e-105 |
| KPNA2 | 1.99586210935944e-73 | 0.372901604044075 | 0.601 | 0.407 | 3.69773373001023e-69 |
| TRAP1 | 2.20059483550733e-62 | 0.372532483907373 | 0.369 | 0.152 | 4.07704205174442e-58 |
| PSMD1 | 1.26929190598994e-94 | 0.372487808874815 | 0.76 | 0.602 | 2.35161711422755e-90 |
| HAVCR2 | 4.73316890494136e-52 | 0.372322511411838 | 0.368 | 0.233 | 8.76914203018486e-48 |
| IER2 | 7.48354490420936e-25 | -0.372268875464328 | 0.746 | 0.808 | 1.38647636440287e-20 |
| RRP15 | 2.01636564323198e-94 | 0.37164043242245 | 0.514 | 0.307 | 3.73572062721588e-90 |
| ANTXR2 | 1.61006267696521e-38 | -0.371432307784372 | 0.345 | 0.515 | 2.98296312161345e-34 |
| CD3EAP | 2.75348835001628e-40 | 0.371262843197655 | 0.309 | 0.121 | 5.10138786607515e-36 |
| DESI1 | 1.64874709198832e-100 | 0.371048818365603 | 0.639 | 0.455 | 3.05463373732676e-96 |
| PPM1G | 2.3162347118361e-109 | 0.369540934129023 | 0.895 | 0.824 | 4.29128805061873e-105 |
| POLR3K | 2.82881881864667e-93 | 0.368538163983099 | 0.577 | 0.41 | 5.24095262530668e-89 |
| UBE2L6 | 1.6008182315016e-90 | 0.367150753699297 | 0.774 | 0.623 | 2.96583593750301e-86 |
| BCAT1 | 8.21329867216715e-69 | 0.367021821762598 | 0.427 | 0.264 | 1.52167784499241e-64 |
| ADD3 | 9.36544631240182e-47 | -0.366473022166142 | 0.473 | 0.648 | 1.73513623829869e-42 |
| ORAI2 | 5.6209396529746e-41 | -0.366383799119985 | 0.521 | 0.658 | 1.0413914895066e-36 |
| NMRK1 | 7.07434053616334e-48 | -0.366028086721953 | 0.405 | 0.561 | 1.31066307113498e-43 |
| RAB8B | 9.10930040565992e-54 | -0.36548641213195 | 0.384 | 0.572 | 1.68768008615661e-49 |
| MTIF3 | 3.17856500460062e-48 | -0.36500686149348 | 0.519 | 0.667 | 5.88892738402357e-44 |
| SDC4 | 1.59078024806743e-45 | 0.36498096575974 | 0.327 | 0.16 | 2.94723856559452e-41 |
| AAK1 | 1.2441290417095e-40 | -0.364418831153583 | 0.681 | 0.816 | 2.30499787557518e-36 |
| RNASEH2B | 2.53149938069033e-43 | -0.364022539578855 | 0.598 | 0.719 | 4.69010890260498e-39 |
| FUOM | 2.53730313703976e-84 | 0.363865541897931 | 0.423 | 0.22 | 4.70086152199357e-80 |
| RANGAP1 | 1.83201158600228e-91 | 0.363426369274005 | 0.503 | 0.31 | 3.39416786538642e-87 |
| GLTSCR2 | 7.38026360843787e-51 | -0.363385227151872 | 0.802 | 0.866 | 1.36734143873528e-46 |
| RGS10 | 8.67784821324342e-48 | -0.363200590248735 | 0.796 | 0.87 | 1.60774493846761e-43 |
| NPC2 | 1.38588056638783e-41 | -0.363052713006479 | 0.439 | 0.595 | 2.56762092534674e-37 |
| EIF2S1 | 5.12833254287738e-95 | 0.362641375424305 | 0.757 | 0.603 | 9.50126170218892e-91 |
| CTPS1 | 2.99460503515261e-55 | 0.362509075528913 | 0.36 | 0.138 | 5.54810474862725e-51 |
| METRN | 2.99893205753823e-75 | 0.362159526802008 | 0.418 | 0.231 | 5.55612142300108e-71 |
| LY6E | 1.0997579178587e-74 | 0.36182753758828 | 0.868 | 0.829 | 2.03752149441681e-70 |
| MRPL27 | 1.47026127923165e-100 | 0.361694686071585 | 0.656 | 0.476 | 2.72395307203248e-96 |
| ATP5B | 3.62051308567568e-128 | 0.361670674366466 | 0.967 | 0.916 | 6.70772459383133e-124 |
| CDV3 | 1.58770527658203e-97 | 0.361032056800874 | 0.934 | 0.878 | 2.94154156592353e-93 |
| GZMA | 2.56054595539159e-05 | -0.360872917238106 | 0.36 | 0.395 | 0.474392349155401 |
| RPF2 | 6.95402427165739e-89 | 0.360475005152437 | 0.495 | 0.315 | 1.28837207680996e-84 |
| PSMB5 | 4.08697929795183e-99 | 0.360225242288987 | 0.689 | 0.534 | 7.57194654531536e-95 |
| EIF5B | 6.92675577283931e-101 | 0.359867378883826 | 0.951 | 0.908 | 1.28332004203394e-96 |
| CCDC124 | 3.6523529970167e-95 | 0.359709491279122 | 0.68 | 0.517 | 6.76671439757285e-91 |
| GGCT | 6.27202304681648e-87 | 0.358931996421664 | 0.603 | 0.436 | 1.16201770988369e-82 |
| SNRPA1 | 2.99568882288183e-90 | 0.358735545153975 | 0.516 | 0.328 | 5.55011268215317e-86 |
| PABPC4 | 1.1325565945082e-91 | 0.358569982433827 | 0.534 | 0.338 | 2.09828760264534e-87 |
| STOML2 | 1.73099601969838e-112 | 0.358282383779314 | 0.843 | 0.731 | 3.20701632569518e-108 |
| LSM2 | 1.47502771462936e-97 | 0.357644834932711 | 0.813 | 0.726 | 2.73278384689382e-93 |
| EIF4A3 | 1.27830947278429e-92 | 0.357266566670839 | 0.685 | 0.52 | 2.36832396022745e-88 |
| ANP32B | 4.09441812367412e-105 | 0.357060478975613 | 0.956 | 0.914 | 7.58572845773104e-101 |
| RPL39 | 2.01754640175149e-166 | -0.356935213547016 | 1 | 1 | 3.73790821852499e-162 |
| PSMB7 | 3.52529676179982e-107 | 0.356923805124367 | 0.845 | 0.715 | 6.53131731058653e-103 |
| UBE2M | 1.19087711728868e-107 | 0.356542405101361 | 0.874 | 0.778 | 2.20633803520074e-103 |
| PARP14 | 4.4545000809811e-72 | 0.355711747865683 | 0.764 | 0.655 | 8.25285230003369e-68 |
| MAP3K8 | 2.48670524390267e-68 | 0.355582482953435 | 0.389 | 0.226 | 4.60711880537848e-64 |
| ATP5E | 5.23449204927572e-94 | -0.354896660377088 | 1 | 1 | 9.69794341969313e-90 |
| AHSA1 | 6.6758296442665e-91 | 0.354886250310644 | 0.708 | 0.547 | 1.23683095819325e-86 |
| STK17A | 2.03319275672573e-38 | -0.354824580107226 | 0.846 | 0.925 | 3.76689622038577e-34 |
| UPP1 | 1.3517498844714e-68 | 0.354539110269079 | 0.537 | 0.39 | 2.50438701096016e-64 |
| NAA10 | 3.29015775791311e-97 | 0.354402891788242 | 0.765 | 0.614 | 6.09567527808563e-93 |
| UBE2T | 3.84367647765344e-51 | 0.353925252867346 | 0.355 | 0.197 | 7.12117941014852e-47 |
| MPDU1 | 6.70144007827537e-94 | 0.3535894962145 | 0.473 | 0.259 | 1.24157580330208e-89 |
| PHLDA2 | 1.97096840575178e-31 | 0.353500680815748 | 0.38 | 0.26 | 3.65161316533632e-27 |
| MAGOHB | 7.80980721342621e-96 | 0.353283116435297 | 0.482 | 0.285 | 1.44692298243147e-91 |
| EIF4G2 | 3.73680684133473e-94 | 0.353023004952491 | 0.936 | 0.87 | 6.92318203494086e-90 |
| SSSCA1 | 2.25569599483125e-87 | 0.352958482649961 | 0.553 | 0.362 | 4.17912796962386e-83 |
| AK2 | 5.07120343047312e-93 | 0.35261619418846 | 0.542 | 0.344 | 9.39541859563756e-89 |
| RRP7A | 8.0823239419297e-96 | 0.352527614014476 | 0.533 | 0.332 | 1.49741215672132e-91 |
| BZW2 | 7.97139579488223e-86 | 0.35205153974104 | 0.469 | 0.277 | 1.47686049891783e-81 |
| SNRPD3 | 8.99830841279421e-107 | 0.352048192997005 | 0.895 | 0.785 | 1.66711659963838e-102 |
| STK4 | 3.37452918999024e-40 | -0.351833536313701 | 0.789 | 0.889 | 6.25199023029492e-36 |
| PSMD7 | 6.8165467258326e-106 | 0.351663027855294 | 0.929 | 0.844 | 1.26290161189501e-101 |
| PDAP1 | 5.35761125063956e-98 | 0.350904257767895 | 0.851 | 0.728 | 9.92604636405991e-94 |
| DDX39A | 1.95749293642471e-80 | 0.350285450418353 | 0.616 | 0.435 | 3.62664716331406e-76 |
| COTL1 | 6.43842860341928e-23 | -0.350027814996341 | 0.884 | 0.942 | 1.19284766735549e-18 |
| C19orf48 | 4.20159203830205e-46 | 0.349997381119303 | 0.327 | 0.161 | 7.78428956936221e-42 |
| PDGFA | 1.07568224436567e-30 | 0.349889371256041 | 0.272 | 0.117 | 1.99291649413627e-26 |
| FEN1 | 8.24507359071569e-33 | 0.348631008354805 | 0.281 | 0.129 | 1.5275647841519e-28 |
| RNF19A | 4.30245516790103e-45 | 0.34848457622557 | 0.732 | 0.705 | 7.97115868957023e-41 |
| FASN | 1.90755750860575e-90 | 0.348194966385291 | 0.495 | 0.281 | 3.53413179619388e-86 |
| HSPB1 | 9.94715691209281e-32 | -0.34732213899054 | 0.528 | 0.638 | 1.84290976110343e-27 |
| SORL1 | 1.70344075888914e-38 | -0.347224946320478 | 0.166 | 0.352 | 3.15596469399391e-34 |
| ALDOA | 9.73761737370339e-133 | 0.347142172870235 | 0.998 | 0.996 | 1.80408837082603e-128 |
| F2R | 7.57183284625392e-45 | -0.347116472817328 | 0.261 | 0.444 | 1.40283347142546e-40 |
| ATAD3B | 9.66680915331081e-52 | 0.346701187300231 | 0.349 | 0.147 | 1.79096973183389e-47 |
| FARSA | 9.86243272963254e-93 | 0.346695053110588 | 0.57 | 0.386 | 1.82721291181902e-88 |
| DNAJC2 | 2.19195276708681e-77 | 0.346636886091042 | 0.623 | 0.455 | 4.06103089158173e-73 |
| CD48 | 1.94653129695551e-32 | -0.345564550715114 | 0.754 | 0.865 | 3.60633853386947e-28 |
| BTG2 | 3.20149264677841e-19 | -0.345174755316982 | 0.475 | 0.636 | 5.93140542668637e-15 |
| PLAGL2 | 7.25989975925469e-64 | 0.34511817366751 | 0.38 | 0.18 | 1.34504162839712e-59 |
| NARF | 1.44227962476601e-67 | 0.344771081088141 | 0.666 | 0.574 | 2.67211146080399e-63 |
| NOL7 | 1.33840816288932e-99 | 0.344744761027923 | 0.926 | 0.844 | 2.47966880338503e-95 |
| SRPRB | 7.77141820042268e-91 | 0.344479027247478 | 0.516 | 0.318 | 1.43981064999231e-86 |
| STRA13 | 6.57760047884909e-72 | 0.343931662443315 | 0.639 | 0.501 | 1.21863204071637e-67 |
| FRMD4B | 2.11085734529895e-44 | 0.343742891554503 | 0.569 | 0.452 | 3.91078540363537e-40 |
| PSMC1 | 1.03767468272463e-95 | 0.343080289230919 | 0.829 | 0.692 | 1.92249988468392e-91 |
| LAPTM5 | 5.14211667165787e-24 | -0.342438633335105 | 0.822 | 0.922 | 9.52679955758053e-20 |
| ACOT7 | 1.69203963960453e-67 | 0.342195658723539 | 0.396 | 0.186 | 3.1348418402953e-63 |
| FASLG | 1.18593830686443e-47 | 0.341573357516929 | 0.348 | 0.189 | 2.19718790112772e-43 |
| ODF2L | 3.6971655301648e-42 | -0.341166638792506 | 0.404 | 0.571 | 6.84973857773633e-38 |
| RPL35 | 0 | 0.341106579566845 | 1 | 1 | 0 |
| DTYMK | 1.11347990461117e-71 | 0.340797763405285 | 0.479 | 0.309 | 2.06294421927312e-67 |
| MCM5 | 6.53430885524069e-38 | 0.340719440054129 | 0.316 | 0.172 | 1.21061140161044e-33 |
| DDX1 | 8.47907462204662e-85 | 0.340685729735741 | 0.633 | 0.45 | 1.57091815522658e-80 |
| IFIT2 | 2.01122489097738e-31 | 0.340550606946686 | 0.317 | 0.094 | 3.72619635551379e-27 |
| UBE2N | 1.34042686396728e-98 | 0.340084012999659 | 0.845 | 0.725 | 2.48340885087218e-94 |
| AIMP1 | 3.27626022529111e-80 | 0.34006811695137 | 0.774 | 0.656 | 6.06992731939685e-76 |
| CAMK1 | 6.83105090930238e-62 | 0.339933754254835 | 0.407 | 0.242 | 1.26558880196645e-57 |
| YWHAE | 3.92076420537868e-93 | 0.338819403156011 | 0.925 | 0.845 | 7.26399984330508e-89 |
| SEC61B | 1.32522431697052e-111 | 0.338615561207834 | 0.991 | 0.985 | 2.45524309205128e-107 |
| IARS | 5.3925041753904e-82 | 0.338552269841885 | 0.567 | 0.397 | 9.9906924857458e-78 |
| ARHGAP15 | 1.31517462623524e-40 | -0.338325962470047 | 0.559 | 0.703 | 2.43662403002602e-36 |
| ADRM1 | 2.43001128841286e-89 | 0.338262296127929 | 0.817 | 0.689 | 4.50208191404251e-85 |
| TMEM208 | 9.64838907190436e-92 | 0.338108021942797 | 0.77 | 0.621 | 1.78755704335172e-87 |
| C11orf31 | 6.80508061161098e-96 | 0.337111806412839 | 0.899 | 0.827 | 1.26077728491317e-91 |
| ARHGDIA | 9.1459232803498e-100 | 0.33701093253787 | 0.937 | 0.854 | 1.69446520615041e-95 |
| CLDND1 | 6.7553451112054e-28 | -0.336782919647965 | 0.79 | 0.861 | 1.25156278875302e-23 |
| XRCC5 | 1.17368681240596e-104 | 0.336679650219914 | 0.98 | 0.957 | 2.17448955734453e-100 |
| MED10 | 1.66828560777718e-80 | 0.336061294799335 | 0.767 | 0.644 | 3.09083274552878e-76 |
| ANKRD12 | 7.84740208635777e-27 | -0.335902248919451 | 0.843 | 0.919 | 1.4538881845395e-22 |
| TSR1 | 2.5988368282525e-71 | 0.335727901444428 | 0.42 | 0.224 | 4.81486499170341e-67 |
| KPNB1 | 7.51758305820428e-79 | 0.335194553128909 | 0.888 | 0.819 | 1.39278261319351e-74 |
| DIXDC1 | 4.95052243417415e-49 | 0.335181872139559 | 0.346 | 0.167 | 9.17183291379445e-45 |
| DUSP5 | 4.78305153033076e-45 | 0.335155616828508 | 0.432 | 0.275 | 8.86155957024379e-41 |
| DOCK8 | 3.79993770979348e-35 | -0.334811182264035 | 0.658 | 0.789 | 7.04014459493437e-31 |
| PRELID1 | 5.74172565936035e-123 | 0.334639106140536 | 0.983 | 0.962 | 1.06376951290969e-118 |
| POMP | 1.34158858265347e-127 | 0.334451772690331 | 0.992 | 0.986 | 2.48556116708209e-123 |
| ESF1 | 1.51219348661467e-69 | 0.333954252009587 | 0.574 | 0.416 | 2.801640872651e-65 |
| PTBP1 | 2.22967901957703e-79 | 0.333709172667504 | 0.623 | 0.446 | 4.13092631957036e-75 |
| TNFSF10 | 3.67792296586415e-48 | 0.333679561075234 | 0.777 | 0.775 | 6.81408787885652e-44 |
| RARS | 8.66502258278205e-84 | 0.333411235241398 | 0.597 | 0.414 | 1.60536873391203e-79 |
| DCAF13 | 1.47333334652882e-80 | 0.333323144222498 | 0.638 | 0.498 | 2.72964469111394e-76 |
| SESN3 | 8.2431073252937e-16 | -0.333314055008512 | 0.089 | 0.252 | 1.52720049415716e-11 |
| CDKN1B | 6.39988049990657e-45 | -0.333126053598579 | 0.335 | 0.513 | 1.18570586021769e-40 |
| CLSPN | 3.52771513573343e-14 | 0.333075672183065 | 0.197 | 0.072 | 6.53579783197333e-10 |
| ALKBH7 | 2.35652730240039e-49 | -0.332773611538124 | 0.318 | 0.496 | 4.36593813315721e-45 |
| KDELR2 | 3.39382817023467e-82 | 0.332763309634228 | 0.728 | 0.568 | 6.28774545099378e-78 |
| EVI2A | 5.13260865059655e-37 | -0.332433905869709 | 0.31 | 0.496 | 9.50918404696022e-33 |
| RPL30 | 1.18658998615744e-176 | -0.332360341803387 | 1 | 1 | 2.19839526735389e-172 |
| HIF1A | 1.09933982622147e-53 | 0.332307535514938 | 0.813 | 0.736 | 2.03674689604051e-49 |
| LIMA1 | 3.07354253212632e-55 | 0.331974175700548 | 0.723 | 0.626 | 5.69435224927043e-51 |
| MCM3 | 1.41490584132078e-51 | 0.331842395830145 | 0.451 | 0.35 | 2.62139605221501e-47 |
| AK4 | 2.20218979210502e-34 | 0.331824645162269 | 0.301 | 0.128 | 4.07999702783297e-30 |
| YIF1A | 2.88285761708811e-83 | 0.331225578583516 | 0.656 | 0.514 | 5.34107030717915e-79 |
| JUN | 1.83014184486895e-34 | 0.330658015756417 | 0.588 | 0.483 | 3.3907037959887e-30 |
| LINC00152 | 1.70903753936677e-39 | 0.330344900845243 | 0.76 | 0.662 | 3.16633384918481e-35 |
| SPATS2L | 4.3707112459709e-67 | 0.329981125920668 | 0.649 | 0.525 | 8.09761672541029e-63 |
| POP7 | 1.04184597693989e-88 | 0.32979602562811 | 0.566 | 0.39 | 1.93022804147653e-84 |
| SKIL | 7.35182704686228e-70 | 0.329382414241332 | 0.452 | 0.252 | 1.36207299697217e-65 |
| RPS19BP1 | 2.91286006205908e-99 | 0.328983381523326 | 0.961 | 0.907 | 5.39665583697686e-95 |
| PGAP1 | 1.71879192867426e-59 | 0.328939553610785 | 0.435 | 0.274 | 3.1844058062548e-55 |
| IFI44 | 8.30856705973914e-25 | 0.328731536677324 | 0.27 | 0.098 | 1.53932821915787e-20 |
| TBC1D10C | 1.09350400130542e-39 | -0.328649491480081 | 0.492 | 0.656 | 2.02593486321855e-35 |
| LEPROTL1 | 2.84734024546456e-37 | -0.3274726091074 | 0.693 | 0.787 | 5.27526727277219e-33 |
| C5orf56 | 1.02244614990782e-36 | -0.327309628802402 | 0.432 | 0.581 | 1.89428598193423e-32 |
| OSTF1 | 4.61839349672567e-29 | -0.327087318464133 | 0.859 | 0.913 | 8.55649763138365e-25 |
| LY96 | 5.33909971601518e-40 | -0.326784710450173 | 0.254 | 0.448 | 9.89175004386132e-36 |
| CSTB | 1.11203423724212e-37 | -0.326770057180006 | 0.951 | 0.981 | 2.06026583133848e-33 |
| MRPS25 | 1.16676033900458e-82 | 0.326706955840261 | 0.528 | 0.352 | 2.16165688007378e-78 |
| PSMD3 | 2.04171446859974e-86 | 0.326661674275018 | 0.578 | 0.396 | 3.78268439597474e-82 |
| RGCC | 9.36155788553345e-29 | 0.326576088640705 | 0.592 | 0.586 | 1.73441582945278e-24 |
| ISG20 | 1.42033106168021e-13 | -0.326087485554721 | 0.573 | 0.701 | 2.63144735797492e-09 |
| CXCR6 | 2.00848554978788e-14 | -0.32601567288696 | 0.466 | 0.527 | 3.72112117809201e-10 |
| SEC61G | 5.46111981821793e-116 | 0.325609200758176 | 0.993 | 0.979 | 1.01178166872124e-111 |
| BCL2L1 | 4.41730371738018e-60 | 0.325599796641959 | 0.488 | 0.339 | 8.18393859719025e-56 |
| VIM | 9.96863577278546e-44 | 0.325270099604012 | 0.988 | 0.993 | 1.84688914962396e-39 |
| ATP6V0B | 2.22454824730731e-87 | 0.324737147816569 | 0.924 | 0.829 | 4.12142053778626e-83 |
| GSTK1 | 1.06259143926347e-33 | -0.32397762187799 | 0.834 | 0.909 | 1.96866315952343e-29 |
| CYTH1 | 6.95803308418496e-40 | -0.323712562328237 | 0.436 | 0.582 | 1.28911478950695e-35 |
| PSMD8 | 1.08401235444337e-116 | 0.323656808291235 | 0.98 | 0.939 | 2.00834968907723e-112 |
| ZC3H12D | 6.40326724695276e-35 | -0.3236096455992 | 0.354 | 0.51 | 1.18633332284294e-30 |
| APEX1 | 1.76137708733918e-73 | 0.32338052808963 | 0.7 | 0.583 | 3.2633033297133e-69 |
| TPP1 | 5.42159984328968e-44 | -0.323348729848181 | 0.357 | 0.525 | 1.00445980296628e-39 |
| PSMC5 | 1.42781726142249e-103 | 0.323169224910013 | 0.929 | 0.847 | 2.64531704023745e-99 |
| BTG3 | 1.11127145566525e-52 | 0.322654629876514 | 0.579 | 0.432 | 2.05885262591101e-48 |
| SUSD3 | 1.51155485382759e-43 | -0.32253609426332 | 0.301 | 0.483 | 2.80045767768638e-39 |
| IPO5 | 4.80434203926884e-70 | 0.322480638303626 | 0.426 | 0.25 | 8.90100449615338e-66 |
| RSAD2 | 3.74296240085856e-12 | 0.322110440695437 | 0.205 | 0.056 | 6.93458644007066e-08 |
| PTGES3 | 7.35027235860972e-102 | 0.321887366873583 | 0.977 | 0.963 | 1.36178495987962e-97 |
| MDFIC | 9.97526287596542e-48 | 0.321582364799461 | 0.593 | 0.478 | 1.84811695303011e-43 |
| SLC5A3 | 2.99210045129457e-22 | -0.321523756055633 | 0.58 | 0.682 | 5.54346450611344e-18 |
| DUT | 2.68325450415265e-35 | 0.321500379082106 | 0.714 | 0.697 | 4.97126561984361e-31 |
| TCTN3 | 1.73235742304457e-48 | 0.320957525843735 | 0.448 | 0.333 | 3.20953859767467e-44 |
| RNASET2 | 1.83941053510372e-30 | -0.320823059594383 | 0.557 | 0.682 | 3.40787589838666e-26 |
| CDC37 | 8.28343532702991e-86 | 0.320734143078063 | 0.851 | 0.748 | 1.53467206303883e-81 |
| TPM4 | 8.66986517987518e-59 | 0.31997044930034 | 0.972 | 0.957 | 1.60626592187548e-54 |
| PIM2 | 5.07017537727213e-26 | -0.3199185926235 | 0.593 | 0.723 | 9.39351392147208e-22 |
| PDE3B | 1.86911328718576e-34 | -0.319854218805009 | 0.264 | 0.401 | 3.46290618716906e-30 |
| CST7 | 5.77015394213613e-14 | -0.3197644073953 | 0.573 | 0.669 | 1.06903642085956e-09 |
| CD9 | 1.40629135503283e-46 | 0.319735096312519 | 0.351 | 0.233 | 2.60543599346932e-42 |
| TIMM10 | 3.49544669644331e-77 | 0.319533264909485 | 0.576 | 0.4 | 6.47601409450053e-73 |
| ALG3 | 1.10768300769608e-78 | 0.319503255964369 | 0.486 | 0.312 | 2.05220430835853e-74 |
| NDFIP2 | 7.08528269861534e-65 | 0.319498604877545 | 0.509 | 0.344 | 1.31269032557246e-60 |
| TRIM28 | 2.76795895531476e-72 | 0.319395481338471 | 0.618 | 0.46 | 5.12819755651165e-68 |
| NOP58 | 4.38135778513159e-63 | 0.318928029237134 | 0.804 | 0.679 | 8.11734156851329e-59 |
| AC016831.7 | 9.37445421992367e-55 | 0.318909178077314 | 0.373 | 0.249 | 1.73680513332526e-50 |
| RBM17 | 4.21318832828336e-72 | 0.318523533943344 | 0.776 | 0.667 | 7.80577401581058e-68 |
| TRAT1 | 6.96171933306874e-29 | -0.318276070223642 | 0.523 | 0.668 | 1.28979774083765e-24 |
| GRWD1 | 3.42785369311366e-68 | 0.318013873983366 | 0.413 | 0.227 | 6.35078453723168e-64 |
| MCM6 | 7.16656219197251e-57 | 0.317759376550995 | 0.541 | 0.378 | 1.32774897730675e-52 |
| TMA16 | 1.06903253924882e-71 | 0.317679036539675 | 0.531 | 0.354 | 1.9805965854663e-67 |
| CISD3 | 1.25033571270255e-71 | 0.316830730595217 | 0.674 | 0.521 | 2.31649697492402e-67 |
| IFIT1 | 1.91518700607295e-13 | 0.316705761491748 | 0.242 | 0.044 | 3.54826696615135e-09 |
| MIR4435-2HG | 1.66821019035439e-27 | 0.316122187648364 | 0.608 | 0.531 | 3.09069301966957e-23 |
| CCDC86 | 1.65557446497967e-27 | 0.31596502486405 | 0.29 | 0.103 | 3.06728281126783e-23 |
| FAM117A | 2.02979563213777e-44 | -0.315960294509715 | 0.262 | 0.449 | 3.76060236766165e-40 |
| EMG1 | 3.25132730920219e-78 | 0.315740832220616 | 0.49 | 0.31 | 6.02373410575889e-74 |
| RASGRP1 | 1.56459161629901e-23 | -0.315670080070906 | 0.696 | 0.811 | 2.89871888751718e-19 |
| VAMP2 | 3.49536746213519e-36 | -0.315607756133216 | 0.563 | 0.696 | 6.47586729709786e-32 |
| JAML | 1.23681623953073e-23 | -0.315392426316823 | 0.584 | 0.713 | 2.29144944697858e-19 |
| EVI5 | 3.7875018019642e-34 | 0.315332149219672 | 0.317 | 0.152 | 7.01710458849908e-30 |
| USP12 | 7.87113123271368e-75 | 0.314967395298313 | 0.492 | 0.302 | 1.45828448348486e-70 |
| B2M | 9.97154760876371e-51 | -0.314783812433778 | 1 | 1 | 1.84742862547565e-46 |
| CDK4 | 9.40395538635764e-69 | 0.314746929725991 | 0.477 | 0.33 | 1.74227081443048e-64 |
| OSM | 3.23609688079982e-08 | 0.314679339083939 | 0.168 | 0.041 | 0.000599551669105783 |
| OASL | 1.99196283007564e-41 | 0.313695985424914 | 0.359 | 0.18 | 3.69050953528114e-37 |
| ABCE1 | 4.07617464414894e-61 | 0.313095532898325 | 0.714 | 0.595 | 7.55192876321475e-57 |
| HSPA4 | 4.28740927012967e-68 | 0.312998311889374 | 0.655 | 0.489 | 7.94328315476924e-64 |
| HIGD2A | 1.2942213265049e-37 | -0.312977591519599 | 0.697 | 0.793 | 2.39780385161563e-33 |
| MRPS7 | 1.17018919213317e-81 | 0.312734765438634 | 0.685 | 0.535 | 2.16800951626513e-77 |
| RUVBL1 | 2.10177516063295e-69 | 0.31217123275917 | 0.621 | 0.495 | 3.89395884010466e-65 |
| RABGAP1L | 2.04966002237426e-39 | -0.311546012370041 | 0.374 | 0.53 | 3.79740512345279e-35 |
| UBE2L3 | 1.19572152172032e-96 | 0.310294264500191 | 0.938 | 0.857 | 2.21531326329124e-92 |
| IDH2 | 4.18881474330862e-32 | -0.3102864577776 | 0.26 | 0.405 | 7.76061707492787e-28 |
| RASGRP2 | 2.45156437807517e-21 | -0.310168170661127 | 0.132 | 0.286 | 4.54201332325987e-17 |
| C19orf70 | 1.05300769675545e-89 | 0.310090464109805 | 0.874 | 0.776 | 1.95090735977882e-85 |
| LONP1 | 9.61053485696209e-48 | 0.310030579720622 | 0.348 | 0.17 | 1.78054379294937e-43 |
| ATAD3A | 2.754250221729e-36 | 0.309974006241826 | 0.323 | 0.13 | 5.10279938579731e-32 |
| GABPB1-AS1 | 7.47309694527283e-21 | -0.309919091662317 | 0.702 | 0.811 | 1.3845406710507e-16 |
| GARS | 3.27233916034825e-59 | 0.309836592288686 | 0.627 | 0.485 | 6.06266276237721e-55 |
| ICAM1 | 1.59614008033744e-46 | 0.309655446453365 | 0.366 | 0.17 | 2.95716872684117e-42 |
| ATP5G1 | 2.09202981325595e-89 | 0.309547862674463 | 0.911 | 0.841 | 3.87590363501929e-85 |
| GAR1 | 3.75069470636113e-74 | 0.309429626956195 | 0.502 | 0.327 | 6.94891208247526e-70 |
| ARHGEF6 | 9.25621788959827e-35 | -0.309335719771086 | 0.419 | 0.567 | 1.71489948840587e-30 |
| MRPL17 | 4.68806683852155e-74 | 0.309132907699097 | 0.493 | 0.338 | 8.68558143172888e-70 |
| FAM136A | 1.72095610644523e-71 | 0.309015628491679 | 0.518 | 0.369 | 3.18841537841107e-67 |
| HMGB2 | 1.9681675329252e-16 | -0.308855212579968 | 0.413 | 0.567 | 3.64642398825052e-12 |
| METTL1 | 8.36021068182672e-21 | 0.308831428668603 | 0.279 | 0.077 | 1.54889623302204e-16 |
| NDUFS5 | 1.90221690377831e-111 | 0.308696882812567 | 0.999 | 0.996 | 3.52423725763007e-107 |
| YPEL5 | 6.13445606562808e-44 | -0.308635593199278 | 0.291 | 0.467 | 1.13653067527891e-39 |
| EIF3I | 1.83884647200158e-98 | 0.308521169695657 | 0.903 | 0.815 | 3.40683085867732e-94 |
| GMNN | 1.37088543996519e-50 | 0.308458261517839 | 0.395 | 0.257 | 2.53983945462351e-46 |
| RRS1 | 7.06235342660035e-58 | 0.308288379406511 | 0.395 | 0.225 | 1.30844221934625e-53 |
| MT-ND3 | 9.99189106296296e-13 | -0.30822209360445 | 0.997 | 0.999 | 1.85119765723515e-08 |
| ARL6IP5 | 8.45196426559785e-29 | -0.307752749585444 | 0.98 | 0.994 | 1.56589541948731e-24 |
| SPTY2D1 | 1.22014432026725e-46 | 0.307664221813321 | 0.593 | 0.484 | 2.26056138215913e-42 |
| TMEM147 | 3.32282652507658e-70 | 0.307578228104428 | 0.675 | 0.529 | 6.15620070300938e-66 |
| TIPIN | 3.56006283931667e-31 | 0.307125663093216 | 0.301 | 0.129 | 6.59572842240199e-27 |
| GTF3C6 | 1.11848101187509e-91 | 0.306815390854271 | 0.902 | 0.824 | 2.07220977070098e-87 |
| LMNB1 | 2.12903399656042e-62 | 0.306548174683775 | 0.475 | 0.324 | 3.9444612854275e-58 |
| PTGDS | 2.27807184105798e-67 | 0.306181824527919 | 0.72 | 0.568 | 4.22058369992812e-63 |
| COX4I1 | 4.97514135450798e-53 | -0.30615968217958 | 0.997 | 0.999 | 9.21744438749693e-49 |
| TBRG4 | 5.0903118400561e-76 | 0.306125762608949 | 0.487 | 0.305 | 9.43082074607193e-72 |
| BIN2 | 1.62121141456188e-34 | -0.305789131084723 | 0.432 | 0.59 | 3.0036183877588e-30 |
| MXD4 | 3.60179726014349e-31 | -0.305612311078434 | 0.542 | 0.694 | 6.67304978386785e-27 |
| SZRD1 | 3.9573288535131e-73 | 0.304864599848368 | 0.662 | 0.501 | 7.33174316690372e-69 |
| C17orf89 | 6.19236077081791e-74 | 0.304751095982347 | 0.77 | 0.65 | 1.14725868000943e-69 |
| TIMM17A | 3.02798779371595e-72 | 0.304463283635712 | 0.652 | 0.492 | 5.60995298541754e-68 |
| WDR46 | 6.26726355580577e-71 | 0.304025409880499 | 0.509 | 0.343 | 1.16113591898413e-66 |
| CD96 | 2.20351372484846e-20 | -0.303162363529019 | 0.742 | 0.874 | 4.08244987802675e-16 |
| ATP5D | 7.11924731054633e-111 | 0.302985148398614 | 0.969 | 0.931 | 1.31898294922492e-106 |
| STRAP | 4.01738577232291e-75 | 0.302756283844698 | 0.759 | 0.643 | 7.44301062038265e-71 |
| NFKBIA | 3.32001457898507e-33 | 0.302715503052807 | 0.92 | 0.853 | 6.15099101048564e-29 |
| DUS1L | 1.17025043178026e-75 | 0.302662138460072 | 0.532 | 0.362 | 2.16812297495928e-71 |
| RRP9 | 3.99254571760793e-43 | 0.302660274186622 | 0.347 | 0.155 | 7.39698945101221e-39 |
| TKT | 4.90069629684659e-79 | 0.302199130764006 | 0.864 | 0.785 | 9.07952002916768e-75 |
| SIGIRR | 6.71787243348102e-40 | -0.302159767819679 | 0.394 | 0.569 | 1.24462022575103e-35 |
| FAM102A | 2.40738416984431e-28 | -0.301997318153259 | 0.166 | 0.339 | 4.46016065147055e-24 |
| DHX9 | 2.41155181022218e-62 | 0.301651342491136 | 0.633 | 0.504 | 4.46788203879864e-58 |
| BRI3 | 1.29910269207765e-31 | -0.30147942003847 | 0.622 | 0.718 | 2.40684755761226e-27 |
| REL | 2.06802255547764e-30 | 0.301139818874995 | 0.46 | 0.403 | 3.83142538853342e-26 |
| CD40LG | 9.22468997303988e-25 | 0.301020150219779 | 0.35 | 0.351 | 1.7090583113051e-20 |
| IL1B | 4.60110777062971e-07 | 0.300625758893316 | 0.184 | 0.023 | 0.00852447236664567 |
| RNPS1 | 2.60240281518102e-73 | 0.300506104772112 | 0.831 | 0.728 | 4.82147169568587e-69 |
| COA4 | 2.72375293676001e-73 | 0.300378270842782 | 0.745 | 0.628 | 5.04629706593527e-69 |
| ATPIF1 | 4.96012594820499e-38 | -0.300128633852872 | 0.953 | 0.98 | 9.18962534423939e-34 |

**Supplementary Table 3**: Differentially expressed genes (DEGs) with log2 fold change ≥ 0.3 and adjusted p value <0.01 in total human Th17-polarized cells following 12h coculture with primary human oligodendrocytes (OLs) comparing direct contact condition (Th17 cells are in direct contact with OLs) versus insert condition (Th17 cells separated from OLs by a porous membrane), single cell RNA sequencing. n = 1 OL prep and 1 T cell donor.

1. Esmaeili B, Mansouri P, Doustimotlagh AH, Izad M. Redox imbalance and IL-17 responses in memory CD4(+) T cells from patients with psoriasis. Scand J Immunol. 2019;89(1):e12730.

2. Kaufmann U, Kahlfuss S, Yang J, Ivanova E, Koralov SB, Feske S. Calcium Signaling Controls Pathogenic Th17 Cell-Mediated Inflammation by Regulating Mitochondrial Function. Cell Metab. 2019;29(5):1104-18.e6.

3. Moshfegh CM, Collins CW, Gunda V, Vasanthakumar A, Cao JZ, Singh PK, et al. Mitochondrial superoxide disrupts the metabolic and epigenetic landscape of CD4(+) and CD8(+) T-lymphocytes. Redox Biol. 2019;27:101141.

4. Kano S, Sato K, Morishita Y, Vollstedt S, Kim S, Bishop K, et al. The contribution of transcription factor IRF1 to the interferon-gamma-interleukin 12 signaling axis and TH1 versus TH-17 differentiation of CD4+ T cells. Nat Immunol. 2008;9(1):34-41.

5. Karwacz K, Miraldi ER, Pokrovskii M, Madi A, Yosef N, Wortman I, et al. Critical role of IRF1 and BATF in forming chromatin landscape during type 1 regulatory cell differentiation. Nat Immunol. 2017;18(4):412-21.

6. Shan Q, Zeng Z, Xing S, Li F, Hartwig SM, Gullicksrud JA, et al. The transcription factor Runx3 guards cytotoxic CD8(+) effector T cells against deviation towards follicular helper T cell lineage. Nat Immunol. 2017;18(8):931-9.

7. Serroukh Y, Gu-Trantien C, Hooshiar Kashani B, Defrance M, Vu Manh TP, Azouz A, et al. The transcription factors Runx3 and ThPOK cross-regulate acquisition of cytotoxic function by human Th1 lymphocytes. Elife. 2018;7.

8. Adoro S, Park KH, Bettigole SE, Lis R, Shin HR, Seo H, et al. Post-translational control of T cell development by the ESCRT protein CHMP5. Nat Immunol. 2017;18(7):780-90.

9. Wi SM, Min Y, Lee K-Y. Charged MVB protein 5 is involved in T-cell receptor signaling. Experimental & Molecular Medicine. 2016;48(1):e206-e.

10. Crompton JG, Narayanan M, Cuddapah S, Roychoudhuri R, Ji Y, Yang W, et al. Lineage relationship of CD8(+) T cell subsets is revealed by progressive changes in the epigenetic landscape. Cell Mol Immunol. 2016;13(4):502-13.

11. Yang X, Xia R, Yue C, Zhai W, Du W, Yang Q, et al. ATF4 Regulates CD4+ T Cell Immune Responses through Metabolic Reprogramming. Cell Reports. 2018;23(6):1754-66.

12. Quintana FJ, Solomon A, Cohen IR, Nussbaum G. Induction of IgG3 to LPS via Toll-like receptor 4 co-stimulation. PLoS One. 2008;3(10):e3509.

13. Campbell KS, Cooper S, Dessing M, Yates S, Buder A. Interaction of p59fyn kinase with the dynein light chain, Tctex-1, and colocalization during cytokinesis. J Immunol. 1998;161(4):1728-37.

14. Waugh KA, Leach SM, Moore BL, Bruno TC, Buhrman JD, Slansky JE. Molecular Profile of Tumor-Specific CD8+ T Cell Hypofunction in a Transplantable Murine Cancer Model. J Immunol. 2016;197(4):1477-88.

15. Toft-Hansen H, Nuttall RK, Edwards DR, Owens T. Key metalloproteinases are expressed by specific cell types in experimental autoimmune encephalomyelitis. J Immunol. 2004;173(8):5209-18.

16. Ramesh R, Kozhaya L, McKevitt K, Djuretic IM, Carlson TJ, Quintero MA, et al. Pro-inflammatory human Th17 cells selectively express P-glycoprotein and are refractory to glucocorticoids. J Exp Med. 2014;211(1):89-104.

17. Page N, Gros F, Schall N, Décossas M, Bagnard D, Briand JP, et al. HSC70 blockade by the therapeutic peptide P140 affects autophagic processes and endogenous MHCII presentation in murine lupus. Ann Rheum Dis. 2011;70(5):837-43.

18. Orfali N, Shan-Krauer D, O'Donovan TR, Mongan NP, Gudas LJ, Cahill MR, et al. Inhibition of UBE2L6 attenuates ISGylation and impedes ATRA-induced differentiation of leukemic cells. Mol Oncol. 2020;14(6):1297-309.

19. Barbarulo A, Iansante V, Chaidos A, Naresh K, Rahemtulla A, Franzoso G, et al. Poly(ADP-ribose) polymerase family member 14 (PARP14) is a novel effector of the JNK2-dependent pro-survival signal in multiple myeloma. Oncogene. 2013;32(36):4231-42.

20. Cho SH, Goenka S, Henttinen T, Gudapati P, Reinikainen A, Eischen CM, et al. PARP-14, a member of the B aggressive lymphoma family, transduces survival signals in primary B cells. Blood. 2009;113(11):2416-25.

21. Cho SH, Ahn AK, Bhargava P, Lee CH, Eischen CM, McGuinness O, et al. Glycolytic rate and lymphomagenesis depend on PARP14, an ADP ribosyltransferase of the B aggressive lymphoma (BAL) family. Proc Natl Acad Sci U S A. 2011;108(38):15972-7.

22. Mehrotra P, Krishnamurthy P, Sun J, Goenka S, Kaplan MH. Poly-ADP-ribosyl polymerase-14 promotes T helper 17 and follicular T helper development. Immunology. 2015;146(4):537-46.

23. Sugiura A, Andrejeva G, Voss K, Heintzman DR, Xu X, Madden MZ, et al. MTHFD2 is a metabolic checkpoint controlling effector and regulatory T cell fate and function. Immunity. 2021.

24. O'Brien M, Lonergan R, Costelloe L, O'Rourke K, Fletcher JM, Kinsella K, et al. OAS1: a multiple sclerosis susceptibility gene that influences disease severity. Neurology. 2010;75(5):411-8.

25. Yu L, Ma H, Ji X, Volkert MR. The Sub1 nuclear protein protects DNA from oxidative damage. Mol Cell Biochem. 2016;412(1-2):165-71.

26. Pesu M, Watford WT, Wei L, Xu L, Fuss I, Strober W, et al. T-cell-expressed proprotein convertase furin is essential for maintenance of peripheral immune tolerance. Nature. 2008;455(7210):246-50.

27. Ortutay Z, Oksanen A, Aittomäki S, Ortutay C, Pesu M. Proprotein convertase FURIN regulates T cell receptor-induced transactivation. J Leukoc Biol. 2015;98(1):73-83.

28. Shiryaev SA, Remacle AG, Savinov AY, Chernov AV, Cieplak P, Radichev IA, et al. Inflammatory proprotein convertase-matrix metalloproteinase proteolytic pathway in antigen-presenting cells as a step to autoimmune multiple sclerosis. J Biol Chem. 2009;284(44):30615-26.

29. Yamada M, Hayashi H, Yuuki M, Matsushima N, Yuan B, Takagi N. Furin inhibitor protects against neuronal cell death induced by activated NMDA receptors. Sci Rep. 2018;8(1):5212.

30. Porcellini S, Traggiai E, Schenk U, Ferrera D, Matteoli M, Lanzavecchia A, et al. Regulation of peripheral T cell activation by calreticulin. J Exp Med. 2006;203(2):461-71.

31. de Bruyn M, Wiersma VR, Helfrich W, Eggleton P, Bremer E. The ever-expanding immunomodulatory role of calreticulin in cancer immunity. Front Oncol. 2015;5:35.

32. M NF, McMahon J, Reynolds R, Connolly D, Higgins E, Counihan T, et al. Calreticulin and other components of endoplasmic reticulum stress in rat and human inflammatory demyelination. Acta Neuropathol Commun. 2013;1:37.

33. Adam I, Dewi DL, Mooiweer J, Sadik A, Mohapatra SR, Berdel B, et al. Upregulation of tryptophanyl-tRNA synthethase adapts human cancer cells to nutritional stress caused by tryptophan degradation. Oncoimmunology. 2018;7(12):e1486353.

34. Nguyen TTT, Yoon HK, Kim YT, Choi YH, Lee WK, Jin M. Tryptophanyl-tRNA Synthetase 1 Signals Activate TREM-1 via TLR2 and TLR4. Biomolecules. 2020;10(9).

35. Qin R, Zhao C, Wang CJ, Xu W, Zhao JY, Lin Y, et al. Tryptophan potentiates CD8(+) T cells against cancer cells by TRIP12 tryptophanylation and surface PD-1 downregulation. J Immunother Cancer. 2021;9(7).

36. Salem M, Mony JT, Løbner M, Khorooshi R, Owens T. Interferon regulatory factor-7 modulates experimental autoimmune encephalomyelitis in mice. J Neuroinflammation. 2011;8:181.

37. Jefferies CA. Regulating IRFs in IFN Driven Disease. Front Immunol. 2019;10:325.

38. Peng M, Yin N, Chhangawala S, Xu K, Leslie CS, Li MO. Aerobic glycolysis promotes T helper 1 cell differentiation through an epigenetic mechanism. Science. 2016;354(6311):481-4.

39. Xu K, Yin N, Peng M, Stamatiades EG, Chhangawala S, Shyu A, et al. Glycolytic ATP fuels phosphoinositide 3-kinase signaling to support effector T helper 17 cell responses. Immunity. 2021;54(5):976-87.e7.

40. Jeong SI, Kim JW, Ko KP, Ryu BK, Lee MG, Kim HJ, et al. XAF1 forms a positive feedback loop with IRF-1 to drive apoptotic stress response and suppress tumorigenesis. Cell Death Dis. 2018;9(8):806.

41. Shin CH, Lee MG, Han J, Jeong SI, Ryu BK, Chi SG. Identification of XAF1-MT2A mutual antagonism as a molecular switch in cell-fate decisions under stressful conditions. Proc Natl Acad Sci U S A. 2017;114(22):5683-8.

42. Zehntner SP, Bourbonnière L, Moore CS, Morris SJ, Methot D, St Jean M, et al. X-linked inhibitor of apoptosis regulates T cell effector function. J Immunol. 2007;179(11):7553-60.

43. Moore CS, Hebb AL, Blanchard MM, Crocker CE, Liston P, Korneluk RG, et al. Increased X-linked inhibitor of apoptosis protein (XIAP) expression exacerbates experimental autoimmune encephalomyelitis (EAE). J Neuroimmunol. 2008;203(1):79-93.

44. Gotoh T, Terada K, Oyadomari S, Mori M. hsp70-DnaJ chaperone pair prevents nitric oxide- and CHOP-induced apoptosis by inhibiting translocation of Bax to mitochondria. Cell Death Differ. 2004;11(4):390-402.

45. Mansilla MJ, Montalban X, Espejo C. Heat shock protein 70: roles in multiple sclerosis. Mol Med. 2012;18(1):1018-28.

46. Tukaj S, Kotlarz A, Jozwik A, Smolenska Z, Bryl E, Witkowski JM, et al. Hsp40 proteins modulate humoral and cellular immune response in rheumatoid arthritis patients. Cell Stress Chaperones. 2010;15(5):555-66.

47. Kumar V. The Trinity of cGAS, TLR9, and ALRs Guardians of the Cellular Galaxy Against Host-Derived Self-DNA. Front Immunol. 2020;11:624597.

48. Fujii Y, Shiota M, Ohkawa Y, Baba A, Wanibuchi H, Kinashi T, et al. Surf4 modulates STIM1-dependent calcium entry. Biochem Biophys Res Commun. 2012;422(4):615-20.

49. Emmer BT, Hesketh GG, Kotnik E, Tang VT, Lascuna PJ, Xiang J, et al. The cargo receptor SURF4 promotes the efficient cellular secretion of PCSK9. Elife. 2018;7.

50. Wang X, Wang H, Xu B, Huang D, Nie C, Pu L, et al. Receptor-Mediated ER Export of Lipoproteins Controls Lipid Homeostasis in Mice and Humans. Cell Metab. 2021;33(2):350-66.e7.

51. Cervantes-Gracia K, Husi H. Integrative analysis of Multiple Sclerosis using a systems biology approach. Sci Rep. 2018;8(1):5633.

52. Creanza TM, Liguori M, Liuni S, Nuzziello N, Ancona N. Meta-Analysis of Differential Connectivity in Gene Co-Expression Networks in Multiple Sclerosis. Int J Mol Sci. 2016;17(6).

53. Comabella M, Sastre-Garriga J, Borras E, Villar LM, Saiz A, Martínez-Yélamos S, et al. CSF Chitinase 3-Like 2 Is Associated With Long-term Disability Progression in Patients With Progressive Multiple Sclerosis. Neurol Neuroimmunol Neuroinflamm. 2021;8(6).

54. Palau N, Julià A, Ferrándiz C, Puig L, Fonseca E, Fernández E, et al. Genome-wide transcriptional analysis of T cell activation reveals differential gene expression associated with psoriasis. BMC Genomics. 2013;14(1):825.

55. Hu D, Notarbartolo S, Croonenborghs T, Patel B, Cialic R, Yang TH, et al. Transcriptional signature of human pro-inflammatory TH17 cells identifies reduced IL10 gene expression in multiple sclerosis. Nat Commun. 2017;8(1):1600.

56. Seyhan AA, Gregory B, Cribbs AP, Bhalara S, Li Y, Loreth C, et al. Novel biomarkers of a peripheral blood interferon signature associated with drug-naïve early arthritis patients distinguish persistent from self-limiting disease course. Scientific Reports. 2020;10(1):8830.

57. Sun JL, Zhang HZ, Liu SY, Lian CF, Chen ZL, Shao TH, et al. Elevated EPSTI1 promote B cell hyperactivation through NF-κB signalling in patients with primary Sjögren's syndrome. Ann Rheum Dis. 2020;79(4):518-24.

58. Bai D, Du J, Bu X, Cao W, Sun T, Zhao J, et al. ALDOA maintains NLRP3 inflammasome activation by controlling AMPK activation. Autophagy. 2021:1-21.

59. Volchenkov R, Nygaard V, Sener Z, Skålhegg BS. Th17 Polarization under Hypoxia Results in Increased IL-10 Production in a Pathogen-Independent Manner. Front Immunol. 2017;8:698.

60. Hofmann SR, Carlsson E, Kapplusch F, Carvalho AL, Liloglou T, Schulze F, et al. Cyclic AMP Response Element Modulator-α Suppresses PD-1 Expression and Promotes Effector CD4(+) T Cells in Psoriasis. J Immunol. 2021;207(1):55-64.

61. Rauen T, Hedrich CM, Tenbrock K, Tsokos GC. cAMP responsive element modulator: a critical regulator of cytokine production. Trends Mol Med. 2013;19(4):262-9.

62. Perng YC, Lenschow DJ. ISG15 in antiviral immunity and beyond. Nat Rev Microbiol. 2018;16(7):423-39.

63. Oveland E, Ahmad I, Lereim RR, Kroksveen AC, Barsnes H, Guldbrandsen A, et al. Cuprizone and EAE mouse frontal cortex proteomics revealed proteins altered in multiple sclerosis. Sci Rep. 2021;11(1):7174.

64. Zuehlke AD, Beebe K, Neckers L, Prince T. Regulation and function of the human HSP90AA1 gene. Gene. 2015;570(1):8-16.

65. Ofengeim D, Ito Y, Najafov A, Zhang Y, Shan B, DeWitt JP, et al. Activation of necroptosis in multiple sclerosis. Cell Rep. 2015;10(11):1836-49.

66. Schirmer L, Velmeshev D, Holmqvist S, Kaufmann M, Werneburg S, Jung D, et al. Neuronal vulnerability and multilineage diversity in multiple sclerosis. Nature. 2019;573(7772):75-82.

67. Miao Y, Bhushan J, Dani A, Vig M. Na(+) influx via Orai1 inhibits intracellular ATP-induced mTORC2 signaling to disrupt CD4 T cell gene expression and differentiation. Elife. 2017;6.

68. Vidmar L, Maver A, Drulović J, Sepčić J, Novaković I, Ristič S, et al. Multiple Sclerosis patients carry an increased burden of exceedingly rare genetic variants in the inflammasome regulatory genes. Sci Rep. 2019;9(1):9171.

69. Berge T, Eriksson A, Brorson IS, Høgestøl EA, Berg-Hansen P, Døskeland A, et al. Quantitative proteomic analyses of CD4(+) and CD8(+) T cells reveal differentially expressed proteins in multiple sclerosis patients and healthy controls. Clin Proteomics. 2019;16:19.

70. Gal-Ben-Ari S, Barrera I, Ehrlich M, Rosenblum K. PKR: A Kinase to Remember. Front Mol Neurosci. 2018;11:480.

71. McGuire VA, Ruiz-Zorrilla Diez T, Emmerich CH, Strickson S, Ritorto MS, Sutavani RV, et al. Dimethyl fumarate blocks pro-inflammatory cytokine production via inhibition of TLR induced M1 and K63 ubiquitin chain formation. Sci Rep. 2016;6:31159.

72. Liu Z, Xu L. UBE2S promotes the proliferation and survival of human lung adenocarcinoma cells. BMB Rep. 2018;51(12):642-7.

73. Zhang RY, Liu ZK, Wei D, Yong YL, Lin P, Li H, et al. UBE2S interacting with TRIM28 in the nucleus accelerates cell cycle by ubiquitination of p27 to promote hepatocellular carcinoma development. Signal Transduct Target Ther. 2021;6(1):64.

74. Liao W, Lin JX, Wang L, Li P, Leonard WJ. Modulation of cytokine receptors by IL-2 broadly regulates differentiation into helper T cell lineages. Nat Immunol. 2011;12(6):551-9.

75. Hartmann FJ, Khademi M, Aram J, Ammann S, Kockum I, Constantinescu C, et al. Multiple sclerosis-associated IL2RA polymorphism controls GM-CSF production in human TH cells. Nature communications. 2014;5:5056.

76. Sawcer S, Hellenthal G, Pirinen M, Spencer CCA, Patsopoulos NA, Moutsianas L, et al. Genetic risk and a primary role for cell-mediated immune mechanisms in multiple sclerosis. Nature. 2011;476(7359):214-9.

77. Buhelt S, Søndergaard HB, Oturai A, Ullum H, von Essen MR, Sellebjerg F. Relationship between Multiple Sclerosis-Associated IL2RA Risk Allele Variants and Circulating T Cell Phenotypes in Healthy Genotype-Selected Controls. Cells. 2019;8(6).

78. Bielekova B. Daclizumab Therapy for Multiple Sclerosis. Cold Spring Harb Perspect Med. 2019;9(5).

79. Zheleznyakova GY, Piket E, Needhamsen M, Hagemann-Jensen M, Ekman D, Han Y, et al. Small noncoding RNA profiling across cellular and biofluid compartments and their implications for multiple sclerosis immunopathology. Proceedings of the National Academy of Sciences. 2021;118(17):e2011574118.

80. Chou WC, Guo Z, Guo H, Chen L, Zhang G, Liang K, et al. AIM2 in regulatory T cells restrains autoimmune diseases. Nature. 2021;591(7849):300-5.

81. Yu Y, Fu P, Yu Z, Xie M, Wang W, Luo X. NKCC1 Inhibition Attenuates Chronic Cerebral Hypoperfusion-Induced White Matter Lesions by Enhancing Progenitor Cells of Oligodendrocyte Proliferation. J Mol Neurosci. 2018.

82. Wang P, Li Q, Dong X, An H, Li J, Zhao L, et al. Lipocalin-type prostaglandin D synthase levels increase in patients with narcolepsy and idiopathic hypersomnia. Sleep. 2021;44(4).

83. Kannaian B, Sharma B, Phillips M, Chowdhury A, Manimekalai MSS, Adav SS, et al. Abundant neuroprotective chaperone Lipocalin-type prostaglandin D synthase (L-PGDS) disassembles the Amyloid-β fibrils. Sci Rep. 2019;9(1):12579.

84. Sakry D, Yigit H, Dimou L, Trotter J. Oligodendrocyte precursor cells synthesize neuromodulatory factors. PLoS One. 2015;10(5):e0127222.

85. Evans DT, Serra-Moreno R, Singh RK, Guatelli JC. BST-2/tetherin: a new component of the innate immune response to enveloped viruses. Trends Microbiol. 2010;18(9):388-96.

86. Ishikawa J, Kaisho T, Tomizawa H, Lee BO, Kobune Y, Inazawa J, et al. Molecular cloning and chromosomal mapping of a bone marrow stromal cell surface gene, BST2, that may be involved in pre-B-cell growth. Genomics. 1995;26(3):527-34.

87. Vidal-Laliena M, Romero X, March S, Requena V, Petriz J, Engel P. Characterization of antibodies submitted to the B cell section of the 8th Human Leukocyte Differentiation Antigens Workshop by flow cytometry and immunohistochemistry. Cell Immunol. 2005;236(1-2):6-16.

88. Crabos M, Yamakado T, Heizmann CW, Cerletti N, Bühler FR, Erne P. The calcium binding protein tropomyosin in human platelets and cardiac tissue: elevation in hypertensive cardiac hypertrophy. Eur J Clin Invest. 1991;21(5):472-8.

89. Guven K, Gunning P, Fath T. TPM3 and TPM4 gene products segregate to the postsynaptic region of central nervous system synapses. Bioarchitecture. 2011;1(6):284-9.

90. Qi Y, Xiong W, Liu W, Fang H, Lu W. Experimental Study of the Flexural and Compression Performance of an Innovative Pultruded Glass-Fiber-Reinforced Polymer-Wood Composite Profile. PLoS One. 2015;10(10):e0140893.

91. Sajid M, Ullah H, Yan K, He M, Feng J, Shereen MA, et al. The Functional and Antiviral Activity of Interferon Alpha-Inducible IFI6 Against Hepatitis B Virus Replication and Gene Expression. Front Immunol. 2021;12:634937.

92. Ding D, Valdivia AO, Bhattacharya SK. Nuclear prelamin a recognition factor and iron dysregulation in multiple sclerosis. Metab Brain Dis. 2020;35(2):275-82.

93. Barton RM, Worman HJ. Prenylated prelamin A interacts with Narf, a novel nuclear protein. J Biol Chem. 1999;274(42):30008-18.

94. Schroder K, Hertzog PJ, Ravasi T, Hume DA. Interferon-gamma: an overview of signals, mechanisms and functions. J Leukoc Biol. 2004;75(2):163-89.

95. Arellano G, Ottum PA, Reyes LI, Burgos PI, Naves R. Stage-Specific Role of Interferon-Gamma in Experimental Autoimmune Encephalomyelitis and Multiple Sclerosis. Front Immunol. 2015;6:492.

96. Gonzalez-Gronow M, Gopal U, Austin RC, Pizzo SV. Glucose-regulated protein (GRP78) is an important cell surface receptor for viral invasion, cancers, and neurological disorders. IUBMB Life. 2021;73(6):843-54.

97. Kern J, Untergasser G, Zenzmaier C, Sarg B, Gastl G, Gunsilius E, et al. GRP-78 secreted by tumor cells blocks the antiangiogenic activity of bortezomib. Blood. 2009;114(18):3960-7.

98. Guenzi E, Töpolt K, Cornali E, Lubeseder-Martellato C, Jörg A, Matzen K, et al. The helical domain of GBP-1 mediates the inhibition of endothelial cell proliferation by inflammatory cytokines. Embo j. 2001;20(20):5568-77.

99. Honkala AT, Tailor D, Malhotra SV. Guanylate-Binding Protein 1: An Emerging Target in Inflammation and Cancer. Frontiers in Immunology. 2020;10(3139).

100. Lew DJ, Decker T, Strehlow I, Darnell JE. Overlapping elements in the guanylate-binding protein gene promoter mediate transcriptional induction by alpha and gamma interferons. Mol Cell Biol. 1991;11(1):182-91.

101. Kristiansen H, Gad HH, Eskildsen-Larsen S, Despres P, Hartmann R. The oligoadenylate synthetase family: an ancient protein family with multiple antiviral activities. J Interferon Cytokine Res. 2011;31(1):41-7.

102. Son H, Kim S, Jung DH, Baek JH, Lee DH, Roh GS, et al. Insufficient glutamine synthetase activity during synaptogenesis causes spatial memory impairment in adult mice. Sci Rep. 2019;9(1):252.

103. Eelen G, Dubois C, Cantelmo AR, Goveia J, Brüning U, DeRan M, et al. Role of glutamine synthetase in angiogenesis beyond glutamine synthesis. Nature. 2018;561(7721):63-9.

104. Suárez I, Bodega G, Fernández B. Glutamine synthetase in brain: effect of ammonia. Neurochem Int. 2002;41(2-3):123-42.

105. Wang T, Lee MH, Choi E, Pardo-Villamizar CA, Lee SB, Yang IH, et al. Granzyme B-induced neurotoxicity is mediated via activation of PAR-1 receptor and Kv1.3 channel. PLoS One. 2012;7(8):e43950.

106. Poe M, Blake JT, Boulton DA, Gammon M, Sigal NH, Wu JK, et al. Human cytotoxic lymphocyte granzyme B. Its purification from granules and the characterization of substrate and inhibitor specificity. J Biol Chem. 1991;266(1):98-103.

107. Zhang Z, Zhang Y, Xia S, Kong Q, Li S, Liu X, et al. Gasdermin E suppresses tumour growth by activating anti-tumour immunity. Nature. 2020;579(7799):415-20.

108. Liu Y, Fang Y, Chen X, Wang Z, Liang X, Zhang T, et al. Gasdermin E-mediated target cell pyroptosis by CAR T cells triggers cytokine release syndrome. Sci Immunol. 2020;5(43).

109. Hofer S, Pfeil K, Niederegger H, Ebner S, Nguyen VA, Kremmer E, et al. Dendritic cells regulate T-cell deattachment through the integrin-interacting protein CYTIP. Blood. 2006;107(3):1003-9.

110. Boehm T, Hofer S, Winklehner P, Kellersch B, Geiger C, Trockenbacher A, et al. Attenuation of cell adhesion in lymphocytes is regulated by CYTIP, a protein which mediates signal complex sequestration. Embo j. 2003;22(5):1014-24.

111. Heib V, Sparber F, Tripp CH, Ortner D, Stoitzner P, Heufler C. Cytip regulates dendritic-cell function in contact hypersensitivity. Eur J Immunol. 2012;42(3):589-97.

112. Verma S, Goyal S, Jamal S, Singh A, Grover A. Hsp90: Friends, clients and natural foes. Biochimie. 2016;127:227-40.

113. Pearl LH. Review: The HSP90 molecular chaperone-an enigmatic ATPase. Biopolymers. 2016;105(8):594-607.

114. Haase M, Fitze G. HSP90AB1: Helping the good and the bad. Gene. 2016;575(2 Pt 1):171-86.

115. Baker BJ, Akhtar LN, Benveniste EN. SOCS1 and SOCS3 in the control of CNS immunity. Trends Immunol. 2009;30(8):392-400.

116. Liau NPD, Laktyushin A, Lucet IS, Murphy JM, Yao S, Whitlock E, et al. The molecular basis of JAK/STAT inhibition by SOCS1. Nature Communications. 2018;9(1):1558.

117. Cuvertino S, Stuart HM, Chandler KE, Roberts NA, Armstrong R, Bernardini L, et al. ACTB Loss-of-Function Mutations Result in a Pleiotropic Developmental Disorder. Am J Hum Genet. 2017;101(6):1021-33.

118. Cheever TR, Ervasti JM. Actin isoforms in neuronal development and function. Int Rev Cell Mol Biol. 2013;301:157-213.

119. Jian D, Wang W, Zhou X, Jia Z, Wang J, Yang M, et al. Interferon-induced protein 35 inhibits endothelial cell proliferation, migration and re-endothelialization of injured arteries by inhibiting the nuclear factor-kappa B pathway. Acta Physiologica. 2018;223(3):e13037.

120. Xiahou Z, Wang X, Shen J, Zhu X, Xu F, Hu R, et al. NMI and IFP35 serve as proinflammatory DAMPs during cellular infection and injury. Nature Communications. 2017;8(1):950.

121. López-Alemany R, Longstaff C, Hawley S, Mirshahi M, Fábregas P, Jardí M, et al. Inhibition of cell surface mediated plasminogen activation by a monoclonal antibody against alpha-Enolase. Am J Hematol. 2003;72(4):234-42.

122. Ji H, Wang J, Guo J, Li Y, Lian S, Guo W, et al. Progress in the biological function of alpha-enolase. Anim Nutr. 2016;2(1):12-7.

123. Tahvanainen J, Kallonen T, Lähteenmäki H, Heiskanen KM, Westermarck J, Rao KV, et al. PRELI is a mitochondrial regulator of human primary T-helper cell apoptosis, STAT6, and Th2-cell differentiation. Blood. 2009;113(6):1268-77.

124. Gillen AE, Brechbuhl HM, Yamamoto TM, Kline E, Pillai MM, Hesselberth JR, et al. Alternative Polyadenylation of PRELID1 Regulates Mitochondrial ROS Signaling and Cancer Outcomes. Mol Cancer Res. 2017;15(12):1741-51.

125. Verhelst J, Parthoens E, Schepens B, Fiers W, Saelens X. Interferon-inducible protein Mx1 inhibits influenza virus by interfering with functional viral ribonucleoprotein complex assembly. J Virol. 2012;86(24):13445-55.

126. Ortiz E, Sanchis P, Bizzotto J, Lage-Vickers S, Labanca E, Navone N, et al. Myxovirus Resistance Protein 1 (MX1), a Novel HO-1 Interactor, Tilts the Balance of Endoplasmic Reticulum Stress towards Pro-Death Events in Prostate Cancer. Biomolecules. 2020;10(7).

127. Tumpey TM, Szretter KJ, Van Hoeven N, Katz JM, Kochs G, Haller O, et al. The Mx1 gene protects mice against the pandemic 1918 and highly lethal human H5N1 influenza viruses. J Virol. 2007;81(19):10818-21.

128. Zhao X, Zheng S, Chen D, Zheng M, Li X, Li G, et al. LY6E Restricts Entry of Human Coronaviruses, Including Currently Pandemic SARS-CoV-2. J Virol. 2020;94(18).

129. Pfaender S, Mar KB, Michailidis E, Kratzel A, Boys IN, V'Kovski P, et al. LY6E impairs coronavirus fusion and confers immune control of viral disease. Nat Microbiol. 2020;5(11):1330-9.

130. Saitoh S, Kosugi A, Noda S, Yamamoto N, Ogata M, Minami Y, et al. Modulation of TCR-mediated signaling pathway by thymic shared antigen-1 (TSA-1)/stem cell antigen-2 (Sca-2). J Immunol. 1995;155(12):5574-81.

131. Zhang X, Yan C, Hang J, Finci LI, Lei J, Shi Y. An Atomic Structure of the Human Spliceosome. Cell. 2017;169(5):918-29.e14.

132. Zou Y, Zhang WF, Liu HY, Li X, Zhang X, Ma XF, et al. Structure and function of the contactin-associated protein family in myelinated axons and their relationship with nerve diseases. Neural Regen Res. 2017;12(9):1551-8.

133. Zhao XF, Nowak NJ, Shows TB, Aplan PD. MAGOH interacts with a novel RNA-binding protein. Genomics. 2000;63(1):145-8.

134. Loeffen J, Smeets R, Smeitink J, Triepels R, Sengers R, Trijbels F, et al. The human NADH: ubiquinone oxidoreductase NDUFS5 (15 kDa) subunit: cDNA cloning, chromosomal localization, tissue distribution and the absence of mutations in isolated complex I-deficient patients. J Inherit Metab Dis. 1999;22(1):19-28.

135. Vyshkina T, Banisor I, Shugart YY, Leist TP, Kalman B. Genetic variants of Complex I in multiple sclerosis. J Neurol Sci. 2005;228(1):55-64.

136. He X, Huang Q, Qiu X, Liu X, Sun G, Guo J, et al. LAP3 promotes glioma progression by regulating proliferation, migration and invasion of glioma cells. Int J Biol Macromol. 2015;72:1081-9.

137. Jösch C, Klotz LO, Sies H. Identification of cytosolic leucyl aminopeptidase (EC 3.4.11.1) as the major cysteinylglycine-hydrolysing activity in rat liver. Biol Chem. 2003;384(2):213-8.

138. Cappiello M, Lazzarotti A, Buono F, Scaloni A, D'Ambrosio C, Amodeo P, et al. New role for leucyl aminopeptidase in glutathione turnover. Biochem J. 2004;378(Pt 1):35-44.

139. Voges D, Zwickl P, Baumeister W. The 26S proteasome: a molecular machine designed for controlled proteolysis. Annu Rev Biochem. 1999;68:1015-68.

140. Demartino GN, Gillette TG. Proteasomes: machines for all reasons. Cell. 2007;129(4):659-62.

141. Tanaka K, Kasahara M. The MHC class I ligand-generating system: roles of immunoproteasomes and the interferon-gamma-inducible proteasome activator PA28. Immunol Rev. 1998;163:161-76.

142. Jacquot JP, de Lamotte F, Fontecave M, Schürmann P, Decottignies P, Miginiac-Maslow M, et al. Human thioredoxin reactivity-structure/function relationship. Biochem Biophys Res Commun. 1990;173(3):1375-81.

143. Mitchell DA, Marletta MA. Thioredoxin catalyzes the S-nitrosation of the caspase-3 active site cysteine. Nat Chem Biol. 2005;1(3):154-8.

144. Wei SJ, Botero A, Hirota K, Bradbury CM, Markovina S, Laszlo A, et al. Thioredoxin nuclear translocation and interaction with redox factor-1 activates the activator protein-1 transcription factor in response to ionizing radiation. Cancer Res. 2000;60(23):6688-95.

145. Sijts A, Sun Y, Janek K, Kral S, Paschen A, Schadendorf D, et al. The role of the proteasome activator PA28 in MHC class I antigen processing. Mol Immunol. 2002;39(3-4):165-9.

146. Tezuka Y, Okada M, Tada Y, Yamauchi J, Nishigori H, Sanbe A. Regulation of neurite growth by inorganic pyrophosphatase 1 via JNK dephosphorylation. PLoS One. 2013;8(4):e61649.

147. Luo D, Liu D, Shi W, Jiang H, Liu W, Zhang X, et al. PPA1 promotes NSCLC progression via a JNK- and TP53-dependent manner. Oncogenesis. 2019;8(10):53.

148. Mishra DR, Chaudhary S, Krishna BM, Mishra SK. Identification of Critical Elements for Regulation of Inorganic Pyrophosphatase (PPA1) in MCF7 Breast Cancer Cells. PLoS One. 2015;10(4):e0124864.

149. Niu H, Zhou W, Xu Y, Yin Z, Shen W, Ye Z, et al. Silencing PPA1 inhibits human epithelial ovarian cancer metastasis by suppressing the Wnt/β-catenin signaling pathway. Oncotarget. 2017;8(44):76266-78.

150. Nadeau S, Hein P, Fernandes KJ, Peterson AC, Miller FD. A transcriptional role for C/EBP beta in the neuronal response to axonal injury. Mol Cell Neurosci. 2005;29(4):525-35.

151. Chinery R, Brockman JA, Dransfield DT, Coffey RJ. Antioxidant-induced nuclear translocation of CCAAT/enhancer-binding protein beta. A critical role for protein kinase A-mediated phosphorylation of Ser299. J Biol Chem. 1997;272(48):30356-61.

152. Tsukada J, Yoshida Y, Kominato Y, Auron PE. The CCAAT/enhancer (C/EBP) family of basic-leucine zipper (bZIP) transcription factors is a multifaceted highly-regulated system for gene regulation. Cytokine. 2011;54(1):6-19.

153. Lock C, Hermans G, Pedotti R, Brendolan A, Schadt E, Garren H, et al. Gene-microarray analysis of multiple sclerosis lesions yields new targets validated in autoimmune encephalomyelitis. Nat Med. 2002;8(5):500-8.

154. Zhang Y, Mao D, Roswit WT, Jin X, Patel AC, Patel DA, et al. PARP9-DTX3L ubiquitin ligase targets host histone H2BJ and viral 3C protease to enhance interferon signaling and control viral infection. Nat Immunol. 2015;16(12):1215-27.

155. Iwata H, Goettsch C, Sharma A, Ricchiuto P, Goh WW, Halu A, et al. PARP9 and PARP14 cross-regulate macrophage activation via STAT1 ADP-ribosylation. Nat Commun. 2016;7:12849.

156. Yan Q, Xu R, Zhu L, Cheng X, Wang Z, Manis J, et al. BAL1 and its partner E3 ligase, BBAP, link Poly(ADP-ribose) activation, ubiquitylation, and double-strand DNA repair independent of ATM, MDC1, and RNF8. Mol Cell Biol. 2013;33(4):845-57.

157. Ke Y, Wang C, Zhang J, Zhong X, Wang R, Zeng X, et al. The Role of PARPs in Inflammation-and Metabolic-Related Diseases: Molecular Mechanisms and Beyond. Cells. 2019;8(9).

158. Ling XB, Wei HW, Wang J, Kong YQ, Wu YY, Guo JL, et al. Mammalian Metallothionein-2A and Oxidative Stress. Int J Mol Sci. 2016;17(9).

159. Jakovac H, Tota M, Grebic D, Grubic-Kezele T, Barac-Latas V, Mrakovcic-Sutic I, et al. Metallothionein I+II expression as an early sign of chronic relapsing experimental autoimmune encephalomyelitis in rats. Curr Aging Sci. 2013;6(1):37-44.

160. Trendelenburg G, Prass K, Priller J, Kapinya K, Polley A, Muselmann C, et al. Serial analysis of gene expression identifies metallothionein-II as major neuroprotective gene in mouse focal cerebral ischemia. J Neurosci. 2002;22(14):5879-88.

161. van Wijk E, Krieger E, Kemperman MH, De Leenheer EM, Huygen PL, Cremers CW, et al. A mutation in the gamma actin 1 (ACTG1) gene causes autosomal dominant hearing loss (DFNA20/26). J Med Genet. 2003;40(12):879-84.

162. Rivière JB, van Bon BW, Hoischen A, Kholmanskikh SS, O'Roak BJ, Gilissen C, et al. De novo mutations in the actin genes ACTB and ACTG1 cause Baraitser-Winter syndrome. Nat Genet. 2012;44(4):440-4, s1-2.

163. Sato S, Noda S, Torii S, Amo T, Ikeda A, Funayama M, et al. Homeostatic p62 levels and inclusion body formation in CHCHD2 knockout mice. Hum Mol Genet. 2021;30(6):443-53.

164. Liu Y, Levine B. Autosis and autophagic cell death: the dark side of autophagy. Cell Death Differ. 2015;22(3):367-76.

165. Aras S, Bai M, Lee I, Springett R, Hüttemann M, Grossman LI. MNRR1 (formerly CHCHD2) is a bi-organellar regulator of mitochondrial metabolism. Mitochondrion. 2015;20:43-51.

166. Funayama M, Ohe K, Amo T, Furuya N, Yamaguchi J, Saiki S, et al. CHCHD2 mutations in autosomal dominant late-onset Parkinson's disease: a genome-wide linkage and sequencing study. The Lancet Neurology. 2015;14(3):274-82.

167. Boyman O, Sprent J. The role of interleukin-2 during homeostasis and activation of the immune system. Nat Rev Immunol. 2012;12(3):180-90.

168. Ratthé C, Girard D. Interleukin-15 enhances human neutrophil phagocytosis by a Syk-dependent mechanism: importance of the IL-15Ralpha chain. J Leukoc Biol. 2004;76(1):162-8.

169. Cavanillas ML, Alcina A, Núñez C, de las Heras V, Fernández-Arquero M, Bartolomé M, et al. Polymorphisms in the IL2, IL2RA and IL2RB genes in multiple sclerosis risk. Eur J Hum Genet. 2010;18(7):794-9.

170. Usuki K, Saras J, Waltenberger J, Miyazono K, Pierce G, Thomason A, et al. Platelet-derived endothelial cell growth factor has thymidine phosphorylase activity. Biochem Biophys Res Commun. 1992;184(3):1311-6.

171. Chapouly C, Tadesse Argaw A, Horng S, Castro K, Zhang J, Asp L, et al. Astrocytic TYMP and VEGFA drive blood-brain barrier opening in inflammatory central nervous system lesions. Brain. 2015;138(Pt 6):1548-67.

172. Furukawa T, Yoshimura A, Sumizawa T, Haraguchi M, Akiyama S, Fukui K, et al. Angiogenic factor. Nature. 1992;356(6371):668.

173. Engels K, Fox SB, Whitehouse RM, Gatter KC, Harris AL. Up-regulation of thymidine phosphorylase expression is associated with a discrete pattern of angiogenesis in ductal carcinomas in situ of the breast. J Pathol. 1997;182(4):414-20.

174. Nie H, Maika SD, Tucker PW, Gottlieb PD. A role for SATB1, a nuclear matrix association region-binding protein, in the development of CD8SP thymocytes and peripheral T lymphocytes. J Immunol. 2005;174(8):4745-52.

175. Wang F, Tidei JJ, Polich ED, Gao Y, Zhao H, Perrone-Bizzozero NI, et al. Positive feedback between RNA-binding protein HuD and transcription factor SATB1 promotes neurogenesis. Proc Natl Acad Sci U S A. 2015;112(36):E4995-5004.

176. Kelly A, Powis SH, Glynne R, Radley E, Beck S, Trowsdale J. Second proteasome-related gene in the human MHC class II region. Nature. 1991;353(6345):667-8.

177. Zanker D, Pang K, Oveissi S, Lu C, Faou P, Nowell C, et al. LMP2 immunoproteasome promotes lymphocyte survival by degrading apoptotic BH3-only proteins. Immunol Cell Biol. 2018;96(9):981-93.

178. Bollong MJ, Lee G, Coukos JS, Yun H, Zambaldo C, Chang JW, et al. A metabolite-derived protein modification integrates glycolysis with KEAP1-NRF2 signalling. Nature. 2018;562(7728):600-4.

179. Matsumaru S, Oguni H, Ogura H, Shimojima K, Nagata S, Kanno H, et al. A novel PGK1 mutation associated with neurological dysfunction and the absence of episodes of hemolytic anemia or myoglobinuria. Intractable Rare Dis Res. 2017;6(2):132-6.
